# Photoredox‐Catalyzed Cyclobutane Synthesis by a Deboronative Radical Addition–Polar Cyclization Cascade

**DOI:** 10.1002/anie.201813917

**Published:** 2019-02-15

**Authors:** Chao Shu, Adam Noble, Varinder K. Aggarwal

**Affiliations:** ^1^ School of Chemistry University of Bristol Cantock's Close Bristol BS8 1TS UK

**Keywords:** boronic esters, cascade, cyclobutanes, photoredox catalysis, radical-polar crossover

## Abstract

Photoredox‐catalyzed methylcyclobutanations of alkylboronic esters are described. The reactions proceed through single‐electron transfer induced deboronative radical addition to an electron‐deficient alkene followed by single‐electron reduction and polar 4‐*exo*‐*tet* cyclization with a pendant alkyl halide. Key to the success of the methodology was the use of easily oxidizable arylboronate complexes. Structurally diverse cyclobutanes are shown to be conveniently prepared from readily available alkylboronic esters and a range of haloalkyl alkenes. The mild reactions display excellent functional group tolerance, and the radical addition‐polar cyclization cascade also enables the synthesis of 3‐, 5‐, 6‐, and 7‐membered rings.

Cyclobutanes are highly valuable structural motifs in the chemical sciences. They have found prominence as synthetic intermediates due to their high ring strain and are present in numerous bioactive small molecules (Figure 1 a).^1^, ^2^ In particular, the spatially defined arrangement of substituents imparted by their structural rigidity makes them attractive targets for drug discovery.^3^ Synthetic efforts towards these important small rings have largely focused on [2+2] cycloadditions or ring expansion of cyclopropane derivatives.^4^ Alternative approaches involve 1,4‐cyclization reactions of functionalized alkyl (pseudo)halides,4a such as by enolate alkylation or reductive coupling of a tethered alkene.^5^ We considered a related approach utilizing a photoredox‐catalyzed radical addition‐polar cyclization cascade between a carboxylic acid and a haloalkyl alkene (Figure 1 b, *n*=2),^6^ a process that represents an open‐shell variant of Michael‐induced ring closure (MIRC) reactions.^7^ This would allow a fragment coupling‐based cyclobutanation, in which a cyclobutane ring could be incorporated into a complex molecule in a single step, under mild conditions, by substitution of a carboxylic acid, or another suitable radical precursor.^8^

**Figure 1:**
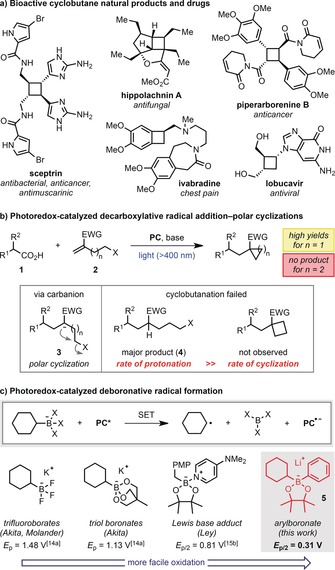
a) Bioactive cyclobutanes. b) Photoredox‐catalyzed decarboxylative radical addition–polar cyclization cascades. c) Boronate complexes as alkyl radical precursors. Reduction potentials are versus SCE in MeCN. EWG=electron‐withdrawing group; PC=photocatalyst; SET=single‐electron transfer; PMP=4‐methoxyphenyl.

We recently reported a photoredox‐catalyzed cyclopropanation and methylcyclopropanation of aliphatic carboxylic acids **1** (Figure 1 b, *n*=1).6a The reaction proceeds by a decarboxylative radical addition to a chloride‐tethered alkene **2** followed by single‐electron reduction and 3‐*exo*‐*tet* cyclization of the resulting carbanion **3**. While this protocol enabled the formation of cyclopropanes and cyclopentanes, a limitation was discovered during our attempts to generate 4‐ and 6‐membered rings. In these cases, the Giese‐type protonated products **4** were obtained instead of the cyclized products,^9^ despite conducting the reactions under rigorously anhydrous conditions. This was attributed to the much slower rates of 4‐ and 6‐*exo*‐*tet* cyclizations,^10^ which resulted in competing protonation of the intermediate carbanion. To prevent the formation of these undesired Giese products, we sought an alternative radical precursor that would enable radical generation under fully aprotic conditions and considered boronic ester derivatives.^11^

We were particularly attracted to arylboronate complexes generated from aryllithium reagents and pinacol boronic esters.^12^ These species offer a number of attractive features that would benefit the proposed cyclobutanation, such as: 1) Pinacol boronic esters are readily available. 2) They can be generated and used in situ under strictly anhydrous conditions, which should inhibit formation of the Giese products. 3) The electron‐rich arylboronate complexes were expected to undergo facile single‐electron oxidation, and this was confirmed by measurement of the reduction potential for arylboronate complex **5** of 0.31 V versus SCE in MeCN (Figure 1 C).^13^ This value is significantly lower than other commonly used boron‐based alkyl radical precursors, such as trifluoroborate salts,^14^ cyclic triol boronates,14a or nitrogen and phosphorus Lewis base complexes,^15^ which can also often suffer from either low solubility, limited availability or limited substrate scope. Herein, we describe a transition metal‐free photoredox‐catalyzed generation of alkyl radicals from arylboronate complexes. These species participate in radical addition–polar cyclization cascades with halide‐tethered alkenes enabling the synthesis of a broad range of functionalized cyclobutanes.

We began our investigation by studying the reaction of cyclohexyl boronic acid pinacol ester (**6**) with iodide‐tethered enoate **7 a** (X=I, Table 1). Arylboronate complex **5** was generated in situ by reaction of **6** with a slight excess of phenyllithium at 0 °C. To the THF solution of **5** was then added enoate **7 a** and 2.0 mol % of the organic photocatalyst 1,2,3,5‐tetrakis(carbazol‐9‐yl)‐4,6‐dicyano‐benzene (4CzIPN),^16^ and the mixture was irradiated with blue LEDs at room temperature for 20 h. Pleasingly, cyclobutane **8** was formed in 45 % yield with none of the undesired Giese product **9** (entry 1). A significant improvement in yield was observed upon performing a solvent switch from THF to MeCN after formation of boronate complex **5**, providing **8** in 70 % yield (entry 2). Changing to alkyl bromide **7 b** (X=Br) resulted in a slightly lower yield of **8**, whereas the corresponding chloride **7 c** (X=Cl) and tosylate **7 d** (X=OTs) only gave Giese products **9 c** and **9 d** (entries 3–5). Evaluation of a range of iridium, ruthenium, and organic photocatalysts did not provide any improvements over 4CzIPN (see the Supporting Information). The catalyst loading was found to have a modest effect on the yield, with 5 mol % proving optimal (entries 6 and 7). The importance of anhydrous conditions was highlighted by the complete reversal of selectivity from cyclobutane to Giese product formation upon addition of water to the reaction (entry 8). Finally, it was found that using DMSO as the solvent provided **8** in similarly high yield to MeCN (entry 9). Control experiments highlighted the importance of phenyllithium activation (entry 10), and no product was observed in the absence of photocatalyst or light (entries 11–12). Interestingly, replacement of arylboronate complex **5** with the corresponding trifluoroborate (entry 13) or a combination of **6** and DMAP (entry 14) failed to give either cyclobutane **8** or the Giese product **9**. Furthermore, submitting enoate **7 a** to the optimized Giese reaction conditions reported by Akita (with BF_3_K salt)14a and Ley (with Bpin and DMAP)15b resulted in no cyclobutane formation.^13^ These results highlight the benefits on reactivity of using easily oxidized arylboronate complexes such as **5**.

**Table 1:** Optimization studies.^[a]^ 
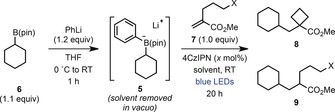

Entry	4CzIPN mol %	Solvent	X	% **8**	% **9**
1	2	THF	I	45	0
2	2	MeCN	I	70	0
3	2	MeCN	Br	62	0
4	2	MeCN	Cl	0	50
5	2	MeCN	OTs	0	55
6	1	MeCN	I	65	0
7	5	MeCN	I	75	0
8^[b]^	5	MeCN	I	0	53
9	5	DMSO	I	76	0
10^[c]^	5	DMSO	I	0	0
11	0	DMSO	I	0	0
12^[d]^	5	DMSO	I	0	0
13^[e]^	5	DMSO	I	0	0
14^[f]^	5	DMSO	I	0	0

[a] All reactions were carried out using **6** (1.1 equiv) and PhLi (1.2 equiv), followed by addition of **7** (0.20 mmol, 1.0 equiv) and photocatalyst (1–5 mol %) in solvent (0.05 m). Yields were determined after aqueous workup by ^1^H NMR analysis using an internal standard. [b] Reaction performed with the addition of 5.0 equiv H_2_O. [c] Reaction performed without phenyllithium activation. [d] Reaction performed in the dark. [e] Reaction performed using potassium cyclohexyltrifluoroborate in place of arylboronate **5**. [f] Reaction performed using DMAP (2.0 equiv) in place of phenyllithium.

We next proceeded to explore the scope of the cyclobutanation reaction with respect to the alkylboronic ester substrate (Table 2). Primary benzylic and α‐oxy boronic esters were competent coupling partners, yielding cyclobutanes **10** and **11** in moderate to good yields. The more challenging unactivated primary boronic esters could also be utilized (**12**–**16**). Furthermore, the functional group tolerance of the protocol was highlighted by the successful synthesis of cyclobutanes bearing methyl ester, nitrile, acetal, and carbazole groups. Application of unactivated cyclic secondary boronic esters led to the corresponding cyclobutanes in high yields (**8** and **17**–**20**), including oxygen‐ and nitrogen‐based heterocycles (**19** and **20**). Boronic esters bearing carbamoyl‐protected α‐amino groups provided access to cyclobutane‐substituted piperidine **21** and pyrrolidine **22**, and a bicyclo[2.2.1]heptane (norbornane) substrate gave the corresponding product **23** in excellent diastereoselectivity. Acyclic secondary boronic esters were also shown to be viable substrates (**24**–**27**). Furthermore, tertiary boronic esters, including acyclic and cyclic, also underwent cyclobutanation to give **28** and **29** in good yields.

**Table 2:** Alkyl boronic ester scope.^[a]^

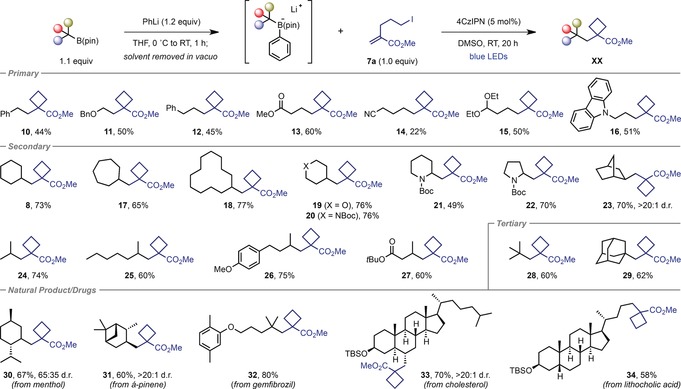

[a] Reactions were carried out on a 0.40 mmol scale with respect to the alkene **7 a**. Yields are of isolated product after chromatographic purification. Diastereomeric ratios were determined by ^1^H NMR analysis of the purified product.

To further demonstrate the utility of this deboronative chemistry for the introduction of cyclobutanes into complex molecules, we applied the optimized conditions to boronic esters derived from natural products and drugs. For example, boronic ester derivatives of the terpenes menthol and α‐pinene provided high yields of the corresponding cyclobutanes **30** and **31**, respectively, with the latter formed in excellent diastereoselectivity. A derivative of the fibrate drug gemfibrozil was prepared in excellent yield (**32**). Finally, the steroids cholesterol and lithocholic acid reacted efficiently to give cyclobutanes **33** (>20:1 d.r.) and **34** in good yields.

We then proceeded to evaluate the scope of the reaction with respect to the halide‐tethered alkene using 4‐piperidinyl boronic ester **35** as a model substrate (Table 3). In addition to methyl ester **7 a**, alkenes functionalized with benzyl esters and thioesters could be utilized, to generate cyclobutanes **37** and **38**, respectively. Other electron‐withdrawing groups that led to successful cyclobutane formation included nitrile (**39**), phenylsulfones (**40**) and pinacol boronic esters (**41**). Although the yield is modest, the formation of boronic ester **41** represents an interesting 2‐carbon boron homologation, involving insertion of both a methylene and a cyclobutane ring. In addition to cyclobutanation, cyclopropanation was also possible by using homoallylic halide substrates (*n*=1). In this case, the high rate of cyclopropane formation enabled the use of the homoallylic chloride (X=Cl) in place of the corresponding iodide. Cyclopropanes functionalized with carboxylate ester (**42**), nitrile (**43**), boronic ester (**44**), and aryl groups (**45**) could be prepared in moderate to excellent yields. Substitution on the tether in the haloalkyl alkene was also tolerated, with *gem*‐dimethyl cyclobutane **46** formed in high yield. Furthermore, extending the tether enabled the synthesis of cyclopentane (**47**), cyclohexane (**48**) and cycloheptane (**49**) products.

**Table 3:** Halide‐tethered alkene scope.^[a]^

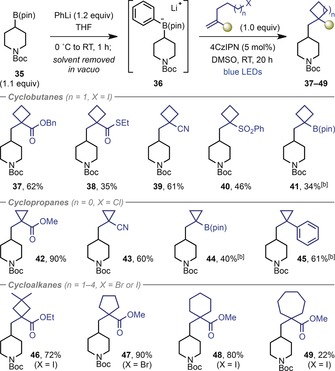

[a] Reactions were carried out on a 0.40 mmol scale with respect to the alkene substrate. Yields are of isolated product after chromatographic purification. [b] Reactions performed using DMF as the solvent.

To probe the mechanism of the reaction, we conducted several experiments to determine the intermediacy of radical and anionic intermediates. The formation of alkyl radicals by single‐electron oxidation and deboronation was confirmed by the isolation of hydroxylamine **50** upon reaction of TEMPO with boronate complex **36** (Scheme 1 a). Additionally, hexenyl boronate complex **51** underwent radical cyclization prior to reaction with **7 a** to give cyclopentane **52** instead of linear product **53** (Scheme 1 b). This suggests that an alternative two‐electron pathway in which the arylboronate complexes undergo direct S_*E*_2 reaction with the Michael acceptor is unlikely.12a

**Scheme 1:**
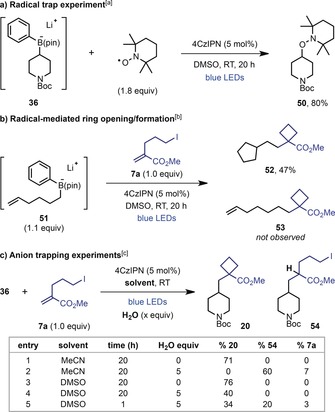
Mechanistic studies. [a] Boronate **36** was prepared in situ from **35** (1.0 equiv) and PhLi (1.1 equiv). [b] Intermediate **51** was prepared in situ from hex‐1‐en‐6‐yl boronic acid pinacol ester (1.1 equiv) and PhLi (1.2 equiv). [c] Using 1.1 equiv of **36** prepared in situ from **35** (1.1 equiv) and PhLi (1.2 equiv).

Support for the proposed radical‐polar crossover, with subsequent S_*N*_2 4‐*exo*‐*tet* cyclization of the resulting carbanion, was provided upon performing the reaction between arylboronate **36** and iodide **7 a** in the presence of H_2_O as a proton source (see Table in Scheme 1 c). With MeCN as the solvent, addition of 5.0 equiv of H_2_O resulted in a 60 % yield of Giese product **54** and none of cyclobutane **20** (entries 1 and 2). This confirms that **20** is generated through a polar (S_*N*_2), rather than a radical (S_*H*_2), cyclization, where the presence of H_2_O results in protonation of the intermediate carbanion outcompeting 4‐*exo*‐*tet* cyclization. Intriguingly, when the same reaction was performed in DMSO, only cyclobutane **20** was isolated and Giese product **54** was not observed (entries 3 and 4). This remarkable switch in selectivity seemed to suggest a change in cyclization mechanism from S_*N*_2 in MeCN to S_*H*_2 in DMSO. However, the poor mass recovery (40 % vs. 76 %) in the reaction with added H_2_O prompted further investigations. Reducing the reaction time to 1 h resulted in a mixture of **20** and **54** in 34 % and 20 % yield, respectively (entry 5), proving that **54** is unstable under the reaction conditions. These results indicate that an S_*N*_2 cyclization occurs in both MeCN and DMSO. However, the formation of cyclobutane **20** in wet DMSO appears to be a result of an S_*H*_2 cyclization, which was further supported by the observation that the yield of **20** did not decrease upon increasing the concentration of H_2_O.^13^

Based on these observations, we propose the following mechanism (Scheme 2). Highly exergonic single‐electron transfer (SET) between the excited state photocatalyst (4CzIPN*, *E*_1/2_ [PC*/PC^.−^]=1.35 V vs. SCE in MeCN)16b and arylboronate complex **5** (*E*_p/2_=0.31 V vs. SCE in MeCN) generates alkyl radical **55** and phenylboronic acid pinacol ester (**56**). Addition of radical **55** to alkene **7 a** leads to the stabilized radical **57**. SET with the reduced state of the photocatalyst (**PC^.−^**, *E*_1/2_ [PC/PC^.−^]=−1.21 V vs. SCE in MeCN for 4CzIPN) then gives anion **58** prior to polar 4‐*exo*‐*tet* cyclization to yield the cyclobutane product **8** (path A). In the presence of a proton source, **58** is intercepted to give Giese product **9 a**. In reactions performed in DMSO, an alternative pathway involving S_*H*_2 cyclization of radical **57** to form cyclobutane **8** is also operative (path B), with SET between **PC^.−^** and an iodine radical completing the catalytic cycle.

**Scheme 2:**
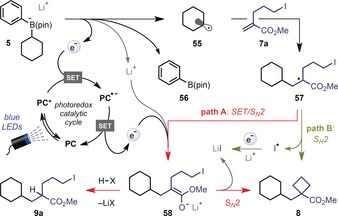
Proposed mechanism.

In conclusion, we have described the first application of alkylboronate complexes generated from pinacol boronic esters and phenyllithium in photoredox‐catalyzed deboronative transformations. The low reduction potential of these complexes allows facile single‐electron oxidation to generate non‐stabilized alkyl radicals, including primary radicals, under mild conditions. Their synthetic utility has been demonstrated in radical addition–polar cyclization cascades with halide‐tethered alkenes, providing access to structurally diverse cyclobutanes. A broad substrate scope was demonstrated and the method was readily extended to the formation of other ring systems. Given the wide availability of alkylboronic esters, this new radical deboronation strategy could find wide application in other photoredox‐catalyzed transformations.

## Conflict of interest

The authors declare no conflict of interest.

## Supporting information

As a service to our authors and readers, this journal provides supporting information supplied by the authors. Such materials are peer reviewed and may be re‐organized for online delivery, but are not copy‐edited or typeset. Technical support issues arising from supporting information (other than missing files) should be addressed to the authors.

Supplementary
