# Supplementary material for: Photoredox‐Catalyzed Cyclobutane Synthesis by a Deboronative Radical Addition–Polar Cyclization Cascade
Source: Angew Chem Int Ed Engl. 2019 Feb 15;58(12):3870–4. doi: 10.1002/anie.201813917 (PMC6492184; doi:10.1002/anie.201813917)

Angewandte Chemie
Eine Zeitschrift der Gesellschaft Deutscher Chemiker

Supporting Information

Photoredox-Catalyzed Cyclobutane Synthesis by a Deboronative Radical Addition–Polar Cyclization Cascade

*Chao Shu, Adam Noble, and Varinder K. Aggarwal**

anie_201813917_sm_miscellaneous_information.pdf

Contents

1. General Information 2
  1.1. Solvents and Reagents 2
  1.2. Chromatography and Instrumentation 2
  1.3. Photochemical Equipment and Setup 3

2. General Procedures 4

3. Optimization Studies 6
  3.1. Photocatalyst Evaluation 6
  3.2. Evaluation of Other Photoredox-catalyzed Deboronative Giese Protocols 7

4. Synthesis of Starting Materials 9
  4.1. Boronic Esters 9
  4.2. Halide-Tethered Alkenes 15

5. Product Characterization 24
  5.1. Cyclobutane Products (Table 2) 24
  5.2. Cyclopropane Products (Table 3) 45
  5.3. Other Cycloalkane Products (Table 3) 48

6. Unsuccessful Substrates 51

7. Mechanistic Studies 54
  7.1. Cyclic Volammetry 54
  7.2. Radical Trapping Experiments 56
  7.3. Anion Trapping Experiments 58
  7.4. Characterization of Giese Products 60

8. References 64

9. NMR Spectra 65

1. General Information

1.1. Solvents and Reagents

All reagents were used as received unless otherwise stated. Water is de-ionised and brine refers to a saturated aqueous solution of NaCl. DMF and DMSO were anhydrous, purchased from Sigma-Aldrich and used as received. Dichloromethane (DCM) was anhydrous (purification using a column composed of activated alumina). 4CzIPN was prepared following the method of Zhang and co-workers.[1]

1.2. Chromatography and Instrumentation

Flash column chromatography was carried out using silica gel (Aldrich, silica gel 60, 40-63 μm). Analytical thin-layer chromatography (TLC) was performed using aluminium-backed silica plates (0.25 mm, Merck, silica gel 60 F254). Compounds were visualised under UV light or by staining with aqueous basic potassium permanganate, an ethanolic solution of phosphomolybdic acid (PMA), or an ethanolic solution of ninhydrin.

^{1}H, ^{13}C and ^{11}B NMR spectra were acquired at various field strengths, as indicated, using Bruker 400 MHz, Varian VNMR 400 MHz, Varian VNMR 500 MHz, and Bruker Cryo 500 MHz spectrometers. All NMR spectra were recorder at 25 °C unless otherwise stated. Chemical shifts (δ) are given in parts per million (ppm) and referenced to $CDCl_3$ (^{1}H: 7.26 ppm) or DMSO-d_6 (^{1}H: 2.50 ppm). Coupling constants (J) are given in Hertz (Hz) and refer to apparent multiplicities (s = singlet, br. s = broad singlet, d = doublet, t = triplet, q = quartet, quin = quintet, sex = sextet, h = heptet, m = multiplet, dd = doublet of doublets, etc.). The ^{1}H NMR spectra are reported as follows: chemical shift (multiplicity, coupling constants, number of protons).

Gas chromatography (GC) was performed on an Agilent Technologies 6890N Network GC System using an Agilent HP-5 column (15 m × 0.25 mm × 0.25 μm).

High-resolution mass spectra (HRMS) were recorded on a Bruker micrOTOF instrument using electrospray ionisation (ESI). Low-resolution mass spectra (LRMS) were recorded on an Agilent 7820A GC-MS equipped with a HP-5MS UI column (30 m x 0.25 mm x 0.25 μm) using electron ionisation (EI).

Infra-red (IR) spectra were recorded on a Perkin Elmer Spectrum One FT-IR spectrometer as a thin film. Selected absorption maxima (ν_{max}) are reported in wavenumbers (cm^{-1}).

Melting points were recorded in degrees Celsius (°C), using a Kofler hot-stage microscope apparatus and are reported uncorrected.

Optical rotations ($[\alpha]_D^T$) were measured on a Bellingham and Stanley Ltd. ADP220 polarimeter and are quoted in (° mL)(g dm)$^{-1}$.

1.3. Photochemical Equipment and Setup

The blue LED lamps were either 40 W Kessil A160WE Tuna Blue LED Aquarium Lights (used with the colour dial turned fully anticlockwise and the intensity dial turned fully clockwise) or 40 W Kessil PR160-427 nm LED Photoredox Lights (used with the intensity dial set to 100).

All photoredox reactions were carried out at room temperature (r.t.). Fan assisted cooling was used to maintain this temperature.

Reaction set-up:

The reaction flasks were positioned 5 cm from a single 40 W Kessil LED lamp (Figure S1).

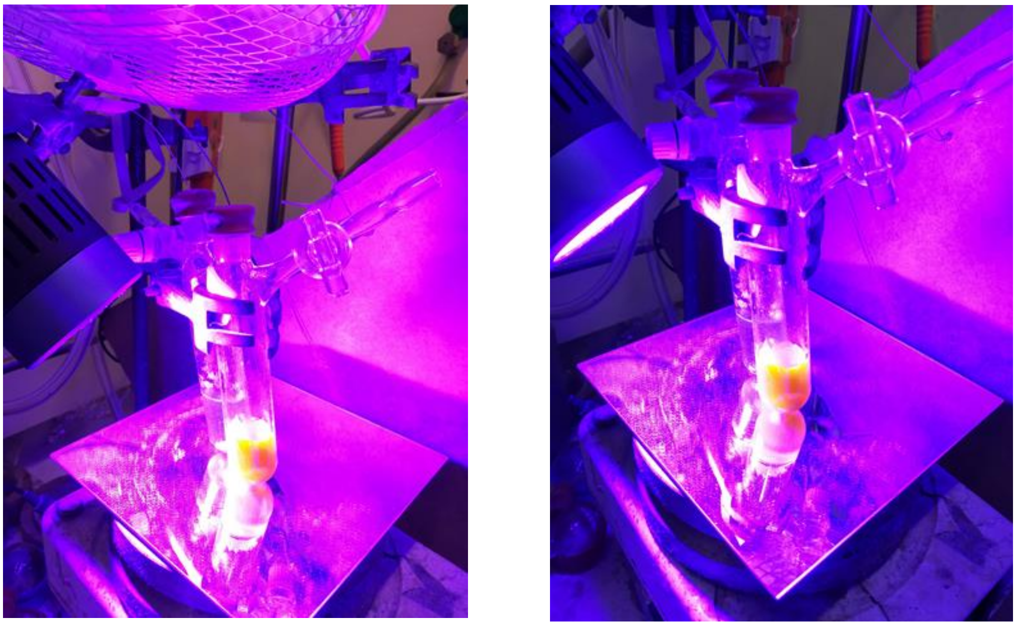

Figure S1. Photoredox reaction setup.

2. General Procedures

General Procedure A (for reactions of alkyl boronic esters with iodide **7a**, see Table 2):

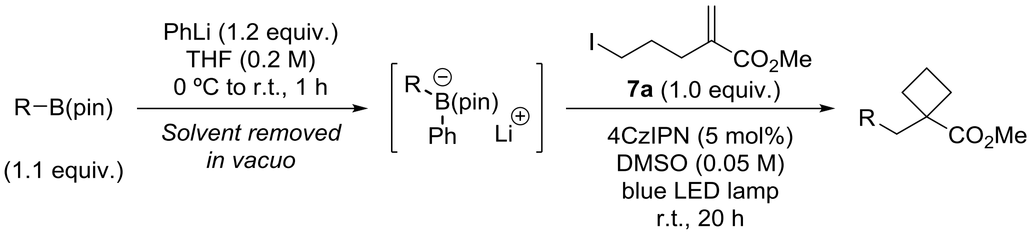

To a stirred solution of boronic ester (0.44 mmol, 1.1 equiv.) in THF (1.75 mL) under N_2 at 0 °C was added phenyllithium (1.9 M in dibutyl ether, 0.48 mmol, 1.2 equiv.) dropwise. The solution was then stirred for 1 h at 0 °C, warmed to r.t. and stirred for a further 10 min before removing the solvent under vacuum. Degassed dry DMSO (5 mL) was added to the system. The mixture was irradiated with a 40 W Kessil LED lamp with fan cooling. A degassed solution of iodide-tethered alkene **7a** (0.40 mmol, 1.0 equiv.) and 4CzIPN (5.0 mol%) in DMSO (3.0 mL) was added under irradiation. The N_2 inlet was removed and the flask sealed with parafilm. The reaction mixture was stirred vigorously overnight (20 h) under constant irradiation. The reaction mixture was diluted with DCM (60 mL) and the solution washed with saturated aqueous NH_4Cl (30 mL), water (30 mL) and brine (30 mL). The resulting organic phase was dried ($MgSO_4$), filtered and concentrated *in vacuo*. The crude product was then purified by flash column chromatography.

General Procedure B (for reactions of boronic ester **35** with halide-tethered alkenes, see Table 3):

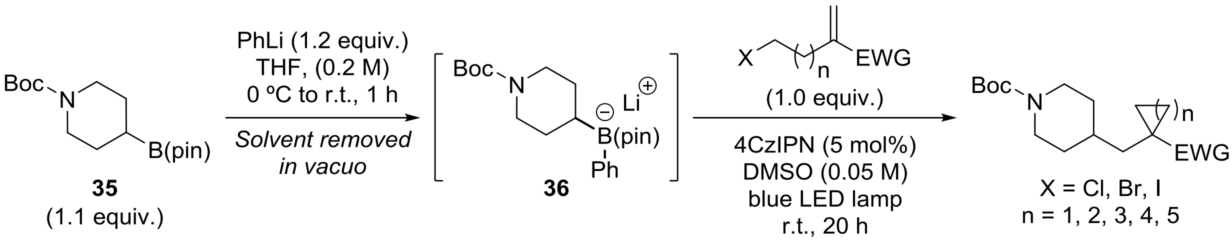

To a stirred solution of *N*-Boc-piperidine-4-boronic acid pinacol ester (**35**) (0.440 mmol, 137 mg, 1.1 equiv.) in THF (1.75 mL) under N_2 at 0 °C was added phenyllithium (1.9 M in dibutyl ether, 0.48 mmol, 1.2 equiv.) dropwise. The solution was then stirred for 1 h at 0 °C, warmed to r.t. and stirred for a further 10 min before removing the solvent under vacuum. Degassed dry solvent (DMSO or DMF, 5 mL) was added to the system. The mixture was irradiated with a 40 W Kessil LED lamp with fan cooling. A degassed solution of the halide-tethered alkene (0.40 mmol, 1.0 equiv.) and 4CzIPN (2.0–5.0 mol%) in DMSO or DMF (3.0 mL) was added under irradiation. The N_2 inlet was removed and the flask sealed with parafilm. The reaction mixture was stirred vigorously overnight (20 h) under constant irradiation.

The reaction mixture was diluted with DCM (60 mL) and the solution washed with saturated aqueous NH_4Cl (30 mL), water (30 mL) and brine (30 mL). The resulting organic phase was dried ($MgSO_4$), filtered and concentrated *in vacuo*. The crude product was then purified by flash column chromatography.

3. Optimization Studies

3.1. Photocatalyst Evaluation

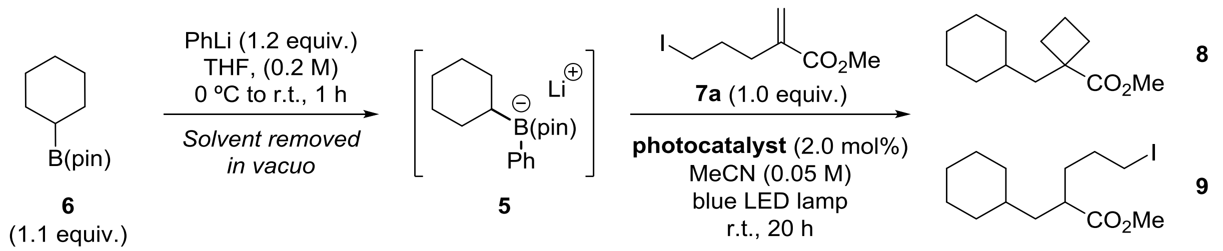

Entry	**Photocatalyst**	Cyclobutane **8** (%)	Giese-type product **9** (%)
1	4CzIPN	70	0
2	$Ir[dF(CF_3)ppy]_2(dtbbpy)PF_6$	45	22
3	$Ir[dF(CF_3)ppy]_2(bpy)PF_6$	46	13
4	$Ir(ppy)_3$	4	10
5	$Ru(bpy)_3(PF_6)_2$	48	23
6	Eosin Y	30	25

All reactions were carried out using **6** (1.1 equiv) and PhLi (1.2 equiv), followed by addition of **7** (0.20 mmol, 1.0 equiv) and photocatalyst (2 mol%) in solvent (0.05 M). Yields were determined after aqueous workup by ^{1}H NMR analysis using an internal standard.

3.2. Evaluation of Other Photoredox-catalyzed Deboronative Giese Protocols

<u>Reaction without phenyllithium activation of **6**:</u>

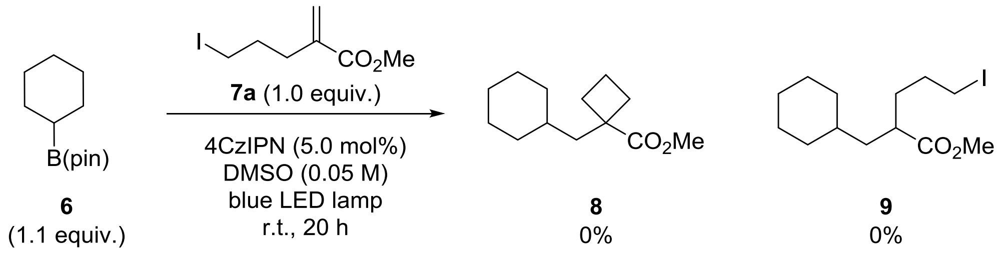

To a 7 mL vial equipped with a magnetic stir bar was added 2-cyclohexyl-4,4,5,5-tetramethyl-1,3,2-dioxaborolane (**6**) (23 mg, 0.11 mmol, 1.1 equiv.), 4CzIPN (3.9 mg, 5.0 mol%) and anhydrous DMSO (0.05 M), followed by the iodide-tethered alkene **7a** (25 mg, 0.10 mmol, 1.0 equiv.). The vial was sealed with a septum and the reaction mixture degassed by sparging with nitrogen for 10 min. The nitrogen inlet was removed, and the vial further sealed with parafilm. The reaction mixture was stirred at 800 rpm and irradiated with a 40 W Kessil LED lamp with fan cooling for 20 h. The reaction mixture was diluted with DCM (20 mL) and the solution washed with saturated aqueous NH_4Cl (20 mL), water (2 × 20 mL) and brine (30 mL). The resulting organic phase was dried ($MgSO_4$), filtered and concentrated *in vacuo*. Yields were determined by ^1H NMR using diethyl phthalate as internal standard.

<u>Reaction using the corresponding trifluoroborate salt:</u>

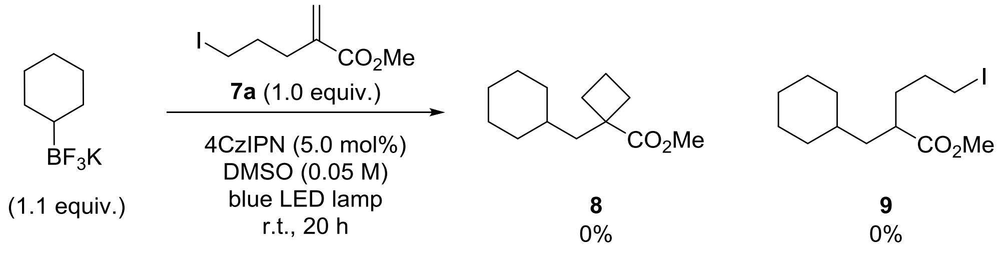

To a 7 mL vial equipped with a magnetic stir bar was added the potassium cyclohexyltrifluoroborate (21 mg, 0.11 mmol, 1.1 equiv.), 4CzIPN (3.9 mg, 5.0 mol%) and anhydrous DMSO (0.05 M), followed by the iodide-tethered alkene **7a** (25 mg, 0.1 mmol, 1.0 equiv.). The vial was sealed with a septum and the reaction mixture degassed by sparging with nitrogen for 10 min. The nitrogen inlet was removed, and the vial further sealed with parafilm. The reaction mixture was stirred at 800 rpm and irradiated with a 40 W Kessil LED lamp with fan cooling for 20 h. The reaction mixture was diluted with DCM (20 mL) and the solution washed with saturated aqueous NH_4Cl (20 mL), water (2 × 20 mL) and brine (30 mL). The resulting organic phase was dried ($MgSO_4$), filtered and concentrated *in vacuo*. Yields were determined by ^1H NMR using diethyl phthalate as internal standard.

Reaction using DMAP to activate **6**:

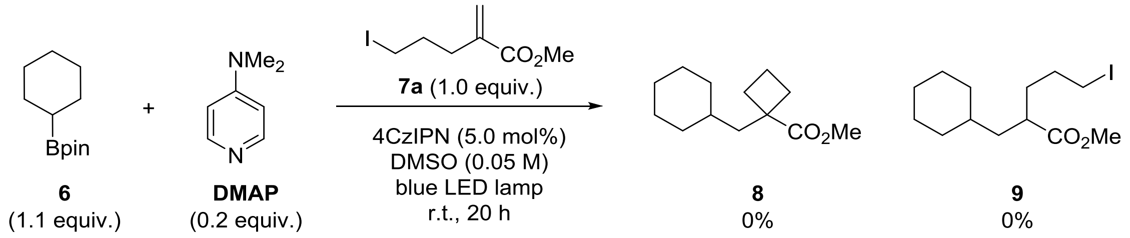

To a 7 mL vial equipped with a magnetic stir bar was added 2-cyclohexyl-4,4,5,5-tetramethyl-1,3,2-dioxaborolane (**6**) (23 mg, 0.11 mmol, 1.1 equiv.), 4CzIPN (3.9 mg, 5.0 mol%), DMAP (2.4 mg, 0.02 mmol, 0.2 equiv.) and anhydrous DMSO (0.05 M), followed by the iodide-tethered alkene **7a** (25 mg, 0.10 mmol, 1.0 equiv.). The vial was sealed with a septum and the reaction mixture degassed by sparging with nitrogen for 10 min. The nitrogen inlet was removed, and the vial further sealed with parafilm. The reaction mixture was stirred at 800 rpm and irradiated with a 40 W Kessil LED lamp with fan cooling for 20 h. The reaction mixture was diluted with DCM (20 mL) and the solution washed with saturated aqueous NH_4Cl (20 mL), water (2 × 20 mL) and brine (30 mL). The resulting organic phase was dried ($MgSO_4$), filtered and concentrated *in vacuo*. Yields were determined by ^1H NMR using diethyl phthalate as internal standard. Complete decomposition of **7a** was observed under these reaction conditions.

Reactions using previously reported deboronative Giese conditions:

Submitting iodide-tethered alkene **7a** to Akita and co-workers' optimized conditions for Giese reactions of trifluoroborate salts (*Adv. Synth. Catal.* **2012**, *354*, 3414) gave no desired product. This was also the case when using Ley and co-workers' optimized conditions for Giese reactions of Lewis base-activated pinacol boronic esters (*Angew. Chem. Int. Ed.* **2017**, *56*, 15136).

Conditions of Akita and co-workers:

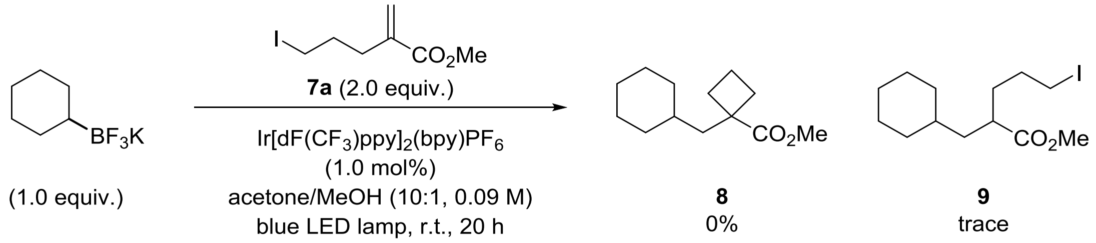

Conditions of Ley and co-workers:

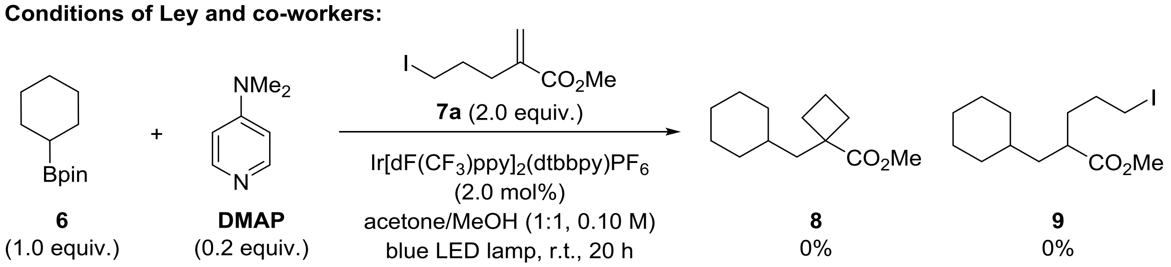

4. Synthesis of Starting Materials

4.1. Boronic Esters

The following boronic esters were purchased from commercial suppliers:

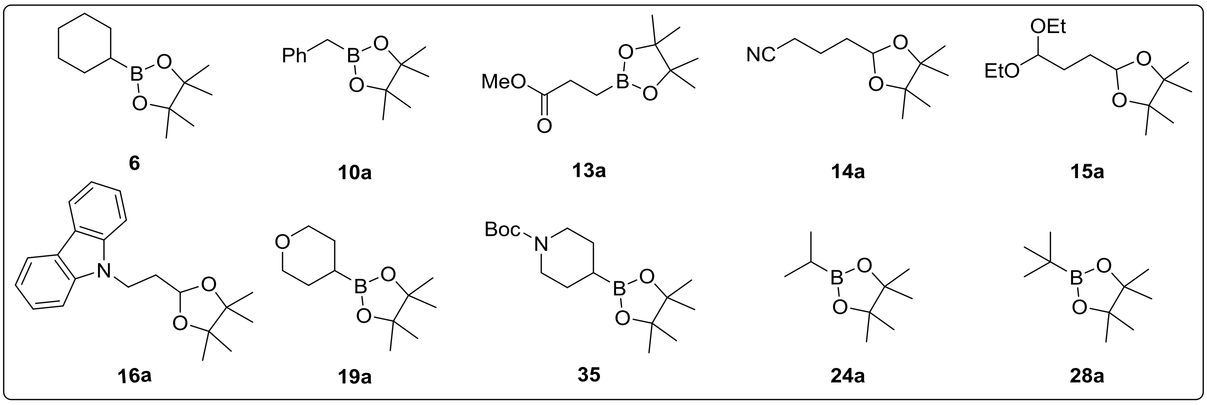

The syntheses of the following boronic esters have been previously reported by our group:[2]

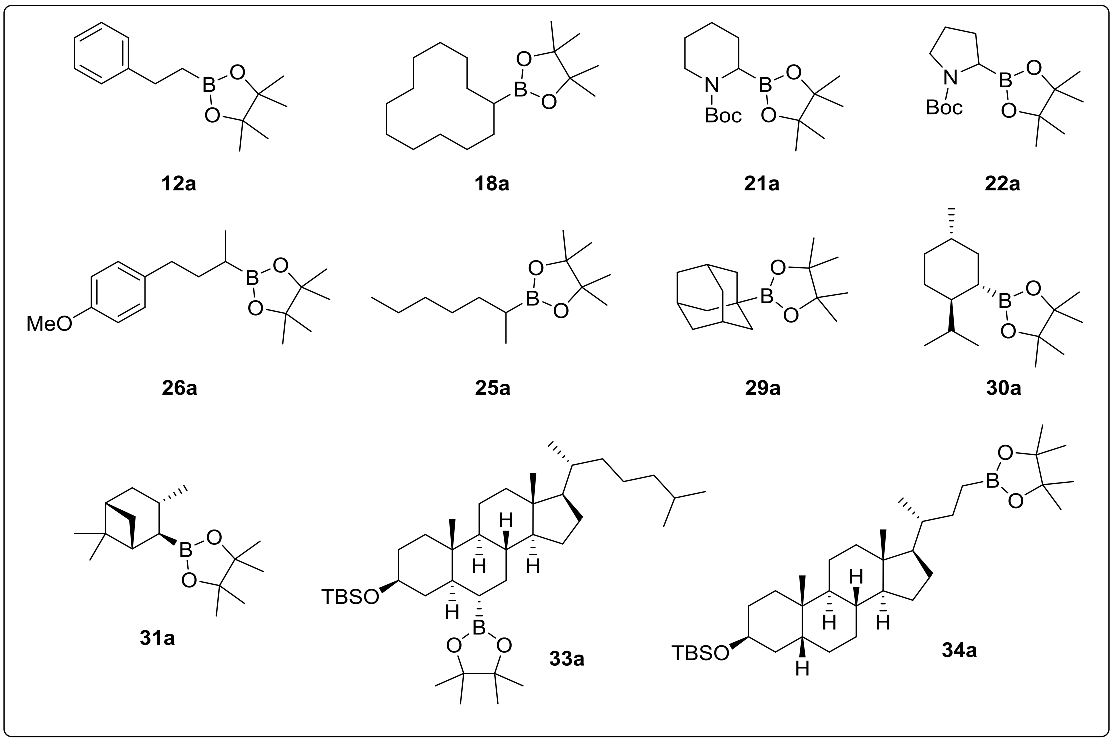

2-((Benzyloxy)methyl)-4,4,5,5-tetramethyl-1,3,2-dioxaborolane (11a)

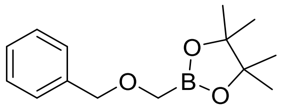

2-((Benzyloxy)methyl)-4,4,5,5-tetramethyl-1,3,2-dioxaborolane (**11a**) was prepared following a modified literature procedure:[3] To a solution of benzyl alcohol (5.00 mmol, 540 mg, 1.0 equiv.) in anhydrous DMSO (20 mL) was added portionwise NaH (60% in mineral oil, 300 mg, 7.5 mmol, 1.5 equiv.) at 0 °C under nitrogen. The reaction was stirred for 1 h at r.t. before being cooled to 0 ºC and 2-(bromomethyl)-4,4,5,5-tetramethyl-1,3,2-dioxaborolane (1.6 g, 7.5 mmol, 1.5 equiv.) added. The resulting mixture was allowed to warm to r.t. and stirred overnight. The reaction was slowly quenched with water (30 mL) and extracted with DCM (3 × 40 mL). The combined organic layers were washed with brine (30 mL), dried ($MgSO_4$), filtered and concentrated *in vacuo*. The resulting residue was purified by flash column chromatography (10% EtOAc/hexane) to give the pure compound (**11a**) (868 mg, 3.5 mmol, 70%) as a pale-yellow oil.

TLC: R_f = 0.41 (10% EtOAc/hexane, $KMnO_4$ stain).

^{1}H NMR (400 MHz, $CDCl_3$): δ_H 7.37 – 7.24 (m, 5H), 4.51 (s, 2H), 3.28 (s, 2H), 1.27 (s, 12H) ppm.

^{13}C NMR (101 MHz, $CDCl_3$): δ_C 138.1, 128.2, 128.2, 127.5, 83.9, 75.8, 24.8 ppm. *The carbon attached to boron could not be observed due to quadrupolar relaxation.*

IR (film) ν_{max}: 3410, 2979, 2870, 1713, 1474, 1451, 1370, 1143, 850, 713 cm^{-1}.

HRMS (ESI$^+$) calcd. for $C_{14}H_{21}BNaO_3$ $[M+Na]^+$ 271.1467, found 271.1466.

***tert*-Butyl 3-(4,4,5,5-tetramethyl-1,3,2-dioxaborolan-2-yl)butanoate** (**27a**)

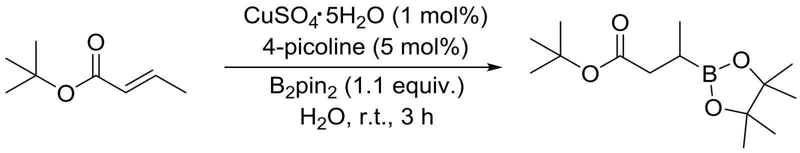

tert-Butyl 3-(4,4,5,5-tetramethyl-1,3,2-dioxaborolan-2-yl)butanoate (**27a**) was synthesised in one step from *tert*-butyl (*E*)-but-2-enoate following a literature procedure. All recorded spectroscopic data matched those previously reported in the literature.[4]

^{1}H NMR (400 MHz, $CDCl_3$): δ_H 2.27 (dd, J = 17.0, 7.5 Hz, 1H), 2.35 (dd, J = 17.0, 7.5 Hz, 1H), 1.43 (s, 9H), 1.39 – 1.29 (m, 1H), 1.231 (s, 6H), 1.225 (s, 6H), 0.98 (d, J = 7.5 Hz, 3H) ppm.

^{13}C NMR (101 MHz, $CDCl_3$): δ_C 173.3, 83.0, 79.8, 38.8, 28.1, 24.7, 24.7, 14.9 ppm. *The carbon attached to boron could not be observed due to quadrupolar relaxation*.

General Procedure for Borylation of Alkyl Bromides (for preparation of **17a** and **23a**):

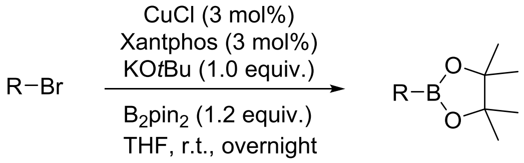

Using a modified literature procedure.[5] Under a N_2 atmosphere, copper(I) chloride (15 mg, 0.15 mmol), bis(pinacolato)diboron (1.52 g, 6.00 mmol, 1.2 equiv.), and Xantphos (87 mg, 0.15 mmol) were placed in an oven-dried Schlenk flask. The flask was evacuated and backfilled with nitrogen three times. THF (10 mL) and KO*t*Bu (1.0 M in THF, 5.0 mL, 5.0 mmol, 1.0 equiv.) were added. Then alkyl halide (5.0 mmol, 1.0 equiv.) was added dropwise. The reaction was stirred at r.t. overnight before being passed through a short silica column eluting with ethyl acetate. The crude mixture was further purified by flash column chromatography to give the pure product.

2-Cycloheptyl-4,4,5,5-tetramethyl-1,3,2-dioxaborolane (**17a**)

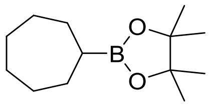

Prepared following the above **General Procedure** with bromocycloheptane. Purification by flash column chromatography (5% EtOAc/hexane) gave the title compound (885 mg, 4.0 mmol, 79%) as a colourless oil. All recorded spectroscopic data matched those previously reported in the literature.[6]

^{1}H NMR (400 MHz, $CDCl_3$): δ_H 1.79 – 1.62 (m, 4H), 1.58 – 1.52 (m, 2H), 1.52 – 1.40 (m, 6H), 1.23 (s, 12H), 1.11 – 1.01 (m, 1H) ppm.

^{13}C NMR (101 MHz, $CDCl_3$): δ_C 82.7, 29.6, 29.0, 28.3, 24.7 ppm. *The carbon attached to boron could not be observed due to quadrupolar relaxation*.

2-((1*S*,4*S*)-Bicyclo[2.2.1]heptan-2-yl)-4,4,5,5-tetramethyl-1,3,2-dioxaborolane (23a)

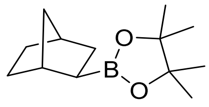

Prepared following the above **General Procedure** with (1*S*,4*R*)-2-bromobicyclo[2.2.1]heptane. Purification by flash column chromatography (5% EtOAc/hexane) gave the title compound (777 mg, 3.5 mmol, 70%) as a colourless oil. All recorded spectroscopic data matched those previously reported in the literature.[7]

^{1}H NMR (400 MHz, $CDCl_3$): δ_H 2.28 – 2.24 (m, 1H), 2.22 – 2.17 (m, 1H), 1.58 – 1.42 (m, 3H), 1.38 – 1.30 (m, 1H), 1.22 (s, 12H), 1.26 – 1.11 (m, 4H), 0.90 – 0.84 (m, 1H) ppm.

^{13}C NMR (101 MHz, $CDCl_3$): δ_C 82.8, 38.7, 38.1, 36.6, 32.2, 32.2, 29.3, 24.7 ppm. *The carbon attached to boron could not be observed due to quadrupolar relaxation.*

2-(5-(2,5-Dimethylphenoxy)-2-methylpentan-2-yl)-4,4,5,5-tetramethyl-1,3,2-dioxaborolane (32a)

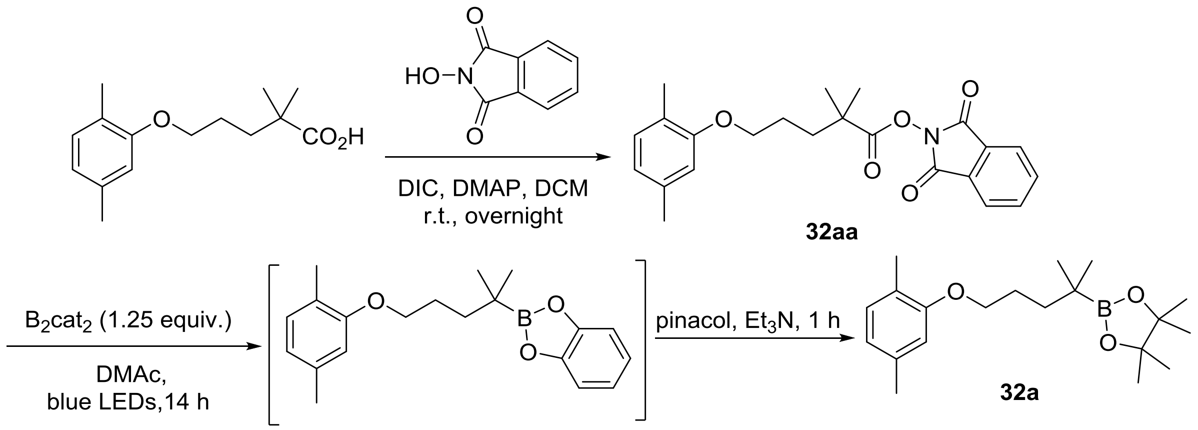

Prepared following a literature procedure.[2b] *N*-Hydroxyphthalimide (0.65 g, 4.0 mmol, 1.0 equiv.), DMAP (49 mg, 0.40 mmol, 0.1 equiv.) and gemfibrozil (1.0 g, 4.0 mmol, 1.0 equiv.) were added to dichloromethane (40 mL). DIC (0.50 g, 4.0 mmol, 1.0 equiv.) was added and the reaction mixture was allowed to stir at r.t. overnight before concentrating under reduced pressure. The crude residue was purified by flash column chromatography to afford the corresponding NHPI ester (**32aa**) (1.4 g, 3.6 mmol) in 90% yield as a pale-yellow oil.

NHPI ester **32aa** (1.5 g, 4.0 mmol, 1.0 equiv.) and B_2cat_2 (1.2 g, 5.0 mmol, 1.25 equiv.) were added to a flame-dried vial containing a small magnetic stirrer bar. DMAc (30 mL) was added and then the headspace of the vial was purged with a gentle stream of argon for approximately 10 seconds. The vial was sealed and the reaction stirred under blue LED irradiation for 14 h. Pinacol (1.9 g, 16 mmol, 4.0 equiv.) was dissolved in Et_3N (20 mL), added to the reaction mixture and stirred for 2 h. The reaction

was diluted with H_2O (30 mL) and saturated aqueous NH_4Cl (30 mL) and extracted with EtOAc (3 × 50 mL). The organic layers were combined and concentrated under reduced pressure. The crude residue was purified by flash column chromatography (5% EtOAc/hexane) to yield boronic ester **32a** (0.80 g, 2.5 mmol) in 62% yield as a colourless oil.

TLC: R_f = 0.42 (5% EtOAc/hexane, $KMnO_4$ stain).

^{1}H NMR (400 MHz, $CDCl_3$): δ_H 7.02 (d, *J* = 7.4 Hz, 1H), 6.66 (d, *J* = 7.7 Hz, 1H), 6.64 (s, 1H), 3.94 (t, *J* = 6.6 Hz, 2H), 2.33 (s, 3H), 2.21 (s, 3H), 1.84 – 1.74 (m, 2H), 1.50 – 1.42 (m, 2H), 1.25 (s, 12H), 0.99 (s, 6H) ppm.

^{13}C NMR (101 MHz, $CDCl_3$): δ_C 157.1, 136.3, 130.2, 123.6, 120.4, 112.0, 82.9, 68.6, 37.3, 26.5, 24.8, 24.7, 21.4, 15.8 ppm. *The carbon attached to boron could not be observed due to quadrupolar relaxation*.

^{11}B NMR (128 MHz, $CDCl_3$): δ_B 34.4 (br. s, 1B) ppm.

IR (film) ν_{max}: 2978, 2868, 1615, 1585, 1508, 1474, 1307, 1264, 1037, 850, 801 cm^{-1}.

HRMS (ESI$^+$) calcd. for $C_{20}H_{33}BNaO_3$ [M+Na]$^+$ 355.2415, found 355.2415.

2-(Hex-5-en-1-yl)-4,4,5,5-tetramethyl-1,3,2-dioxaborolane (50a)

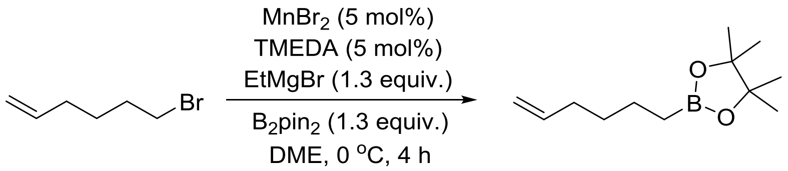

Prepared following a literature procedure.[1] A flame-dried vial was charged with $MnBr_2$ (64 mg, 5.0 mol%) and B_2pin_2 (1.98 g, 1.30 equiv.) before evacuating and back-filling with N_2. DME (12 mL, 0.5 M) was added, followed by TMEDA (48 µL, 5.0 mol%), and the vial was placed in an ice bath. Ethylmagnesium bromide (3 M in Et_2O, 2.6 mL, 1.3 equiv.) was added dropwise and the reaction was allowed to stir for 2 minutes. 6-Bromohex-1-ene (978 mg, 6.00 mmol, 1.0 equiv.) was added to the reaction mixture in one portion. The reaction mixture was stirred at 0 °C for 5 hours. The reaction was quenched with 1 M aqueous HCl (10 mL), extracted with Et_2O (3 × 40 mL), dried ($MgSO_4$), filtered and concentrated *in vacuo*. The crude residue was purified by flash column chromatography (5% EtOAc/hexane) to yield the desired boronic ester (0.85 g, 4.0 mmol) in 67% yield as a colourless oil. All recorded spectroscopic data matched those previously reported in the literature.[8]

TLC: R_f = 0.20 (5% EtOAc/hexane, $KMnO_4$ stain).

^{1}H NMR (400 MHz, $CDCl_3$): δ_H 5.87 – 5.75 (m, 1H), 5.02 – 4.88 (m, 2H), 2.07 – 2.00 (m, 2H), 1.47 – 1.34 (m, 4H), 1.24 (s, 12H), 0.77 (t, *J* = 6.9 Hz, 2H) ppm.

^{13}C NMR (101 MHz, $CDCl_3$): δ_C 139.2, 114.0, 82.9, 33.6, 31.7, 24.8, 23.6 ppm. *The carbon attached to boron could not be observed due to quadrupolar relaxation*.

4.2. Halide-Tethered Alkenes

Compounds 7a-7c, 42a-45a, 46a, and 47a have been previously reported by our group:[9]

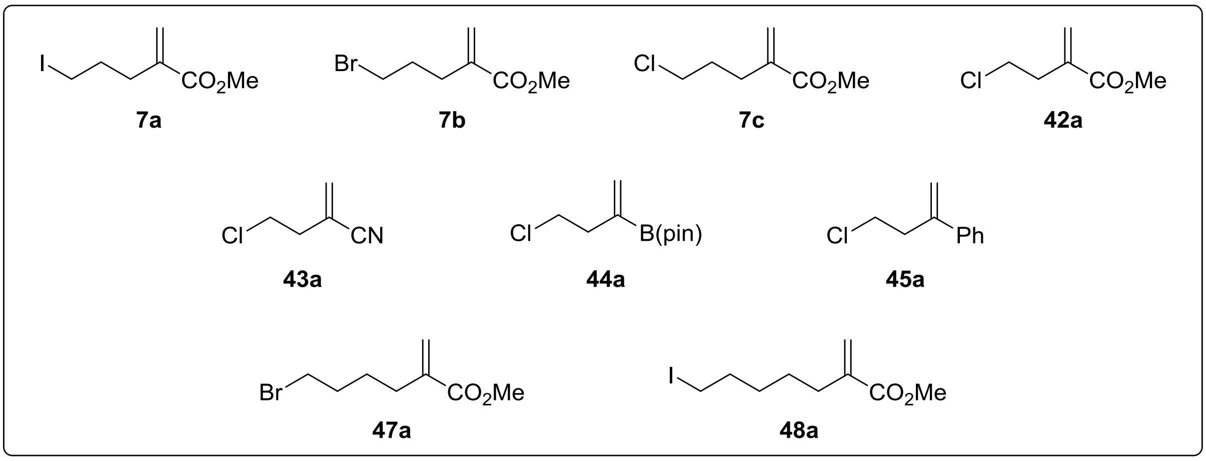

Methyl 2-methylene-5-(tosyloxy)pentanoate (7d)

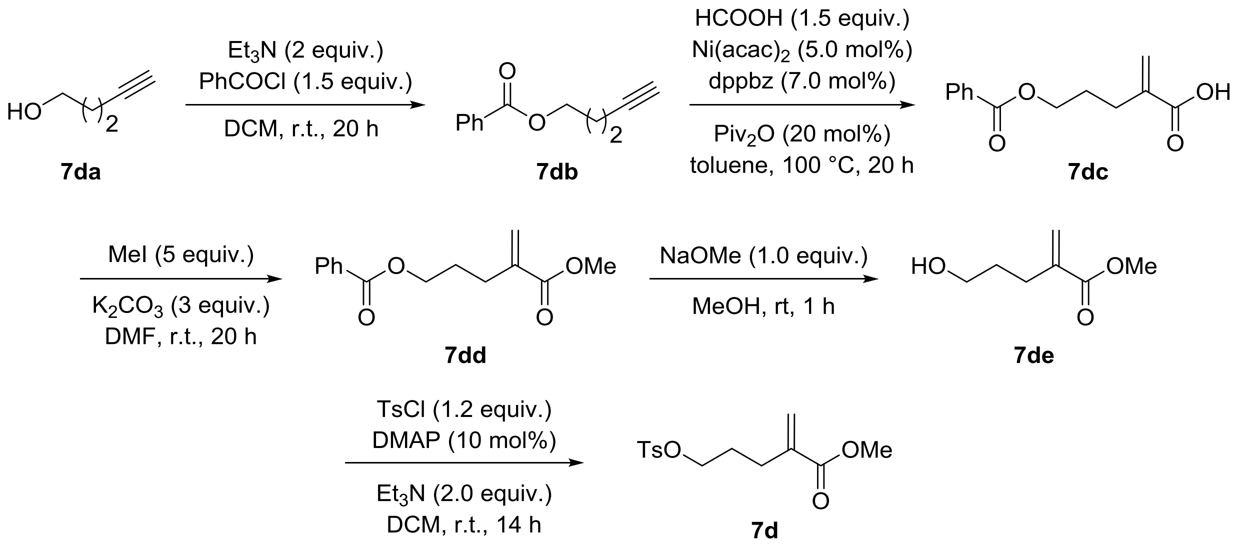

4-Pentyn-1-ol (**7da**) (1.68 g, 20 mmol, 1.0 equiv.) and Et_3N (4.2 g, 40 mmol, 2.0 equiv.) were added to DCM (40 mL). Benzoyl chloride (4.2 g, 30 mmol, 1.5 equiv.) was added at 0 °C, and the reaction mixture was allowed to stir at r.t. for 20 h. The reaction was then quenched with water (20 mL), extracted with DCM (3 × 60 mL). The combined organic layers were washed with brine (60 mL), dried ($MgSO_4$), filtered and concentrated *in vacuo*. The crude residue was purified by flash column chromatography to afford the corresponding ester **7db** (3.4 g, 18 mmol) in 90% yield as a pale-yellow oil.

Compounds **7dc** and **7dd** were prepared following a modified literature procedure:[9] A Schlenk tube containing a magnetic stir bar was charged with $Ni(acac)_2$ (128 mg, 0.500 mmol, 5.00 mol%) and 1,2-bis(diphenylphosphino)benzene (312 mg, 0.700 mmol, 7.00 mol%). The tube was evacuated and back-

filled with N_2 three times. Toluene (30 mL) was added, followed by **7db** (1.9 g, 10 mmol, 1.0 equiv.), formic acid (690 mg, 15.0 mmol, 1.50 equiv.) and pivalic anhydride (374 mg, 2.00 mmol, 20.0 mol%). The reaction mixture was heated to 100 °C for 24 h before cooling to r.t. and concentrating *in vacuo*. The crude residue was purified by flash column chromatography (50% EtOAc/hexane) to give carboxylic acid **7dc** (2.2 g, 9.5 mmol, 95%) as a colourless oil.

K_2CO_3 (3.7 g, 27 mmol, 3.0 equiv.) was added to a solution of **7dc** (2.1 g, 9.0 mmol, 1.0 equiv.) in DMF (18 mL) at r.t. To the solution was added MeI (6.4 g, 45 mmol, 5.0 equiv.) at 0 °C. The mixture was vigorously stirred at r.t. overnight before adding water (25 mL) and DCM (50 mL). The organic layer was separated, and the aqueous layer was extracted with DCM (3 × 50 mL). The combined organic layers were washed with H_2O (3 × 50 mL), saturated aqueous $NaHCO_3$, brine, dried ($MgSO_4$), filtered and concentrated *in vacuo*. The crude residue was purified by flash column chromatography (20% EtOAc/hexane), providing methyl ester **7dd** (1.71 g, 6.93 mmol, 77%) as a yellow oil.

To a solution of **7dd** (1.5 g, 6.0 mmol, 1.0 equiv.) in MeOH (12 mL) was added 1 M aqueous NaOMe (6.0 mL, 6.0 mmol, 1.0 equiv.). The corresponding mixture was stirred at r.t. for 1 h before adding water (20 mL) and extracting with DCM (3 × 40 mL). The combined organic layers were washed with brine (30 mL), dried ($MgSO_4$), filtered and concentrated *in vacuo*. The crude residue was purified by flash column chromatography (30% EtOAc/hexane) to give alcohol **7de** (0.61 g, 4.2 mmol, 70%) as a pale-yellow oil.

To a solution of alcohol **7de** (0.29 g, 2.0 mmol, 1.0 equiv.) in DCM (20 mL) at r.t. was added triethylamine (0.53 g, 4.0 mol, 2.0 equiv.), DMAP (24 mg, 0.20 mol, 10 mol%) and *p*-toluenesulfonyl chloride (0.47 g, 2.4 mmol, 1.2 equiv.). After 14 h, aqueous 1 M HCl (10 mL) was added and the phases were separated. The aqueous phase was extracted into DCM (3 × 20 mL) and the combined organic phases dried ($MgSO_4$), filtered, and concentrated *in vacuo*. Purification by flash column chromatography (10% EtOAc/hexane) gave sulfonate **7d** (187.5 mg, 1.3 mmol, 65%) as a colourless oil.

TLC: R_f = 0.30 (10% EtOAc/hexane, $KMnO_4$ stain).

^{1}H NMR (400 MHz, $CDCl_3$): δ_H 7.78 (d, *J* = 8.2 Hz, 2H), 7.34 (d, *J* = 8.1 Hz, 2H), 6.14 (s, 1H), 5.49 (s, 1H), 4.03 (t, *J* = 6.2 Hz, 2H), 3.72 (s, 3H), 2.45 (s, 3H), 2.34 (t, *J* = 7.5 Hz, 2H), 1.88 – 1.79 (m, 2H) ppm.

^{13}C NMR (101 MHz, $CDCl_3$): δ_C 167.1, 144.7, 138.7, 133.1, 129.8, 127.9, 126.1, 69.5, 51.8, 28.0, 27.6, 21.6 ppm.

IR (film) ν_{max}: 2988, 2969, 1717(s), 1631, 1597, 1439, 1358, 1174, 1096, 907, 729 cm^{-1}.

HRMS (ESI$^+$): calcd. for $C_{14}H_{18}NaO_5S$ $[M+Na]^+$ 321.0767, found 321.0772.

Benzyl 5-iodo-2-methylenepentanoate (37a)

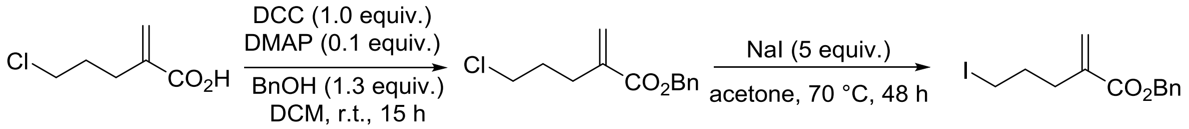

37aa **37a**

Prepared following a modified literature procedure.[10] A 50 mL round-bottomed flask was charged with carboxylic acid[9] (5.0 mmol, 0.74 g, 1.0 equiv.), benzyl alcohol (0.70 g, 6.5 mmol, 1.3 equiv.), 4-(dimethylamino)pyridine (61 mg, 0.50 mmol, 0.10 equiv.) and DCM (30 mL) and cooled to 0 °C. Dicyclohexylcarbodiimide (1.0 g, 5.0 mmol, 1.0 equiv.) was added in three portions. The reaction mixture was allowed to warm to r.t. and stirred for 15 h. The resulting suspension was filtered through Celite, and the filtrate was washed with saturated aqueous $NaHCO_3$ (30 mL), water (30 mL), brine (30 mL), dried ($MgSO_4$), filtered and concentrated *in vacuo*. Purification by flash column chromatography (10% EtOAc/hextane) gave acrylate **37aa** (952 mg, 4.0 mmol, 80%) as a colourless oil.

A solution of acrylate **37aa** (952 mg, 4.0 mmol, 1.0 equiv.) and NaI (3.0 g, 20 mmol, 5.0 equiv.) in acetone (8.0 mL) was heated to reflux for 48 h. The reaction was cooled to r.t., quenched with water (30 mL), extracted with DCM (3 × 40 mL). The combined organic layers were washed with brine (30 mL), dried ($MgSO_4$), filtered and concentrated *in vacuo*. The resulting residue was purified by flash column chromatography (10% EtOAc/hexane) to give the pure compound (**37a**) (0.91 g, 2.7 mmol, 69%) as a pale yellow oil.

TLC: R_f = 0.45 (10% EtOAc/hexane, $KMnO_4$ stain).

^{1}H NMR (400 MHz, $CDCl_3$): δ_H 7.40 – 7.30 (m, 5H), 6.26 (s, 1H), 5.65 (s, 1H), 5.20 (s, 2H), 3.18 (t, *J* = 6.8 Hz, 2H), 2.46 (t, *J* = 7.2 Hz, 2H), 2.06 – 1.96 (m, 2H) ppm.

^{13}C NMR (101 MHz, $CDCl_3$): δ_C 166.6, 138.7, 135.9, 128.6, 128.2, 128.1, 126.4, 66.5, 32.7, 31.9, 5.9 ppm.

IR (film) ν_{max}: 2979, 2870, 1713(s), 1629, 1497, 1454, 1264, 1217, 1110, 750, 734 cm^{-1}.

HRMS (ESI^+) calcd. for $C_{13}H_{15}INaO_2$ $[M+Na]^+$ 353.0009, found 352.9996.

S-Ethyl 5-iodo-2-methylenepentanethioate (38a)

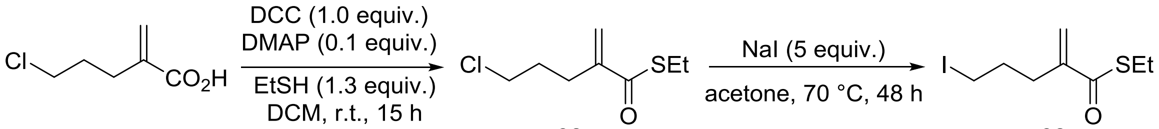

Prepared following a modified literature procedure.[10] A 50 mL round-bottomed flask was charged with carboxylic acid (5.0 mmol, 0.74 g, 1.0 equiv.), ethanethiol (0.40 g, 6.5 mmol, 1.3 equiv.), 4-(dimethylamino)pyridine (61 mg, 0.50 mmol, 0.10 equiv.) and DCM (20 mL) and cooled to 0 °C. Dicyclohexylcarbodiimide (1.0 g, 5.0 mmol, 1.0 equiv.) was added in three portions. The reaction mixture was allowed to warm to r.t. and stirred for 15 h. The resulting suspension was filtered through Celite, and the filtrate was washed with saturated aqueous $NaHCO_3$ (30 mL), water (30 mL), brine (30 mL), dried ($MgSO_4$), filtered and concentrated *in vacuo*. Purification by flash column chromatography (10% EtOAc/hextane) gave the corresponding thioester (**38aa**) (848 mg, 4.4 mmol, 88%) as a colourless oil.

A solution of compound (**38aa**) (4.0 mmol, 0.77 g, 1.0 equiv.) and NaI (3.0 g, 20 mmol, 5.0 equiv.) in acetone (0.5 M) was heated to reflux for 48 h. The reaction was cooled to r.t., quenched with water (30 mL), extracted with DCM (3 × 40 mL). The combined organic layers were washed with brine (30 mL), dried ($MgSO_4$), filtered and concentrated *in vacuo*. The resulting residue was purified by flash column chromatography (10% EtOAc/hexane) to give the pure compound (**38a**) (0.77 g, 2.7 mmol, 68%) as a pale yellow oil.

TLC: R_f = 0.45 (10% EtOAc/hexane, $KMnO_4$ stain).

^{1}H NMR (400 MHz, $CDCl_3$): δ_H 6.13 (s, 1H), 5.63 (s, 1H), 3.17 (t, *J* = 6.8 Hz, 2H), 2.92 (q, *J* = 7.4 Hz, 2H), 2.52 – 2.39 (m, 2H), 2.05 – 1.93 (m, 2H), 1.27 (t, *J* = 7.4 Hz, 3H) ppm.

^{13}C NMR (101 MHz, $CDCl_3$): δ_C 193.5, 146.5, 123.2, 32.6, 31.7, 23.3, 14.6, 5.8 ppm.

IR (film) ν_{max}: 2968, 2929, 2870, 1656(s), 1449, 1264, 1212, 993, 843, 753 cm^{-1}.

HRMS (ESI^+) calcd. for $C_8H_{14}IOS$ $[M+H]^+$ 284.9805, found 284.9809.

5-Iodo-2-methylenepentanenitrile (39a)

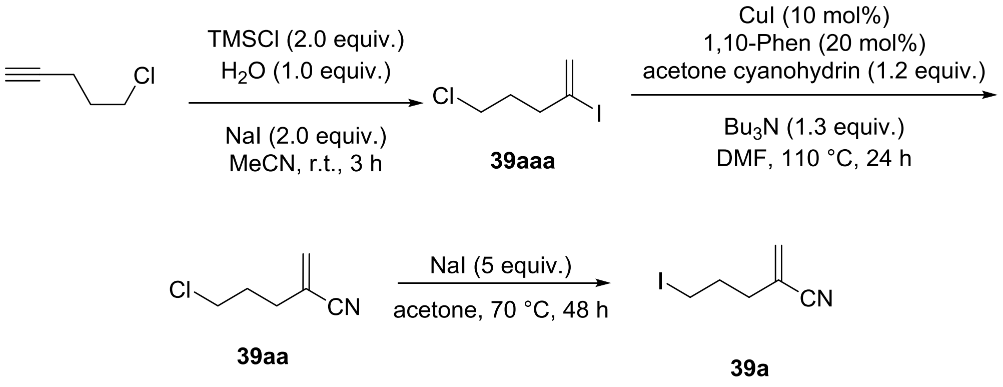

Prepared following a modified literature procedure.[11] To a solution of NaI (7.62 g, 51.0 mmol, 2.00 equiv.) in MeCN (40 mL) at r.t. was added TMSCl (6.48 mL, 51.0 mmol, 2.00 equiv.) followed by H_2O (0.450 mL, 25.5 mmol, 1.00 equiv.), and the cloudy solution was stirred for 10 min. 5-chloro-1-pentyne (2.70 mL, 25.5 mmol, 1.00 equiv.) in MeCN (10 mL) was then added dropwise and the solution stirred for an additional 3 h. The reaction was quenched with H_2O (30 mL) and extracted into Et_2O (3 × 40 mL). The organic extracts were combined and washed with 5% aqueous NaOH (40 mL), brine (50 mL), dried ($MgSO_4$), filtered, and concentrated *in vacuo*. The residue was then purified by flash column chromatography (5% EtOAc/hexane) to give the alkenyl iodide **39aaa** (3.52 g, 15.3 mmol, 60%) as a yellow oil.

A flask was charged with the alkenyl iodide **39aaa** (2.5 g, 11 mmol, 1.0 equiv.), copper iodide (209 mg, 1.10 mmol, 10.0 mol%) and 1,10-phenanthroline (396 mg, 2.20 mmol, 20.0 mol%). The flask was fitted with a septum, evacuated under high vacuum and backfilled with argon. Dry DMF (25 mL) was added and the mixture was stirred at r.t. for 5 min. Tri-*n*-butylamine (3.38 mL, 14.3 mmol, 1.30 equiv.) and acetone cyanohydrin (1.21 mL, 13.2 mmol, 1.20 equiv.) were then successively added and the reaction mixture was heated to 110 °C for 24 h. The resulting solution was cooled to r.t. and diluted with Et_2O (50 mL) and H_2O (50 mL). The organic phase was washed with H_2O (50 mL) and brine (50 mL), dried ($MgSO_4$), filtered, and concentrated *in vacuo*. The residue was purified by flash column chromatography (30% EtOAc/hexane) to afford alkenyl nitrile **39aa** (0.42 g, 3.3 mmol, 30%) as a colourless oil.

A solution of **39aa** (3.0 mmol, 0.39 g, 1.0 equiv.) and NaI (2.2 g, 15 mmol, 5.0 equiv.) in acetone (6.0 mL) was heated to reflux for 48 h. The reaction was cooled to r.t., quenched with water (30 mL), extracted with DCM (3 × 40 mL). The combined organic layers were washed with brine (30 mL), dried ($MgSO_4$), filtered and concentrated *in vacuo*. The residue was purified by flash column chromatography (30% EtOAc/hexane) to give **39a** (0.53 g, 2.4 mmol, 80%) as a pale-yellow oil.

TLC: R_f = 0.40 (30% EtOAc/hexane, $KMnO_4$ stain).

^{1}H NMR (400 MHz, $CDCl_3$): δ_H 5.91 (s, 1H), 5.82 (s, 1H), 3.19 (t, *J* = 6.6 Hz, 2H), 2.41 (t, *J* = 7.2 Hz, 2H), 2.11 – 2.00 (m, 2H) ppm.

^{13}C NMR (101 MHz, $CDCl_3$): δ_C 131.6, 121.1, 118.1, 35.0, 30.4, 4.3 ppm.

IR (film) ν_{max}: 2988, 2952, 2869, 2221, 1621, 1430, 1263, 1212, 937, 733 cm^{-1}.

HRMS (APCI$^+$) calcd. for C_6H_9IN [M+H]$^+$ 221.9774, found 221.9779.

((5-Iodopent-1-en-2-yl)sulfonyl)benzene (40a)

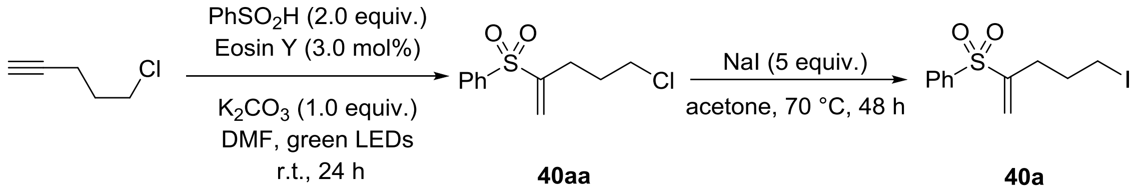

Prepared following a modified literature procedure.[12] A mixture of 5-chloro-1-pentyne (512 mg, 5.00 mmol, 1.00 equiv.), benzenesulphinic acid (1.42 g, 10.0 mmol, 2.00 equiv.), Cs_2CO_3 (1.63 g, 5.00 mmol, 1.00 equiv.), eosin Y (65 mg, 0.010 mmol, 2.0 mol%) in degassed dry DMF (40 mL) was stirred under an N_2 atmosphere and irradiated with green LEDs for 24 h. H_2O (50 mL) was added and the mixture was extracted into EtOAc (3 × 30 mL). The combined organic phases were dried ($MgSO_4$), filtered, and concentrated *in vacuo*. The residue was purified by flash column chromatography (40% EtOAc/hexane) to afford sulfone **40aa** (281 mg, 1.15 mmol, 23%) as a yellow oil.

((5-Iodopent-1-en-2-yl)sulfonyl)benzene (**40aa**) was prepared following the same procedure as described in the synthesis of **39a**, with sulfone **40aa** (270 mg, 1.10 mmol, 1.0 equiv.), NaI (0.82 g, 5.5 mmol, 5.0 equiv.). The crude product was purified by flash column chromatography (40% EtOAc/hexane) to afford **40a** (358 mg, 1.10 mmol, 97%) as a yellow oil.

TLC: R_f = 0.40 (40% EtOAc/hexane, $KMnO_4$ stain).

^{1}H NMR (400 MHz, $CDCl_3$): δ_H 7.89 (d, *J* = 7.5 Hz, 2H), 7.64 (t, *J* = 7.4 Hz, 1H), 7.56 (t, *J* = 7.6 Hz, 2H), 6.40 (s, 1H), 5.80 (s, 1H), 3.11 (t, *J* = 6.6 Hz, 2H), 2.38 (t, *J* = 7.4 Hz, 2H), 2.05 – 1.94 (m, 2H) ppm.

^{13}C NMR (101 MHz, $CDCl_3$): δ_C 148.9, 138.6, 133.6, 129.3, 128.3, 124.4, 31.2, 30.3, 4.8 ppm.

IR (film) ν_{max}: 2988, 2943, 2869, 1584, 1445, 1425, 1302, 1079, 949, 743 cm^{-1}.

HRMS (ESI$^+$) calcd. for $C_{11}H_{13}INaO_2S$ [M+Na]$^+$ 358.9573, found 358.9585.

2-(4-Iodobut-1-en-2-yl)-4,4,5,5-tetramethyl-1,3,2-dioxaborolane (41a)

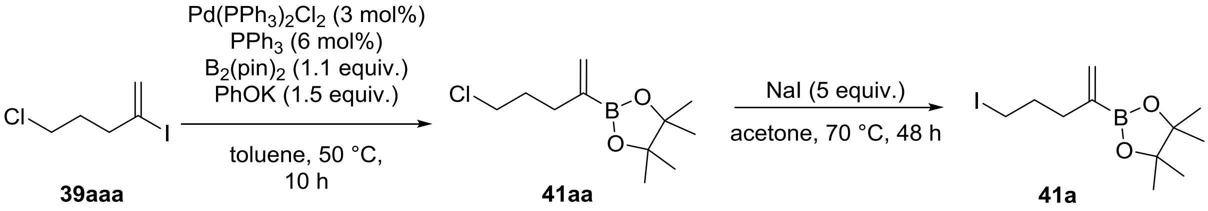

Alkenyl boronic ester **41aa** was prepared following a modified literature procedure:[13] $PdCl_2(PPh_3)_2$ (126 mg, 0.180 mmol, 3.00 mol%), Ph_3P (96 mg, 0.36 mmol, 6.0 mol%), bis(pinacolato)diboron (1.67 g, 6.60 mmol, 1.10 equiv.), and PhOK (fine powder, 1.19 g, 9.00 mmol, 1.50 equiv.) were added to a flask equipped with a magnetic stir bar, a septum inlet, and a condenser. The flask was flushed with N_2 and then charged with toluene (36 mL) and 5-chloro-2-iodopent-1-ene (**39aaa**) (1.38 g, 6.00 mmol, 1.00 equiv.). The reaction was then stirred at 50 °C for 10 h before cooling to r.t. and treating with H_2O (20 mL). The mixture was extracted into Et_2O (3 × 20 mL), washed with brine (20 mL), dried ($MgSO_4$), filtered, and concentrated *in vacuo*. The residue was purified by flash column chromatography (5% Et_2O/pentane) to give **41aa** (968 mg, 4.20 mmol, 70%) as a colourless liquid.

2-(4-Iodobut-1-en-2-yl)-4,4,5,5-tetramethyl-1,3,2-dioxaborolane (**41a**) was prepared following the same procedure as described in the synthesis of **39a**, with boronic ester **41aa** (460 mg, 2.00 mmol, 1.0 equiv.), NaI (1.5 g, 10 mmol, 5.0 equiv.), The crude product was purified by flash column chromatography (5% EtOAc/hexane) to afford **41a** (580 mg, 1.80 mmol, 90%) as a yellow oil.

TLC: R_f = 0.45 (5% EtOAc/hexane, $KMnO_4$ stain).

^{1}H NMR (400 MHz, $CDCl_3$): δ_H 5.82 (s, 1H), 5.66 (s, 1H), 3.16 (t, J = 7.1 Hz, 2H), 2.24 (t, J = 7.1 Hz, 2H), 2.02 – 1.91 (m, 2H), 1.26 (s, 12H) ppm.

^{13}C NMR (101 MHz, $CDCl_3$): δ_C 130.4, 83.5, 36.0, 32.9, 24.8, 6.7 ppm. *The carbon attached to boron could not be observed due to quadrupolar relaxation*.

^{11}B NMR (128 MHz, $CDCl_3$): δ_B 29.3 (br. s, 1B) ppm.

IR (film) ν_{max}: 2978, 2869, 1615, 1445, 1369, 1310, 854, 736 cm^{-1}.

HRMS ($APCI^+$) calcd. for $C_{11}H_{21}BIO_2$ $[M+H]^+$ 323.0674, found 323.0683.

Ethyl 5-iodo-4,4-dimethyl-2-methylenepentanoate (**46a**)

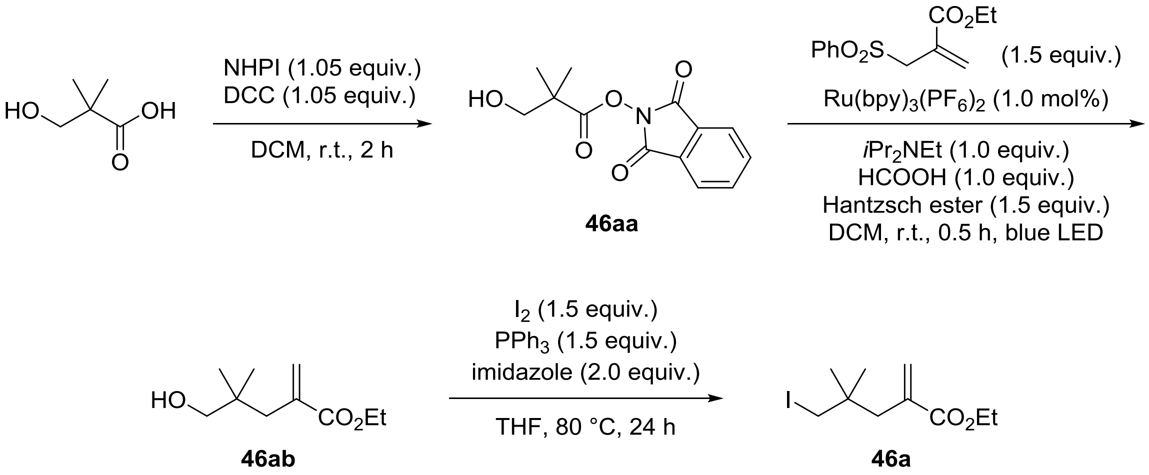

Alcohol **46ab** was prepared following a literature procedure.[14] To a solution of 3-hydroxypivalic acid (1.09 g, 9.20 mmol) and *N*-hydroxyphthalimide (1.63 g, 10.0 mmol) in DCM (40 mL) at 0 °C was added DCC (2.06 g, 10.0 mmol). The reaction was removed from the ice bath and stirred at r.t. for 2 h. The mixture was filtered through Celite, eluting with DCM, and the filtrate was concentrated *in vacuo*. Purification by flash column chromatography (30% EtOAc/hexane) gave *N*-hydroxyphthalimide ester **46aa** (1.69 g, 6.44 mmol) in 70% yield as a white solid.

To a solution of Ru(bpy)$_3$(PF$_6$)$_2$ (19 mg, 0.020 mmol, 1.0 mol%), Hantzsch ester (760 mg, 3.00 mmol, 1.50 equiv.), allyl sulfone (763 mg, 3.00 mmol, 1.50 equiv.), *i*Pr$_2$NEt (258 mg, 2.00 mmol, 1.00 equiv.) and formic acid (92 mg, 2.0 mmol, 1.0 equiv.) in DCM (20 mL) was added *N*-hydroxyphthalimide ester **46aa** (526 mg, 2.00 mmol, 1.00 equiv.). The solution was stirred at r.t., positioned 5 cm from a single 40 W Kessil LED lamp and irradiated for 0.5 h. The reaction was concentrated *in vacuo* and directly the residue purified by falsh column chromatography to give ethyl ester **46ab** (223 mg, 1.20 mmol) in 60% yield as a colourless oil.

Iodide **46a** was prepared following a modified literature procedure.[15] To a solution of alcohol **46ab** (130 mg, 0.700 mmol, 1.00 equiv.) in THF (15 mL) at r.t. was added I$_2$ (253 mg, 1.05 mmol, 1.50 equiv.), Ph$_3$P (262 mg, 1.05 mmol, 1.50 equiv.) and imidazole (95 mg, 1.4 mmol, 2.0 equiv.). The mixture was heating to reflux and stirred for 24 h, before allowing to cool to r.t. and concentrating *in vacuo*. Purification by flash column chromatography (10% EtOAc/hexane) gave iodide **46a** (126 mg, 0.425 mmol, 61%) as a colourless oil.

TLC: R_f = 0.49 (10% EtOAc/hexane, KMnO$_4$ stain).

^{1}H NMR (400 MHz, CDCl$_3$): δ_H 6.26 (d, *J* = 1.6 Hz, 1H), 5.63 (d, *J* = 1.0 Hz, 1H), 4.20 (q, *J* = 7.1 Hz, 2H), 3.16 (s, 2H), 2.46 (s, 2H), 1.30 (t, *J* = 7.1 Hz, 3H), 1.01 (s, 6H) ppm.

^{13}C NMR (101 MHz, CDCl$_3$): δ_C 167.8, 138.0, 127.9, 60.8, 40.8, 34.4, 26.6, 23.9, 14.2 ppm.

IR (film) ν_{max}: 2969, 2901, 1715(s), 1464, 1407, 1367, 1185, 1151, 1066, 949, 891 cm^{-1}.

HRMS (ESI^+): calcd. for $C_{10}H_{18}IO_2$ $[M+H]^+$ 297.0346, found 294.0354.

Methyl 8-iodo-2-methyleneoctanoate (49a)

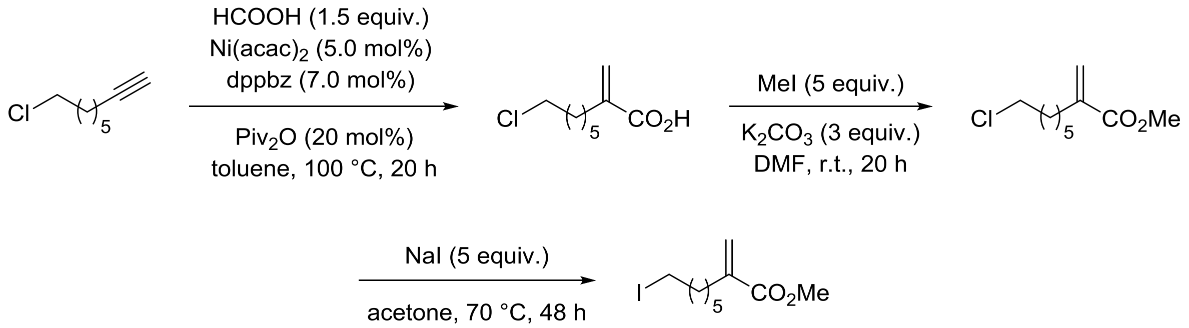

49a

Methyl 8-iodo-2-methylenehexanoate (**49a**) was prepared following the same procedure as described in our previous work,[9] with 8-chlorooct-1-yne (868 mg, 6.00 mmol). The crude product was purified by flash column chromatography (10% EtOAc/hexane) to afford **49a** (1.0 g, 3.4 mmol) as a yellow oil in 57% yield over three steps.

TLC: R_f = 0.45 (10% EtOAc/hexane, $KMnO_4$ stain).

^{1}H NMR (400 MHz, $CDCl_3$): δ_H 6.12 (s, 1H), 5.52 (s, 1H), 3.74 (s, 3H), 3.18 (t, *J* = 7.0 Hz, 2H), 2.29 (t, *J* = 7.5 Hz, 2H), 1.86 – 1.77 (m, 2H), 1.52 – 1.30 (m, 6H) ppm.

^{13}C NMR (101 MHz, $CDCl_3$): δ_C 167.7, 140.5, 124.6, 51.8, 33.4, 31.8, 30.2, 28.2, 28.1, 7.1 ppm.

IR (film) ν_{max}: 2930, 2587, 1718(s), 1630, 1436, 1264, 1196, 941, 816, 733 cm^{-1}.

HRMS (ESI^+) calcd. for $C_{10}H_{17}INaO_2$ $[M+Na]^+$ 319.0165, found 319.0173.

5. Product Characterization

5.1. Cyclobutane Products (Table 2)

Methyl 1-phenethylcyclobutane-1-carboxylate (**10**)

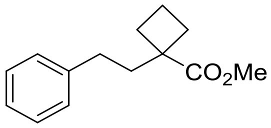

Prepared following **General Procedure A** using 2-benzyl-4,4,5,5-tetramethyl-1,3,2-dioxaborolane (96 mg, 0.44 mmol, 1.1 equiv.), 4CzIPN (16 mg, 0.020 mmol, 5.0 mol%), PhLi (1.9 M in dibutyl ether, 0.25 mL, 0.48 mmol, 1.2 equiv.), methyl 5-iodo-2-methylenepentanoate (**7a**) (101 mg, 0.400 mmol, 1.00 equiv.) and dry DMSO (8.0 mL), which was irradiated with 1 × Kessil lamp for 20 h. Purification by flash column chromatography (5% EtOAc/hexane) gave the title compound (38.4 mg, 0.18 mmol, 44%) as a colourless oil.

TLC: R_f = 0.49 (5% EtOAc/hexane, $KMnO_4$ stain).

^{1}H NMR (400 MHz, $CDCl_3$): δ_H 7.31 – 7.24 (m, 2H), 7.21 – 7.15 (m, 3H), 3.68 (s, 3H), 2.55 – 2.41 (m, 4H), 2.14 – 2.05 (m, 2H), 2.00 – 1.85 (m, 4H) ppm.

^{13}C NMR (101 MHz, $CDCl_3$): δ_C 177.4, 141.9, 128.3, 128.3, 125.8, 51.7, 47.6, 39.8, 31.4, 30.1, 15.6 ppm.

IR (film) ν_{max}: 2988, 2945, 2869, 1726(s), 1603, 1496, 1453, 1161, 739, 697 cm^{-1}.

HRMS (ESI$^+$) calcd. for $C_{14}H_{18}NaO_2$ [M+Na]$^+$ 241.1199, found 241.1190.

Methyl 1-(2-(benzyloxy)ethyl)cyclobutane-1-carboxylate (**11**)

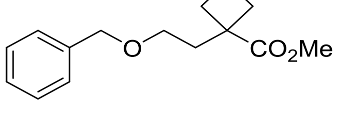

Prepared following **General Procedure A** using 2-((benzyloxy)methyl)-4,4,5,5-tetramethyl-1,3,2-dioxaborolane (109 mg, 0.440 mmol, 1.10 equiv.), 4CzIPN (16 mg, 0.020 mmol, 5.0 mol%), PhLi (1.9 M in dibutyl ether, 0.25 mL, 0.48 mmol, 1.2 equiv.), methyl 5-iodo-2-methylenepentanoate (**7a**) (101 mg, 0.400 mmol, 1.00 equiv.) and dry DMSO (8.0 mL), which was irradiated with 1 × Kessil lamp for 20 h. Purification by flash column chromatography (10% EtOAc/hexane) gave the title compound (49.7 mg, 0.20 mmol, 50%) as a colourless oil.

TLC: R_f = 0.44 (10% EtOAc/hexane, $KMnO_4$ stain).

^{1}H NMR (400 MHz, $CDCl_3$): δ_H 7.38 – 7.22 (m, 5H), 4.44 (s, 2H), 3.61 (s, 3H), 3.45 (t, *J* = 6.6 Hz, 2H), 2.49 – 2.38 (m, 2H), 2.14 (t, *J* = 6.6 Hz, 2H), 2.00 – 1.84 (m, 4H) ppm.

^{13}C NMR (101 MHz, $CDCl_3$): δ_C 177.4, 138.4, 128.3, 127.6, 127.5, 73.1, 67.0, 51.7, 45.8, 37.6, 30.2, 30.2, 15.7 ppm.

IR (film) ν_{max}: 2947, 2988, 2869, 1728(s), 1495, 1453, 1435, 1362, 1202, 1028, 735 cm^{-1}.

HRMS (ESI^+) calcd. for $C_{15}H_{20}NaO_3$ $[M+Na]^+$ 271.1305, found 271.1308.

Methyl 1-(3-phenylpropyl)cyclobutane-1-carboxylate (**12**)

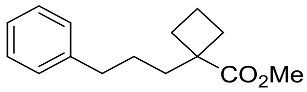

Prepared following **General Procedure A** using 4,4,5,5-tetramethyl-2-phenethyl-1,3,2-dioxaborolane (102 mg, 0.440 mmol, 1.10 equiv.), 4CzIPN (16 mg, 0.020 mmol, 5.0 mol%), PhLi (1.9 M in dibutyl ether, 0.25 mL, 0.48 mmol, 1.2 equiv.), methyl 5-iodo-2-methylenepentanoate (**7a**) (101 mg, 0.400 mmol, 1.00 equiv.) and dry DMSO (8.0 mL), which was irradiated with 1 × Kessil lamp for 20 h. Purification by flash column chromatography (5% EtOAc/hexane) gave the title compound (41.8 mg, 0.18 mmol, 45%) as a colourless oil.

TLC: R_f = 0.49 (5% EtOAc/hexane, $KMnO_4$ stain).

^{1}H NMR (400 MHz, $CDCl_3$): δ_H 7.29 – 7.22 (m, 2H), 7.19 – 7.12 (m, 3H), 3.65 (s, 3H), 2.58 (t, *J* = 7.7 Hz, 2H), 2.45 – 2.32 (m, 2H), 1.90 – 1.74 (m, 6H), 1.54 – 1.43 (m, 2H) ppm.

^{13}C NMR (101 MHz, $CDCl_3$): δ_C 177.7, 142.2, 128.3, 128.3, 125.7, 51.7, 47.6, 37.7, 36.0, 30.1, 26.7, 15.6 ppm.

IR (film) ν_{max}: 2988, 2940, 2869, 1727(s), 1603, 1496, 1452, 1206, 1112, 747, 698 cm^{-1}.

HRMS (ESI^+) calcd. for $C_{15}H_{20}NaO_2$ $[M+Na]^+$ 255.1356, found 255.1358.

Methyl 1-(4-methoxy-4-oxobutyl)cyclobutane-1-carboxylate (**13**)

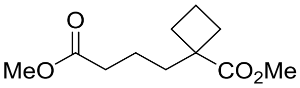

Prepared following **General Procedure A** using methyl 3-(4,4,5,5-tetramethyl-1,3,2-dioxaborolan-2-yl)propanoate (94 mg, 0.44 mmol, 1.1 equiv.), 4CzIPN (16 mg, 0.020 mmol, 5.0 mol%), PhLi (1.9 M in dibutyl ether, 0.25 mL, 0.48 mmol, 1.2 equiv.), methyl 5-iodo-2-methylenepentanoate (**7a**) (101 mg, 0.400 mmol, 1.00 equiv.) and dry DMSO (8.0 mL), which was irradiated with 1 × Kessil lamp for 20 h. Purification by flash column chromatography (10% EtOAc/hexane) gave the title compound (51.4 mg, 0.24 mmol, 60%) as a colourless oil.

TLC: R_f = 0.30 (10% EtOAc/pentane, $KMnO_4$ stain).

^{1}H NMR (400 MHz, $CDCl_3$): δ_H 3.69 (s, 3H), 3.67 (s, 3H), 2.47 – 2.37 (m, 2H), 2.30 (t, J = 7.4 Hz, 2H), 1.95 – 1.86 (m, 4H), 1.81 – 1.75 (m, 2H), 1.56 – 1.47 (m, 2H) ppm.

^{13}C NMR (101 MHz, $CDCl_3$): δ_C 177.4, 173.8, 51.7, 51.5, 47.5, 37.4, 34.1, 30.0, 20.5, 15.6 ppm.

IR (film) ν_{max}: 2988, 2950, 2869, 1728(s), 1435, 1333, 1199, 1001, 926, 693 cm^{-1}.

HRMS (ESI^+) calcd. for $C_{11}H_{18}NaO_4$ $[M+Na]^+$ 237.1097, found 237.1100.

Methyl 1-(4-cyanobutyl)cyclobutane-1-carboxylate (**14**)

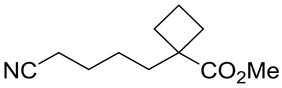

Prepared following **General Procedure A** using 4-(4,4,5,5-tetramethyl-1,3,2-dioxaborolan-2-yl)butanenitrile (86 mg, 0.44 mmol, 1.1 equiv.), 4CzIPN (16 mg, 0.020 mmol, 5.0 mol%), PhLi (1.9 M in dibutyl ether, 0.25 mL, 0.48 mmol, 1.2 equiv.), methyl 5-iodo-2-methylenepentanoate (**7a**) (101 mg, 0.400 mmol, 1.00 equiv.) and dry DMSO (8.0 mL), which was irradiated with 1 × Kessil lamp for 20 h. Purification by flash column chromatography (50% EtOAc/hexane) gave the title compound (17.2 mg, 0.088 mmol, 22%) as a colourless oil.

TLC: R_f = 0.30 (30% EtOAc/hexane, $KMnO_4$ stain).

^{1}H NMR (400 MHz, $CDCl_3$): δ_H 3.69 (s, 3H), 2.46 – 2.38 (m, 2H), 2.33 (t, J = 7.2 Hz, 2H), 1.94 – 1.84 (m, 4H), 1.81 – 1.75 (m, 2H), 1.69 – 1.60 (m, 2H), 1.38 – 1.28 (m, 2H) ppm.

^{13}C NMR (400 MHz, $CDCl_3$): δ_C 177.3, 119.5, 51.8, 47.4, 37.1, 30.0, 25.5, 24.2 17.0, 15.6 ppm.

IR (film) v_{max}: 2988, 2944, 2869, 2246, 1726(s), 1435, 1359, 1208, 1117, 858 cm^{-1}.

HRMS (ESI$^+$): calcd. for $C_{11}H_{17}NNaO_2$ [M+Na]$^+$ 218.1151, found 218.1155.

Methyl 1-(4,4-diethoxybutyl)cyclobutane-1-carboxylate (**15**)

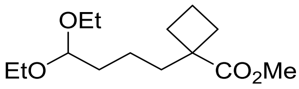

Prepared following **General Procedure A** using 2-(3,3-diethoxypropyl)-4,4,5,5-tetramethyl-1,3,2-dioxaborolane (114 mg, 0.440 mmol, 1.10 equiv.), 4CzIPN (16 mg, 0.020 mmol, 5.0 mol%), PhLi (1.9 M in dibutyl ether, 0.25 mL, 0.48 mmol, 1.2 equiv.), methyl 5-iodo-2-methylenepentanoate (**7a**) (101 mg, 0.400 mmol, 1.00 equiv.) and dry DMSO (8.0 mL), which was irradiated with 1 × Kessil lamp for 20 h. Purification by flash column chromatography (10% EtOAc/hexane) gave the title compound (51.7 mg, 0.20 mmol, 50%) as a colourless oil.

TLC: R_f = 0.32 (10% EtOAc/hexane, $KMnO_4$ stain).

^{1}H NMR (400 MHz, $CDCl_3$): δ_H 4.45 (t, J = 5.8 Hz, 1H), 3.67 (s, 3H), 3.66 – 3.56 (m, 2H), 3.51 – 3.42 (m, 2H), 2.45 – 2.34 (m, 2H), 1.93 – 1.81 (m, 4H), 1.79 – 1.73 (m, 2H), 1.63 – 1.54 (m, 2H), 1.27 – 1.12 (m, 8H) ppm.

^{13}C NMR (101 MHz, $CDCl_3$): δ_C 177.6, 102.7, 61.0, 51.6, 47.7, 37.8, 33.8, 30.0, 20.3, 15.6, 15.3 ppm.

IR (film) v_{max}: 2978, 2947, 2870, 1730(s), 1443, 1378, 1195, 1059, 844 cm^{-1}.

HRMS (ESI$^+$) calcd. for $C_{14}H_{26}NaO_4$ [M+Na]$^+$ 281.1723, found 281.1731.

Methyl 1-(3-(9*H*-carbazol-9-yl)propyl)cyclobutane-1-carboxylate (**16**)

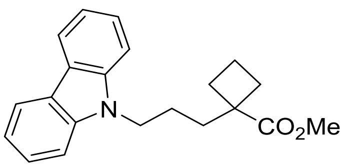

Prepared following **General Procedure A** using 9-(2-(4,4,5,5-tetramethyl-1,3,2-dioxaborolan-2-yl)ethyl)-9*H*-carbazole (141 mg, 0.440 mmol, 1.10 equiv.), 4CzIPN (16 mg, 0.020 mmol, 5.0 mol%), PhLi (1.9 M in dibutyl ether, 0.25 mL, 0.48 mmol, 1.2 equiv.), methyl 5-iodo-2-methylenepentanoate (**7a**) (101 mg, 0.400 mmol, 1.00 equiv.) and dry DMSO (8.0 mL), which was irradiated with 1 × Kessil

lamp for 20 h. Purification by flash column chromatography (20% EtOAc/hextane) gave the title compound (65.6 mg, 0.20 mmol, 51%) as a colourless oil.

TLC: R_f = 0.55 (20% EtOAc/hexane, $KMnO_4$ stain).

^{1}H NMR (400 MHz, $CDCl_3$): δ_H 8.12 (d, J = 7.8 Hz, 2H), 7.51 – 7.46 (m, 2H), 7.41 (d, J = 8.2 Hz, 2H), 7.28 – 7.22 (m, 2H), 4.31 (t, J = 6.8 Hz, 2H), 3.61 (s, 3H), 2.45 – 2.34 (m, 2H), 1.91 – 1.76 (m, 8H) ppm.

^{13}C NMR (101 MHz, $CDCl_3$): δ_C 177.3, 140.3, 125.6, 122.8, 120.3, 118.8, 108.6, 51.7, 47.4, 43.0, 35.3, 30.0, 24.5, 15.5 ppm.

IR (film) ν_{max}: 2977, 2869, 1727(s), 1596, 1484, 1452, 1325, 1142, 844, 749 cm^{-1}.

HRMS (ESI$^+$) calcd. for $C_{21}H_{23}NNaO_2$ $[M+Na]^+$ 344.1621, found 344.1613.

Methyl 1-(cyclohexylmethyl)cyclobutane-1-carboxylate (**8**)

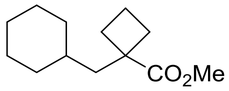

Prepared following **General Procedure A** using 2-cyclohexyl-4,4,5,5-tetramethyl-1,3,2-dioxaborolane (92 mg, 0.44 mmol, 1.1 equiv.), 4CzIPN (16 mg, 0.020 mmol, 5.0 mol%), PhLi (1.9 M in dibutyl ether, 0.25 mL, 0.48 mmol, 1.2 equiv.), methyl 5-iodo-2-methylenepentanoate (**7a**) (101 mg, 0.400 mmol, 1.00 equiv.) and dry DMSO (8.0 mL), which was irradiated with 1 × Kessil lamp for 20 h. Purification by flash column chromatography (5% EtOAc/hexane) gave the title compound (61.4 mg, 0.29 mmol, 73%) as a colourless oil.

TLC: R_f = 0.49 (5% EtOAc/hexane, $KMnO_4$ stain).

^{1}H NMR (400 MHz, $CDCl_3$): δ_H 3.67 (s, 3H), 2.47 – 2.35 (m, 2H), 1.97 – 1.80 (m, 4H), 1.70 (d, J = 6.6 Hz, 2H), 1.67 – 1.52 (m, 5H), 1.27 – 1.05 (m, 4H), 0.96 – 0.80 (m, 2H) ppm.

^{13}C NMR (101 MHz, $CDCl_3$): δ_C 178.2, 51.5, 47.5, 46.2, 35.5, 33.7, 31.5, 26.3, 26.3, 15.9 ppm.

IR (film) ν_{max}: 2988, 2921, 2852, 1729(s), 1447, 1308, 1196, 843, 750 cm^{-1}.

HRMS (ESI$^+$) calcd. for $C_{13}H_{22}NaO_2$ $[M+Na]^+$ 233.1512, found 233.1518.

Methyl 1-(cycloheptylmethyl)cyclobutane-1-carboxylate (**17**)

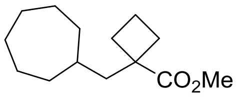

Prepared following **General Procedure A** using 2-cycloheptyl-4,4,5,5-tetramethyl-1,3,2-dioxaborolane (99 mg, 0.44 mmol, 1.1 equiv.), 4CzIPN (16 mg, 0.020 mmol, 5.0 mol%), PhLi (1.9 M in dibutyl ether, 0.25 mL, 0.48 mmol, 1.2 equiv.), methyl 5-iodo-2-methylenepentanoate (**7a**) (101 mg, 0.400 mmol, 1.00 equiv.) and dry DMSO (8.0 mL), which was irradiated with 1 × Kessil lamp for 20 h. Purification by flash column chromatography (5% EtOAc/hexane) gave the title compound (58.3 mg, 0.26 mmol, 65%) as a colourless oil.

TLC: R_f = 0.49 (5% EtOAc/hexane, $KMnO_4$ stain).

^{1}H NMR (400 MHz, $CDCl_3$): δ_H 3.68 (s, 3H), 2.45 – 2.37 (m, 2H), 1.96 – 1.80 (m, 4H), 1.74 (d, *J* = 6.6 Hz, 2H), 1.60 – 1.49 (m, 6H), 1.48 – 1.29 (m, 5H), 1.19 – 1.07 (m, 2H) ppm.

^{13}C NMR (101 MHz, $CDCl_3$): δ_C 178.3, 51.5, 47.8, 46.7, 37.2, 35.0, 31.5, 28.4, 26.0, 16.0 ppm.

IR (film) ν_{max}: 2988, 2918, 2856, 1728(s), 1459, 1201, 1156, 842, 737 cm^{-1}.

HRMS (ESI$^+$) calcd. for $C_{14}H_{24}NaO_2$ [M+Na]$^+$ 247.1669, found 247.1669.

Methyl 1-(cyclododecylmethyl)cyclobutane-1-carboxylate (**18**)

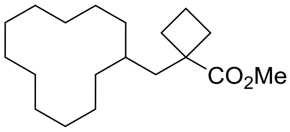

Prepared following **General Procedure A** using 2-cyclododecyl-4,4,5,5-tetramethyl-1,3,2-dioxaborolane (129 mg, 0.440 mmol, 1.10 equiv.), 4CzIPN (16 mg, 0.020 mmol, 5.0 mol%), PhLi (1.9 M in dibutyl ether, 0.25 mL, 0.48 mmol, 1.2 equiv.), methyl 5-iodo-2-methylenepentanoate (**7a**) (101 mg, 0.400 mmol, 1.00 equiv.) and dry DMSO (8.0 mL), which was irradiated with 1 × Kessil lamp for 20 h. Purification by flash column chromatography (5% EtOAc/hexane) gave the title compound (90.7 mg, 0.31 mmol, 77%) as a colourless oil.

TLC: R_f = 0.49 (5% EtOAc/hexane, $KMnO_4$ stain).

^{1}H NMR (400 MHz, $CDCl_3$): δ_H 3.68 (s, 3H), 2.47 – 2.37 (m, 2H), 1.98 – 1.81 (m, 3H), 1.72 (d, *J* = 6.1 Hz, 2H), 1.42 – 1.24 (m, 22H), 1.17 – 1.06 (m, 2H) ppm.

^{13}C NMR (101 MHz, $CDCl_3$): δ_C 178.2, 51.5, 48.0, 44.0, 31.6, 31.4, 29.9, 24.4, 23.7, 23.6, 23.5, 21.9, 16.0 ppm.

IR (film) ν_{max}: 2988, 2930, 2862, 1730(s), 1445, 1196, 927, 842, 737 cm^{-1}.

HRMS (ESI^+) calcd. for $C_{19}H_{34}NaO_2$ $[M+Na]^+$ 317.2451, found 317.2446.

Methyl 1-((tetrahydro-2*H*-pyran-4-yl)methyl)cyclobutane-1-carboxylate (**19**)

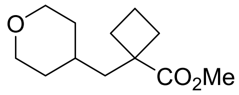

Prepared following **General Procedure A** using 4,4,5,5-tetramethyl-2-(tetrahydro-2H-pyran-4-yl)-1,3,2-dioxaborolane (93 mg, 0.44 mmol, 1.1 equiv.), 4CzIPN (16 mg, 0.020 mmol, 5.0 mol%), PhLi (1.9 M in dibutyl ether, 0.25 mL, 0.48 mmol, 1.2 equiv.), methyl 5-iodo-2-methylenepentanoate (**7a**) (101 mg, 0.400 mmol, 1.00 equiv.) and dry DMSO (8.0 mL), which was irradiated with 1 × Kessil lamp for 20 h. Purification by flash column chromatography (10% EtOAc/hexane) gave the title compound (63.7 mg, 0.30 mmol, 76%) as a colourless oil.

TLC: R_f = 0.45 (10% EtOAc/hexane, $KMnO_4$ stain).

^{1}H NMR (400 MHz, $CDCl_3$): δ_H 3.90 – 3.84 (m, 2H), 3.68 (s, 3H), 3.33 – 3.26 (m, 2H), 2.47 – 2.37 (m, 2H), 1.97 – 1.82 (m, 4H), 1.75 (d, *J* = 6.1 Hz, 2H), 1.49 – 1.39 (m, 3H), 1.31 – 1.20 (m, 2H) ppm.

^{13}C NMR (101 MHz, $CDCl_3$): δ_C 177.9, 67.9, 51.6, 47.2, 45.5, 33.3, 32.9, 31.4, 16.0 ppm.

IR (film) ν_{max}: 2988, 2948, 2869, 1727(s), 1442, 1239, 1199, 1015, 845, 753 cm^{-1}.

HRMS (ESI^+) calcd. for $C_{12}H_{20}NaO_3$ $[M+Na]^+$ 235.1305, found 235.1301.

***tert*-Butyl 4-((1-(methoxycarbonyl)cyclobutyl)methyl)piperidine-1-carboxylate** (**20**)

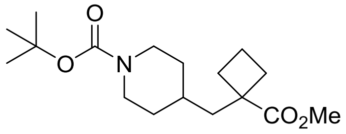

Prepared following **General Procedure A** using *tert*-butyl 4-(4,4,5,5-tetramethyl-1,3,2-dioxaborolan-2-yl)piperidine-1-carboxylate (137 mg, 0.440 mmol, 1.10 equiv.), 4CzIPN (16 mg, 0.020 mmol, 5.0 mol%), PhLi (1.9 M in dibutyl ether, 0.25 mL, 0.48 mmol, 1.2 equiv.), methyl 5-iodo-2-methylenepentanoate (**7a**) (101 mg, 0.400 mmol, 1.00 equiv.) and dry DMSO (8.0 mL), which was

irradiated with 1 × Kessil lamp for 20 h. Purification by flash column chromatography (20% EtOAc/hexane) gave the title compound (94.6 mg, 0.30 mmol, 76%) as a colourless oil.

TLC: R_f = 0.48 (20% EtOAc/hexane, $KMnO_4$).

^{1}H NMR (400 MHz, $CDCl_3$): δ_H 4.01 (br. s, 2H), 3.68 (s, 3H), 2.60 (br. t, *J* = 12.9 Hz, 2H), 2.49 – 2.36 (m, 2H), 1.98 – 1.81 (m, 4H), 1.75 (d, *J* = 6.7 Hz, 2H), 1.51 (d, *J* = 13.3 Hz, 2H), 1.43 (s, 9H), 1.40 – 1.29 (m, 1H), 1.13 – 1.02 (m, 2H) ppm.

^{13}C NMR (101 MHz, $CDCl_3$): δ_C 177.9, 154.8, 79.2, 51.7, 47.3, 45.2, 43.8, 33.9, 32.4, 31.4, 28.4, 16.0 ppm.

IR (film) ν_{max}: 2977, 2933, 2859, 1730(s), 1690(s), 1423, 1365, 1242, 1046, 864, 732 cm^{-1}.

HRMS (ESI^+) calcd. for $C_{17}H_{29}NNaO_4$ $[M+Na]^+$ 334.1989, found 334.1987.

***tert*-Butyl 2-((1-(methoxycarbonyl)cyclobutyl)methyl)piperidine-1-carboxylateylate** (**21**)

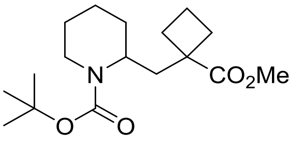

Prepared following **General Procedure A** using *tert*-butyl 2-(4,4,5,5-tetramethyl-1,3,2-dioxaborolan-2-yl)piperidine-1-carboxylate (137 mg, 0.440 mmol, 1.10 equiv.), 4CzIPN (16 mg, 0.020 mmol, 5.0 mol%), PhLi (1.9 M in dibutyl ether, 0.25 mL, 0.48 mmol, 1.2 equiv.), methyl 5-iodo-2-methylenepentanoate (**7a**) (101 mg, 0.400 mmol, 1.00 equiv.) and dry DMSO (8.0 mL), which was irradiated with 1 × Kessil lamp for 20 h. Purification by flash column chromatography (20% EtOAc/hexane) gave the title compound (61.0 mg, 0.20 mmol, 49%) as a colourless oil.

TLC: R_f = 0.48 (20% EtOAc/hexane, $KMnO_4$).

^{1}H NMR (400 MHz, $CDCl_3$): δ_H 4.25 (br. s, 1H), 3.92 (br. s, 1H), 3.64 (s, 3H), 2.76 (br. t, *J* = 13.2 Hz, 1H), 2.55 – 2.45 (m, 1H), 2.39 – 2.29 (m, 2H), 2.02 – 1.85 (m, 4H), 1.79 (dd, *J* = 14.1, 5.1 Hz, 1H), 1.60 – 1.49 (m, 4H), 1.43 (s, 9H), 1.46 – 1.28 (m, 2H) ppm.

^{13}C NMR (101 MHz, $CDCl_3$): δ_C 177.0, 154.5, 79.2, 51.8, 47.9 (br.), 46.3, 37.9, 31.3, 29.9, 29.6, 28.4, 25.6, 19.4, 15.9 ppm.

IR (film) ν_{max}: 2978, 2935, 2868, 1731(s), 1686(s), 1413, 1363, 1199, 1161, 866, 767 cm^{-1}.

HRMS (ESI^+) calcd. for $C_{17}H_{29}NNaO_4$ $[M+Na]^+$ 334.1989, found 334.1987.

***tert*-Butyl 2-((1-(methoxycarbonyl)cyclobutyl)methyl)pyrrolidine-1-carboxylate** (**22**)

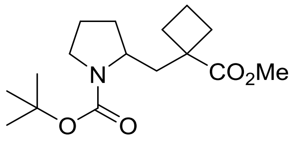

Prepared following **General Procedure A** using *tert*-butyl 2-(4,4,5,5-tetramethyl-1,3,2-dioxaborolan-2-yl)pyrrolidine-1-carboxylate (131 mg, 0.440 mmol, 1.10 equiv.), 4CzIPN (16 mg, 0.020 mmol, 5.0 mol%), PhLi (1.9 M in dibutyl ether, 0.25 mL, 0.48 mmol, 1.2 equiv.), methyl 5-iodo-2-methylenepentanoate (**7a**) (101 mg, 0.400 mmol, 1.00 equiv.) and dry DMSO (8.0 mL), which was irradiated with 1 × Kessil lamp for 20 h. Purification by flash column chromatography (20% EtOAc/hexane) gave the title compound (83.3 mg, 0.28 mmol, 70%) as a colourless oil.

The d.r. was determined to be 66:34 by NMR in $CDCl_3$.

TLC: R_f = 0.48 (20% EtOAc/hexane, $KMnO_4$ stain).

^{1}H NMR (400 MHz, $CDCl_3$): δ_H 3.85 – 3.64 (br. m, 1H), 3.67 (s, 3H), 3.40 – 3.19 (br. m, 2H), 2.49 – 2.32 (m, 2H), 2.29 – 2.02 (m, 2H), 1.99 – 1.70 (m, 7H), 1.49 – 1.38 (m, 1H), 1.46 (s, 9H) ppm.

^{13}C NMR (101 MHz, $CDCl_3$): Rotameric mixture: δ_C 177.7, 154.4, 79.4 + 78.7 (rotameric peaks), 54.3, 51.6, 46.2 + 45.7 (rotameric peaks), 42.2, 32.4, 30.5 + 30.2 (rotameric peaks), 29.0, 28.6, 23.8 + 23.0 (rotameric peaks), 15.9 (undetermined) ppm.

IR (film) ν_{max}: 2978, 2870, 1728(s), 1688(s), 1399, 1364, 1248, 1105, 877, 734 cm^{-1}.

HRMS (ESI^+) calcd. for $C_{16}H_{27}NNaO_4$ $[M+Na]^+$ 320.1832, found 320.1830.

Methyl 1-(bicyclo[2.2.1]heptan-2-ylmethyl)cyclobutane-1-carboxylate (**23**)

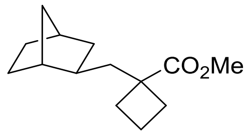

Prepared following **General Procedure A** using 2-((1*S*,4*S*)-bicyclo[2.2.1]heptan-2-yl)-4,4,5,5-tetramethyl-1,3,2-dioxaborolane (98 mg, 0.44 mmol, 1.1 equiv.), 4CzIPN (16 mg, 0.020 mmol, 5.0 mol%), PhLi (1.9 M in dibutyl ether, 0.25 mL, 0.48 mmol, 1.2 equiv.), methyl 5-iodo-2-methylenepentanoate (**7a**) (101 mg, 0.400 mmol, 1.00 equiv.) and dry DMSO (8.0 mL), which was irradiated with 1 × Kessil lamp for 20 h. Purification by flash column chromatography (5% EtOAc/hexane) gave the title compound (62.2 mg, 0.28 mmol, 70%, >20:1 d.r.) as a colourless oil.

TLC: R_f = 0.49 (5% EtOAc/hexane, $KMnO_4$ stain).

^{1}H NMR (400 MHz, $CDCl_3$): δ_H 3.67 (s, 3H), 2.47 – 2.33 (m, 2H), 2.18 – 2.12 (m, 1H), 1.96 – 1.76 (m, 6H), 1.65 – 1.57 (m, 1H), 1.47 – 1.23 (m, 5H), 1.13 – 1.02 (m, 3H), 1.01 – 0.93 (m, 1H) ppm.

^{13}C NMR (101 MHz, $CDCl_3$): δ_C 178.4, 51.5, 47.8, 46.3, 41.8, 39.1, 38.9, 36.6, 35.5, 31.1, 30.9, 30.0, 28.6, 15.8 ppm.

IR (film) ν_{max}: 2987, 2946, 2869, 1729(s), 1453, 1303, 1196, 1089, 981, 738 cm^{-1}.

HRMS (ESI^+): calcd. for $C_{14}H_{22}NaO_2$ $[M+Na]^+$ 245.1512, found 245.1499.

Methyl 1-isobutylcyclobutane-1-carboxylate (**24**)

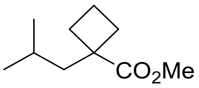

Prepared following **General Procedure A** using 2-isopropyl-4,4,5-trimethyl-1,3,2-dioxaborolane (69 mg, 0.44 mmol, 1.1 equiv.), 4CzIPN (16 mg, 0.020 mmol, 5.0 mol%), PhLi (1.9 M in dibutyl ether, 0.25 mL, 0.48 mmol, 1.2 equiv.), methyl 5-iodo-2-methylenepentanoate (**7a**) (101 mg, 0.400 mmol, 1.00 equiv.) and dry DMSO (8.0 mL), which was irradiated with 1 × Kessil lamp for 20 h. Purification by flash column chromatography (5% EtOAc/hexane) gave the title compound (50.4 mg, 0.30 mmol, 74%) as a colourless oil.

TLC: R_f = 0.49 (5% EtOAc/hexane, $KMnO_4$ stain).

^{1}H NMR (400 MHz, $CDCl_3$): δ_H 3.68 (s, 3H), 2.48 – 2.38 (m, 2H), 1.98 – 1.81 (m, 4H), 1.71 (d, J = 6.9 Hz, 2H), 1.53 (hept, J = 6.7 Hz, 1H), 0.83 (d, J = 6.6 Hz, 6H) ppm.

^{13}C NMR (101 MHz, $CDCl_3$): δ_C 178.1, 51.5, 47.8, 47.5, 31.5, 26.1, 23.1, 16.0 ppm.

IR (film) ν_{max}: 2978, 2869, 1733(s), 1453, 1381, 1031, 928, 748 cm^{-1}.

HRMS (ESI^+) calcd. for $C_{10}H_{19}O_2$ $[M+H]^+$ 171.1380, found 171.1378.

Methyl 1-(2-methylheptyl)cyclobutane-1-carboxylate (**25**)

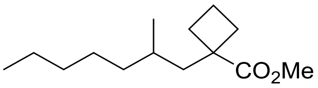

Prepared following **General Procedure A** using 2-(heptan-2-yl)-4,4,5,5-tetramethyl-1,3,2-dioxaborolane (99 mg, 0.44 mmol, 1.1 equiv.), 4CzIPN (16 mg, 0.020 mmol, 5.0 mol%), PhLi (1.9 M in dibutyl ether, 0.25 mL, 0.48 mmol, 1.2 equiv.), methyl 5-iodo-2-methylenepentanoate (**7a**) (101 mg, 0.400 mmol, 1.00 equiv.) and dry DMSO (8.0 mL), which was irradiated with 1 × Kessil lamp for 20 h. Purification by flash column chromatography (5% EtOAc/hexane) gave the title compound (54.3 mg, 0.24 mmol, 60%) as a colourless oil.

TLC: R_f = 0.49 (5% EtOAc/hexane, $KMnO_4$ stain).

^{1}H NMR (400 MHz, $CDCl_3$): δ_H 3.68 (s, 3H), 2.47 – 2.37 (m, 2H), 1.99 – 1.79 (m, 5H), 1.66 – 1.57 (m, 1H), 1.38 – 1.15 (m, 8H), 1.11 – 1.02 (m, 1H), 0.87 (t, *J* = 7.0 Hz, 3H), 0.78 (d, *J* = 6.7 Hz, 3H) ppm.

^{13}C NMR (101 MHz, $CDCl_3$): δ_C 178.2, 51.5, 47.8, 45.9, 37.7, 32.1, 31.9, 31.2, 30.8, 26.5, 22.6, 19.9, 16.0, 14.1 ppm

IR (film) ν_{max}: 2988, 2870, 1732(s), 1448, 1391, 1195, 929, 841, 752 cm^{-1}.

HRMS (ESI$^+$) calcd. for $C_{14}H_{26}NaO_2$ [M+Na]$^+$ 249.1825, found 249.1812.

Methyl 1-(4-(4-methoxyphenyl)-2-methylbutyl)cyclobutane-1-carboxylate (**26**)

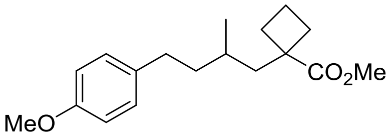

Prepared following **General Procedure A** using 2-(4-(4-methoxyphenyl)butan-2-yl)-4,4,5,5-tetramethyl-1,3,2-dioxaborolane (128 mg, 0.440 mmol, 1.10 equiv.), 4CzIPN (16 mg, 0.020 mmol, 5.0 mol%), PhLi (1.9 M in dibutyl ether, 0.25 mL, 0.48 mmol, 1.2 equiv.), methyl 5-iodo-2-methylenepentanoate (**7a**) (101 mg, 0.400 mmol, 1.00 equiv.) and dry DMSO (8.0 mL), which was irradiated with 1 × Kessil lamp for 20 h. Purification by flash column chromatography (5% EtOAc/hexane) gave the title compound (87.1 mg, 0.30 mmol, 75%) as a colourless oil.

TLC: R_f = 0.33 (5% EtOAc/hexane, $KMnO_4$ stain).

^{1}H NMR (400 MHz, $CDCl_3$): δ_H 7.07 (d, *J* = 8.6 Hz, 2H), 6.81 (d, *J* = 8.6 Hz, 2H), 3.78 (s, 3H), 3.65 (s, 3H), 2.63 – 2.38 (m, 4H), 2.00 – 1.82 (m, 5H), 1.67 (dd, *J* = 13.8, 8.0 Hz, 1H), 1.59 – 1.49 (m, 1H), 1.48 – 1.33 (m, 2H), 0.86 (d, *J* = 6.3 Hz, 3H) ppm.

^{13}C NMR (101 MHz, $CDCl_3$): δ_C 178.0, 157.6, 134.8, 129.1, 113.7, 55.2, 51.5, 47.8, 45.7, 39.7, 32.3, 31.9, 31.3, 30.5, 19.8, 16.0 ppm.

IR (film) ν_{max}: 2977, 2869, 1729(s), 1612, 1512, 1456, 1380, 1244, 1038, 823 cm^{-1}.

HRMS (ESI^+) calcd. for $C_{18}H_{26}NaO_3$ $[M+Na]^+$ 313.1774, found 313.1752.

Methyl 1-(4-(*tert*-butoxy)-2-methyl-4-oxobutyl)cyclobutane-1-carboxylate (**27**)

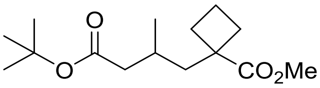

Prepared following **General Procedure A** using *tert*-butyl 3-(4,4,5,5-tetramethyl-1,3,2-dioxaborolan-2-yl)butanoate (119 mg, 0.440 mmol, 1.00 equiv.), 4CzIPN (16 mg, 0.020 mmol, 5.0 mol%), PhLi (1.9 M in dibutyl ether, 0.25 mL, 0.48 mmol, 1.2 equiv.), methyl 5-iodo-2-methylenepentanoate (**7a**) (101 mg, 0.400 mmol, 1.00 equiv.) and dry DMSO (8.0 mL), which was irradiated with 1 × Kessil lamp for 20 h. Purification by flash column chromatography (10% EtOAc/hexane) gave the title compound (64.9 mg, 0.24 mmol, 60%) as a colourless oil.

TLC: R_f = 0.31 (10% EtOAc/hexane, $KMnO_4$ stain).

^{1}H NMR (400 MHz, $CDCl_3$): δ_H 3.68 (s, 3H), 2.48 – 2.36 (m, 2H), 2.14 (dd, *J* = 14.3, 5.9 Hz, 1H), 2.03 – 1.80 (m, 7H), 1.75 – 1.66 (m, 1H), 1.43 (s, 9H), 0.84 (d, *J* = 6.5 Hz, 3H) ppm.

^{13}C NMR (101 MHz, $CDCl_3$): δ_C 177.8, 172.1, 80.1, 51.6, 47.5, 44.9, 43.5, 32.1, 30.7, 28.5, 28.1, 19.7, 16.0 ppm.

IR (film) ν_{max}: 2979, 2870, 1726(s), 1456, 1366, 1256, 967, 842 cm^{-1}.

HRMS (ESI^+) calcd. for $C_{15}H_{26}NaO_4$ $[M+Na]^+$ 293.1723, found 293.1722.

Methyl 1-neopentylcyclobutane-1-carboxylate (**28**)

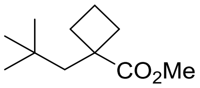

Prepared following **General Procedure A** using 2-(*tert*-butyl)-4,4,5-trimethyl-1,3,2-dioxaborolane (75 mg, 0.44 mmol, 1.1 equiv.), 4CzIPN (16 mg, 0.020 mmol, 5.0 mol%), PhLi (1.9 M in dibutyl ether, 0.25 mL, 0.48 mmol, 1.2 equiv.), methyl 5-iodo-2-methylenepentanoate (**7a**) (101 mg, 0.400 mmol, 1.00 equiv.) and dry DMSO (8.0 mL), which was irradiated with 1 × Kessil lamp for 20 h. Purification by flash column chromatography (5% EtOAc/hexane) gave the title compound (44.2 mg, 0.24 mmol, 60%) as a colourless oil.

TLC: R_f = 0.49 (5% EtOAc/hexane, $KMnO_4$ stain).

^{1}H NMR (400 MHz, $CDCl_3$): δ_H 3.68 (s, 3H), 2.47 – 2.38 (m, 2H), 2.07 – 1.98 (m, 2H), 1.93 – 1.77 (m, 4H), 0.84 (s, 9H) ppm.

^{13}C NMR (101 MHz, $CDCl_3$): δ_C 178.6, 52.6, 51.4, 47.9, 33.2, 32.0, 30.2, 16.7 ppm

IR (film) ν_{max}: 2988, 2870, 1735(s),1360, 1245, 929, 800 cm^{-1}.

HRMS (ESI$^+$) calcd. for $C_{11}H_{21}O_2$ [M+H]$^+$ 185.1536, found 185.1532.

Methyl 1-(((3*r*,5*r*,7*r*)-adamantan-1-yl)methyl)cyclobutane-1-carboxylate (**29**)

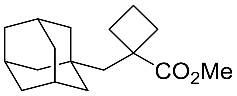

Prepared following **General Procedure A** using 2-((3*r*,5*r*,7*r*)-adamantan-1-yl)-4,4,5,5-tetramethyl-1,3,2-dioxaborolane (115 mg, 0.440 mmol, 1.10 equiv.), 4CzIPN (16 mg, 0.020 mmol, 5.0 mol%), PhLi (1.9 M in dibutyl ether, 0.25 mL, 0.48 mmol, 1.2 equiv.), methyl 5-iodo-2-methylenepentanoate (**7a**) (101 mg, 0.400 mmol, 1.00 equiv.) and dry DMSO (8.0 mL), which was irradiated with 1 × Kessil lamp for 20 h. Purification by flash column chromatography (5% EtOAc/hexane) gave the title compound (65.1 mg, 0.25 mmol, 62%) as a colourless oil.

TLC: R_f = 0.49 (5% EtOAc/hexane, $KMnO_4$ stain).

^{1}H NMR (400 MHz, $CDCl_3$): δ_H 3.68 (s, 3H), 2.46 – 2.37 (m, 2H), 2.06 – 1.96 (m, 2H), 1.92 – 1.77 (m, 5H), 1.67 – 1.56 (m, 8H), 1.44 (d, *J* = 2.6 Hz, 6H) ppm.

^{13}C NMR (101 MHz, $CDCl_3$): δ_C 178.8, 53.8, 51.4, 47.0, 42.8, 36.9, 34.1, 33.3, 28.7, 24.6, 16.7 ppm.

IR (film) v_{max}: 2988, 2847, 1728(s), 1447, 1292, 1204, 1083, 986, 751 cm^{-1}.

HRMS (ESI$^+$) calcd. for $C_{17}H_{26}NaO_2$ [M+Na]$^+$ 285.1825, found 285.1817.

Methyl 1-(((2S,5R)-2-isopropyl-5-methylcyclohexyl)methyl)cyclobutane-1-carboxylate (**30**)

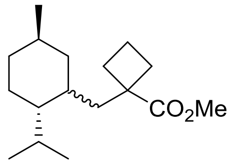

Prepared following **General Procedure A** using 2-((1*R*,2*R*,5*R*)-2-isopropyl-5-methylcyclohexyl)-4,4,5,5-tetramethyl-1,3,2-dioxaborolane (117 mg, 0.440 mmol, 1.10 equiv.), 4CzIPN (16 mg, 0.020 mmol, 5.0 mol%), PhLi (1.9 M in dibutyl ether, 0.25 mL, 0.48 mmol, 1.2 equiv.), methyl 5-iodo-2-methylenepentanoate (**7a**) (101 mg, 0.400 mmol, 1.00 equiv.) and dry DMSO (8.0 mL), which was irradiated with 1 × Kessil lamp for 20 h. Purification by flash column chromatography (5% EtOAc/hexane) gave the title compound (71.4 mg, 0.27 mmol, 67%, 65:35 d.r.) as a colourless oil.

TLC: R_f = 0.49 (3% EtOAc/hexane, $KMnO_4$).

^{1}H NMR (400 MHz, $CDCl_3$): 65:35 ratio of diastereomers: δ_H 3.674 (s, 1.95 H), 3.669 (s, 1.05 H), 2.55 – 2.44 (m, 1H), 2.43 – 2.32 (m, 1H), 2.16 (dd, *J* = 13.7, 3.1 Hz, 0.65H), 2.07 – 1.47 (m, 9.35H), 1.43 – 1.32 (m, 1H), 1.26 – 0.89 (m, 3H), 0.89 – 0.76 (m, 2H), 0.88 (d, *J* = 6.9 Hz, 1.95H), 0.87 (d, *J* = 6.5 Hz, 1.05H), 0.83 (d, *J* = 6.6 Hz, 1.05H), 0.80 (d, *J* = 6.5 Hz, 1.95H), 0.77 (d, *J* = 6.4 Hz, 1.05H), 0.69 (d, *J* = 6.9 Hz, 1.95H) ppm.

^{13}C NMR (101 MHz, $CDCl_3$): 65:35 ratio of diastereomers: δ_C 178.2 (minor), 178.1 (major), 51.5 (major), 51.4 (minor), 48.8 (major), 48.1 (minor), 47.7 (minor), 47.5 (major), 42.0 (major), 41.3 (major), 38.1 (minor), 37.4 (major), 35.9 (minor), 35.1 (major), 33.6 (minor), 32.9 (minor), 32.6 (major), 32.5 (major), 32.2 (minor), 31.5 (major), 31.1 (minor), 29.1 (minor), 26.34 (major), 26.32 (minor), 25.1 (minor), 24.2 (major), 22.8 (major), 22.5 (minor), 21.7 (minor), 21.6 (major), 20.4 (minor), 16.3 (minor), 16.2 (major), 15.0 (major) ppm.

IR (film) v_{max}: 2947, 2869, 1728(s), 1455, 1264, 1196, 737, 704 cm^{-1}.

HRMS (ESI$^+$) calcd. for $C_{17}H_{30}NaO_2$ [M+Na]$^+$ 289.2138, found 289.2143.

Methyl 1-(((1*S*,2*R*,3*R*,5*S*)-2,6,6-trimethylbicyclo[3.1.1]heptan-3-yl)methyl)cyclobutane-1-carboxylate (**31**)

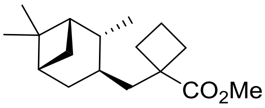

Prepared following **General Procedure A** using 4,4,5,5-tetramethyl-2-((1*R*,2*R*,3*S*,5*R*)-3,6,6-trimethylbicyclo[3.1.1]heptan-2-yl)-1,3,2-dioxaborolane (116 mg, 0.440 mmol, 1.10 equiv.), 4CzIPN (16 mg, 0.020 mmol, 5.0 mol%), PhLi (1.9 M in dibutyl ether, 0.25 mL, 0.48 mmol, 1.2 equiv.), methyl 5-iodo-2-methylenepentanoate (**7a**) (101 mg, 0.400 mmol, 1.00 equiv.) and dry DMSO (8.0 mL), which was irradiated with 1 × Kessil lamp for 20 h. Purification by flash column chromatography (5% EtOAc/hexane) gave the title compound (63.4 mg, 0.24 mmol, 60%, >20:1 d.r.) as a colourless oil.

TLC: R_f = 0.49 (5% EtOAc/hexane, $KMnO_4$ stain).

^{1}H NMR (400 MHz, $CDCl_3$): δ_H 3.69 (s, 3H), 2.49 – 2.33 (m, 2H), 2.34 – 2.23 (m, 1H), 2.05 – 1.70 (m, 8H), 1.63 – 1.52 (m, 1H), 1.45 – 1.35 (m, 1H), 1.30 – 1.21 (m, 1H), 1.16 (s, 3H), 0.98 (d, *J* = 6.6 Hz, 3H), 0.96 (s, 3H), 0.93 – 0.70 (m, 2H) ppm.

^{13}C NMR (101 MHz, $CDCl_3$): δ_C 178.3, 51.5, 49.7, 48.2, 48.1, 44.6, 42.1, 38.6, 34.9, 34.7, 34.2, 32.1, 30.2, 28.2, 23.0, 20.8, 16.0 ppm.

IR (film) ν_{max}: 2982, 2948, 1726(s), 1452, 1264, 1201, 1143, 856, 704 cm^{-1}.

HRMS (ESI^+) calcd. for $C_{17}H_{28}NaO_2$ $[M+Na]^+$ 287.1982, found 287.1959.

$[\alpha]_D^{24}$: –12 (*c* = 1.0, $CHCl_3$)

Methyl 1-(5-(2,5-dimethylphenoxy)-2,2-dimethylpentyl)cyclobutane-1-carboxylate (**32**)

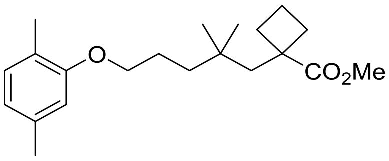

Prepared following **General Procedure A** using 2-(5-(2,5-dimethylphenoxy)-2-methylpentan-2-yl)-4,4,5,5-tetramethyl-1,3,2-dioxaborolane (146 mg, 0.440 mmol, 1.10 equiv.), 4CzIPN (16 mg, 0.020 mmol, 5.0 mol%), PhLi (1.9 M in dibutyl ether, 0.25 mL, 0.48 mmol, 1.2 equiv.), methyl 5-iodo-2-methylenepentanoate (**7a**) (101 mg, 0.400 mmol, 1.00 equiv.) and dry DMSO (8.0 mL), which was irradiated with 1 × Kessil lamp for 20 h. Purification by flash column chromatography (5% EtOAc/hexane) gave the title compound (106.4 mg, 0.32 mmol, 80%) as a colourless oil.

TLC: R_f = 0.45 (5% EtOAc/hexane, $KMnO_4$ stain).

^{1}H NMR (400 MHz, $CDCl_3$): δ_H 7.02 (d, *J* = 7.4 Hz, 1H), 6.67 (d, *J* = 7.6 Hz, 1H), 6.63 (s, 1H), 3.90 (t, *J* = 6.5 Hz, 2H), 3.70 (s, 3H), 2.51 – 2.42 (m, 2H), 2.32 (s, 3H), 2.20 (s, 3H), 2.11 – 2.02 (m, 2H), 1.97 – 1.73 (m, 4H), 1.88 (s, 2H), 1.38 – 1.29 (m, 2H), 0.87 (s, 6H). ppm.

^{13}C NMR (101 MHz, $CDCl_3$): δ_C 178.6, 157.0, 136.4, 130.2, 123.5, 120.5, 111.9, 68.5, 51.5, 50.6, 47.8, 39.8, 34.2, 33.3, 27.1, 24.2, 21.4, 16.8, 15.8 ppm.

IR (film) ν_{max}: 2977, 2948, 2870, 1727(s), 1585, 1508, 1470, 1264, 1202, 1155, 907, 803, 730 cm^{-1}.

HRMS (ESI^+) calcd. for $C_{21}H_{32}NaO_3$ $[M+Na]^+$ 355.2244, found 355.2259.

Methyl 1-(((3*S*,5*R*,6*R*,8*S*,9*S*,10*S*,13*R*,14*S*,17*R*)-3-((tert-butyldimethylsilyl)oxy)-10,13-dimethyl-17-((*R*)-6-methylheptan-2-yl)hexadecahydro-1*H*-cyclopenta[a]phenanthren-6-yl)methyl)cyclobutane-1-carboxylate (**33**)

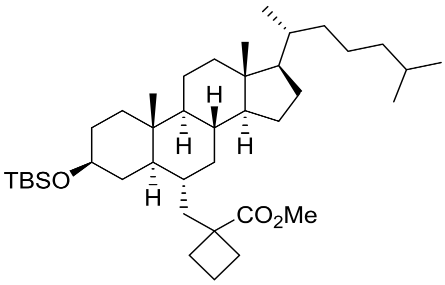

Prepared following **General Procedure A** using *tert*-butyl(((3*S*,5*R*,6*S*,8*R*,9*S*,10*S*,13*R*,14*S*,17*R*)-10,13-dimethyl-17-((*R*)-6-methylheptan-2-yl)-6-(4,4,5,5-tetramethyl-1,3,2-dioxaborolan-2-yl)hexadecahydro-1*H*-cyclopenta[a]phenanthren-3-yl)oxy)dimethylsilane (277 mg, 0.440 mmol, 1.10 equiv.), 4CzIPN (16 mg, 0.020 mmol, 5.0 mol%), PhLi (1.9 M in dibutyl ether, 0.25 mL, 0.48 mmol, 1.2 equiv.), methyl 5-iodo-2-methylenepentanoate (**7a**) (101 mg, 0.400 mmol, 1.00 equiv.) and dry DMSO (8.0 mL), which was irradiated with 1 × Kessil lamp for 20 h. Purification by flash column chromatography (5% EtOAc/hexane) gave the title compound (176.1 mg, 0.28 mmol, 70%, >20:1 d.r.) as a colourless oil.

TLC: R_f = 0.49 (5% EtOAc/hextane, $KMnO_4$).

^{1}H NMR (400 MHz, $CDCl_3$): δ_H 3.67 (s, 3H), 3.53 – 3.42 (m, 1H), 2.45 – 2.35 (m, 2H), 2.02 (dd, *J* = 13.7, 3.1 Hz, 1H), 1.98 – 1.74 (m, 7H), 1.70 – 1.62 (m, 2H), 1.57 – 1.43 (m, 5H), 1.41 – 1.28 (m, 6H), 1.28 – 1.18 (m, 4H), 1.16 – 1.01 (m, 8H), 0.93 – 0.82 (m, 21H), 0.77 (s, 3H), 0.61 (s, 3H), 0.06 (s, 3H), 0.06 (s, 3H) ppm.

^{13}C NMR (101 MHz, $CDCl_3$): δ_C 178.2, 72.7, 56.5, 56.3, 54.1, 51.6, 49.7, 47.5, 42.5, 42.1, 40.1, 39.5, 38.7, 37.2, 36.2, 35.9, 35.8, 34.7, 33.9, 33.7, 32.7, 31.6, 30.1, 28.2, 28.0, 26.0, 24.1, 23.8, 22.8, 22.6, 21.2, 18.7, 18.3, 15.9, 13.3, 12.1, -4.5 ppm.

IR (film) ν_{max}: 2979, 2949, 2869, 1724(s), 1381, 1264, 1142, 836, 704 cm^{-1}.

HRMS (ESI$^+$) calcd. for $C_{40}H_{72}NaO_3Si$ $[M+Na]^+$ 651.5143, found 651.5138.

$[\alpha]_D^{24}$: +10 (c = 0.5, $CHCl_3$).

Methyl 1-((*R*)-4-((3*S*,5*R*,8*R*,9*S*,10*S*,13*R*,14*S*,17*R*)-3-((*tert*-butyldimethylsilyl)oxy)-10,13-dimethylhexadecahydro-1*H*-cyclopenta[a]phenanthren-17-yl)pentyl)cyclobutane-1-carboxylate (**34**)

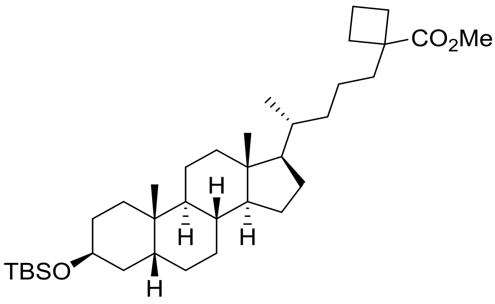

Prepared following **General Procedure A** using *tert*-butyl(((3*S*,5*R*,8*R*,9*S*,10S,13R,14*S*,17*R*)-10,13-dimethyl-17-((*R*)-4-(4,4,5,5-tetramethyl-1,3,2-dioxaborolan-2-yl)butan-2-yl)hexadecahydro-1*H*-cyclopenta[a]phenanthren-3-yl)oxy)dimethylsilane (172 mg, 0.300 mmol, 1.10 equiv.), 4CzIPN (12 mg, 0.015 mmol, 5.0 mol%), PhLi (1.9 M in dibutyl ether, 0.17 mL, 0.33 mmol, 1.20 equiv.), methyl 5-iodo-2-methylenepentanoate (**7a**) (69 mg, 0.27 mmol, 1.0 equiv.) and dry DMSO (6.0 mL), which was irradiated with 1 × Kessil lamp for 20 h. Purification by flash column chromatography (5% EtOAc/hexane) gave the title compound (90.0 mg, 0.16 mmol, 58%) as a colourless oil.

TLC: R_f = 0.49 (5% EtOAc/hexane, $KMnO_4$ stain).

^{1}H NMR (400 MHz, $CDCl_3$): δ_H 3.69 (s, 3H), 3.62 – 3.53 (m, 1H), 2.47 – 2.36 (m, 2H), 1.96 – 1.70 (m, 10H), 1.69 – 1.63 (m, 1H), 1.57 – 1.51 (m, 2H), 1.48 – 1.29 (m, 10H), 1.27 – 1.17 (m, 4H), 1.16 – 0.95 (m, 8H), 0.93 – 0.85 (m, 14H), 0.62 (s, 3H), 0.05 (s, 6H) ppm.

^{13}C NMR (101 MHz, $CDCl_3$): δ_C 177.9, 72.8, 56.4, 56.4, 51.6, 47.8, 42.7, 42.3, 40.2, 40.2, 38.6, 36.9, 36.1, 35.9, 35.7, 35.6, 34.6, 31.0, 30.2, 30.0, 28.3, 27.3, 26.4, 26.0, 24.2, 23.4, 21.6, 20.8, 18.5, 18.3, 15.6, 12.0, -4.6 ppm.

IR (film) ν_{max}: 2930, 2862, 1733(s), 1470, 1378, 1250, 1078, 870, 834, 773 cm^{-1}.

HRMS (ESI^+) calcd. for $C_{36}H_{64}NaO_3Si$ $[M+Na]^+$ 595.4517, found 595.4526.

$[\alpha]_D^{24}$: +19 (c = 1.0, $CHCl_3$).

***tert*-Butyl 4-((1-((benzyloxy)carbonyl)cyclobutyl)methyl)piperidine-1-carboxylate** (**37**)

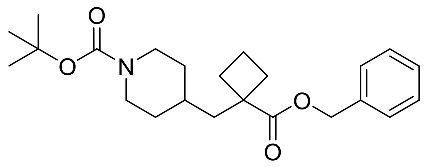

Prepared following **General Procedure B** using *tert*-butyl 4-(4,4,5,5-tetramethyl-1,3,2-dioxaborolan-2-yl)piperidine-1-carboxylate (137 mg, 0.440 mmol, 1.10 equiv.), 4CzIPN (16 mg, 0.020 mmol, 5.0 mol%), PhLi (1.9 M in dibutyl ether, 0.25 mL, 0.48 mmol, 1.2 equiv.), benzyl 5-iodo-2-methylenepentanoate (**37a**) (132 mg, 0.400 mmol, 1.00 equiv.) and dry DMF (8.0 mL), which was irradiated with 1 × Kessil lamp for 20 h. Purification by flash column chromatography (20% EtOAc/hexane) gave the title compound (96.1 mg, 0.25 mmol, 62%) as a colourless oil.

TLC: R_f = 0.45 (20% EtOAc/hextane, $KMnO_4$).

^{1}H NMR (400 MHz, $CDCl_3$): δ_H 7.38 – 7.29 (m, 5H), 5.15 (s, 2H), 3.94 (br. s, 2H), 2.53 – 2.40 (m, 4H), 1.98 – 1.82 (m, 4H), 1.74 (d, *J* = 6.7 Hz, 2H), 1.49 – 1.39 (m, 2H), 1.43 (s, 9H), 1.28 – 1.16 (m, 1H), 1.08 – 0.94 (m, 2H) ppm.

^{13}C NMR (101 MHz, $CDCl_3$): δ_C 177.1, 154.7, 136.2, 128.5, 128.4, 128.2, 79.2, 66.2, 47.5, 45.3, 43.8 (br.), 33.9, 32.4, 31.6, 28.4, 16.1ppm.

IR (film) ν_{max}: 2977, 2938, 2867, 1724, 1686, 1497, 1424, 1364, 1265, 972, 863, 734 cm^{-1}.

HRMS (ESI^+) calcd. for $C_{23}H_{33}NNaO_4$ $[M+Na]^+$ 410.2302, found 410.2305.

***tert*-Butyl 4-((1-((ethylthio)carbonyl)cyclobutyl)methyl)piperidine-1-carboxylate** (**38**)

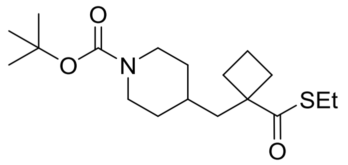

Prepared following **General Procedure B** using *tert*-butyl 4-(4,4,5,5-tetramethyl-1,3,2-dioxaborolan-2-yl)piperidine-1-carboxylate (137 mg, 0.440 mmol, 1.10 equiv.), 4CzIPN (16 mg, 0.020 mmol, 5.0 mol%), PhLi (1.9 M in dibutyl ether, 0.25 mL, 0.48 mmol, 1.2 equiv.), *S*-ethyl 5-iodo-2-

methylenepentanethioate (**38a**) (113 mg, 0.400 mmol, 1.00 equiv.) and dry DMF (8.0 mL), which was irradiated with 1 × Kessil lamp for 20 h. Purification by flash column chromatography (20% EtOAc/hexane) gave the title compound (47.8 mg, 0.14 mmol, 35%) as a colourless oil.

TLC: R_f = 0.45 (20% EtOAc/hextane, $KMnO_4$).

^{1}H NMR (400 MHz, $CDCl_3$): δ_H 4.00 (br. s, 2H), 2.89 (q, *J* = 7.4 Hz, 2H), 2.60 (t, *J* = 12.8 Hz, 2H), 2.53 – 2.44 (m, 2H), 2.01 – 1.83 (m, 4H), 1.81 (d, *J* = 6.6 Hz, 2H), 1.56 (d, *J* = 13.3 Hz, 2H), 1.49 – 1.35 (m, 1H), 1.44 (s, 9H), 1.26 (t, *J* = 7.4 Hz, 3H), 1.16 – 1.03 (m, 2H) ppm.

^{13}C NMR (101 MHz, $CDCl_3$): δ_C 205.5, 154.8, 79.2, 55.2, 45.9, 43.9 (br.), 33.8, 32.6, 32.2, 28.5, 22.9, 16.0, 14.8 ppm.

IR (film) ν_{max}: 2976, 2930, 2869, 1689(s), 1673(s), 1421, 1364, 1243, 946, 865, 768 cm^{-1}.

HRMS (ESI$^+$) calcd. for $C_{18}H_{31}NNaO_3S$ [M+Na]$^+$ 364.1917, found 364.1926.

***tert*-Butyl 4-((1-cyanocyclobutyl)methyl)piperidine-1-carboxylate** (**39**)

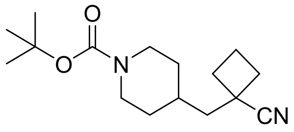

Prepared following **General Procedure B** using *tert*-butyl 4-(4,4,5,5-tetramethyl-1,3,2-dioxaborolan-2-yl)piperidine-1-carboxylate (137 mg, 0.440 mmol, 1.10 equiv.), 4CzIPN (16 mg, 0.020 mmol, 5.0 mol%), PhLi (1.9 M in dibutyl ether, 0.25 mL, 0.48 mmol, 1.2 equiv.), 5-iodo-2-methylenepentanenitrile (**39a**) (88 mg, 0.40 mmol, 1.0 equiv.) and dry DMSO (8.0 mL), which was irradiated with 1 × Kessil lamp for 20 h. Purification by flash column chromatography (40% EtOAc/hexane) gave the title compound (67.9 mg, 0.24 mmol, 61%) as a colourless oil.

TLC: R_f = 0.30 (40% EtOAc/hextane, $KMnO_4$).

^{1}H NMR (400 MHz, $CDCl_3$): δ_H 4.06 (br. s, 2H), 2.68 (br. t, *J* = 12.9 Hz, 2H), 2.54 – 2.45 (m, 2H), 2.28 – 2.16 (m, 1H), 2.15 – 2.05 (m, 2H), 2.02 – 1.91 (m, 1H), 1.72 – 1.63 (m, 5H), 1.44 (s, 9H), 1.22 – 1.08 (m, 2H) ppm.

^{13}C NMR (101 MHz, $CDCl_3$): δ_C 154.7, 124.8, 79.3, 44.8, 43.6 (br.), 35.4, 34.3, 33.7, 32.2, 28.4, 17.1 ppm.

IR (film) ν_{max}: 2978, 2939, 2868, 2228, 1686(s), 1421, 1364, 1243, 974, 864, 735 cm^{-1}.

HRMS (ESI$^+$) calcd. for $C_{16}H_{26}N_2NaO_2$ [M+Na]$^+$ 301.1886, found 301.1874.

***tert*-Butyl 4-((1-(phenylsulfonyl)cyclobutyl)methyl)piperidine-1-carboxylate** (**40**)

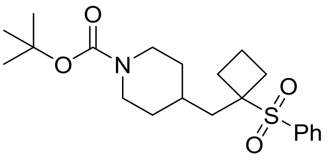

Prepared following **General Procedure B** using *tert*-butyl 4-(4,4,5,5-tetramethyl-1,3,2-dioxaborolan-2-yl)piperidine-1-carboxylate (137 mg, 0.440 mmol, 1.10 equiv.), 4CzIPN (16 mg, 0.020 mmol, 5.0 mol%), PhLi (1.9 M in dibutyl ether, 0.25 mL, 0.48 mmol, 1.2 equiv.), ((5-iodopent-1-en-2-yl)sulfonyl)benzene (**40a**) (134 mg, 0.400 mmol, 1.00 equiv.) and dry DMF (8.0 mL), which was irradiated with 1 × Kessil lamp for 20 h. Purification by flash column chromatography (20% EtOAc/hexane) gave the title compound (72.4 mg, 0.18 mmol, 46%) as a colourless oil.

TLC: R_f = 0.30 (20% EtOAc/hextane, $KMnO_4$).

^{1}H NMR (400 MHz, $CDCl_3$): δ_H 7.93 – 7.85 (m, 2H), 7.69 – 7.60 (m, 1H), 7.59 – 7.54 (m, 2H), 4.02 (br. s, 2H), 2.85 – 2.72 (m, 2H), 2.67 (br. t, *J* = 12.1 Hz, 2H), 2.10 – 1.98 (m, 2H), 1.96 – 1.80 (m, 3H), 1.71 – 1.62 (m, 4H), 1.43 (s, 9H), 1.17 – 1.04 (m, 2H) ppm.

^{13}C NMR (101 MHz, $CDCl_3$): δ_C 154.7, 136.5, 133.6, 129.6, 128.9, 79.3, 65.6, 43.8 (br.), 41.5, 33.4, 28.4, 24.8, 15.0 ppm.

IR (film) ν_{max}: 2927, 2932, 2868, 1682(s), 1446, 1424, 1365, 1166, 912, 724 cm^{-1}.

HRMS (ESI$^+$) calcd. for $C_{21}H_{31}NNaO_4S$ [M+Na]$^+$ 416.1866, found 416.1873.

***tert*-Butyl 4-((1-(4,4,5,5-tetramethyl-1,3,2-dioxaborolan-2-yl)cyclobutyl)methyl)piperidine-1-carboxylate** (**41**)

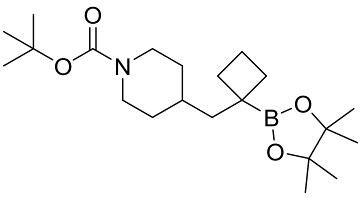

Prepared following **General Procedure B** using *tert*-butyl 4-(4,4,5,5-tetramethyl-1,3,2-dioxaborolan-2-yl)piperidine-1-carboxylate (137 mg, 0.440 mmol, 1.10 equiv.), 4CzIPN (16 mg, 0.020 mmol, 5.0 mol%), PhLi (1.9 M in dibutyl ether, 0.25 mL, 0.48 mmol, 1.2 equiv.), 2-(5-iodopent-1-en-2-yl)-4,4,5,5-tetramethyl-1,3,2-dioxaborolane (**41a**) (129 mg, 0.400 mmol, 1.00 equiv.) and dry DMF (8.0 mL), which was irradiated with 1 × Kessil lamp for 20 h. Purification by flash column chromatography (20% EtOAc/hexane) gave the title compound (51.6 mg, 0.14 mmol, 34%) as a colourless oil.

TLC: R_f = 0.45 (20% EtOAc/hextane, $KMnO_4$).

^{1}H NMR (400 MHz, $CDCl_3$): δ_H 4.02 (br. s, 2H), 2.59 (br. t, *J* = 12.8 Hz, 2H), 2.15 – 2.08 (m, 2H), 1.96 – 1.76 (m, 2H), 1.75 – 1.66 (m, 2H), 1.59 – 1.49 (m, 4H), 1.43 (s, 9H), 1.42 – 1.35 (m, 1H), 1.26 (s, 12H), 1.11 – 0.98 (m, 2H) ppm.

^{13}C NMR (101 MHz, $CDCl_3$): δ_C 154.9, 83.0, 79.1, 47.1, 44.0 (br.), 35.5, 32.5, 31.9, 28.5, 24.8, 24.7, 18.5 ppm.

^{11}B NMR (128 MHz, $CDCl_3$): δ_B 33.9 (br. s, 1B) ppm.

IR (film) ν_{max}: 2976, 2930, 2867, 1690(s), 1421, 1365, 1162, 965, 862, 735 cm^{-1}.

HRMS (ESI^+) calcd. for $C_{21}H_{38}BNNaO_4$ $[M+Na]^+$ 402.2786, found 402.2805.

5.2. Cyclopropane Products (Table 3)

***tert*-Butyl 4-((1-(methoxycarbonyl)cyclopropyl)methyl)piperidine-1-carboxylate** (**42**)

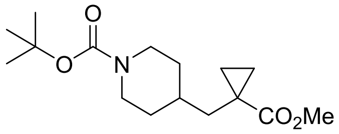

Prepared following **General Procedure B** using *tert*-butyl 4-(4,4,5,5-tetramethyl-1,3,2-dioxaborolan-2-yl)piperidine-1-carboxylate (137 mg, 0.440 mmol, 1.10 equiv.), 4CzIPN (16 mg, 0.020 mmol, 5.0 mol%), PhLi (1.9 M in dibutyl ether, 0.25 mL, 0.48 mmol, 1.2 equiv.), methyl 4-chloro-2-methylenebutanoate (**42a**) (59 mg, 0.40 mmol, 1.0 equiv.) and dry DMSO (8.0 mL), which was irradiated with 1 × Kessil lamp for 20 h. Purification by flash column chromatography (20% EtOAc/hexane) gave the title compound (107.1 mg, 0.36 mmol, 90%) as a colourless oil.

TLC: R_f = 0.48 (20% EtOAc/hexane, $KMnO_4$).

^{1}H NMR (400 MHz, $CDCl_3$): δ_H 4.07 (br. t, *J* = 14.7 Hz, 2H), 3.64 (s, 3H), 2.65 (br. t, *J* = 12.8 Hz, 2H), 1.84 – 1.71 (m, 1H), 1.68 (d, *J* = 9.6 Hz, 2H), 1.48 (d, *J* = 6.9 Hz, 2H), 1.44 (s, 9H), 1.25 – 1.17 (m, 2H), 1.12 – 0.99 (m, 2H), 0.70 – 0.63 (m, 2H) ppm.

^{13}C NMR (101 MHz, $CDCl_3$): δ_C 175.6, 154.8, 79.2, 51.7, 44.0 (br.), 40.8, 35.2, 32.4, 28.5, 21.4, 15.6 ppm.

IR (film) ν_{max}: 2975, 2927, 2849, 1722(s), 1689(s), 1421, 1364, 1243, 1113, 859, 756 cm^{-1}.

HRMS (ESI^+) calcd. for $C_{16}H_{27}NNaO_4$ $[M+Na]^+$ 320.1832, found 320.1832.

***tert*-Butyl 4-((1-cyanocyclopropyl)methyl)piperidine-1-carboxylate** (**43**)

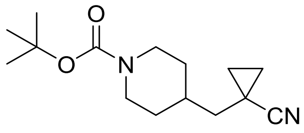

Prepared following **General Procedure B** using *tert*-butyl 4-(4,4,5,5-tetramethyl-1,3,2-dioxaborolan-2-yl)piperidine-1-carboxylate (137 mg, 0.440 mmol, 1.10 equiv.), 4CzIPN (16 mg, 0.020 mmol, 5.0 mol%), PhLi (1.9 M in dibutyl ether, 0.25 mL, 0.48 mmol, 1.2 equiv.), 4-chloro-2-methylenebutanenitrile (**43a**) (46 mg, 0.40 mmol, 1.0 equiv.) and dry DMSO (8.0 mL), which was irradiated with 1 × Kessil lamp for 20 h. Purification by flash column chromatography (40% EtOAc/hexane) gave the title compound (63.4 mg, 0.24 mmol, 60%) as a colourless oil.

TLC: R_f = 0.30 (40% EtOAc/hexane, $KMnO_4$).

^{1}H NMR (400 MHz, $CDCl_3$): δ_H 4.10 (br. s, 2H), 2.73 (br. t, J = 12.9 Hz, 2H), 1.90 – 1.78 (m, 3H), 1.45 (s, 9H), 1.39 (d, J = 6.8 Hz, 2H), 1.27 – 1.22 (m, 2H), 1.17 – 1.05 (m, 2H), 0.80 – 0.75 (m, 2H) ppm.

^{13}C NMR (101 MHz, $CDCl_3$): δ_C 154.8, 123.4, 79.4, 43.7 (br.), 41.7, 35.4, 32.0, 28.4, 14.2, 7.7 ppm.

IR (film) ν_{max}: 2975, 2930, 2852, 2234, 1687(s), 1423, 1365, 1242, 1121, 979, 759 cm^{-1}.

HRMS (ESI$^+$) calcd. for $C_{15}H_{24}N_2NaO_2$ [M+Na]$^+$ 287.1730, found 287.1741.

***tert*-Butyl 4-((1-(4,4,5,5-tetramethyl-1,3,2-dioxaborolan-2-yl)cyclopropyl)methyl)piperidine-1-carboxylate** (**44**)

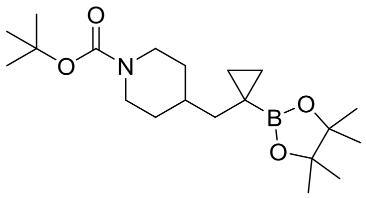

Prepared following **General Procedure B** using *tert*-butyl 4-(4,4,5,5-tetramethyl-1,3,2-dioxaborolan-2-yl)piperidine-1-carboxylate (137 mg, 0.440 mmol, 1.10 equiv.), 4CzIPN (16 mg, 0.020 mmol, 5.0 mol%), PhLi (1.9 M in dibutyl ether, 0.25 mL, 0.48 mmol, 1.2 equiv.), 2-(4-chlorobut-1-en-2-yl)-4,4,5,5-tetramethyl-1,3,2-dioxaborolane (**44a**) (87 mg, 0.40 mmol, 1.0 equiv.) and dry DMF (8.0 mL), which was irradiated with 1 × Kessil lamp for 20 h. Purification by flash column chromatography (20% EtOAc/hexane) gave the title compound (58.4 mg, 0.16 mmol, 40%) as a colourless oil.

TLC: R_f = 0.45 (20% EtOAc/hexane, $KMnO_4$).

^{1}H NMR (400 MHz, $CDCl_3$): δ_H 4.04 (br. s, 2H), 2.65 (br. t, J = 12.1 Hz, 2H), 1.74 – 1.59 (m, 3H), 1.44 (s, 9H), 1.25 – 1.14 (m, 2H), 1.19 (s, 12H), 1.12 – 0.98 (m, 2H), 0.69 – 0.65 (m, 2H), 0.29 – 0.24 (m, 2H) ppm.

^{13}C NMR (101 MHz, $CDCl_3$): δ_C 154.9, 82.9, 79.0, 44.2 (br.), 43.1, 36.6, 32.4, 28.5, 24.5, 11.7 ppm. *The carbon attached to boron could not be observed due to quadrupolar relaxation.*

^{11}B NMR (128 MHz, $CDCl_3$): δ_B 32.9 (br. s, 1B) ppm.

IR (film) ν_{max}: 2976, 2927, 1694(s), 1415, 1365, 1159, 1108, 853 cm^{-1}.

HRMS (ESI$^+$) calcd. for $C_{20}H_{36}BNNaO_4$ [M+Na]$^+$ 388.2630, found 388.2634.

***tert*-Butyl 4-((1-phenylcyclopropyl)methyl)piperidine-1-carboxylate** (**45**)

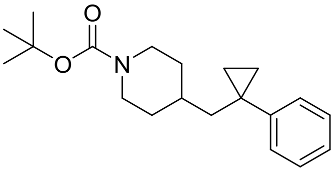

Prepared following **General Procedure B** using *tert*-butyl 4-(4,4,5,5-tetramethyl-1,3,2-dioxaborolan-2-yl)piperidine-1-carboxylate (137 mg, 0.440 mmol, 1.10 equiv.), 4CzIPN (16 mg, 0.020 mmol, 5.0 mol%), PhLi (1.9 M in dibutyl ether, 0.25 mL, 0.48 mmol, 1.2 equiv.), (4-chlorobut-1-en-2-yl)benzene (**45a**) (66 mg, 0.40 mmol, 1.0 equiv.) and dry DMF (8.0 mL), which was irradiated with 1 × Kessil lamp for 20 h. Purification by flash column chromatography (20% EtOAc/hexane) gave the title compound (58.4 mg, 0.16 mmol, 61%) as a colourless oil.

TLC: R_f = 0.45 (20% EtOAc/hexane, $KMnO_4$).

^{1}H NMR (400 MHz, $CDCl_3$): δ_H 7.25 – 7.18 (m, 4H), 7.13 – 7.08 (m , 1H), 3.92 (br. s, 2H), 2.47 (br. t, *J* = 12.7 Hz, 2H), 1.61 (d, *J* = 13.3 Hz, 2H), 1.46 (d, *J* = 6.9 Hz, 2H), 1.36 (s, 9H), 1.32 – 1.19 (m, 1H), 1.03 – 0.90 (m, 2H), 0.76 – 0.72 (m, 2H), 0.58 – 0.53 (m, 2H) ppm.

^{13}C NMR (101 MHz, $CDCl_3$): δ_C 154.8, 145.1, 128.8, 128.2, 125.9, 79.1, 47.2, 44.0 (br.), 34.5, 32.6, 28.5, 23.5, 12.8 ppm.

IR (film) ν_{max}: 2975, 2916, 2847, 1688(s), 1601, 1465, 1420, 1364, 1241, 1128, 975, 867 cm^{-1}.

HRMS (ESI^+) calcd. for $C_{20}H_{29}NNaO_2$ $[M+Na]^+$ 338.2091, found 338.2103.

5.3. Other Cycloalkane Products (Table 3)

***tert*-Butyl 4-((1-(ethoxycarbonyl)-3,3-dimethylcyclobutyl)methyl)piperidine-1-carboxylate (46)**

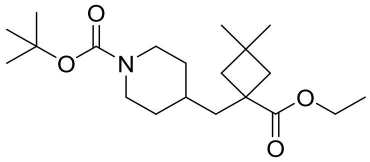

Prepared following **General Procedure A** using *tert*-butyl 4-(4,4,5,5-tetramethyl-1,3,2-dioxaborolan-2-yl)piperidine-1-carboxylate (78 mg, 0.25 mmol, 1.1 equiv.), 4CzIPN (9.9 mg, 0.013 mmol, 5.0 mol%), PhLi (1.9 M in dibutyl ether, 0.145 mL, 0.280 mmol, 1.20 equiv.), ethyl 5-iodo-4,4-dimethyl-2-methylenepentanoate (**46a**) (68 mg, 0.23 mmol, 1.0 equiv.) and dry DMSO (5.0 mL), which was irradiated with 1 × Kessil lamp for 20 h. Purification by flash column chromatography (20% EtOAc/hexane) gave the title compound (56.2 mg, 0.17 mmol, 72%) as a colourless oil.

TLC: R_f = 0.48 (20% EtOAc/hexane, $KMnO_4$).

^{1}H NMR (400 MHz, $CDCl_3$): δ_H 4.15 (q, *J* = 7.1 Hz, 2H), 3.99 (br. s, 2H), 2.58 (br. t, *J* = 12.8 Hz, 2H), 2.31 (d, *J* = 13.0 Hz, 2H), 1.80 – 1.68 (m, 4H), 1.53 (br. d, *J* = 12.7 Hz, 2H), 1.42 (s, 9H), 1.36 – 1.28 (m, 1H), 1.25 (t, *J* = 7.1 Hz, 3H), 1.13 – 0.99 (m, 2H), 1.07 (s, 3H), 1.04 (s, 3H) ppm.

^{13}C NMR (101 MHz, $CDCl_3$): δ_C 178.0, 154.8, 79.1, 60.3, 47.6, 44.8, 43.9 (br.), 40.1, 34.2, 32.3, 31.3, 29.6, 28.4, 14.2 ppm.

IR (film) ν_{max}: 2972, 2926, 1723(s), 1691(s), 1424, 1366, 1246, 1163, 1065, 908, 730 cm^{-1}.

HRMS (ESI^+) calcd. for $C_{20}H_{36}NO_4$ $[M+H]^+$ 354.2639, found 354.2650.

***tert*-Butyl 4-((1-(methoxycarbonyl)cyclopentyl)methyl)piperidine-1-carboxylate** (**47**)

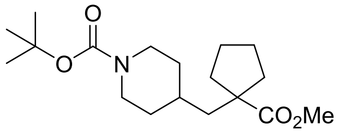

Prepared following **General Procedure B** using *tert*-butyl 4-(4,4,5,5-tetramethyl-1,3,2-dioxaborolan-2-yl)piperidine-1-carboxylate (137 mg, 0.440 mmol, 1.10 equiv.), 4CzIPN (16 mg, 0.020 mmol, 5.0 mol%), PhLi (1.9 M in dibutyl ether, 0.25 mL, 0.48 mmol, 1.2 equiv.), methyl 6-bromo-2-methylenehexanoate (**47a**) (88 mg, 0.40 mmol, 1.0 equiv.) and dry DMSO (8.0 mL), which was irradiated with 1 × Kessil lamp for 20 h. Purification by flash column chromatography (20% EtOAc/hexane) gave the title compound (72.4 mg, 0.18 mmol, 90%) as a colourless oil.

TLC: R_f = 0.45 (20% EtOAc/hexane, $KMnO_4$).

^{1}H NMR (400 MHz, $CDCl_3$): δ_H 3.98 (br. s, 2H), 3.63 (s, 3H), 2.61 (br. t, *J* = 12.8 Hz, 2H), 2.16 – 2.08 (m, 2H), 1.64 – 1.56 (m, 6H), 1.55 – 1.34 (m, 5H), 1.42 (s, 9H), 1.12 – 0.99 (m, 2H) ppm.

^{13}C NMR (101 MHz, $CDCl_3$): δ_C 178.5, 154.7, 79.1, 53.3, 51.6, 45.7, 43.9 (br.), 36.7, 34.0, 32.7, 28.4, 24.5 ppm.

IR (film) ν_{max}: 2976, 2949, 2869, 1726(s), 1687(s), 1423, 1364, 1242, 969, 860, 734 cm^{-1}.

HRMS (ESI^+) calcd. for $C_{18}H_{31}NNaO_4$ $[M+Na]^+$ 348.2145, found 348.2157.

***tert*-Butyl 4-((1-(methoxycarbonyl)cyclohexyl)methyl)piperidine-1-carboxylate** (**48**)

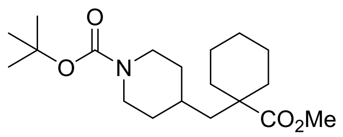

Prepared following **General Procedure B** using *tert*-butyl 4-(4,4,5,5-tetramethyl-1,3,2-dioxaborolan-2-yl)piperidine-1-carboxylate (137 mg, 0.440 mmol, 1.10 equiv.), 4CzIPN (16 mg, 0.020 mmol, 5.0 mol%), PhLi (1.9 M in dibutyl ether, 0.25 mL, 0.48 mmol, 1.2 equiv.), methyl 7-iodo-2-methyleneheptanoate (**48a**) (113 mg, 0.400 mmol, 1.00 equiv.) and dry DMSO (8.0 mL), which was irradiated with 1 × Kessil lamp for 20 h. Purification by flash column chromatography (20% EtOAc/hexane) gave the title compound (108.6 mg, 0.32 mmol, 80%) as a colourless oil.

TLC: R_f = 0.45 (20% EtOAc/hexane, $KMnO_4$).

^{1}H NMR (400 MHz, $CDCl_3$): δ_H 3.98 (br. s, 2H), 3.66 (s, 3H), 2.64 (br. t, *J* = 12.8 Hz, 2H), 2.05 (d, *J* = 12.5 Hz, 2H), 1.60 – 1.46 (m, 8H), 1.43 (s, 9H), 1.39 – 1.29 (m, 2H), 1.28 – 1.16 (m, 3H), 1.12 – 0.98 (m, 2H) ppm.

^{13}C NMR (101 MHz, $CDCl_3$): δ_C 177.7, 154.8, 79.2, 51.4, 46.9, 46.1, 44.0 (br.), 34.8, 33.3, 31.8, 28.4, 25.8, 23.0 ppm.

IR (film) ν_{max}: 2977, 2929, 2856, 1727(s), 1689(s), 1450, 1364, 1209, 1004, 859, 735 cm^{-1}.

HRMS (ESI^+) calcd. for $C_{19}H_{33}NNaO_4$ $[M+Na]^+$ 362.2302, found 362.2302.

tert-Butyl 4-((1-(methoxycarbonyl)cycloheptyl)methyl)piperidine-1-carboxylate (49)

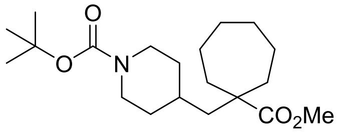

Prepared following **General Procedure B** using _tert_-butyl 4-(4,4,5,5-tetramethyl-1,3,2-dioxaborolan-2-yl)piperidine-1-carboxylate (137 mg, 0.440 mmol, 1.10 equiv.), 4CzIPN (16 mg, 0.020 mmol, 5.0 mol%), PhLi (1.9 M in dibutyl ether, 0.25 mL, 0.48 mmol, 1.2 equiv.), methyl 8-iodo-2-methyleneoctanoate **49a** (118 mg, 0.400 mmol, 1.00 equiv.) and dry DMSO (8.0 mL), which was irradiated with 1 × Kessil lamp for 20 h. Purification by flash column chromatography (20% EtOAc/hexane) gave the title compound (31.1 mg, 0.09 mmol, 22%) as a colourless oil.

TLC: R_f = 0.48 (20% EtOAc/hexane, $KMnO_4$).

^{1}H NMR (400 MHz, $CDCl_3$): δ_H 4.00 (br. s, 2H), 3.65 (s, 3H), 2.63 (br. t, _J_ = 12.7 Hz, 2H), 2.09 – 2.01 (m, 2H), 1.55 – 1.41 (m, 13H), 1.44 (s, 9H), 1.32 – 1.21 (m, 2H), 1.14 – 0.99 (m, 2H) ppm.

^{13}C NMR (101 MHz, $CDCl_3$): δ_C 178.9, 154.8, 79.2, 51.5, 48.6, 43.9 (br.), 47.2, 36.7, 33.0, 32.4, 30.0, 28.5, 23.4 ppm.

IR (film) ν_{max}: 2988, 2869, 1722(s), 1681(s), 1391, 1264, 1071, 896, 732 cm^{-1}.

HRMS (ESI$^+$) calcd. for $C_{20}H_{35}NNaO_4$ $[M+Na]^+$ 376.2458, found 376.2454.

6. Unsuccessful Substrates

During our studies, we found that halide-tethered alkenes bearing β-substitution (**SI-1a** and **SI-2a**) failed to undergo the desired radical addition–polar cyclization cascade with boronic ester **35**.

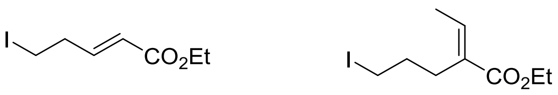

SI-1a **SI-2a**

Reaction of boronic ester **35** with methyl (*E*)-5-iodopent-2-enoate (**SI-1a**) following **General Procedure B** failed to provide 1,2-disubstituted cyclobutane **SI-1**.

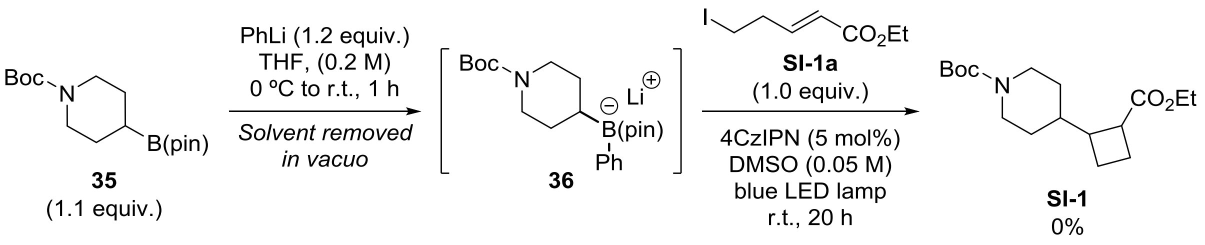

Reaction of boronic ester **35** with ethyl (*E*)-2-ethylidene-5-iodopentanoate (**SI-2a**) following **General Procedure B** failed to provide cyclobutane **SI-2**.

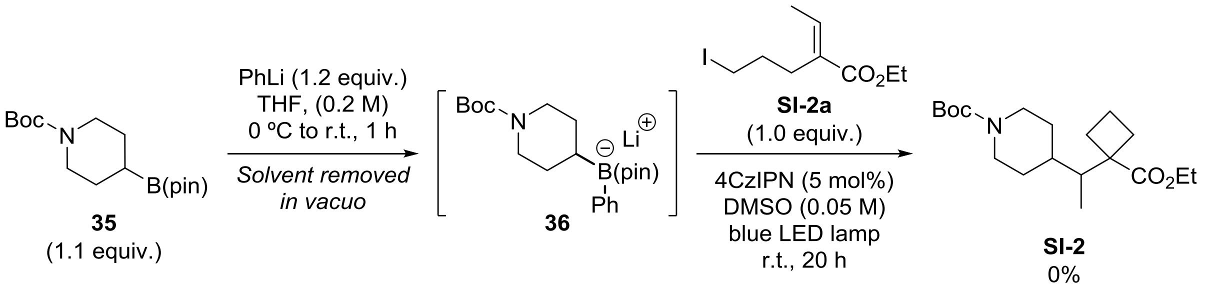

(*E*)-5-Iodopent-2-enoate (**SI-1a**)

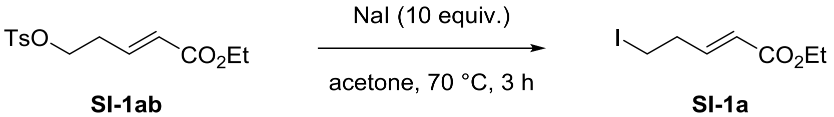

SI-1ab **SI-1a**

To a solution of **SI-1ab**[16] (328 mg, 1.10 mmol, 1.00 equiv.) in acetone (10 mL) at r.t. was added NaI (1.65 g, 11.0 mmol, 10.0 equiv.). The mixture was stirred at 70 °C for 3 h before the solid was removed by filtration, and the filtrate was concentrated *in vacuo*. Purification by flash column chromatography (10% EtOAc/hexane) gave ester **SI-1a** (190 mg, 0.750 mmol) in 68% yield as a yellow oil. All recorded spectroscopic data matched those previously reported in the literature.[17]

^{1}**H NMR** (400 MHz, $CDCl_3$): δ_H 6.83 (dt, *J* = 15.7, 6.9 Hz, 1H), 5.88 (dt, *J* = 15.7, 1.5 Hz, 1H), 4.19 (q, *J* = 7.1 Hz, 2H), 3.20 (t, *J* = 7.1 Hz, 2H), 2.79 (qd, *J* = 7.0, 1.5 Hz, 2H), 1.29 (t, *J* = 7.1 Hz, 3H) ppm.

^{13}**C NMR** (101 MHz, $CDCl_3$): δ_C 166.1, 146.0, 123.3, 60.4, 35.8, 14.2, 1.5 ppm.

Ethyl (*E*)-2-ethylidene-5-iodopentanoate (SI-2a):

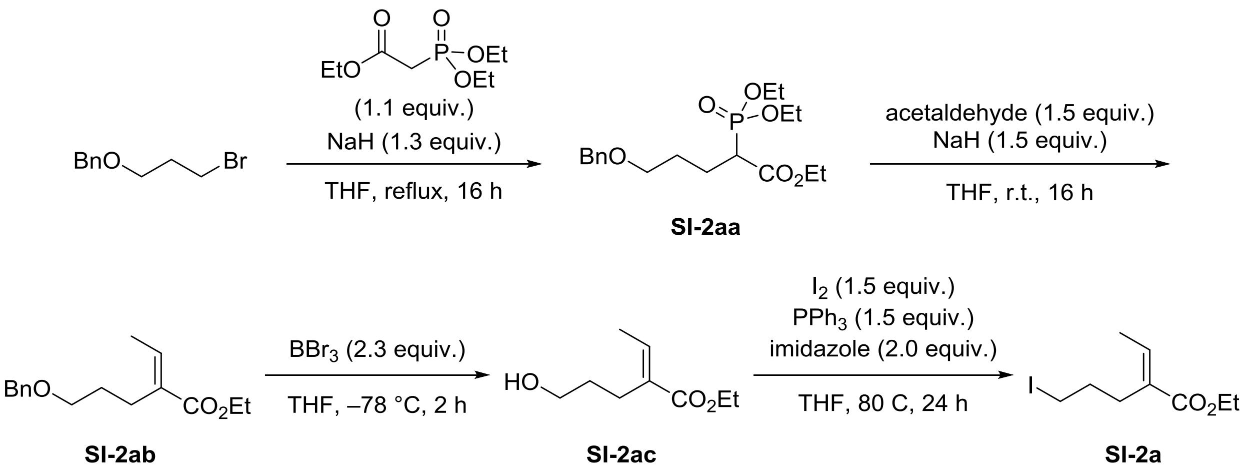

Compound **SI-2aa** was prepared following a modified literature procedure.[18] Triethyl phosphonoacetate (2.24 g, 11.0 mol, 1.10 equiv.) was added dropwise to a suspension of NaH (60% in mineral oil, 520 mg, 13.0 mmol, 1.30 equiv.) in dry THF (40 mL) at r.t. under a N_2 atmosphere. After 1 h, 3-benzyloxy-1-bromopropane (2.29 g, 10.0 mol, 1.00 equiv.) was added and the reaction mixture was heated to reflux and stirred for 16 h. The reaction was allowed to cool to r.t. and quenched with saturated aqueous NH_4Cl (20 mL) and extracted with EtOAc (3 × 30 mL). The combined organic layers were washed with brine, dried ($MgSO_4$), filtered and concentrated *in vacuo*. Purification by flash column chromatography (50% EtOAc/hexane) gave product **SI-2aa** (2.48 g, 6.70 mmol) in 67% yield as a yellow oil.

Compound **SI-2ab** was prepared following a modified literature procedure.[19] To a stirred suspension of NaH (60% in mineral oil, 360 mg, 9.00 mmol, 1.50 equiv.) in dry THF (20 mL) at r.t. was added compound **SI-2aa** (2.23 g, 6.00 mmol, 1.50 equiv.). After 40 min, acetaldehyde (396 mg, 9.00 mmol, 1.50 equiv.) was added, and the reaction mixture was stirred at r.t. for 16 h. The reaction was quenched with saturated aqueous NH_4Cl (20 mL) and extracted with EtOAc (3 × 30 mL). The combined organic layers were washed with brine, dried ($MgSO_4$), filtered and concentrated *in vacuo*. Purification by flash column chromatography (20% EtOAc/hexane) gave alkene **SI-2ab** (0.991 g, 3.78 mmol, 5:1 *E*/*Z*) in 63% yield as a yellow oil.

Compound **SI-2ac** was prepared following a modified literature procedure.[20] To a solution of **SI-2ab** (130 mg, 0.50 mmol, 1.00 equiv.) in DCM (3.0 mL) at –78 °C was added BBr_3 (1 M in DCM, 1.15 mL, 1.15 mmol, 2.30 equiv.). The reaction was stirred at –78 °C for 2 h before being quenched with H_2O (2

mL) and extracted with EtOAc (3 × 30 mL). The combined organic layers were washed with brine, dried ($MgSO_4$), filtered and concentrated *in vacuo*. Purification by flash column chromatography (40% EtOAc/hexane) gave **SI-2ac** (50 mg, 0.30 mmol, 5:1 *E*/*Z*) in 60% yield as a yellow oil.

To a solution of alcohol **SI-2ac** (50 mg, 0.30 mmol, 1.0 equiv.) in THF (5 mL) at r.t. was added I_2 (114 mg, 0.450 mmol, 1.50 equiv.), Ph_3P (118 mg, 0.450 mmol, 1.50 equiv.) and imidazole (41 mg, 0.60 mmol, 2.0 equiv.). The mixture was heating to reflux and stirred for 24 h, before allowing to cool to r.t. and concentrating *in vacuo*. Purification by flash column chromatography (10% EtOAc/hexane) gave iodide **SI-2a** (70 mg, 0.25 mmol, 5:1 *E*/*Z*) in 83% yield as a colourless oil.

TLC: R_f = 0.49 (10% EtOAc/hexane, $KMnO_4$ stain).

^{1}H NMR (400 MHz, $CDCl_3$): δ_H (E isomer) 6.93 – 6.87 (m, 1H), 4.18 (q, *J* = 7.1 Hz, 2H), 3.19 (t, *J* = 6.9 Hz, 2H), 2.42 (t, *J* = 7.4 Hz, 2H), 2.01 – 1.90 (m, 2H), 1.85 (d, *J* = 7.2 Hz, 3H), 1.29 (t, *J* = 7.2 Hz, 3H) ppm.

^{13}C NMR (101 MHz, $CDCl_3$): δ_C (E isomer) 167.5, 138.4, 131.7, 60.4, 32.8, 27.3, 14.5, 14.3, 6.4 ppm.

IR (film) ν_{max}: 2977, 2901, 1704(s), 1648, 1446, 1383, 1263, 1219, 1066, 748 cm^{-1}.

HRMS (ESI^+): calcd. for $C_9H_{15}INaO_2$ $[M+Na]^+$ 305.0009, found 305.0001.

7. Mechanistic Studies

7.1. Cyclic Volammetry

Synthesis and Characterization of Boronate Complex (5)

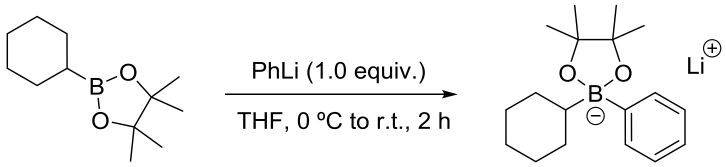

To a 0 °C stirred solution of cyclohexylboronic acid pinacol ester (0.20 g, 0.95 mmol, 1.0 equiv.) in dry THF (5 mL) under an N_2 atmosphere was added dropwise phenyllithium (1.9 M in dibutyl ether, 0.50 mL,0.95 mmol, 1.0 equiv.). The solution was left to warm to r.t. and stirred for 2 h before removing the solvent under vacuum, then dried under vacuum for one hour. Compound **5** was obtained as a white solid (277 mg, 0.94 mmol, 99% yield) which could be stored several weeks in a nitrogen-filled glove box.

^{1}H NMR (400 MHz, DMSO-d_6): δ_H 7.27 – 7.21 (m, 2H), 6.85 (t, J = 7.2 Hz, 2H), 6.73 – 6.67 (m, 1H), 1.55 – 1.42 (m, 5H), 1.01 – 0.80 (m, 4H), 0.94 (s, 6H), 0.66 (s, 6H), 0.60 – 0.47 (m, 2H) ppm.

^{13}C NMR (101 MHz, DMSO-d_6): δ_C 132.4, 124.9, 121.6, 76.8, 30.9, 29.7, 28.4, 28.0, 27.6 ppm. *The carbons attached to boron could not be observed due to quadrupolar relaxation*.

^{11}B NMR (128 MHz, DMSO-d_6): δ_B 6.69 (br. s, 1B) ppm.

IR (film) ν_{max}: 2907, 1446, 1382, 1151, 978, 880, 708 cm^{-1}.

MP: gradual decomposition above 120 °C.

HRMS (ESI$^+$) calcd. for $[M+H+Na]^+$ ($C_{18}H_{29}BNaO_2$) requires m/z 311.2153, found m/z 311.2154.

Cyclic Voltammetry Measurement

Cyclic voltammograms were recorded using an Autolab potentiostat. The sample was prepared using 0.025 mmol of boronate complex **5** in 5 mL of a 0.1 M solution of $N(nBu)_4PF_6$ in dry, degassed MeCN. Measurements used a glassy carbon working electrode, a platinum counter electrode, and a Ag/Ag^+ reference electrode with scan rates of 100 and 250 mV/s. Oxidation potentials were normalised to the ferrocene/ferrocenium redox couple and then converted to saturated calomel electrode (SCE) by adding 0.38 V.

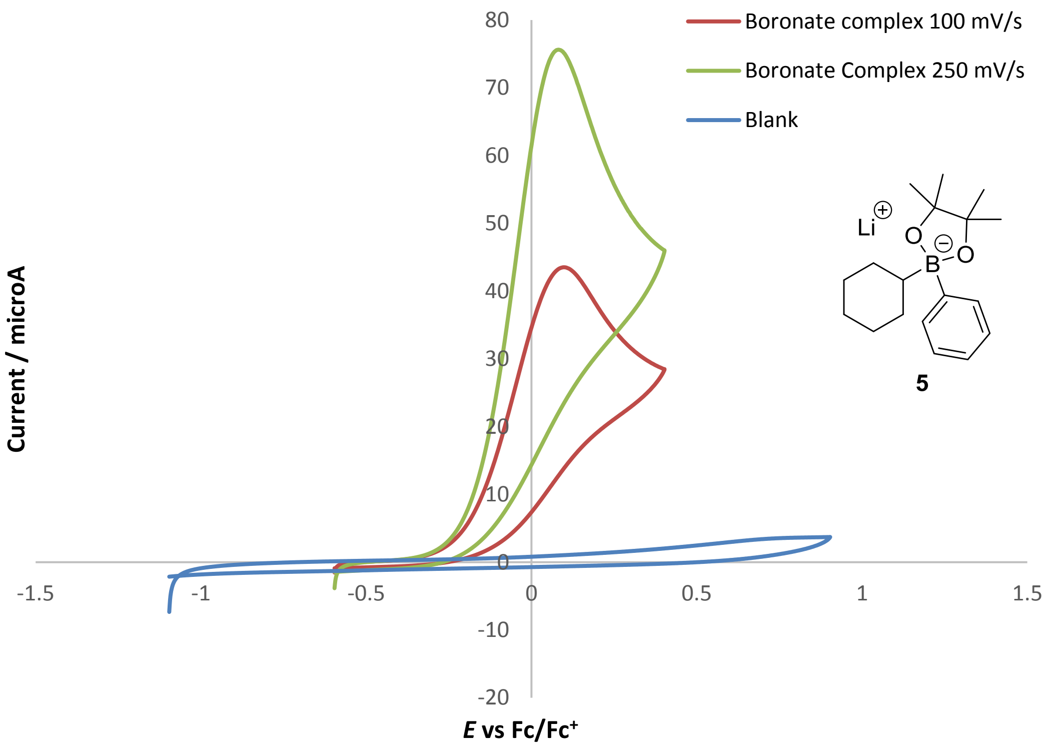

Figure S2. Cyclic voltammograms of boronate complex **5** in MeCN.

The half peak potential ($E_{p/2}$) was determined to be –0.07 vs. Fc/Fc^+ (0.31 vs. SCE).

7.2. Radical Trapping Experiments

***tert*-Butyl 4-((2,2,6,6-tetramethylpiperidin-1-yl)oxy)piperidine-1-carboxylate** (**50**)

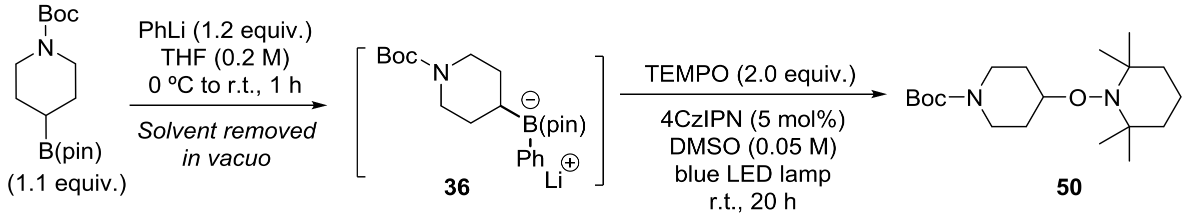

Following **General Procedure A** (except with TEMPO in place of iodide-tethered alkene **7a**) using *N*-Boc-piperidine-4-boronic acid pinacol ester (93 mg, 0.30 mmol, 1.1 equiv.), 4CzIPN (12 mg, 0.015 mmol, 5.0 mol%), PhLi (1.9 M in dibutyl ether, 0.17 mL, 0.33 mmol, 1.20 equiv.), TEMPO (94 mg, 0.60 mmol, 2.0 equiv.) and dry DMSO (6.0 mL), which was irradiated with 1 × Kessil lamp for 20 h. Purification by flash column chromatography (10% EtOAc/hexane) gave the title compound (81.7 mg, 0.24 mmol, 80%) as a colourless oil.

TLC: R_f = 0.45 (10% EtOAc/hexane, $KMnO_4$).

^{1}H NMR (400 MHz, $CDCl_3$): δ_H 4.01 – 3.89 (br. m, 2H), 3.80 – 3.72 (m, 1H), 2.88 – 2.78 (br. m, 2H), 2.02 – 1.92 (m, 2H), 1.57 – 1.23 (m, 8H), 1.45 (s, 9H), 1.11 (br. s, 12H) ppm.

^{13}C NMR (101 MHz, $CDCl_3$): δ_C 154.8, 79.6, 79.3, 59.7, 42.4 (br.), 40.2, 34.5, 31.9, 28.5, 20.5, 17.3 ppm.

IR (film) ν_{max}: 2978, 2933, 2870, 1693(s), 1424, 1364, 1236, 1168, 1045, 737 cm^{-1}.

HRMS (ESI$^+$) calcd. for $C_{19}H_{37}N_2O_3$ $[M+H]^+$ 341.2799, found 341.2804.

Methyl 1-(2-cyclopentylethyl)cyclobutane-1-carboxylate (**52**)

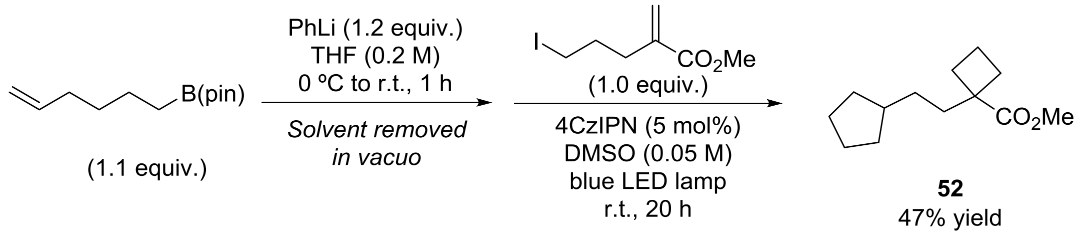

Prepared following **General Procedure B** using 2-(hex-5-en-1-yl)-4,4,5,5-tetramethyl-1,3,2-dioxaborolane (126 mg, 0.660 mmol, 1.10 equiv.), 4CzIPN (24 mg, 0.030 mmol, 5.0 mol%), PhLi (1.9 M in dibutyl ether, 0.38 mL, 0.72 mmol, 1.2 equiv.), methyl 5-iodo-2-methylenepentanoate (**7a**) (152 mg, 0.600 mmol, 1.00 equiv.) and dry DMSO (12.0 mL), which was irradiated with 1 × Kessil lamp for

20 h. Purification by flash column chromatography (5% EtOAc/hexane) gave the title compound (59.3 mg, 0.28 mmol, 47%) as a colourless oil.

TLC: R_f = 0.49 (5% EtOAc/hexane, $KMnO_4$).

^{1}H NMR (400 MHz, $CDCl_3$): δ_H 3.67 (s, 3H), 2.46 – 2.34 (m, 2H), 1.93 – 1.80 (m, 4H), 1.78 – 1.67 (m, 4H), 1.62 – 1.43 (m, 4H), 1.20 – 1.10 (m, 2H), 1.10 – 1.00 (m, 2H), 0.94 – 0.81 (m, 1H) ppm.

^{13}C NMR (101 MHz, $CDCl_3$): δ_C 177.8, 51.6, 47.7, 40.2, 37.3, 32.6, 31.2, 30.0, 25.2, 15.5 ppm.

IR (film) ν_{max}: 2988, 2945, 2869, 1730(s), 1451, 1391, 1201, 986, 844, 750 cm^{-1}.

HRMS (ESI^+) calcd. for $C_{13}H_{23}O_2$ $[M+H]^+$ 211.1693, found 211.1622.

7.3. Anion Trapping Experiments

When the reaction of boronate complex **36** with iodide-tethered alkene **7a** was performed in the presence of H_2O a dramatic solvent effect was observed on the selectivity of the reaction. In MeCN, only Giese product **54** was observed (see entries 1–3, Table in Scheme below). In DMSO, only cyclobutane **20** was observed, even with increased equivalents of H_2O (see entries 4–7, Table in Scheme below). Reducing the reaction time from 20 h to 1 h resulted in a mixture of **20** and **54** (see entry 8, Table in Scheme below), which suggested the **54** was unstable under the wet reaction conditions.

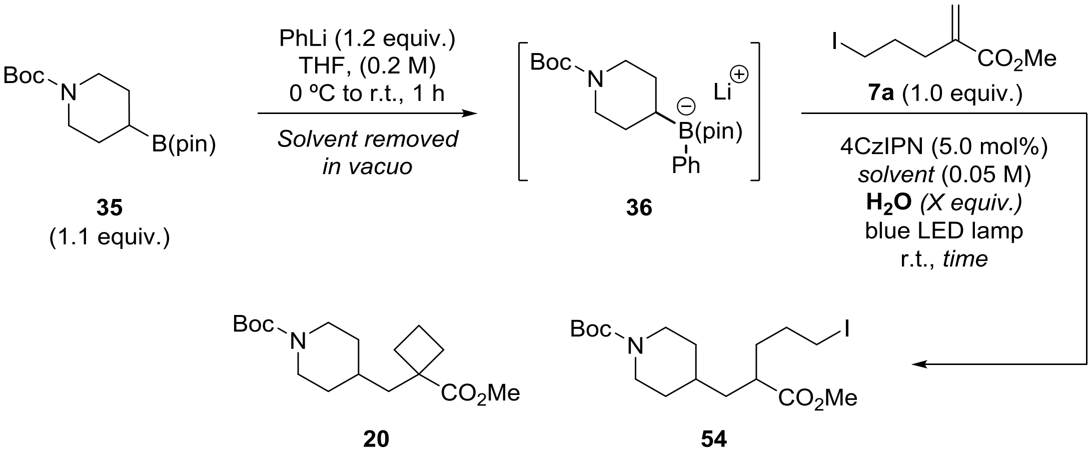

entry	solvent	time (h)	H_2O equiv	% 20	% 53	% 7a
1	MeCN	20	0	71	0	0
2	MeCN	20	5	0	60	7
3	MeCN	20	10	0	45	19
4	DMSO	20	0	76	0	0
5	DMSO	20	5	40	0	0
6	DMSO	20	10	42	0	0
7	DMSO	20	20	36	0	0
8	DMSO	1	5	34	20	3

Yields were determined by ^{1}H NMR using diethyl phthalate as internal standard.

The stability of Giese product **54** under the reaction conditions in wet DMSO was further investigated by treating **54** with LiOH (the by-product formed upon generation of **54**) at r.t. for 20 h. This resulted in complete decomposition, with no identifiable products.

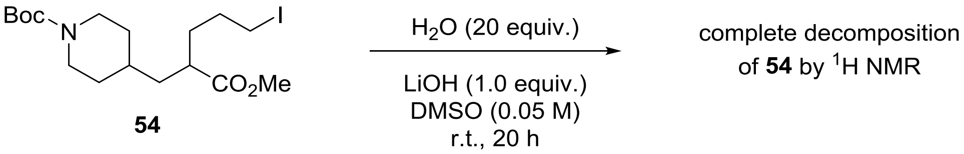

Similar observations were made upon reacting cyclohexylboronic ester **6** with halide-tethered alkenes **7a** (iodide) and **7b** (bromide). When using wet MeCN as the solvent, selective formation of the Giese products **9a** and **9b** was observed.

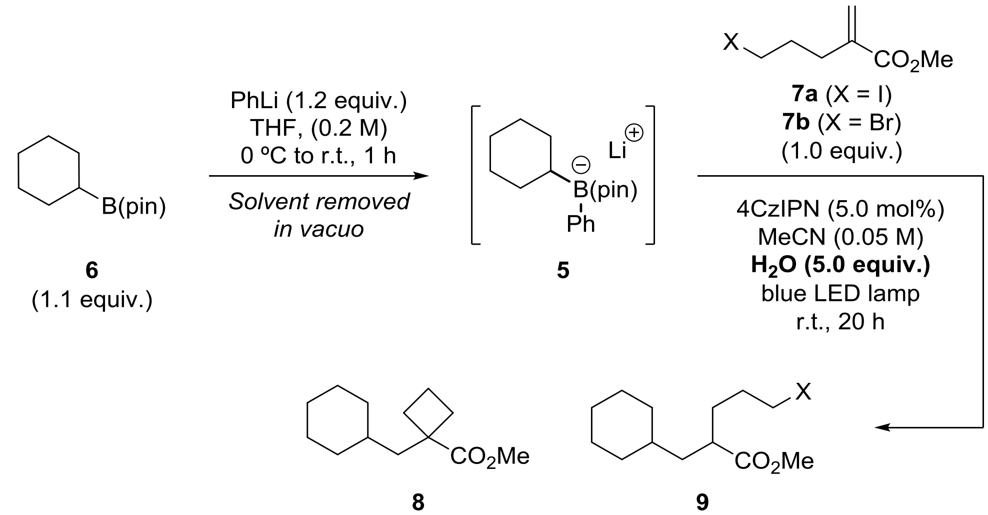

	8	9
with **7a** (X = I)	0%	53%
with **7b** (X = Br)	0%	61%

Yields were determined by ^{1}H NMR using diethyl phthalate as internal standard.

7.4. Characterization of Giese Products

***tert*-Butyl 4-(5-iodo-2-(methoxycarbonyl)pentyl)piperidine-1-carboxylate** (**54**)

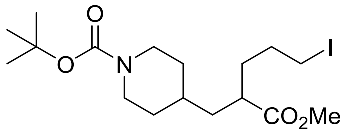

To a stirred solution of *N*-Boc-piperidine-4-boronic acid pinacol ester (137 mg, 0.440 mmol, 1.10 equiv.) in THF (1.75 mL) under N_2 at 0 °C was added phenyllithium (1.9 M in dibutyl ether, 0.25 mL, 0.48 mmol, 1.2 equiv.) dropwise. The solution was then stirred for 1 h at 0 °C, warmed to r.t. and stirred for a further 10 min before removing the solvent under vacuum. Degassed dry MeCN (5 mL) was added to the system. The reaction was irradiated with a 40 W Kessil LED lamp with fan cooling. A degassed solution of the halide-tethered alkene **7a** (101 mg, 0.400 mmol, 1.00 equiv.), 4CzIPN (16 mg, 0.020 mmol, 5.0 mol%) and H_2O (36 mg, 2.0 mmol, 5.0 equiv.) in MeCN (3.0 mL) was added under irradiation. The N_2 inlet was removed and the flask further sealed with parafilm. The reaction mixture was stirred vigorously for 20 h under constant irradiation. The reaction mixture was diluted with DCM (60 mL) and the solution washed with saturated aqueous NH_4Cl (30 mL), water (30 mL) and brine (30 mL). The resulting organic phase was dried ($MgSO_4$), filtered and concentrated *in vacuo*. The yield was determined by ^{1}H NMR using diethyl phthalate as an internal standard to be 60%. Purification by flash column chromatography (20% EtOAc/hexane) gave the title compound (96.6 mg, 0.22 mmol, 55%) as a colourless oil.

TLC: R_f = 0.32 (20% EtOAc/hexane, $KMnO_4$ stain).

^{1}H NMR (400 MHz, $CDCl_3$): δ_H 4.07 (br. s, 2H), 3.69 (s, 3H), 3.17 (t, *J* = 6.7 Hz, 2H), 2.65 (br. t, *J* = 13.6 Hz, 2H), 2.54 – 2.45 (m, 1H), 1.83 – 1.75 (m, 2H), 1.74 – 1.64 (m, 2H), 1.64 – 1.53 (m, 3H), 1.45 (s, 9H), 1.40 – 1.27 (m, 2H), 1.15 – 0.99 (m, 2H) ppm.

^{13}C NMR (101 MHz, $CDCl_3$): δ_C 176.2, 154.8, 79.3, 51.6, 43.9 (br.), 41.8, 39.2, 34.08, 33.6, 32.4, 31.7, 31.0, 28.5, 5.9 ppm.

IR (film) ν_{max}: 2930, 2860, 1734(s), 1690(s), 1425, 1335, 1162, 971, 732 cm^{-1}.

HRMS (ESI$^+$) calcd. for $C_{17}H_{30}INNaO_4$ $[M+Na]^+$ 462.1112, found 462.1116.

Methyl 2-(cyclohexylmethyl)-5-iodopentanoate (9a)

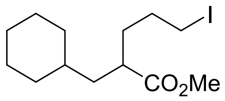

Prepared following the same procedure as used for the synthesis of Giese product **53**, using cyclohexylboronic acid pinacol ester (46 mg, 0.22 mmol, 1.1 equiv.), phenyllithium (1.9 M in dibutyl ether, 0.13 mL, 0.24 mmol, 1.2 equiv.), halide-tethered alkene **7a** (51 mg, 0.20 mmol, 1.0 equiv.), 4CzIPN (7.9 mg, 0.010 mmol, 5.0 mol%) and H_2O (18 mg, 1.0 mmol, 5.0 equiv.) in MeCN (4.0 mL), which was irradiated with 1 × 40 W Kessil LED lamp for 20 h. The yield was determined by ^{1}H NMR using diethyl phthalate as an internal standard to be 53%. Purification by flash column chromatography (5% EtOAc/hexane) gave the title compound (33.8 mg, 0.10 mmol, 50%) as a colourless oil.

TLC: R_f = 0.49 (5% EtOAc/hexane, $KMnO_4$ stain).

^{1}H NMR (400 MHz, $CDCl_3$): δ_H 3.67 (s, 3H), 3.15 (t, J = 6.9 Hz, 2H), 2.53 – 2.43 (m, 1H), 1.83 – 1.51 (m, 10H), 1.31 – 1.05 (m, 5H), 0.93 – 0.76 (m, 2H) ppm.

^{13}C NMR (101 MHz, $CDCl_3$): δ_C 176.6, 51.5, 41.9, 40.1, 35.6, 33.6, 33.5, 32.9, 31.2, 26.5, 26.2, 6.1 ppm.

IR (film) ν_{max}: 2922, 2850, 1734(s), 1448, 1193, 1164, 1043, 971 cm^{-1}.

HRMS (ESI$^+$) calcd. for $C_{13}H_{23}INaO_2$ $[M+Na]^+$ 361.0635, found 361.0620.

Methyl 5-bromo-2-(cyclohexylmethyl)pentanoate (9b)

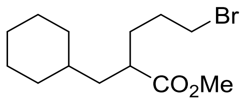

Prepared following the same procedure as used for the synthesis of Giese product **53**, using cyclohexylboronic acid pinacol ester (46 mg, 0.22 mmol, 1.1 equiv.), phenyllithium (1.9 M in dibutyl ether, 0.13 mL, 0.24 mmol, 1.2 equiv.), halide-tethered alkene **7b** (41 mg, 0.20 mmol, 1.0 equiv.), 4CzIPN (7.9 mg, 0.010 mmol, 5.0 mol%) and H_2O (18 mg, 1.0 mmol, 5.0 equiv.) in MeCN (4.0 mL), which was irradiated with 1 × 40 W Kessil LED lamp for 20 h. The yield was determined by ^{1}H NMR using diethyl phthalate as an internal standard to be 61%. Purification by flash column chromatography (5% EtOAc/hexane) gave the title compound (33.8 mg, 0.12 mmol, 58%) as a colourless oil.

TLC: R_f = 0.49 (5% EtOAc/hexane, $KMnO_4$ stain).

^{1}H NMR (400 MHz, $CDCl_3$): δ_H 3.67 (s, 3H), 3.38 (t, *J* = 6.7 Hz, 2H), 2.54 – 2.44 (m, 1H), 1.88 – 1.51 (m, 10H), 1.32 – 1.06 (m, 5H), 0.94 – 0.77 (m, 2H) ppm.

^{13}C NMR (101 MHz, $CDCl_3$): δ_C 176.6, 51.5, 42.1, 40.2, 35.6, 33.5, 33.2, 32.9, 31.3, 30.5, 26.5, 26.2 ppm.

IR (film) ν_{max}: 2922, 2851, 1735(s), 1448, 1194, 1165, 1043, 970 cm^{-1}.

HRMS (ESI^+) calcd. for $C_{13}H_{23}BrNaO_2$ $[M+Na]^+$ 313.0774, found 313.0769.

Methyl 5-chloro-2-(cyclohexylmethyl)pentanoate (9c)

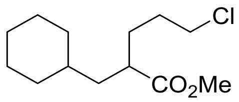

Prepared following **General Procedure A** but with MeCN as the solvent: Using cyclohexylboronic acid pinacol ester (46 mg, 0.22 mmol, 1.1 equiv.), phenyllithium (1.9 M in dibutyl ether, 0.13 mL, 0.24 mmol, 1.2 equiv.), halide-tethered alkene **7c** (33 mg, 0.20 mmol, 1.0 equiv.), 4CzIPN (7.9 mg, 0.010 mmol, 5.0 mol%) in MeCN (4.0 mL), which was irradiated with 1 × 40 W Kessil LED lamp for 20 h. The yield was determined by ^{1}H NMR using diethyl phthalate as an internal standard to be 50%. Purification by flash column chromatography (5% EtOAc/hexane) gave the title compound (23.7 mg, 0.10 mmol, 48%) as a colourless oil.

TLC: R_f = 0.49 (5% EtOAc/hexane, $KMnO_4$ stain).

^{1}H NMR (400 MHz, $CDCl_3$): δ_H 3.68 (s, 3H), 3.52 (t, *J* = 5.9 Hz, 2H), 2.54 – 2.44 (m, 1H), 1.79 – 1.57 (m, 10H), 1.33 – 1.09 (m, 5H), 0.94 – 0.78 (m, 2H) ppm.

^{13}C NMR (101 MHz, $CDCl_3$): δ_C 176.7, 51.5, 44.7, 42.2, 40.2, 35.6, 33.5, 33.0, 30.4, 30.1, 26.5, 26.2, 24.9 ppm.

IR (film) ν_{max}: 2923, 2852, 1735(s), 1448, 1360, 1194, 1164, 1043, 971 cm^{-1}.

HRMS (ESI^+) calcd. for $C_{13}H_{23}ClNaO_2$ $[M+Na]^+$ 269.1279, found 269.1289.

Methyl 2-(cyclohexylmethyl)-5-(tosyloxy)pentanoate (9d)

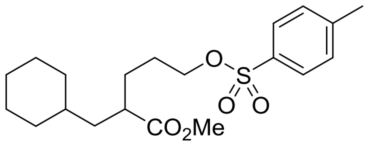

Prepared following **General Procedure A** but with MeCN as the solvent: Using cyclohexylboronic acid pinacol ester (46 mg, 0.22 mmol, 1.1 equiv.), phenyllithium (1.9 M in dibutyl ether, 0.13 mL, 0.24 mmol, 1.2 equiv.), tosylate-tethered alkene **7d** (60 mg, 0.20 mmol, 1.0 equiv.), 4CzIPN (7.9 mg, 0.010 mmol, 5.0 mol%) in MeCN (4.0 mL), which was irradiated with 1 × 40 W Kessil LED lamp for 20 h. The yield was determined by ^{1}H NMR using diethyl phthalate as an internal standard to be 55%. Purification by flash column chromatography (20 % EtOAc/hexane) gave the title compound (38.2 mg, 0.10 mmol, 50%) as a colourless oil.

TLC: R_f = 0.40 (20% EtOAc/hexane, $KMnO_4$ stain).

^{1}H NMR (400 MHz, $CDCl_3$): δ_H 7.78 (d, *J* = 8.3 Hz, 2H), 7.34 (d, *J* = 8.1 Hz, 2H), 4.04 – 3.96 (m, 2H), 3.64 (s, 3H), 2.45 (s, 3H), 2.43 – 2.36 (m, 1H), 1.75 – 1.44 (m, 10H), 1.27 – 1.09 (m, 5H), 0.92 – 0.74 (m, 2H) ppm.

^{13}C NMR (101 MHz, $CDCl_3$): δ_C 176.5, 144.7, 133.1, 129.8, 127.9, 70.1, 51.5, 42.2, 40.1, 35.6, 33.5, 32.9, 28.6, 26.7, 26.5, 26.2, 24.9, 21.6 ppm.

IR (film) ν_{max}: 2923, 2850, 1733(s), 1560, 1450, 1358, 1188, 1175, 1091, 814 cm^{-1}.

HRMS (ESI$^+$) calcd. for $C_{20}H_{30}NaO_5S$ $[M+Na]^+$ 405.1712, found 405.1692.

8. References

[1] Luo, J.; Zhang, J. *ACS Catal.* **2016**, *6*, 873.

[2] a) For boronic ester **12a**, see: Aichhorn, S.; Bigler, R.; Myers, E. L.; Aggarwal, V. K. *J. Am. Chem. Soc.* **2017**, *139*, 9519; b) For boronic esters **18a**, **25a**, **29a**, and **34a**, see: Fawcett, A.; Pradeilles, J.; Wang, Y.; Mutsuga, T.; Myers, E. L.; Aggarwal, V. K. *Science* **2017**, *357*, 283; c) For boronic esters **21a**, **22a**, see: Varela, A.; Garve, L. K. B.; Leonori, D.; Aggarwal, V. K. *Angew. Chem., Int. Ed.* **2017**, *56*, 2127; d) For boronic ester **30a**, see: Odachowski, M.; Bonet, A.; Essafi, S.; Conti-Ramsden, P.; Harvey, J. N.; Leonori, D.; Aggarwal, V. K. *J. Am. Chem. Soc.* **2016**, *138*, 9521; e) For boronic ester **26a**, see: Larouche-Gauthier, R.; Elford, T. G.; Aggarwal, V. K. *J. Am. Chem. Soc.* **2011**, *133*, 16794; f) For boronic esters **31a**, **33a**, see: Bonet, A.; Odachowski, M.; Leonori, D.; Essafi, S.; Aggarwal, V. K. *Nat. Chem.* **2014**, *6*, 584.

[3] Adachi, S.; Cognetta 3rd, A. B.; Niphakis, M. J.; He, Z.; Zajdlik, A.; Denis, J. D. St.; Scully, C. C. G.; Cravatt, B. F.; Yudin, A. K. *Chem. Commun.* **2015**, *51*, 3608.

[4] a) Thorpe, S. B.; Calderone, J. A.; Santos, W. L. *Org. Lett.* **2012**, *14*, 1918; b) Hirano, K.; Yorimitsu, H.; Oshima, K. *Org. Lett*. **2007**, *9*, 5031.

[5] Ito, H.; Kubota, K. *Org. Lett*. **2012**, *14*, 890.

[6] Yi, J.; Liu, J.-H.; Liang, J.; Dai, J.-J.; Yang, C.-T.; Fu, Y.; Liu, L. *Adv. Synth. Catal.* **2012**, *354*, 1685.

[7] Kim, J. H.; Chung, Y. K. *RSC Adv.* **2014**, *4*, 39755.

[8] a) Atack, T. C.; Cook, S. P. *J. Am. Chem. Soc.* **2016**, *138*, 6139; b) Murata, M.; Kawakita, K.; Asana, T.; Watanabe, S.; Masuda, Y. *Bull. Chem. Soc. Jpn.* **2002**, *75*, 825.

[9] Shu, C.; Mega, R. S.; Andreassen, B. J.; Noble, A.; Aggarwal, V. K. *Angew. Chem. Int. Ed.* **2018**, *57*, 15430.

[10] Du, J.; and Yoon, T. P. *J. Am. Chem. Soc.* **2009**, *131*, 14604.

[11] a) Brooks, J. L.; Frontier, A. J. *J. Am. Chem. Soc.* **2012**, *134*, 16551; b) Pradal, A.; Evano, G. *Chem. Commun*. **2014**, *50*, 11907.

[12] Wang, H.; Lu, Q.; Chiang, C.-W.; Luo, Y.; Zhou, J.; Wang, G.; Lei, A. *Angew. Chem. Int. Ed.* **2017**, *56*, 595.

[13] Takagi, J.; Takahashi, K.; Ishiyama, T.; Miyaura, N. *J. Am. Chem. Soc.* **2002**, *124*, 8001.

[14] Hu, C.; Chen, Y. *Org. Chem. Front.* **2015**, *2*, 1352.

[15] Ohkita, T.; Tsuchiya, Y.; Togo, H. *Tetrahedron* **2008**, *64*, 7247.

[16] Chen, Y. G.; Shuai, B.; Ma, C.; Zhang, X. J.; Fang, P.; Mei, T. S. *Org. Lett.* **2017**, *19*, 2969.

[17] Heckenbichler, K.; Schweiger, A.; Brandner, L. A.; Binter, A.; Toplak, M.; Macheroux, P.; Gruber, K.; Breinbauer, R. *Angew. Chem. Int. Ed.* **2018**, *57*, 7240.

[18] Saya, J. M.; Vos, K.; Kleinnijenhuis, R. A.; van Maarseveen, J. H.; Ingemann, S.; Hiemstra, H. *Org. Lett.* **2015**, *17*, 3892.

[19] Bargiggia, F.; Piva, O. *Tetrahedron: Asymmetry* **2003**, *14*, 1819.

[20] Lutteke, G.; Kleinnijenhuis, R. A.; Beuving, R. J.; Gelder, D.; Smits, M. M. M.; Maarseveen, J. H.; Hiemstra, H. *Eur. J. Org. Chem.* **2016**, 5845.

9. NMR Spectra

^{1}H NMR (400 MHz, $CDCl_3$)

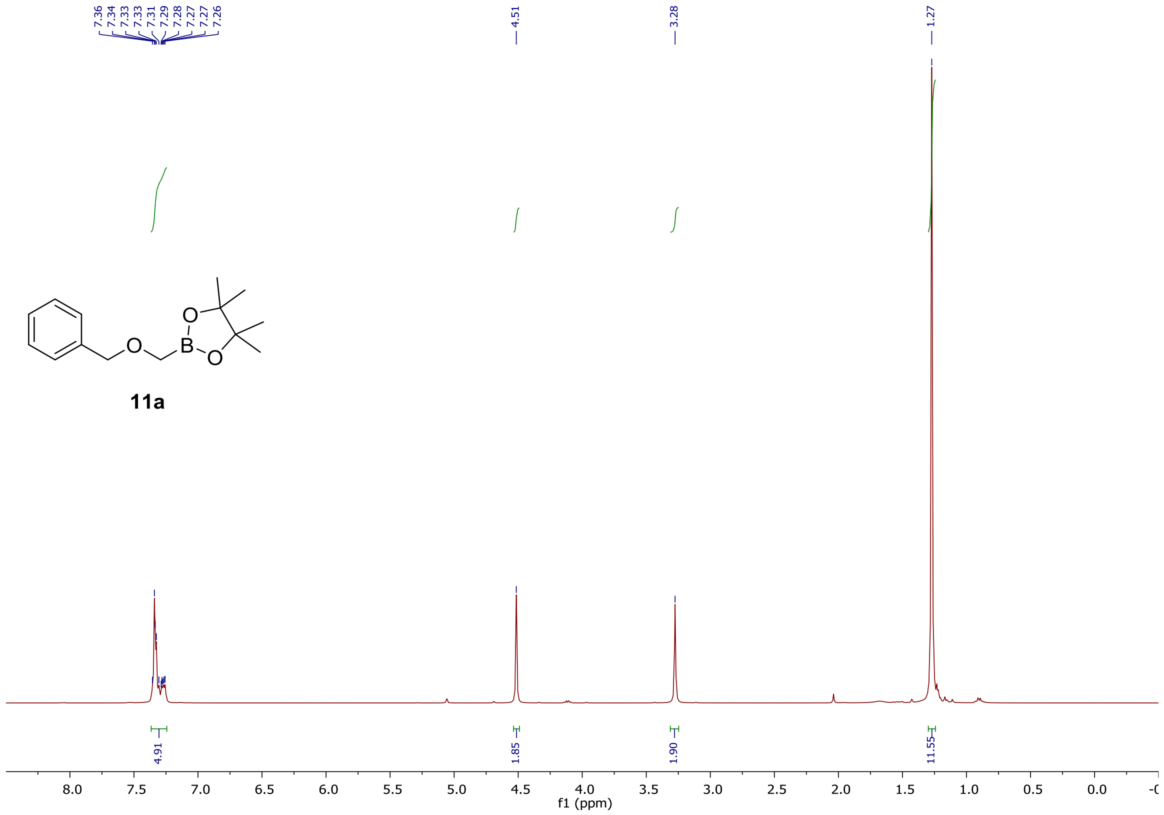

^{13}C NMR (101 MHz, $CDCl_3$)

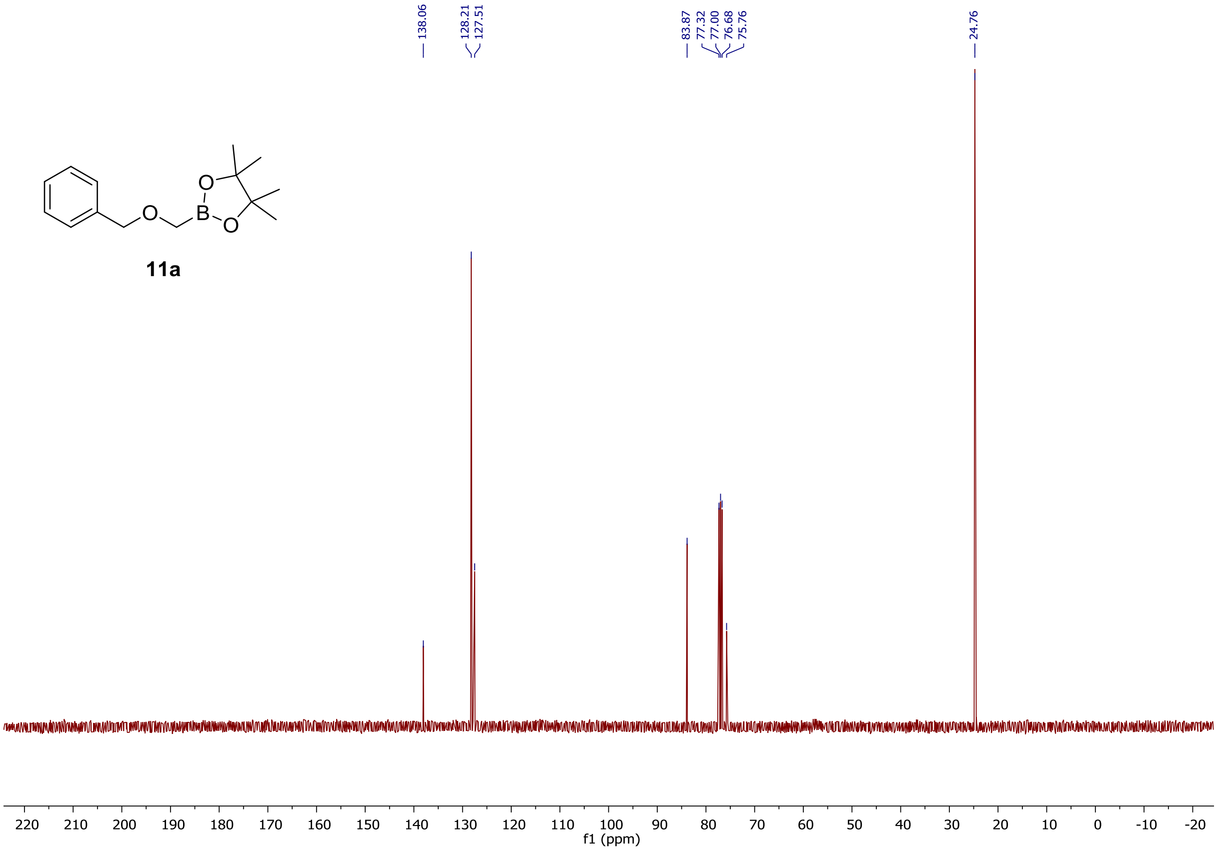

^{1}H NMR (400 MHz, $CDCl_3$)

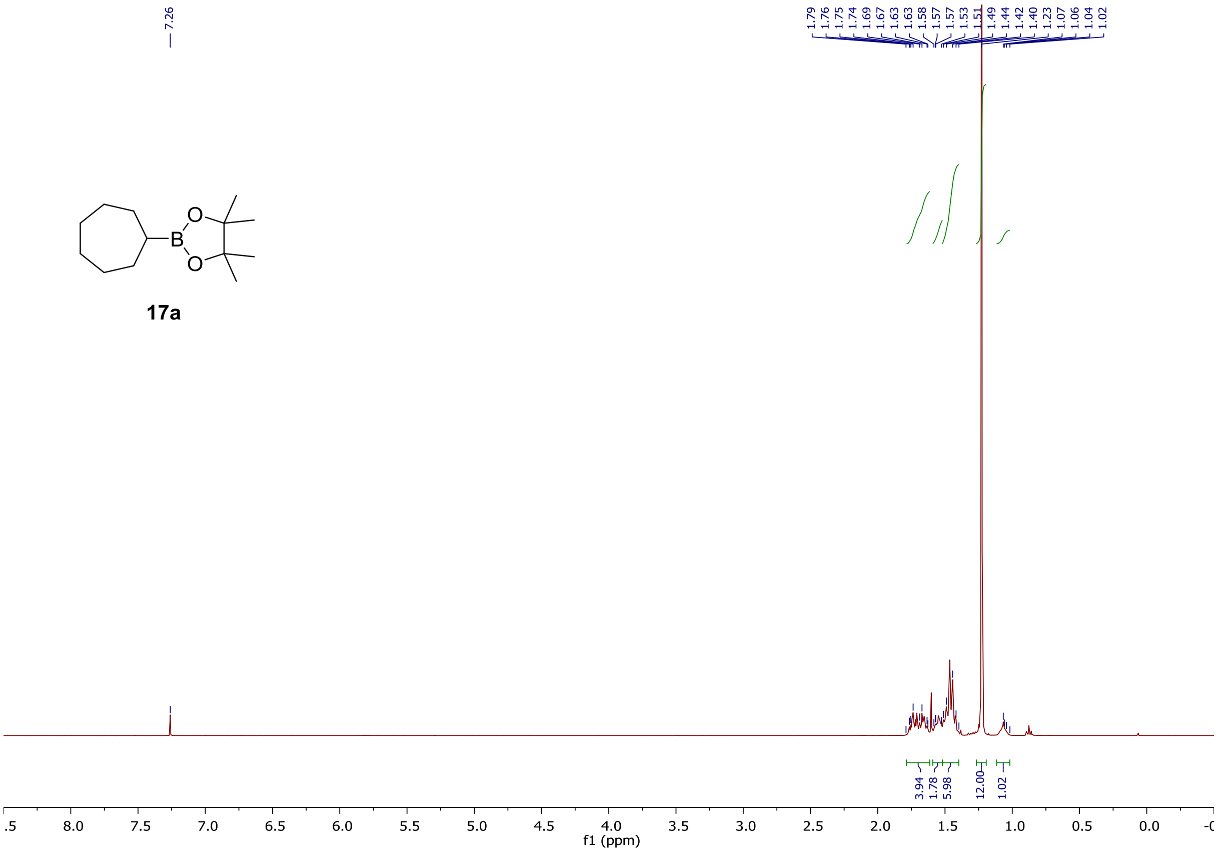

^{13}C NMR (101 MHz, $CDCl_3$)

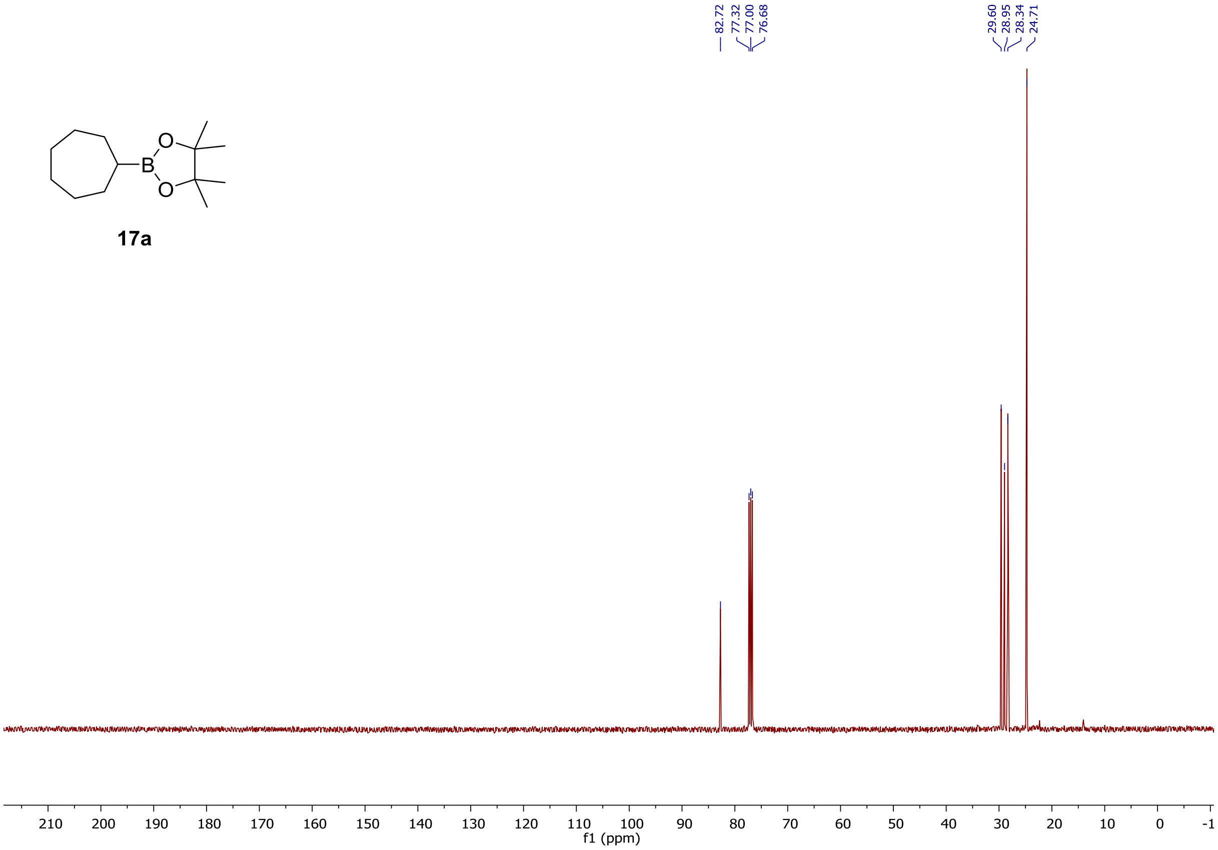

^{1}H NMR (400 MHz, $CDCl_3$)

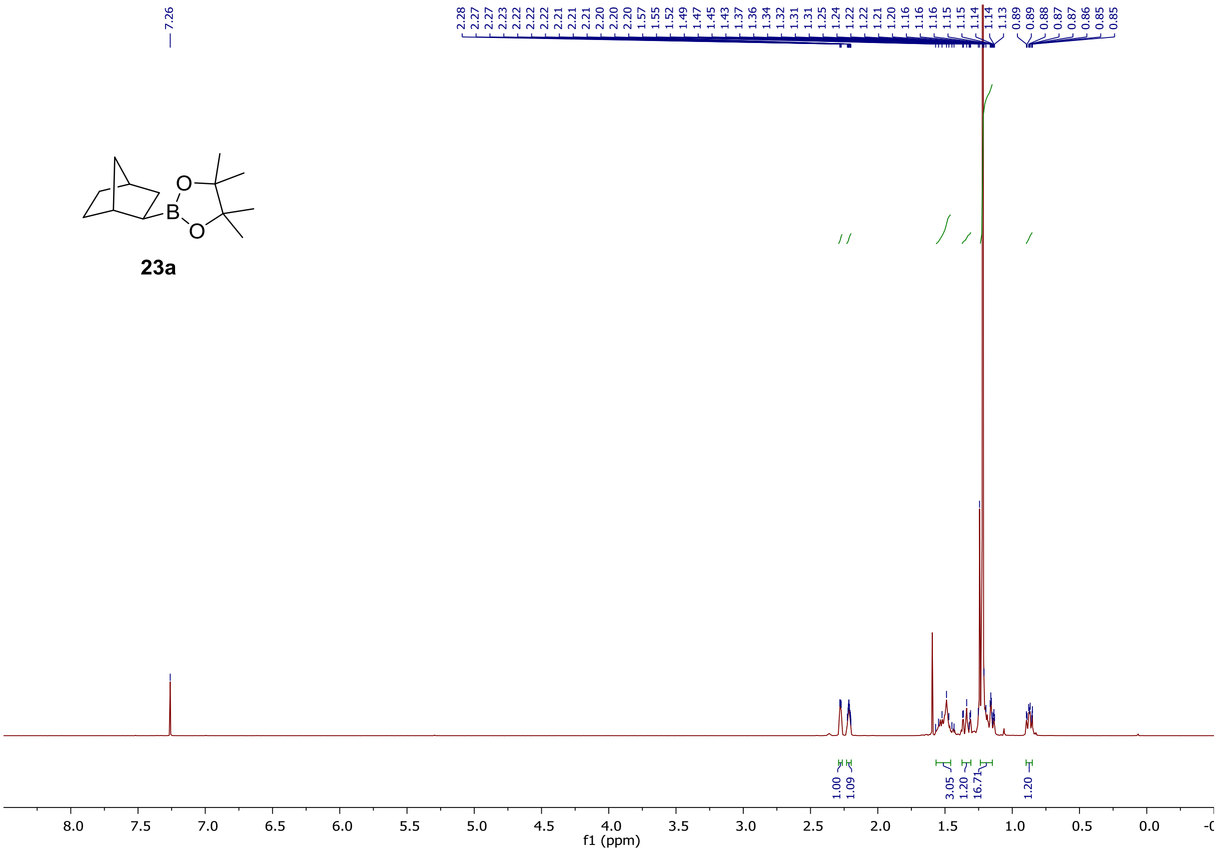

^{13}C NMR (101 MHz, $CDCl_3$)

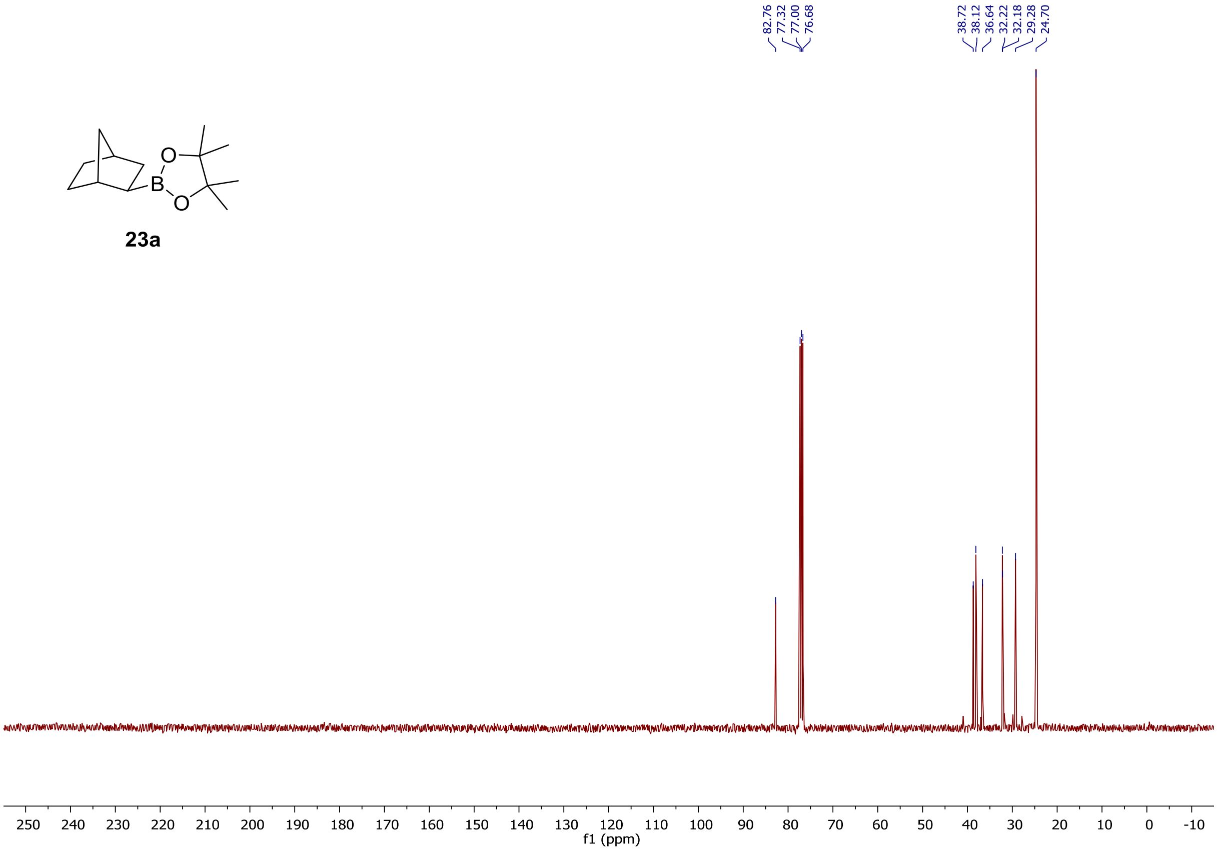

^{1}H NMR (400 MHz, $CDCl_3$)

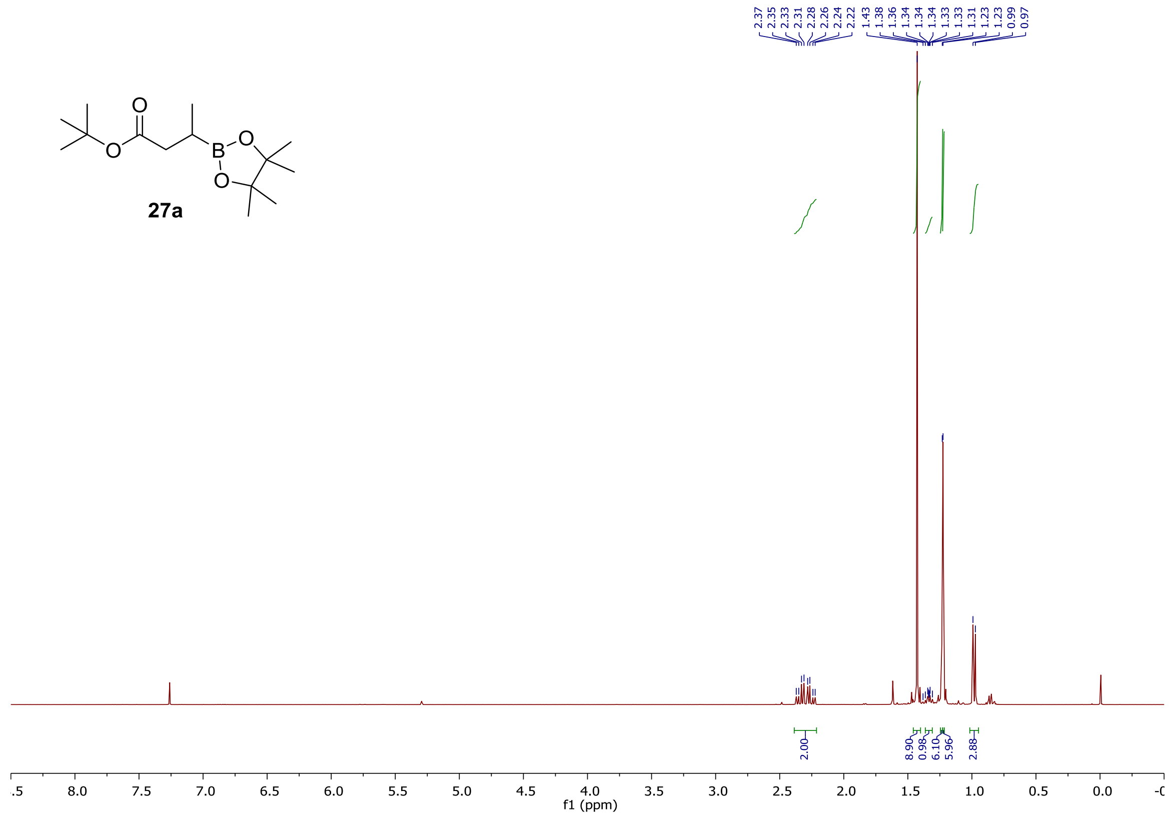

^{13}C NMR (101 MHz, $CDCl_3$)

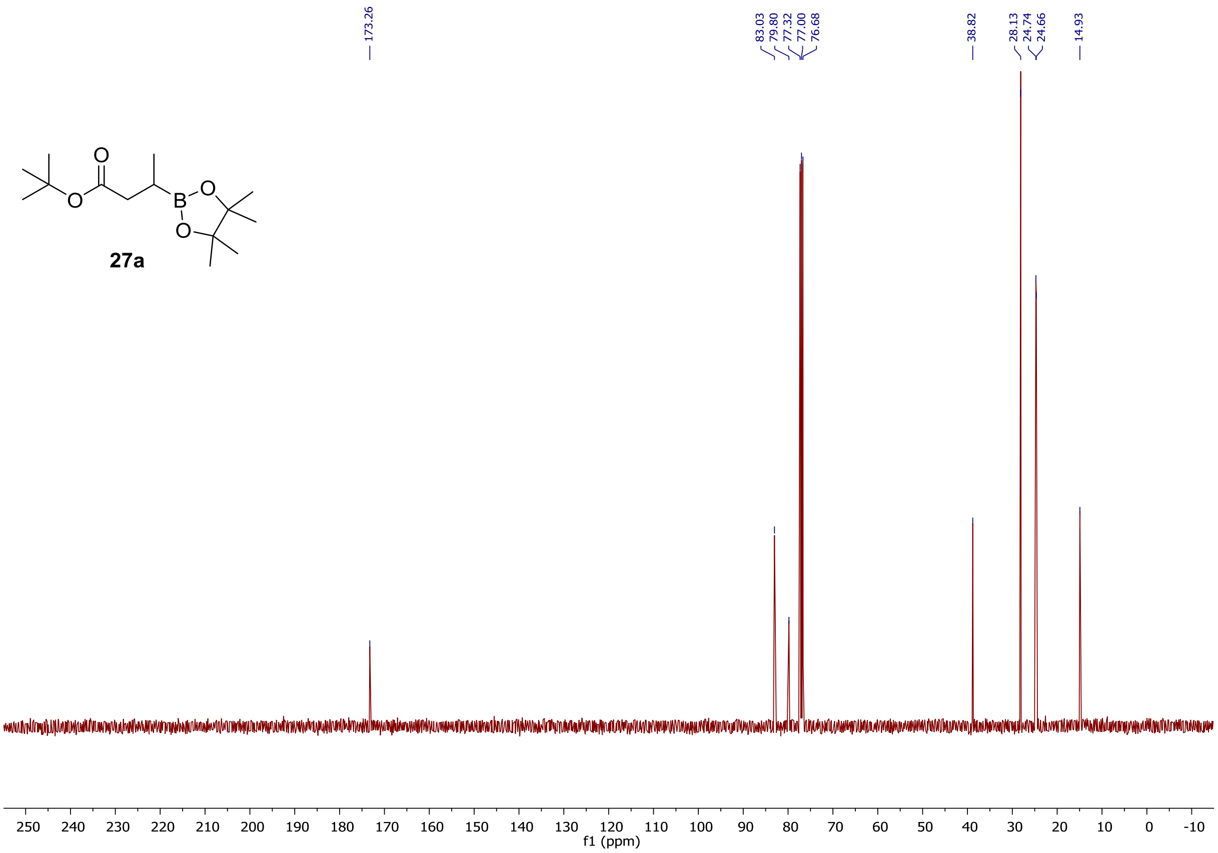

^{1}H NMR (400 MHz, $CDCl_3$)

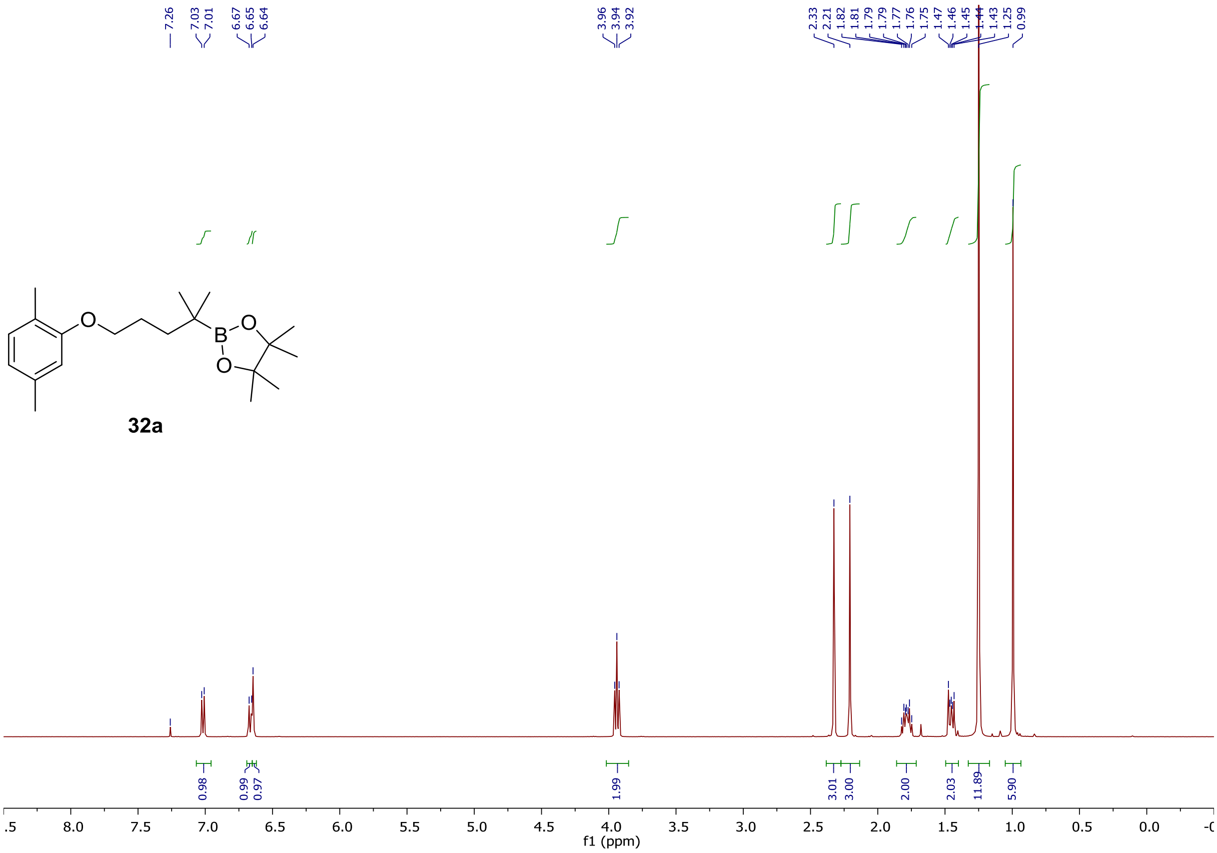

^{13}C NMR (101 MHz, $CDCl_3$)

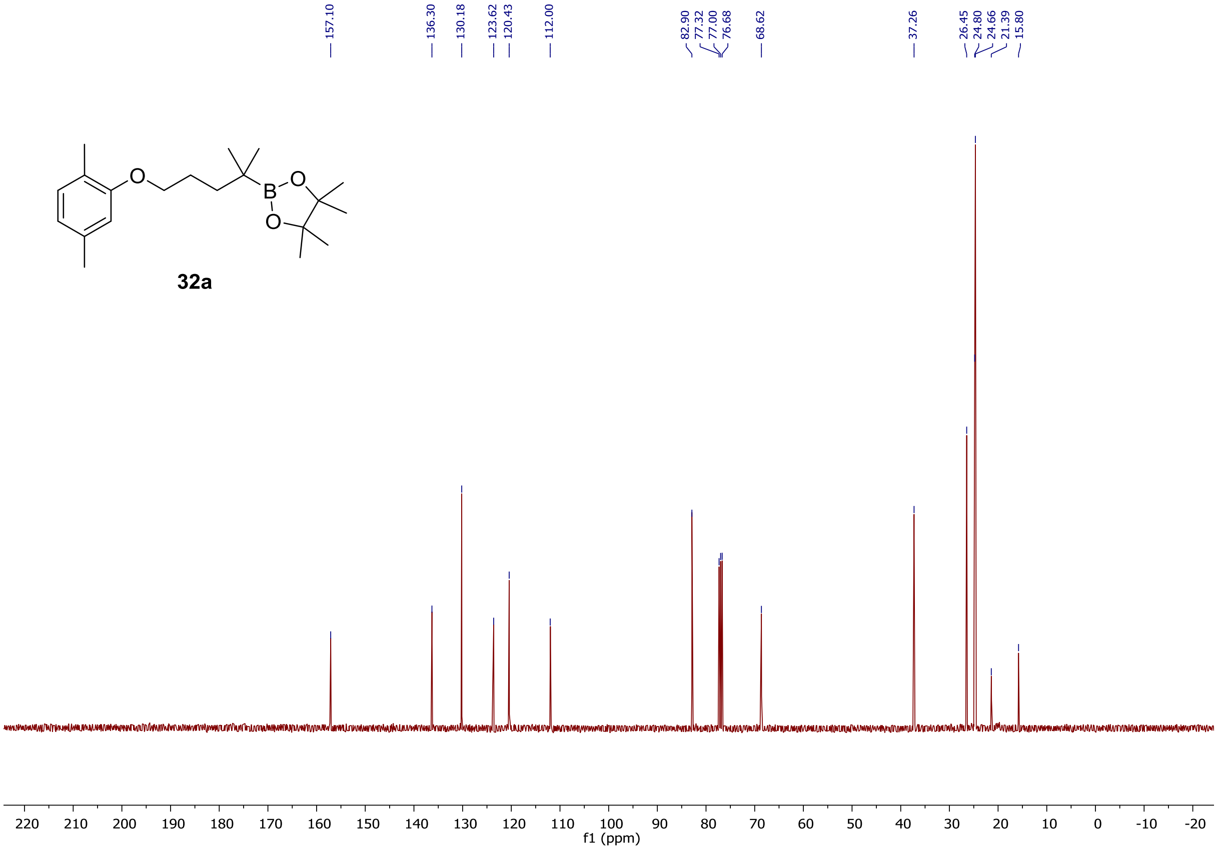

^{1}H NMR (400 MHz, $CDCl_3$)

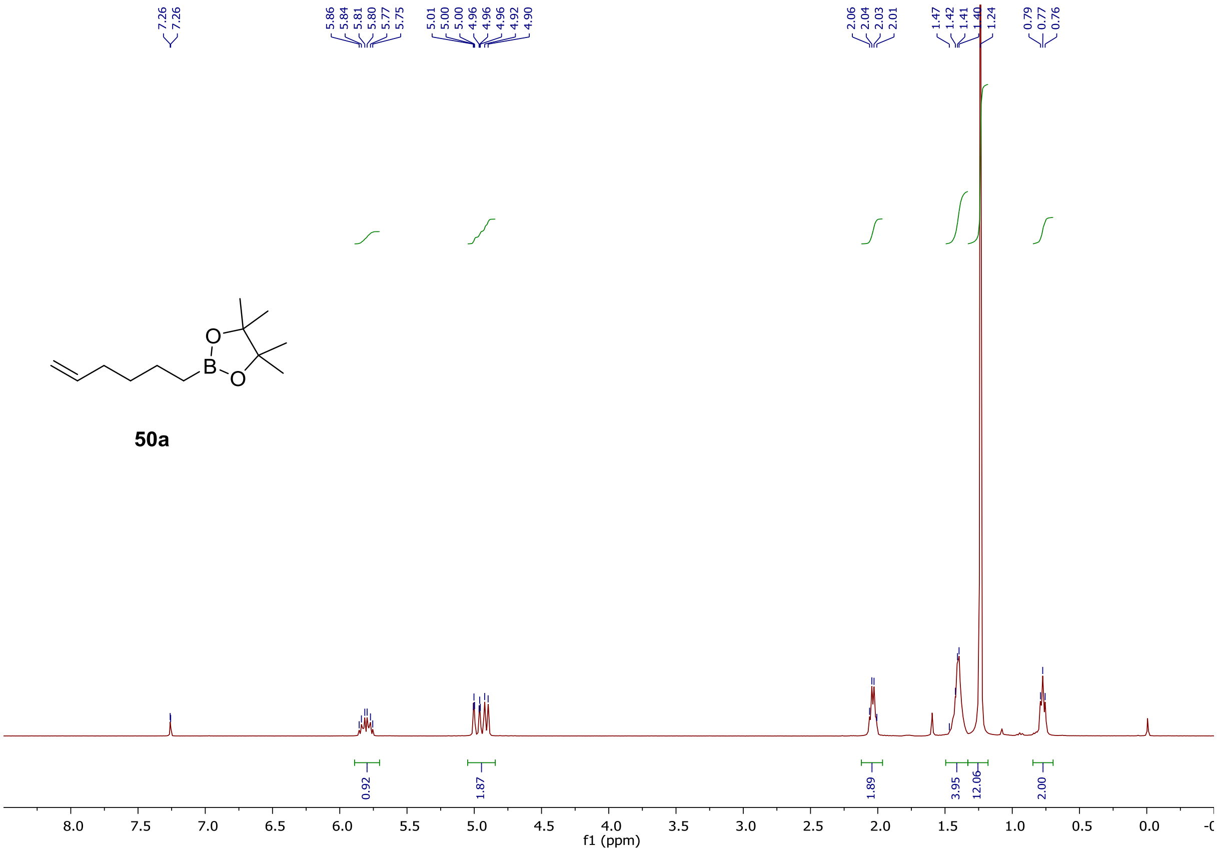

^{13}C NMR (101 MHz, $CDCl_3$)

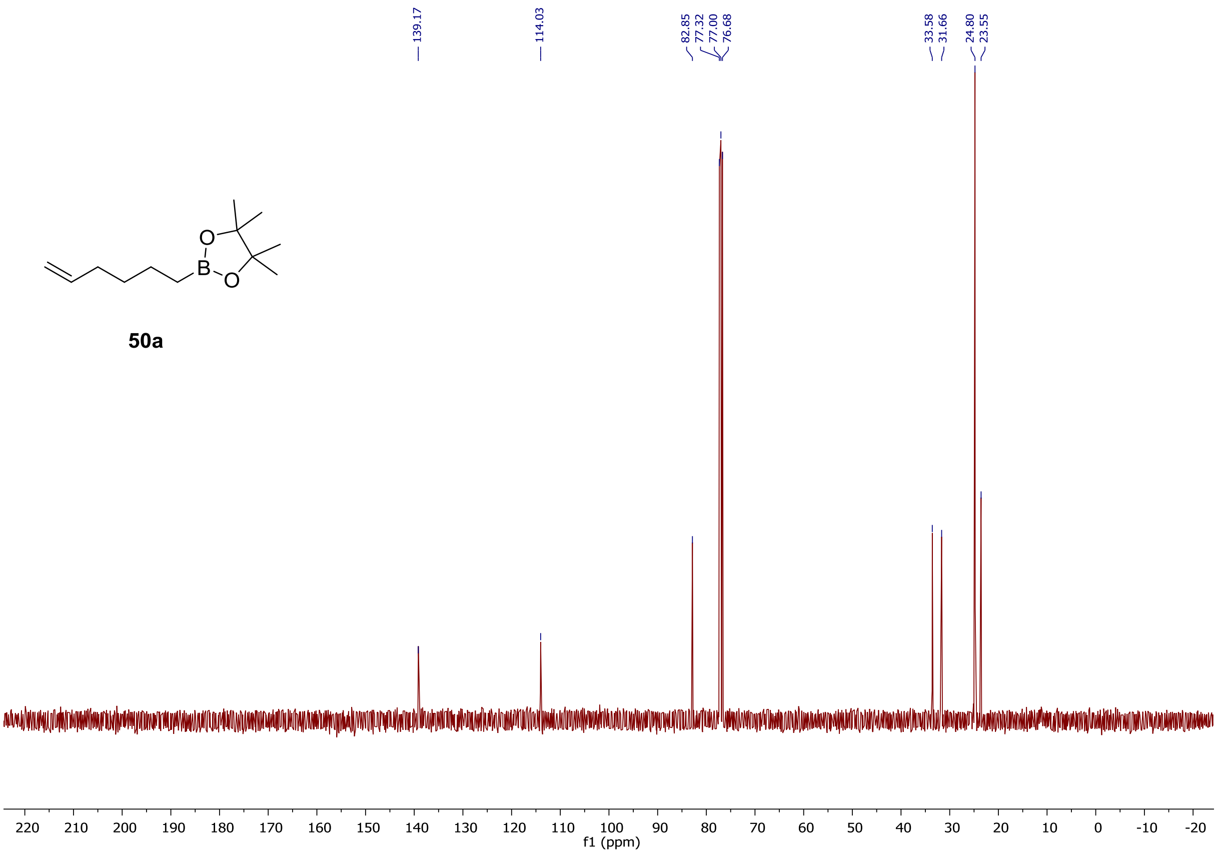

^{1}H NMR (400 MHz, $CDCl_3$)

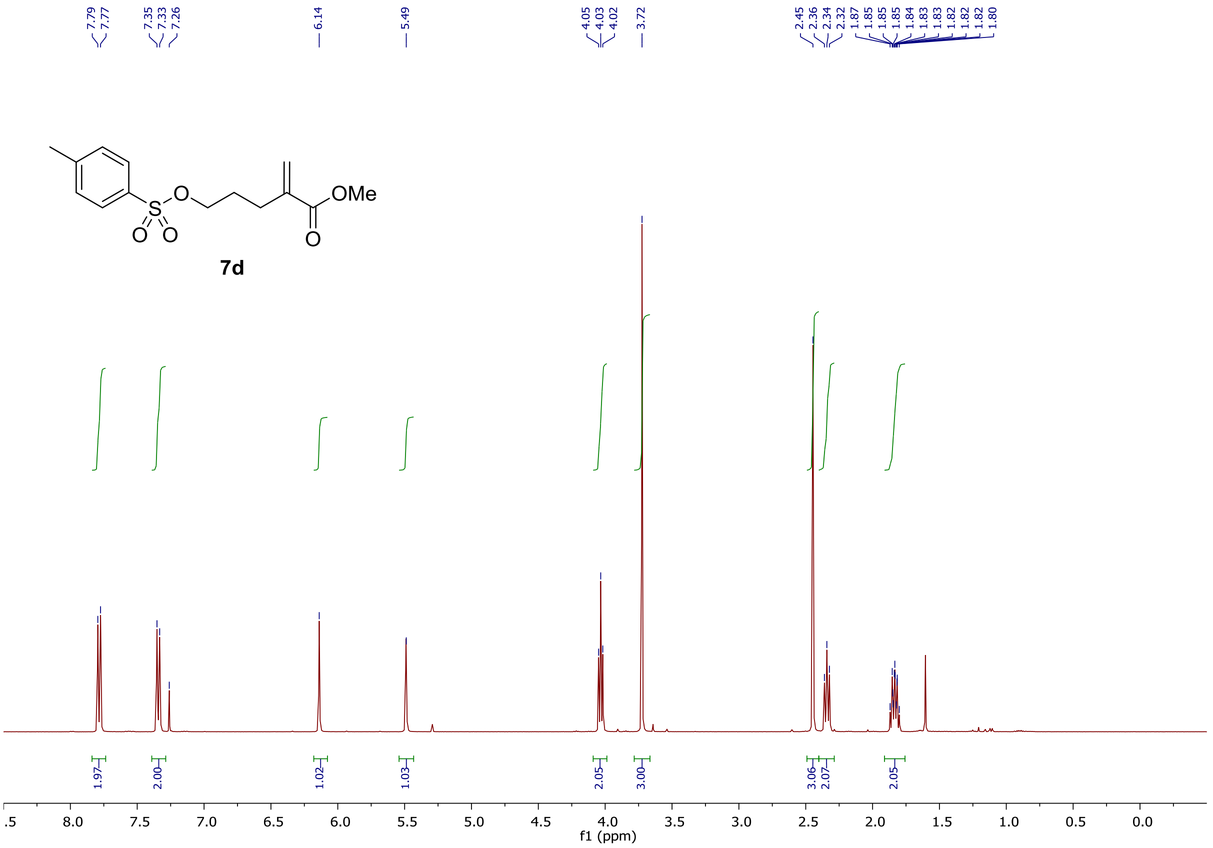

^{13}C NMR (101 MHz, $CDCl_3$)

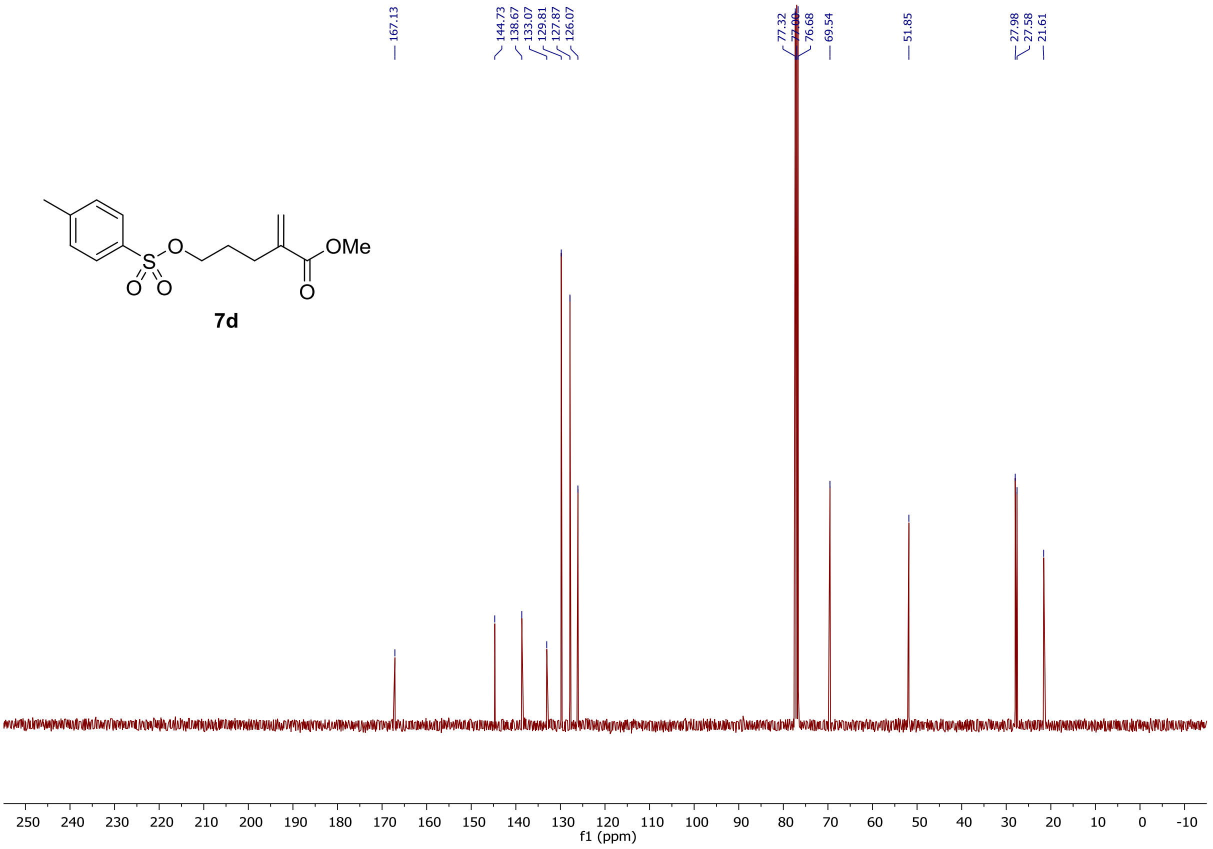

^{1}H NMR (400 MHz, $CDCl_3$)

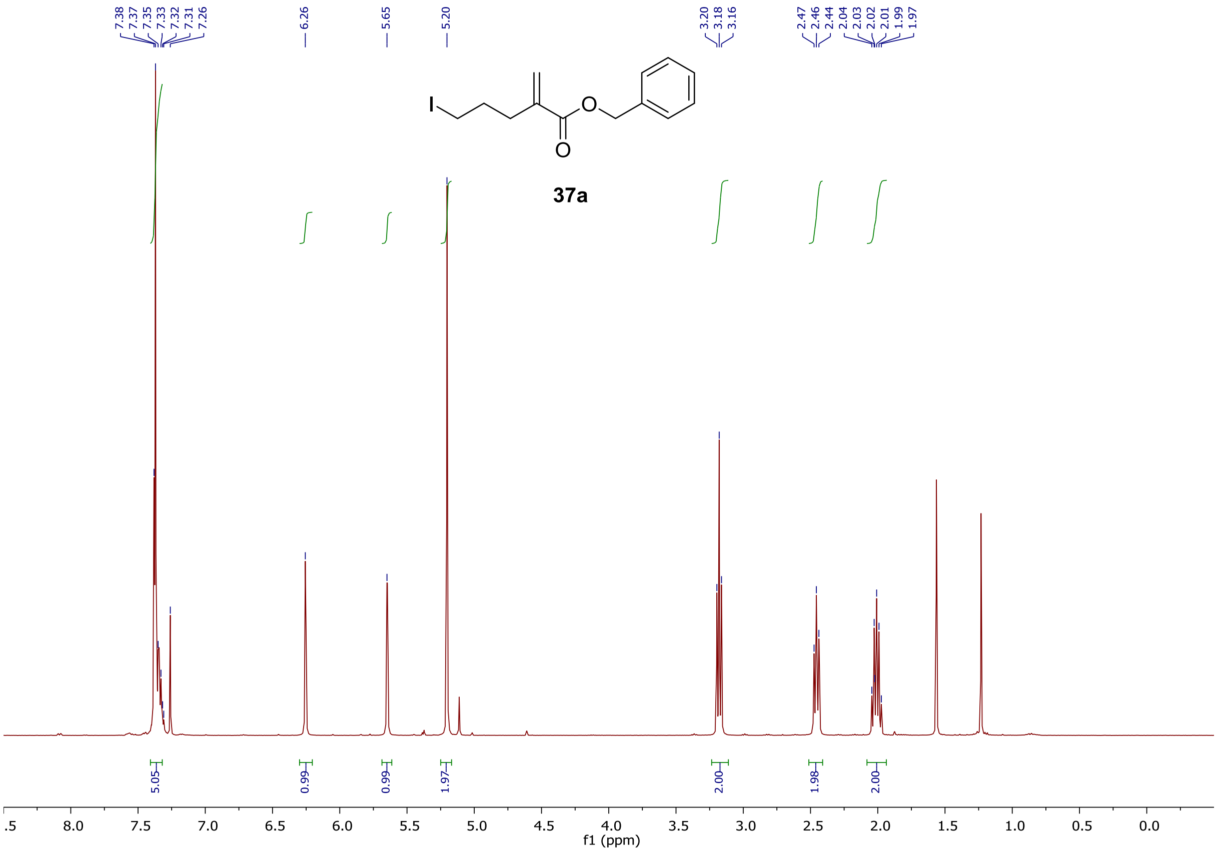

^{13}C NMR (101 MHz, $CDCl_3$)

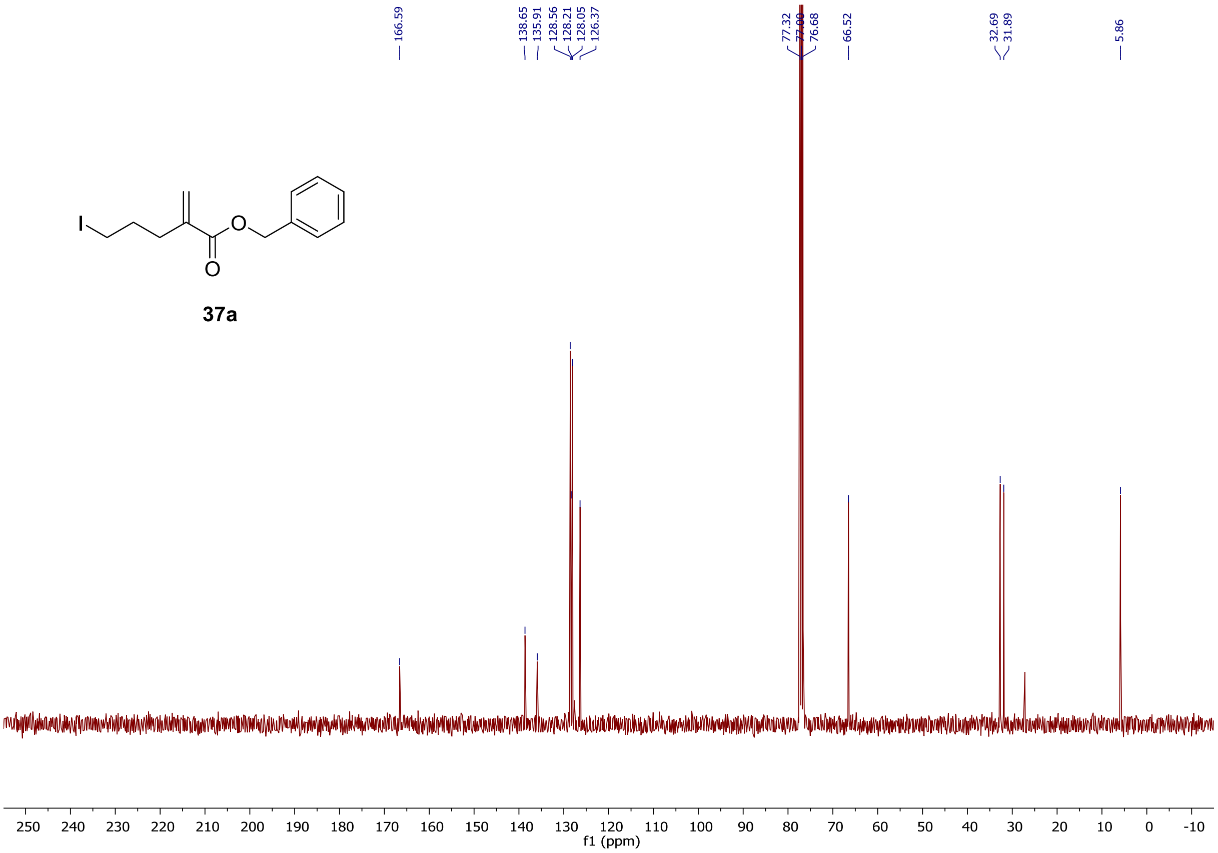

^{1}H NMR (400 MHz, $CDCl_3$)

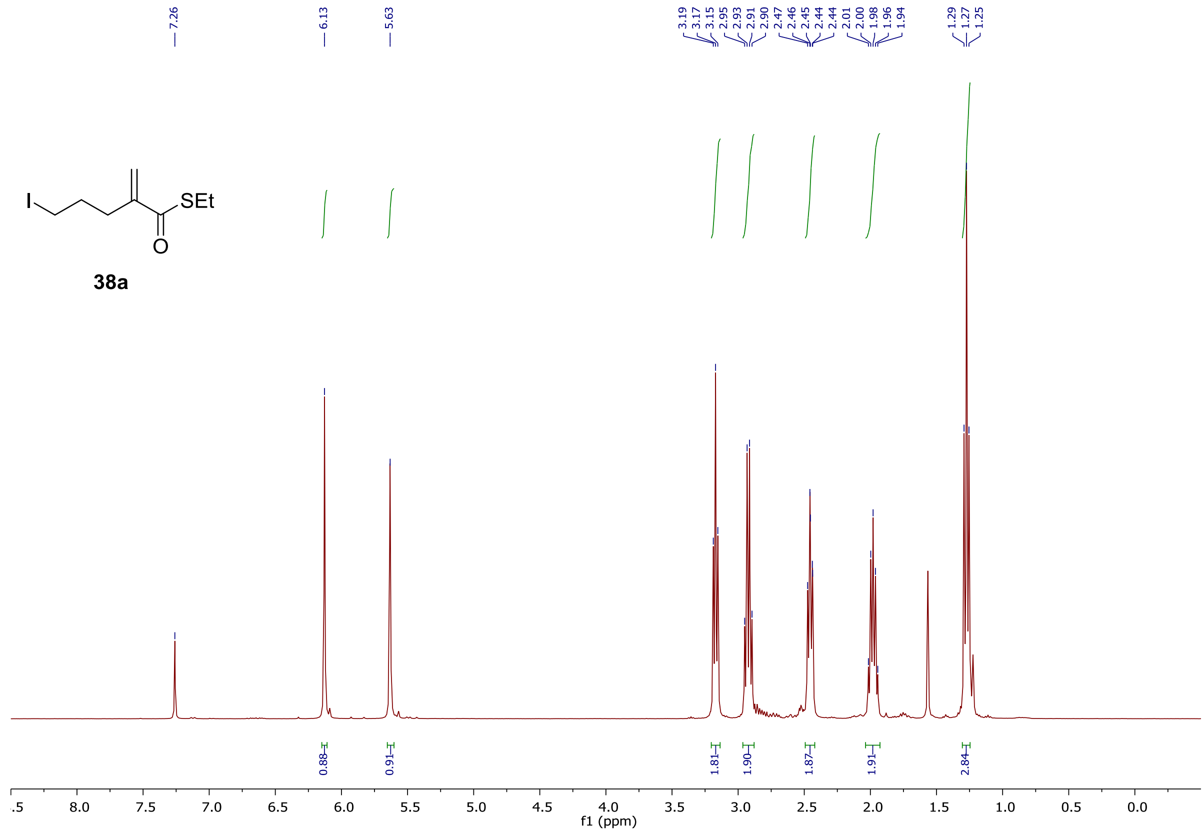

^{13}C NMR (101 MHz, $CDCl_3$)

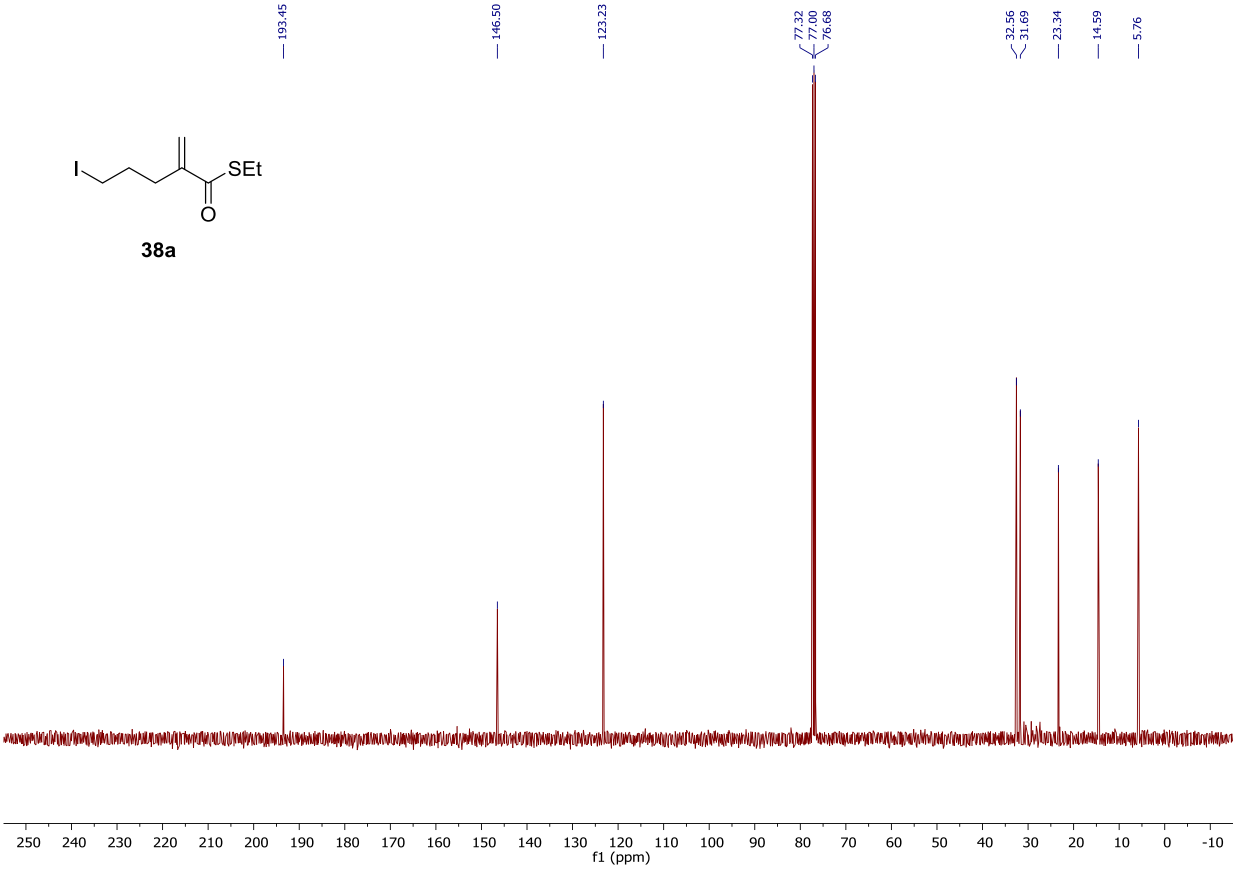

^{1}H NMR (400 MHz, $CDCl_3$)

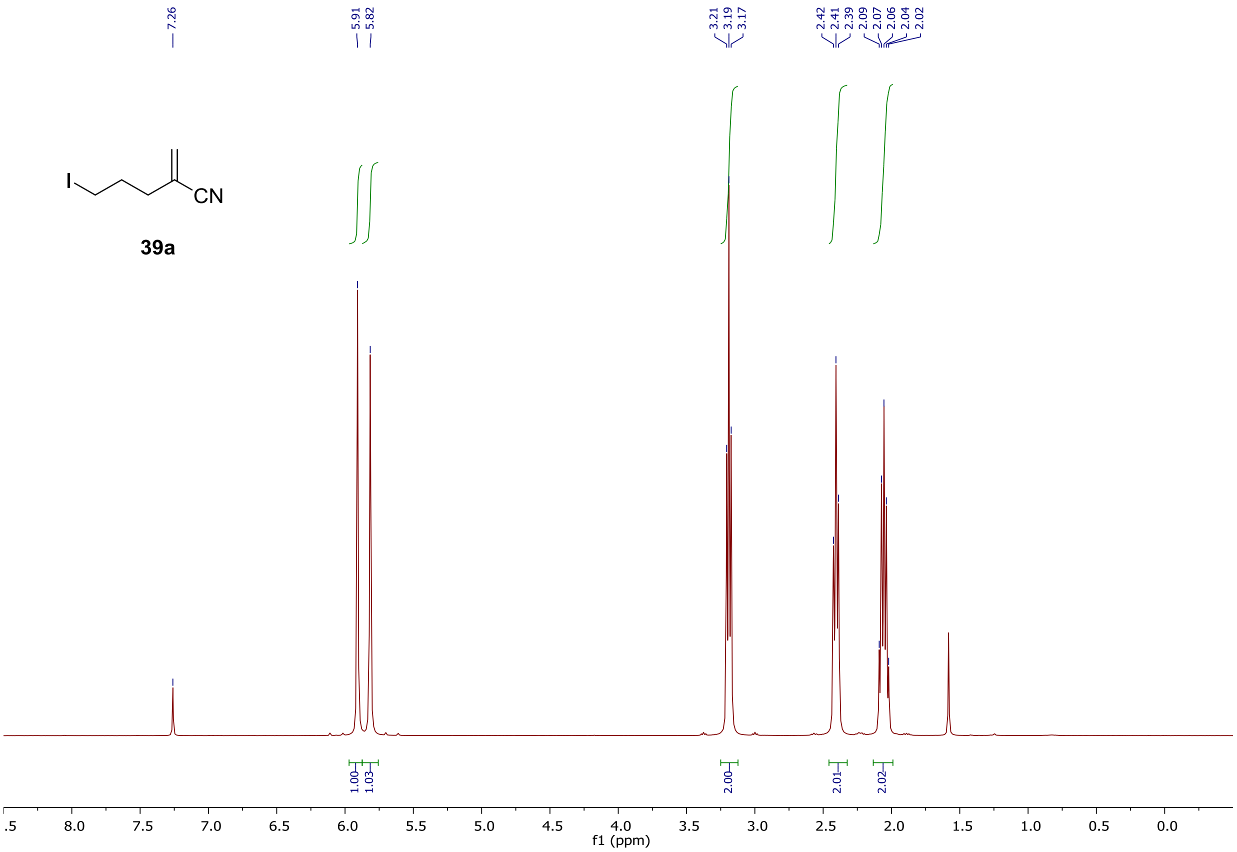

^{13}C NMR (101 MHz, $CDCl_3$)

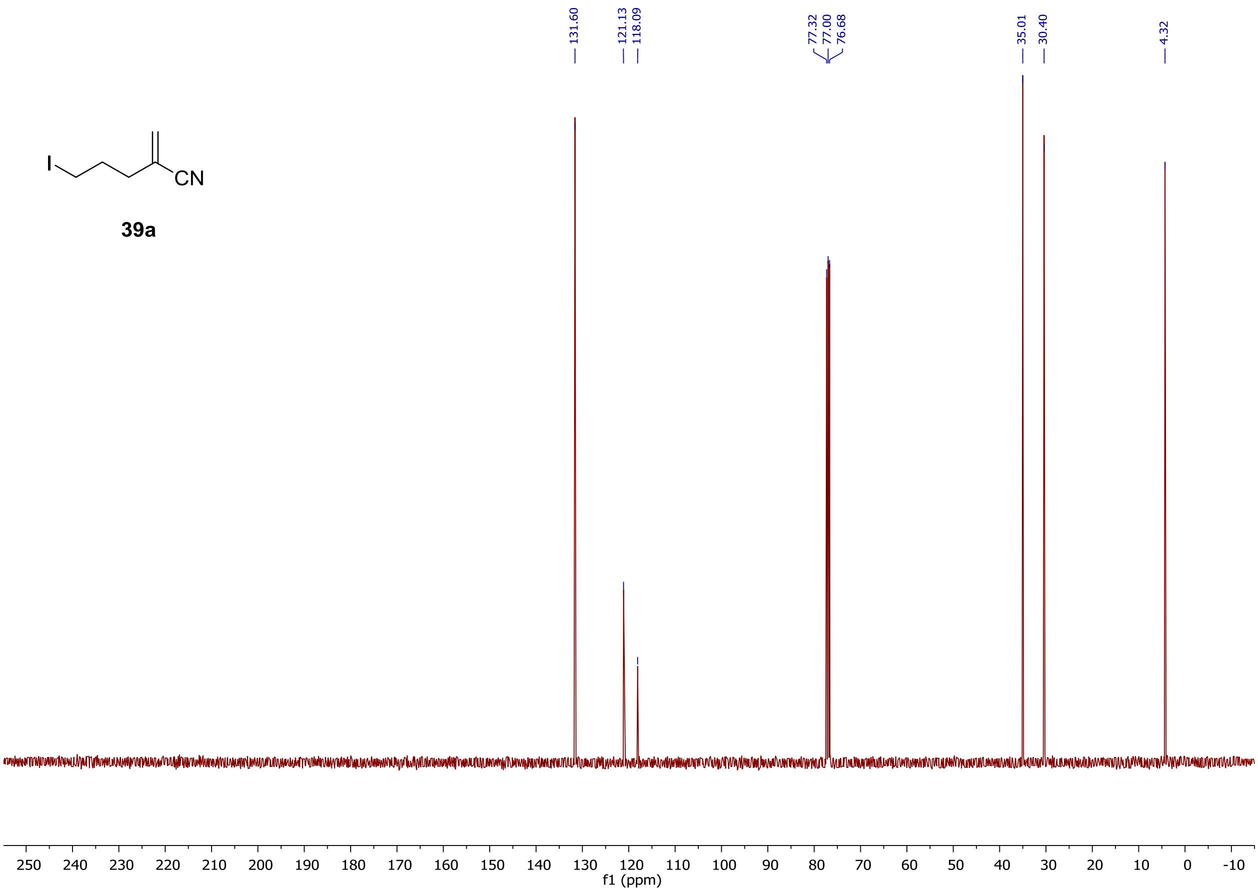

^{1}H NMR (400 MHz, $CDCl_3$)

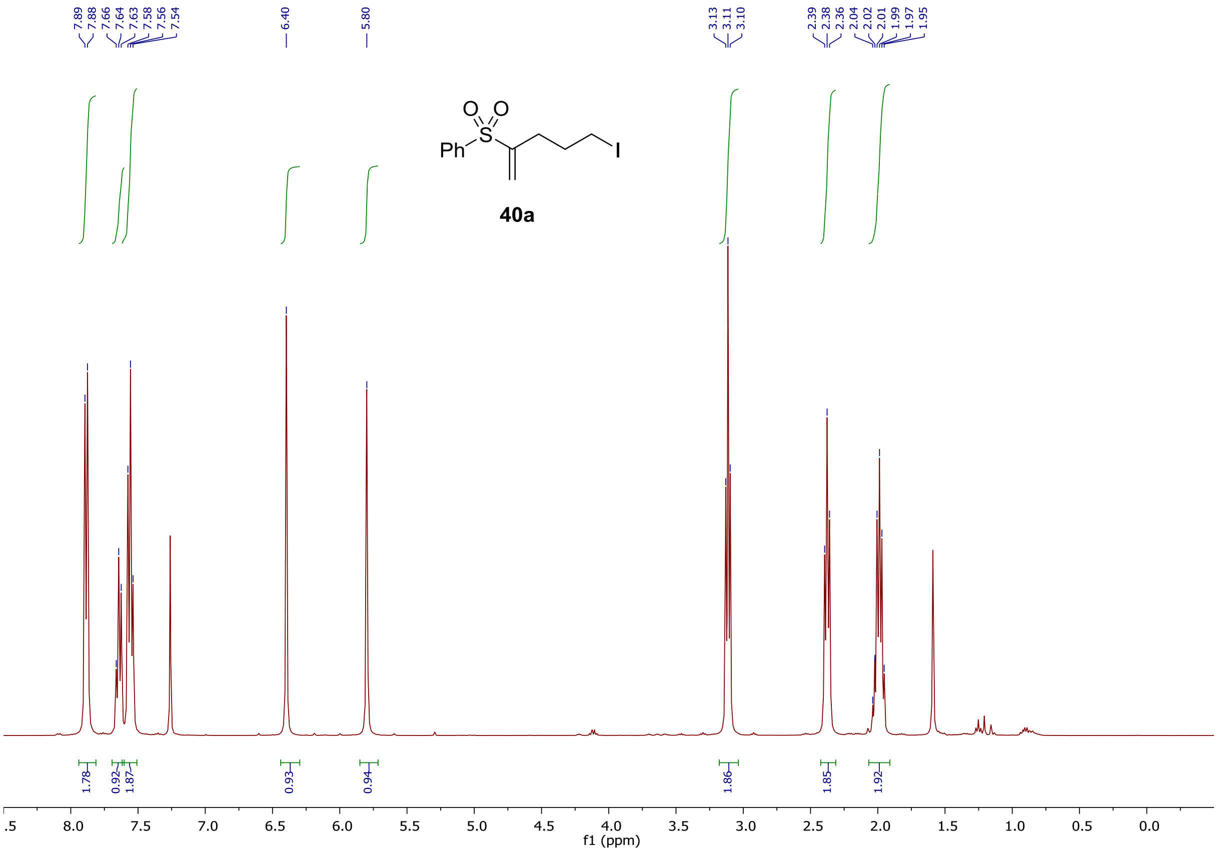

^{13}C NMR (101 MHz, $CDCl_3$)

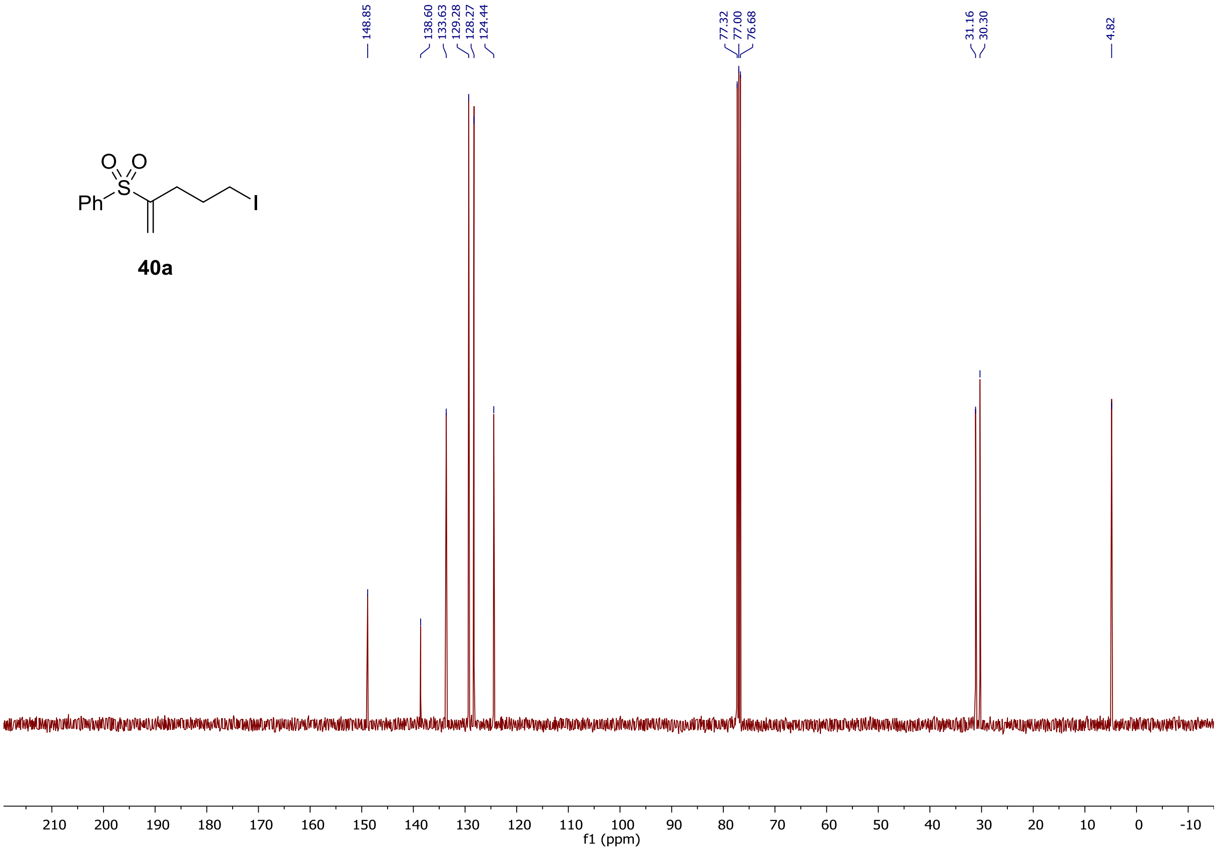

^{1}H NMR (400 MHz, CDCl$_3$)

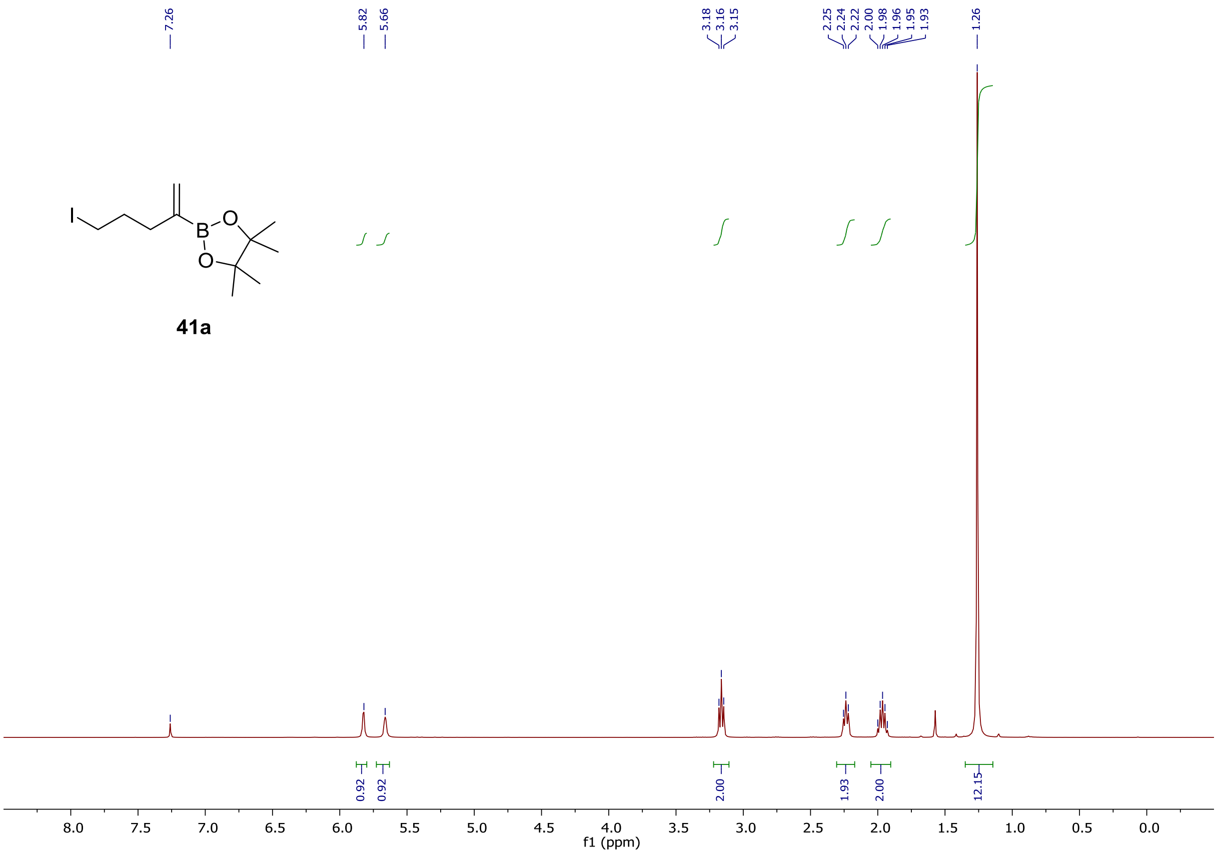

^{13}C NMR (101 MHz, CDCl$_3$)

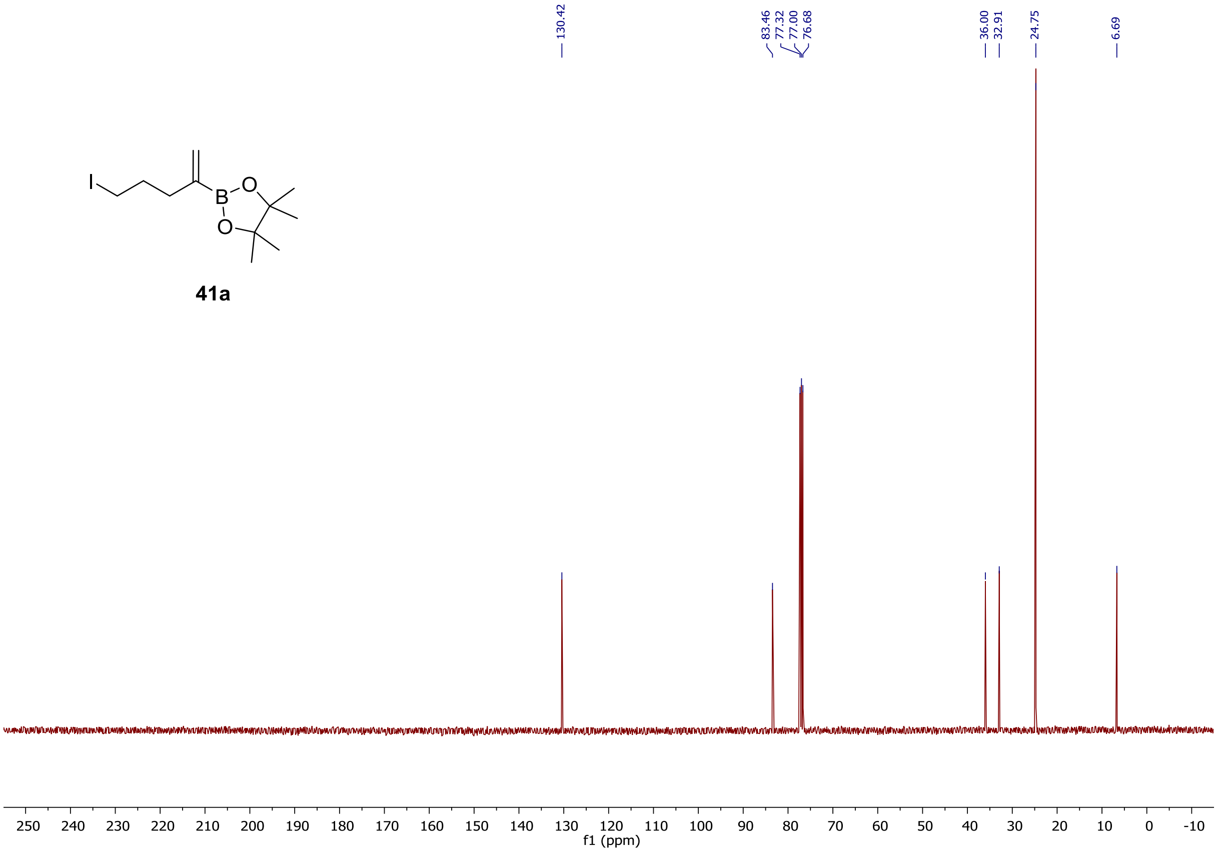

^{1}H NMR (400 MHz, $CDCl_3$)

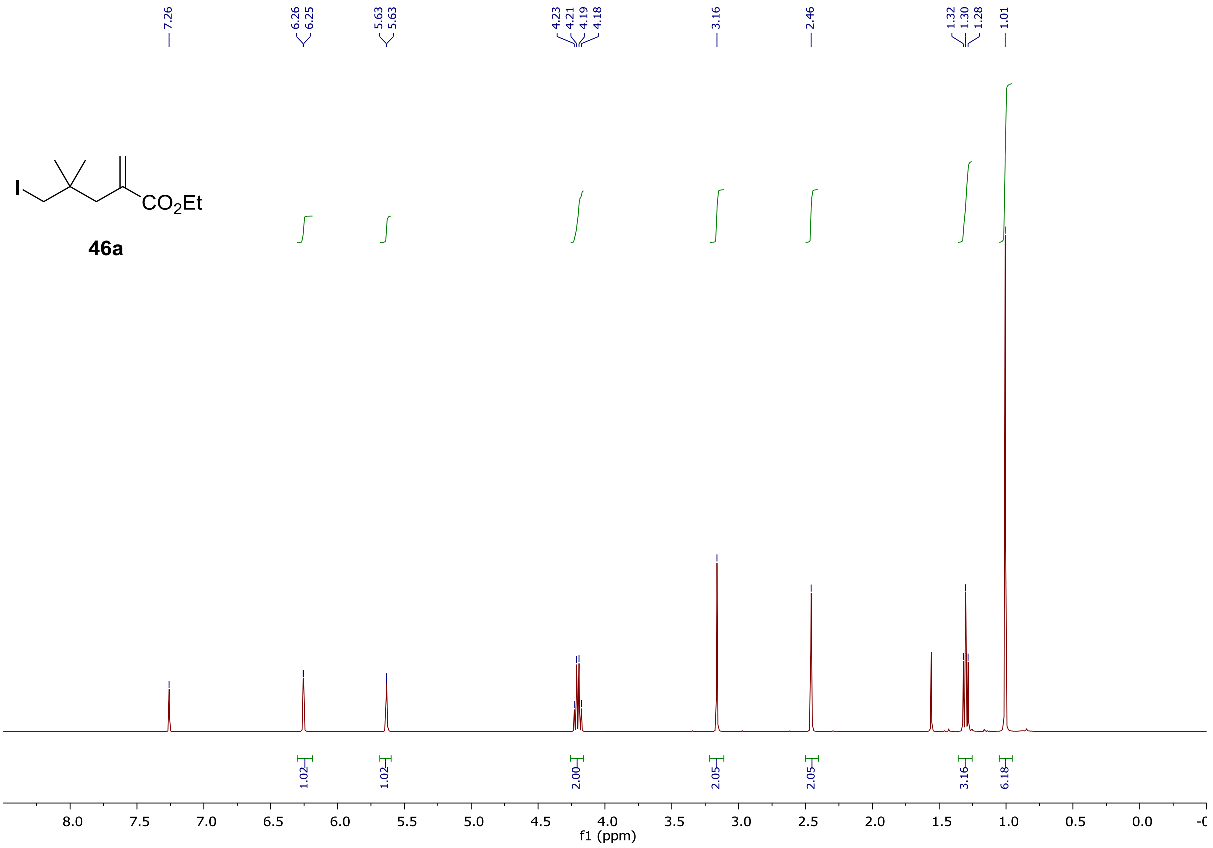

^{13}C NMR (101 MHz, $CDCl_3$)

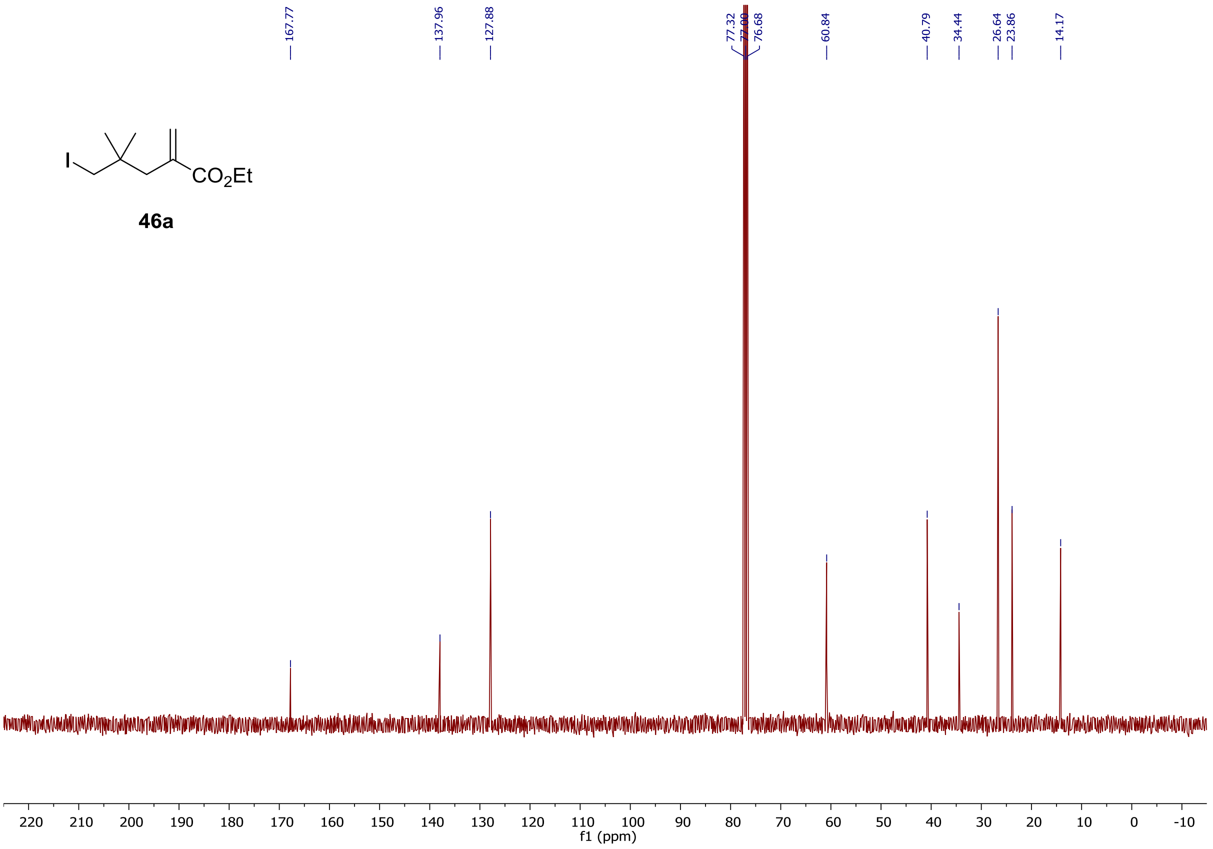

^{1}H NMR (400 MHz, $CDCl_3$)

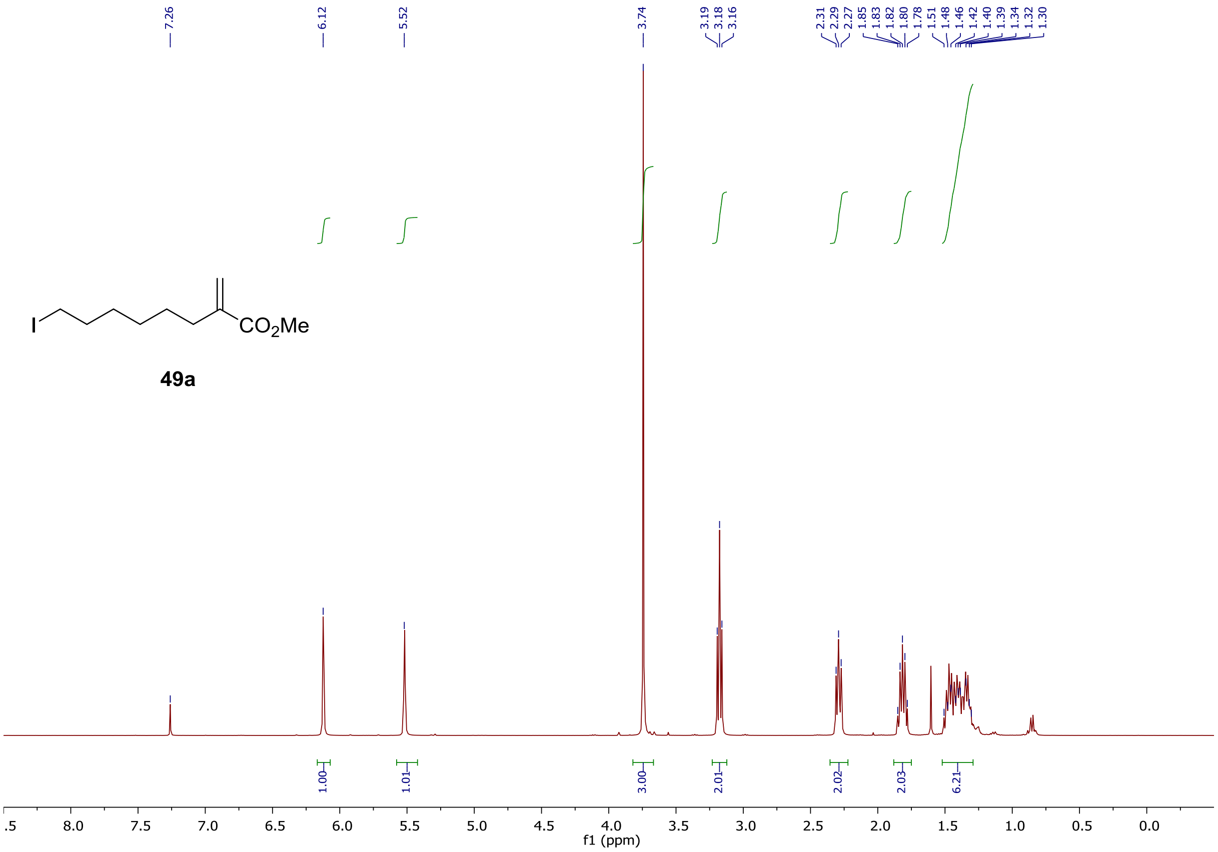

^{13}C NMR (101 MHz, $CDCl_3$)

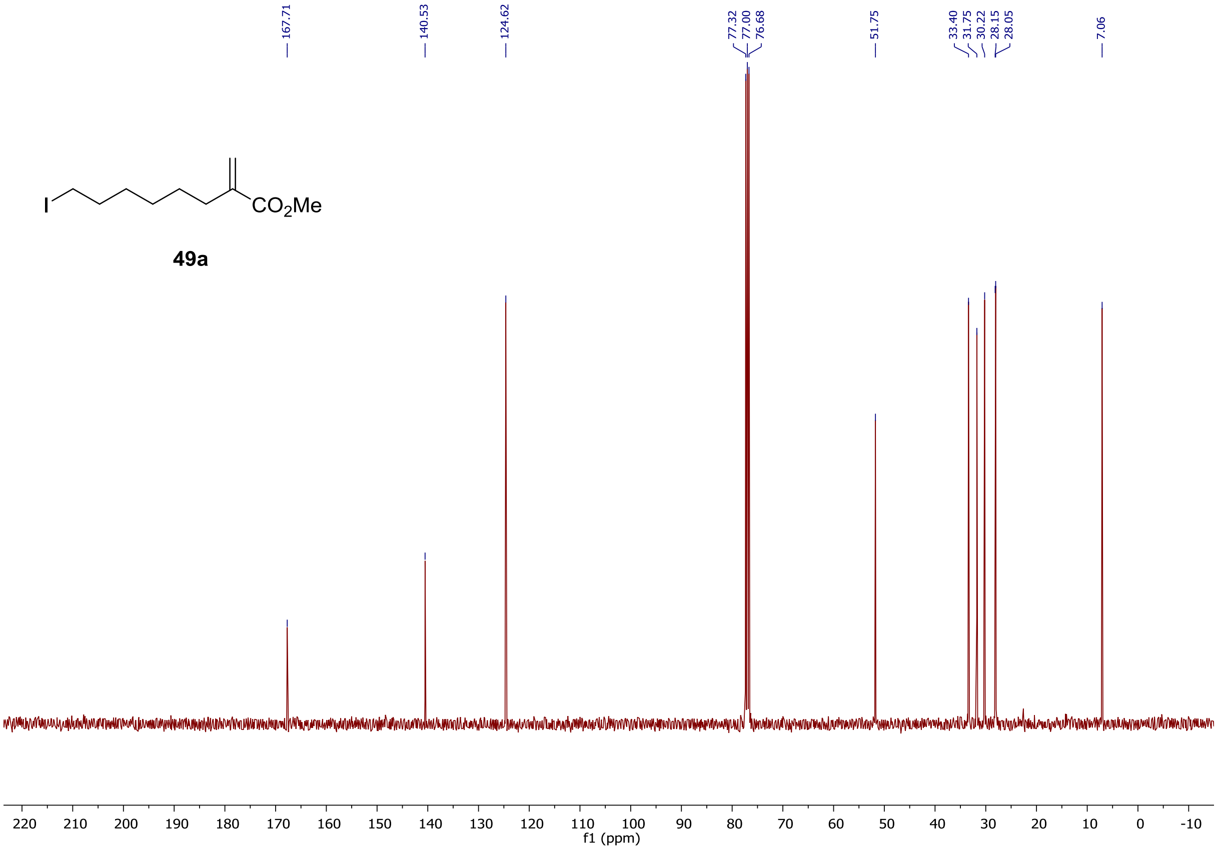

^{1}H NMR (400 MHz, $CDCl_3$)

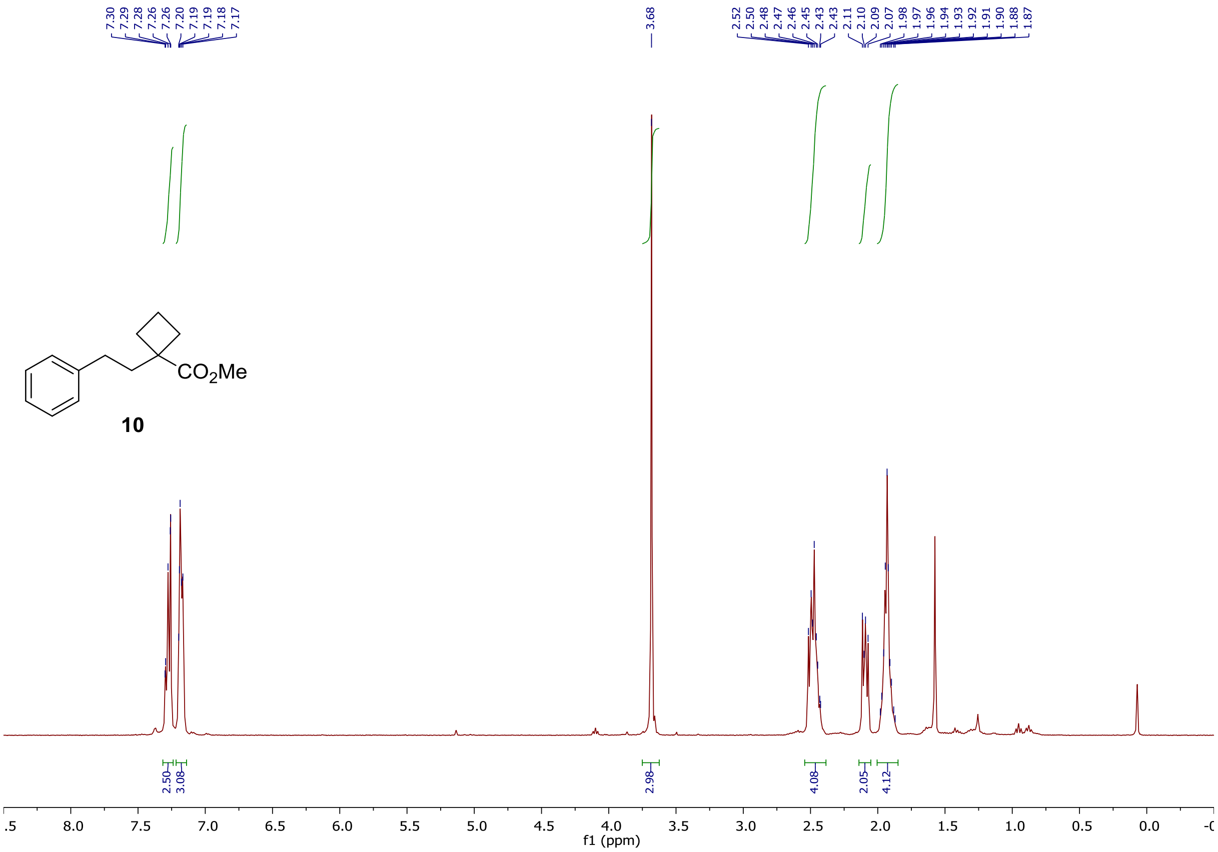

^{13}C NMR (101 MHz, $CDCl_3$)

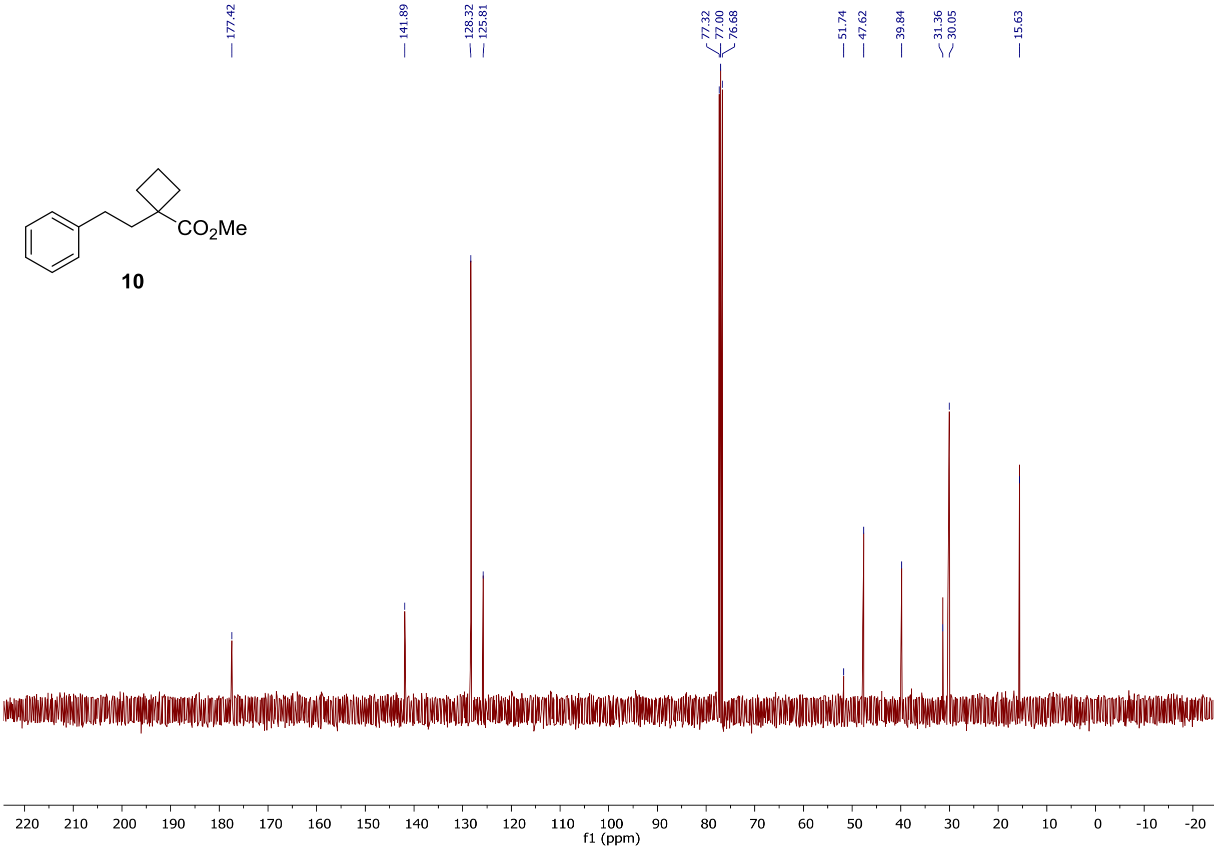

^{1}H NMR (400 MHz, $CDCl_3$)

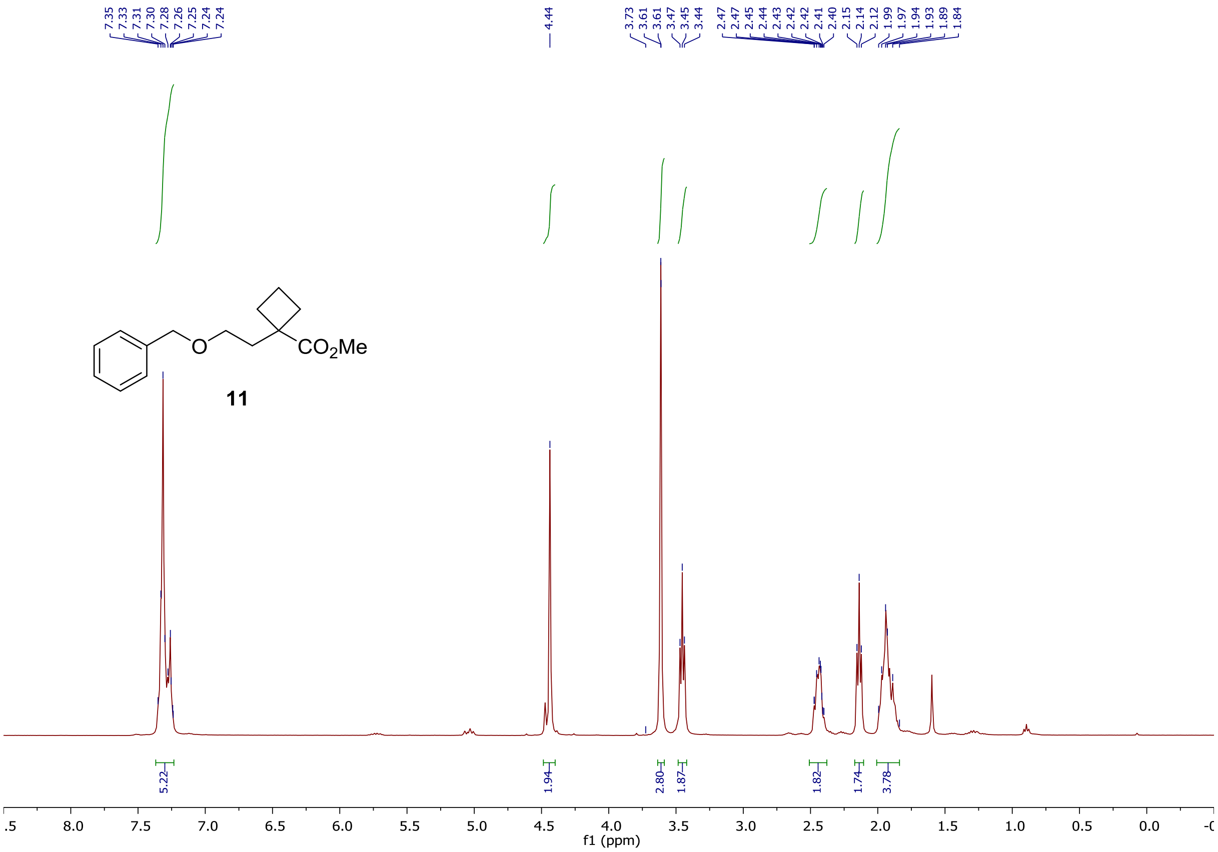

^{13}C NMR (101 MHz, $CDCl_3$)

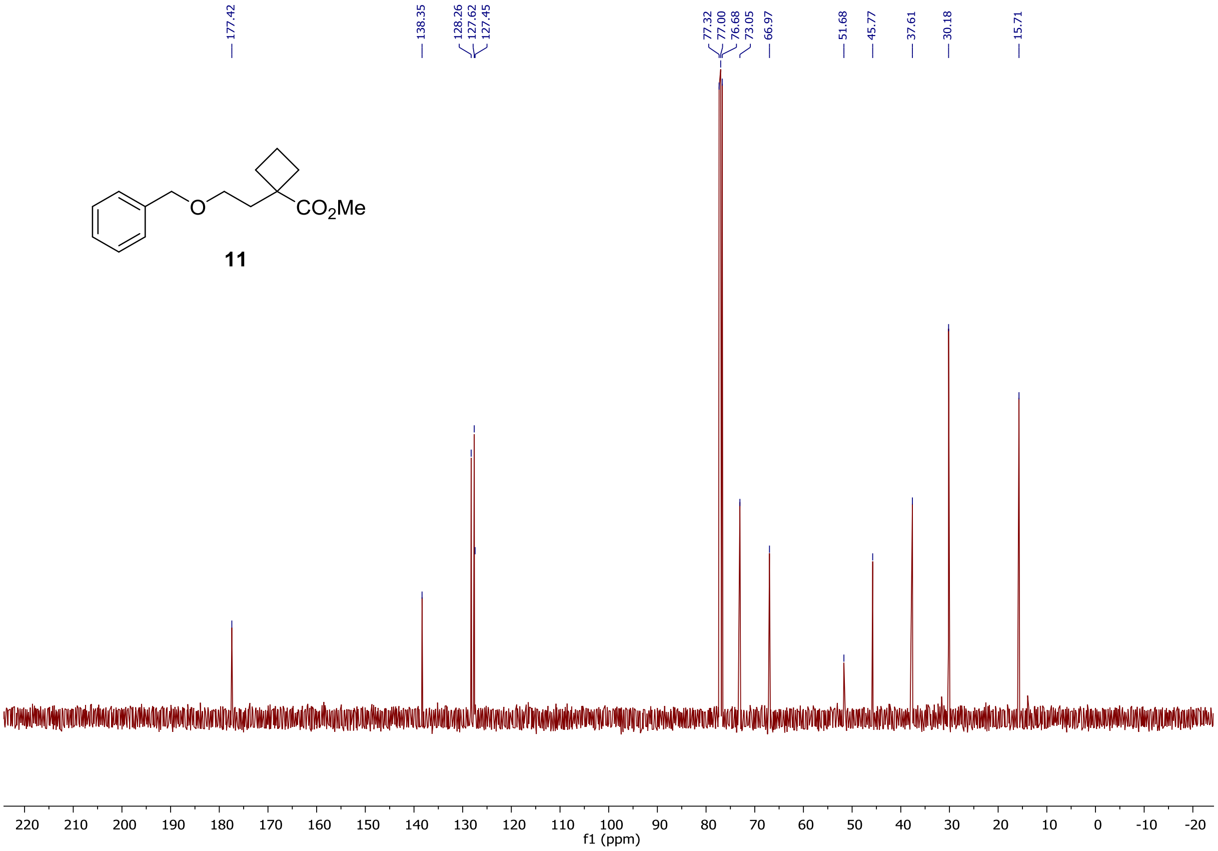

^{1}H NMR (400 MHz, $CDCl_3$)

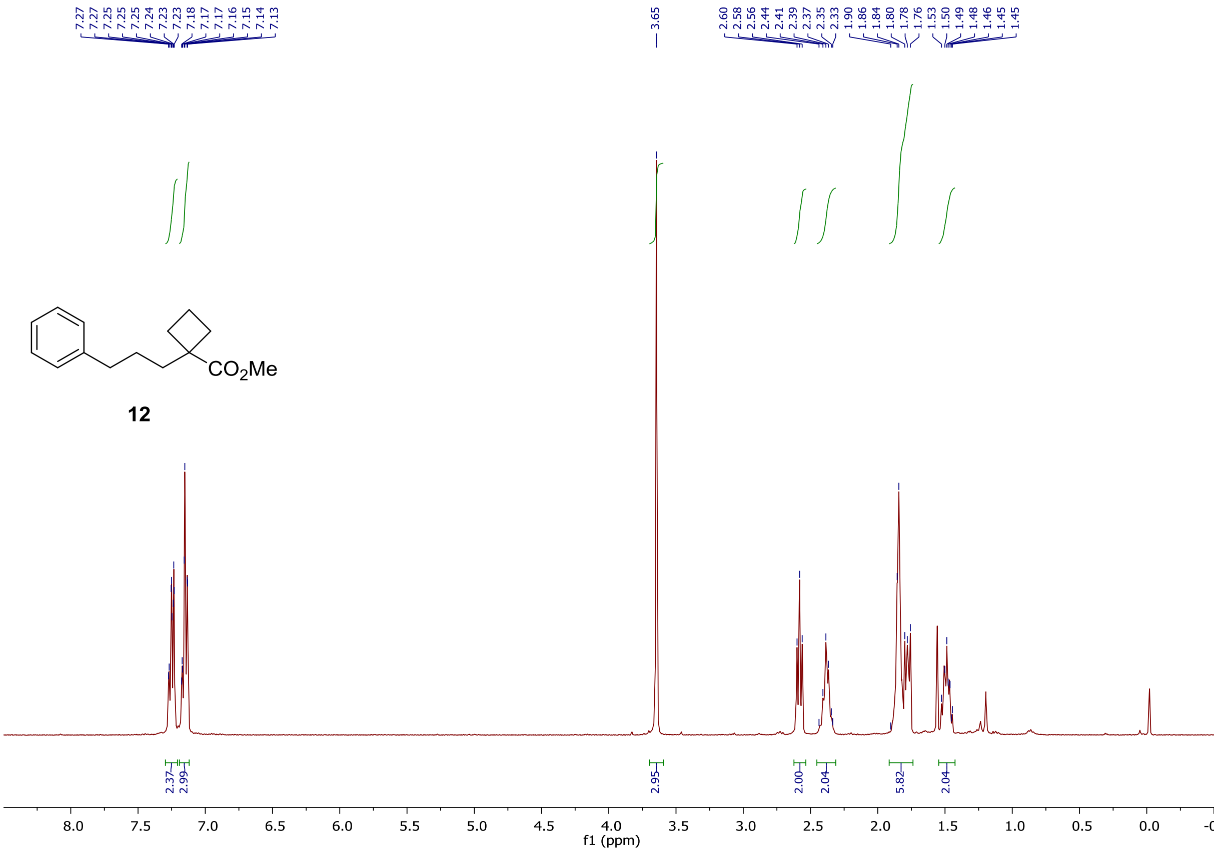

^{13}C NMR (101 MHz, $CDCl_3$)

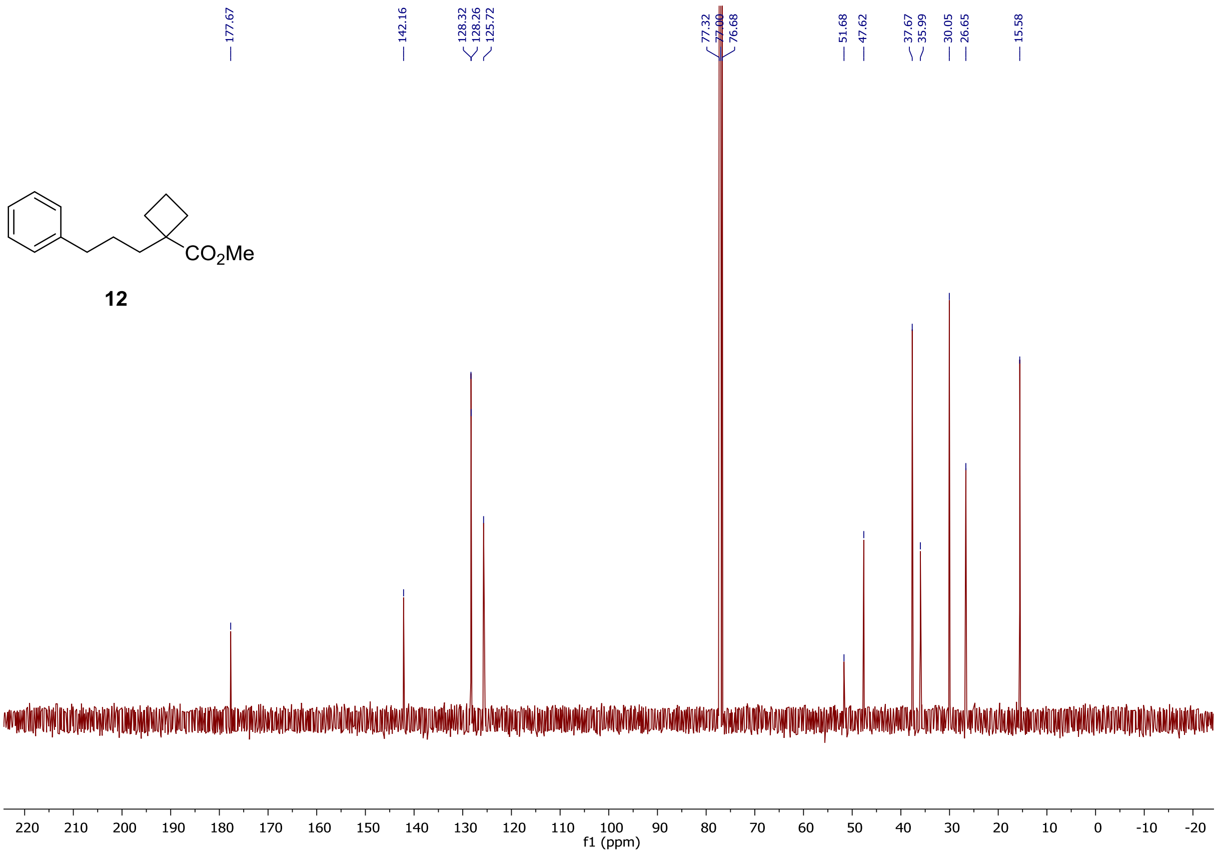

^{1}H NMR (400 MHz, $CDCl_3$)

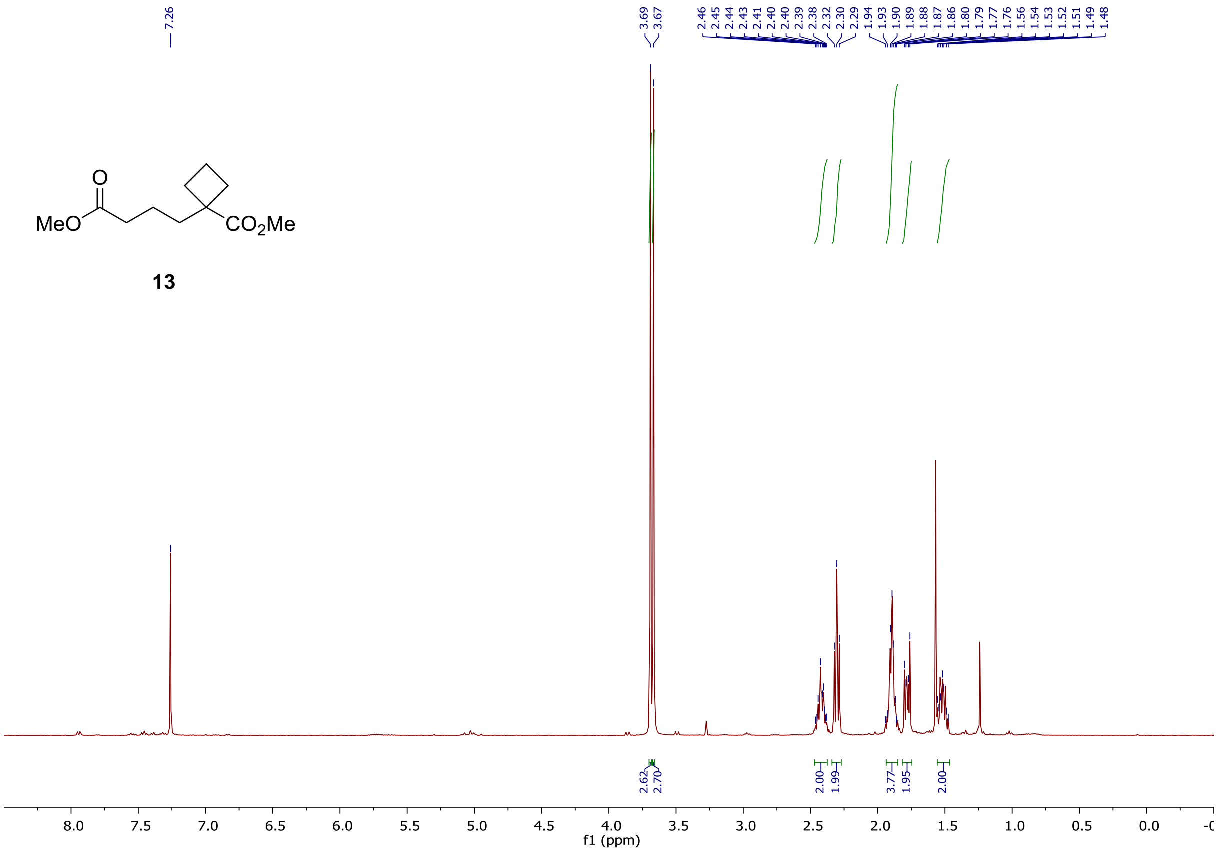

^{13}C NMR (101 MHz, $CDCl_3$)

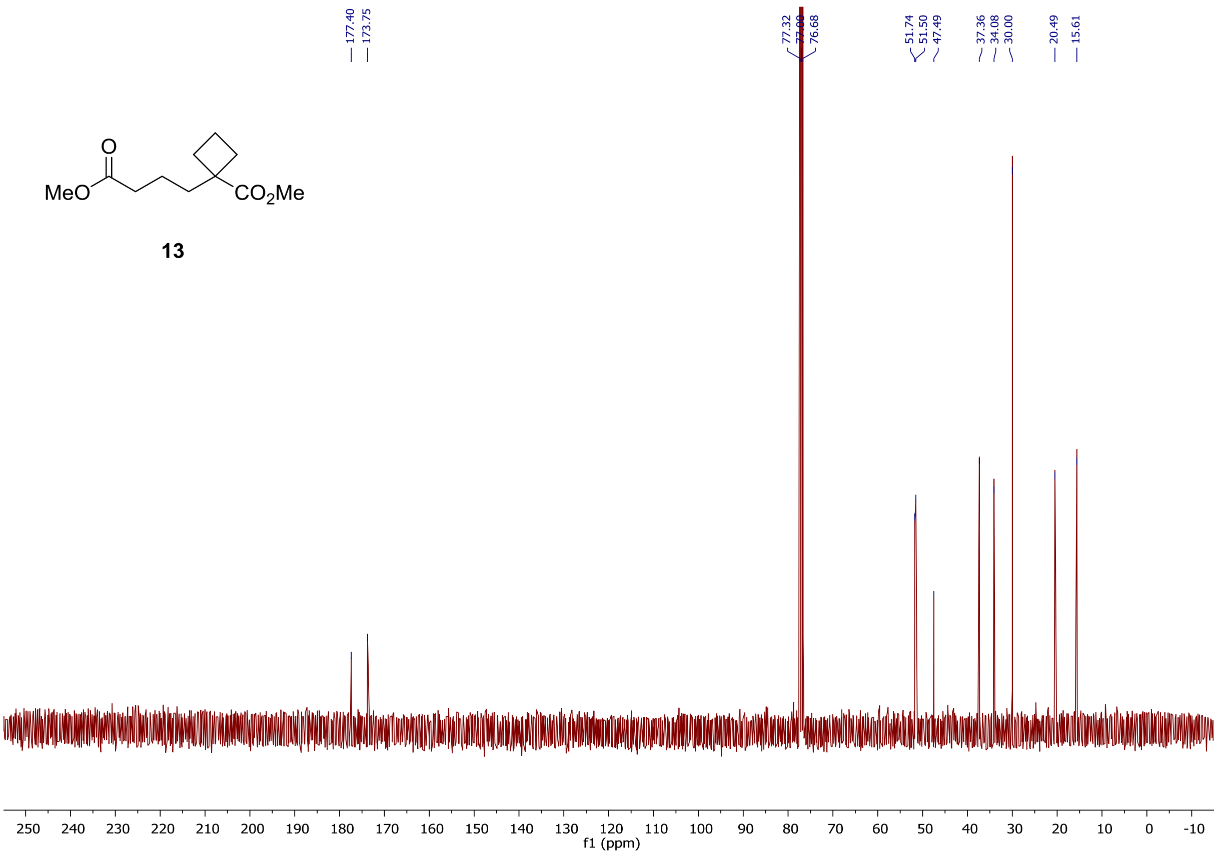

^{1}H NMR (400 MHz, $CDCl_3$)

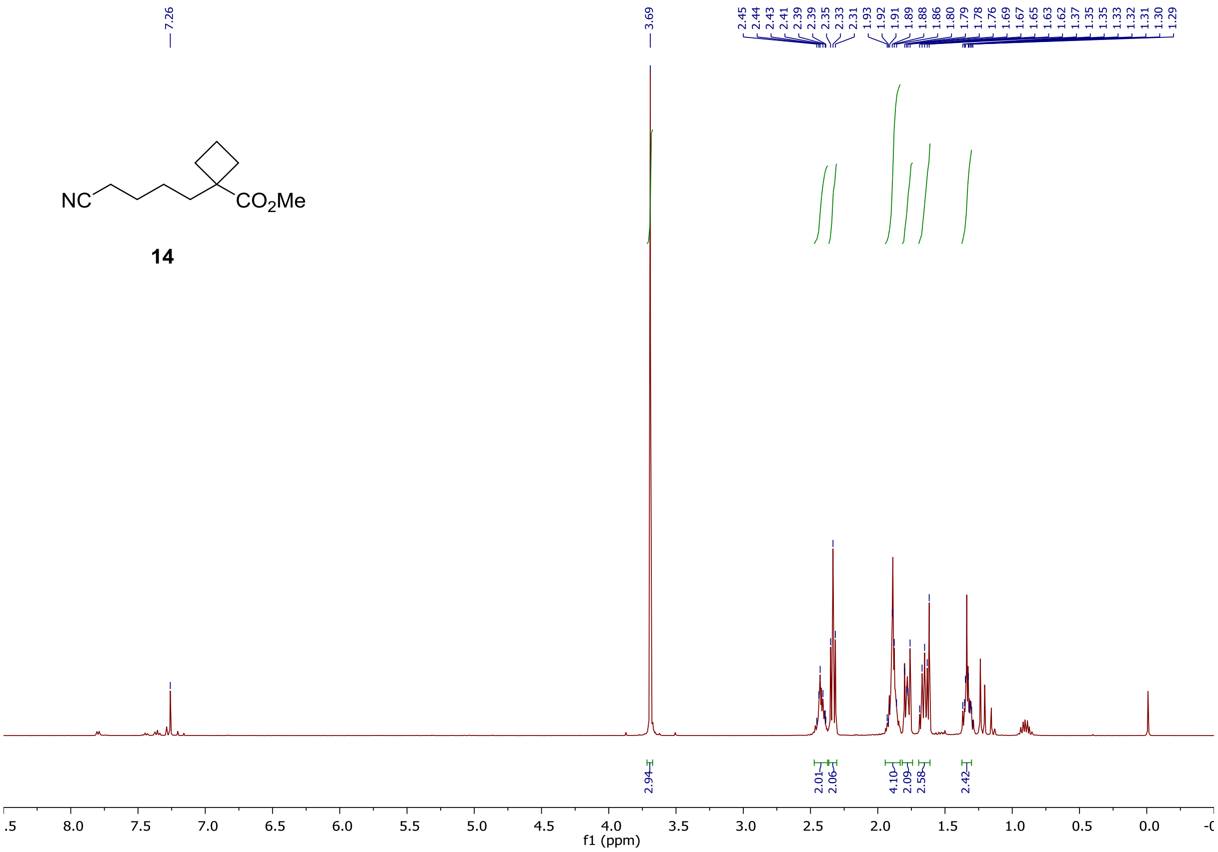

^{13}C NMR (101 MHz, $CDCl_3$)

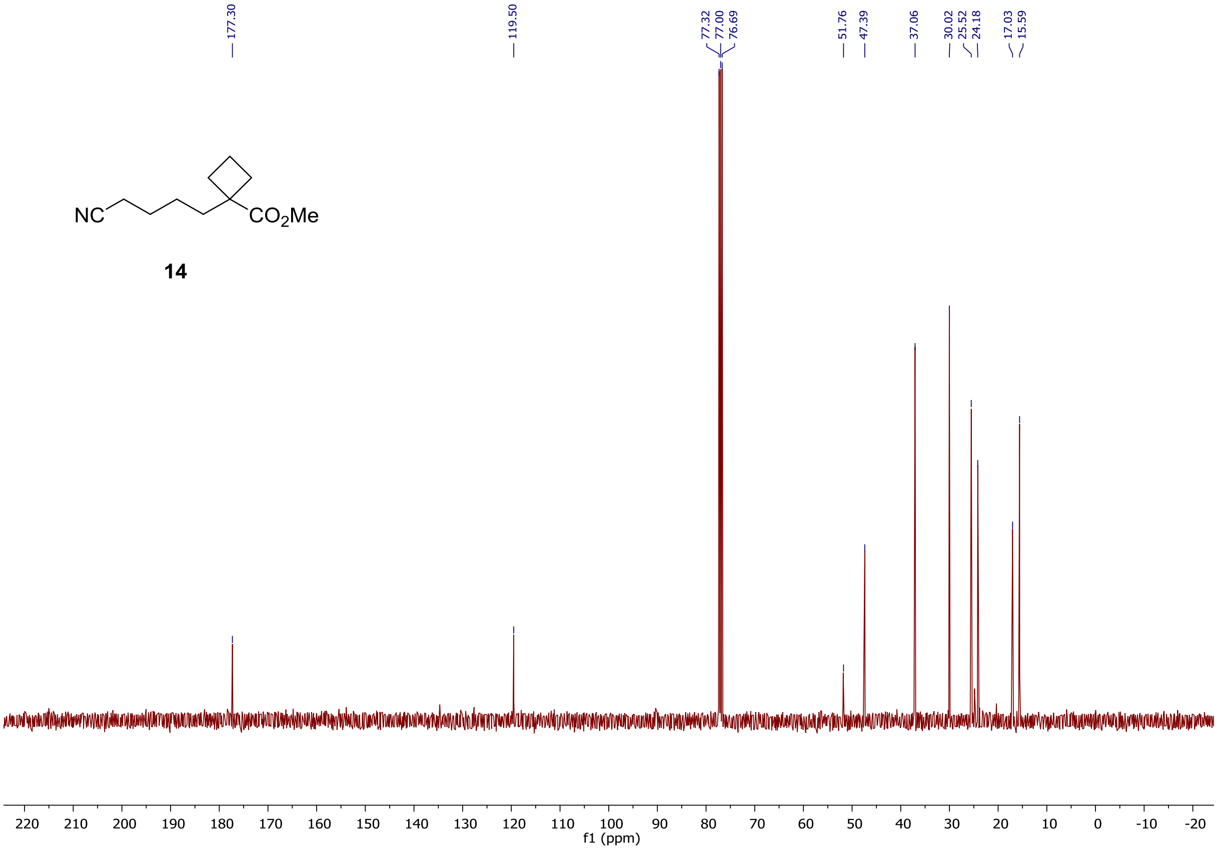

^{1}H NMR (400 MHz, $CDCl_3$)

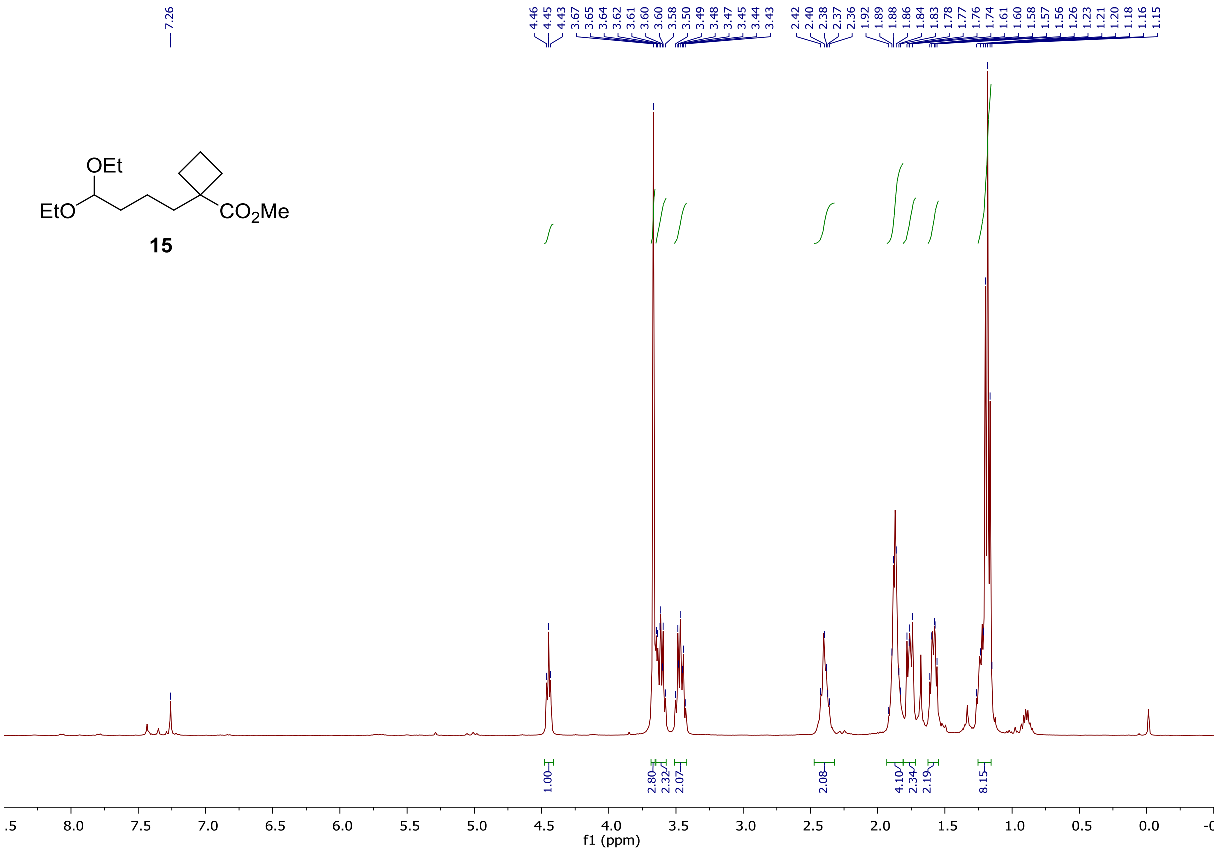

^{13}C NMR (101 MHz, $CDCl_3$)

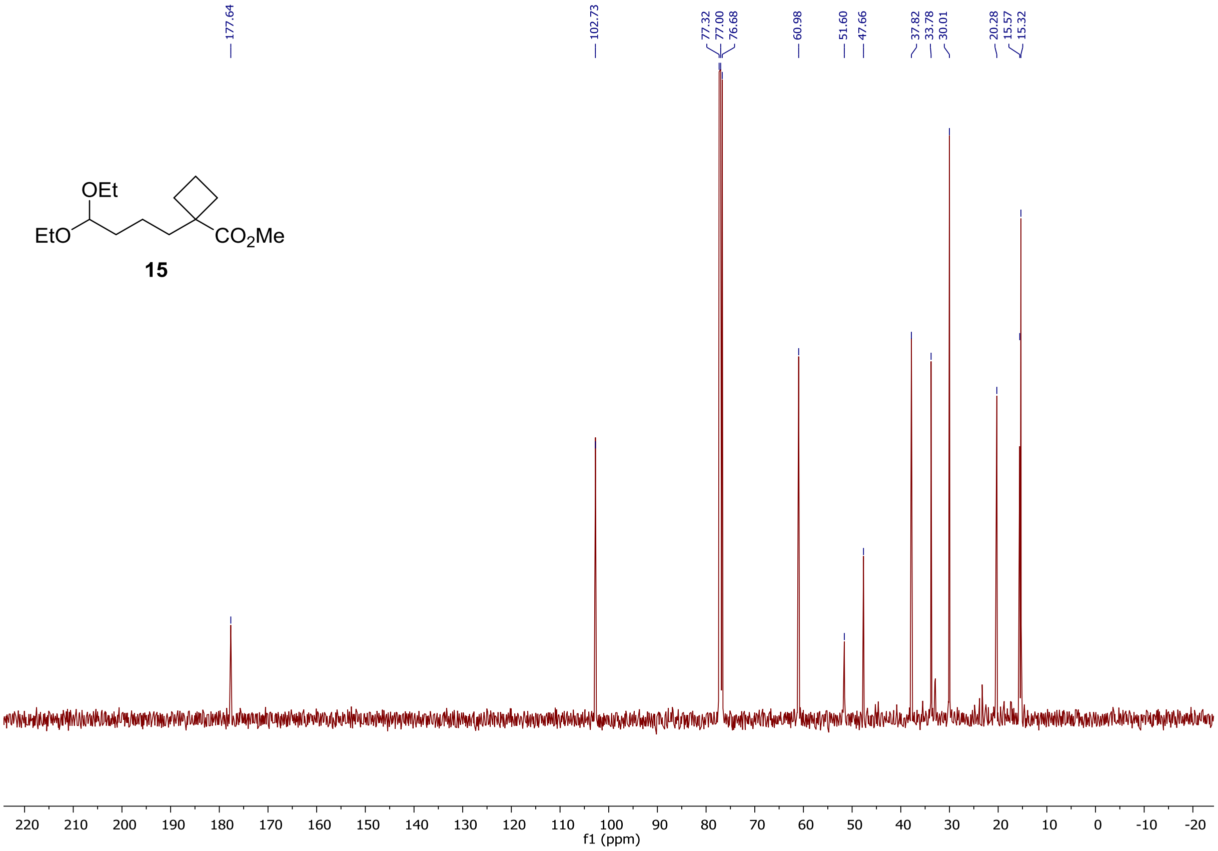

^{1}H NMR (400 MHz, $CDCl_3$)

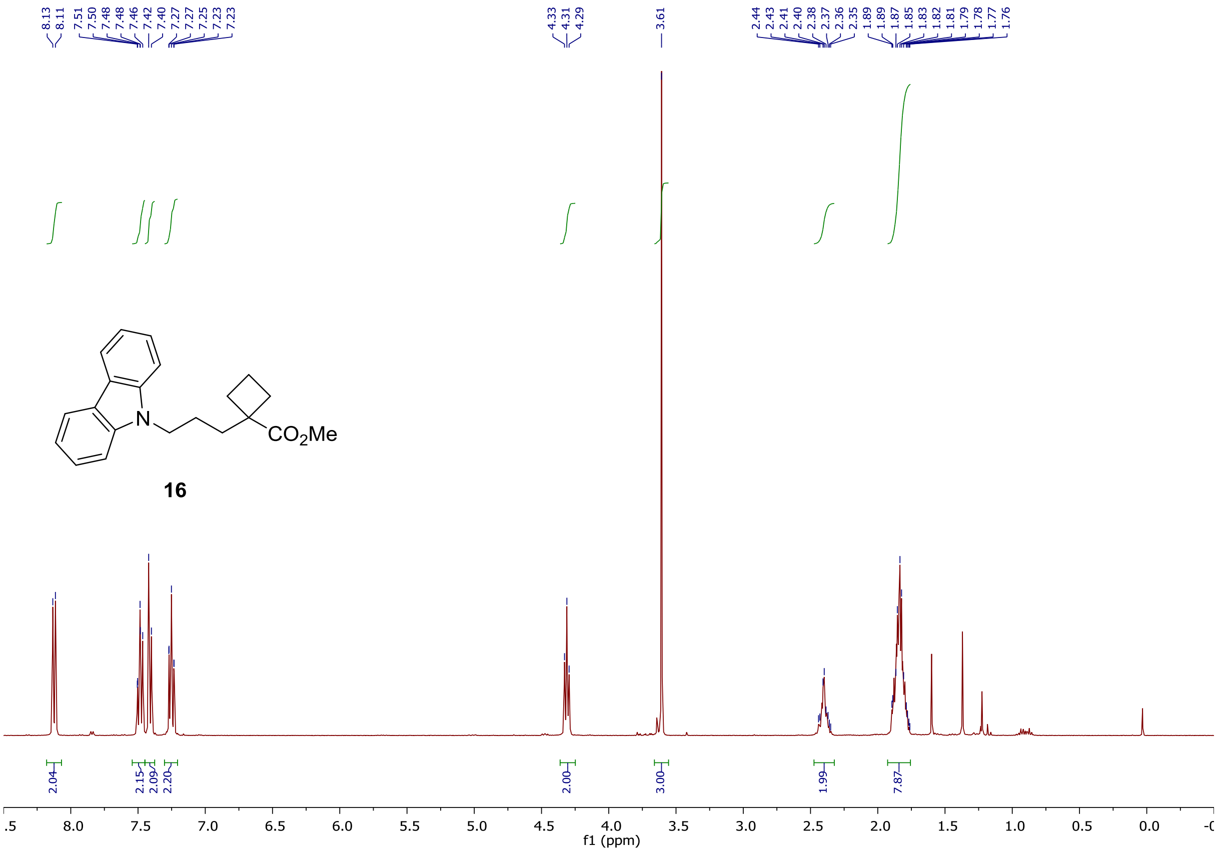

^{13}C NMR (101 MHz, $CDCl_3$)

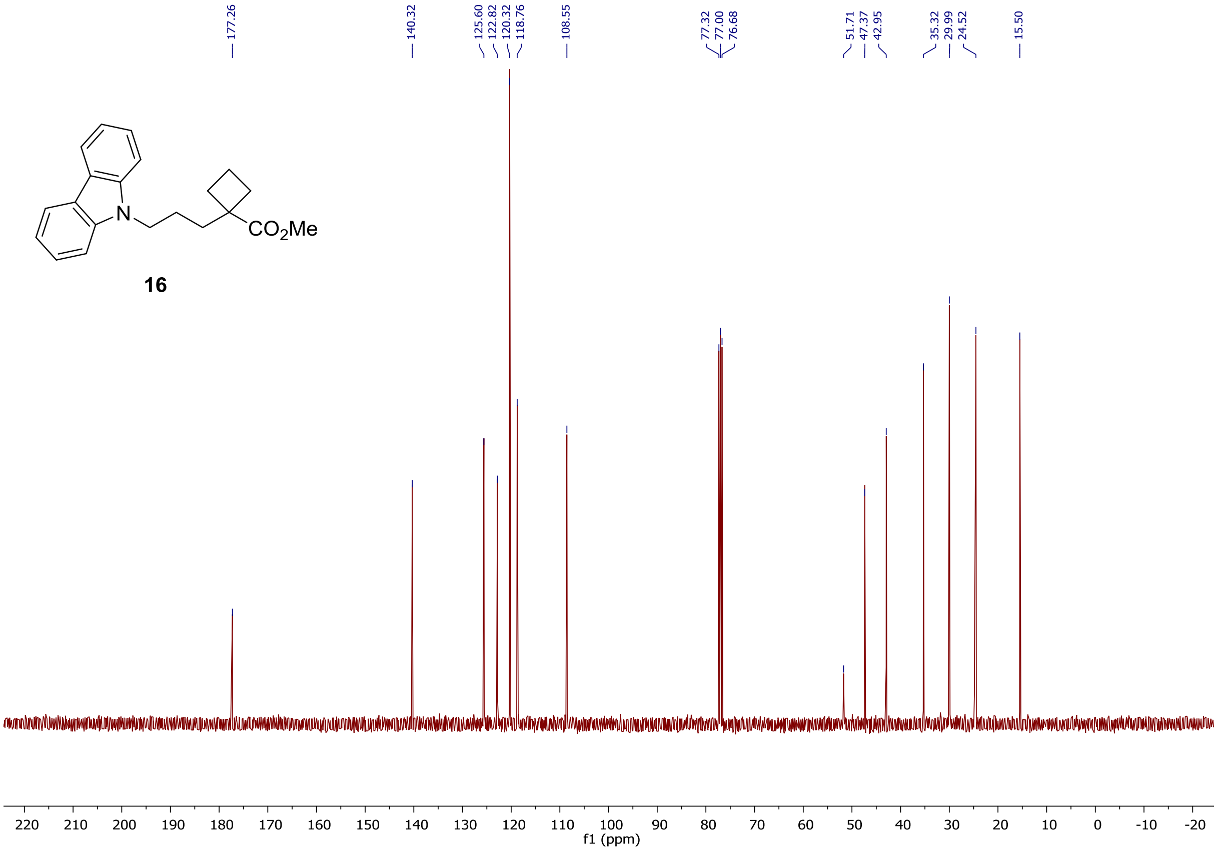

^{1}H NMR (400 MHz, $CDCl_3$)

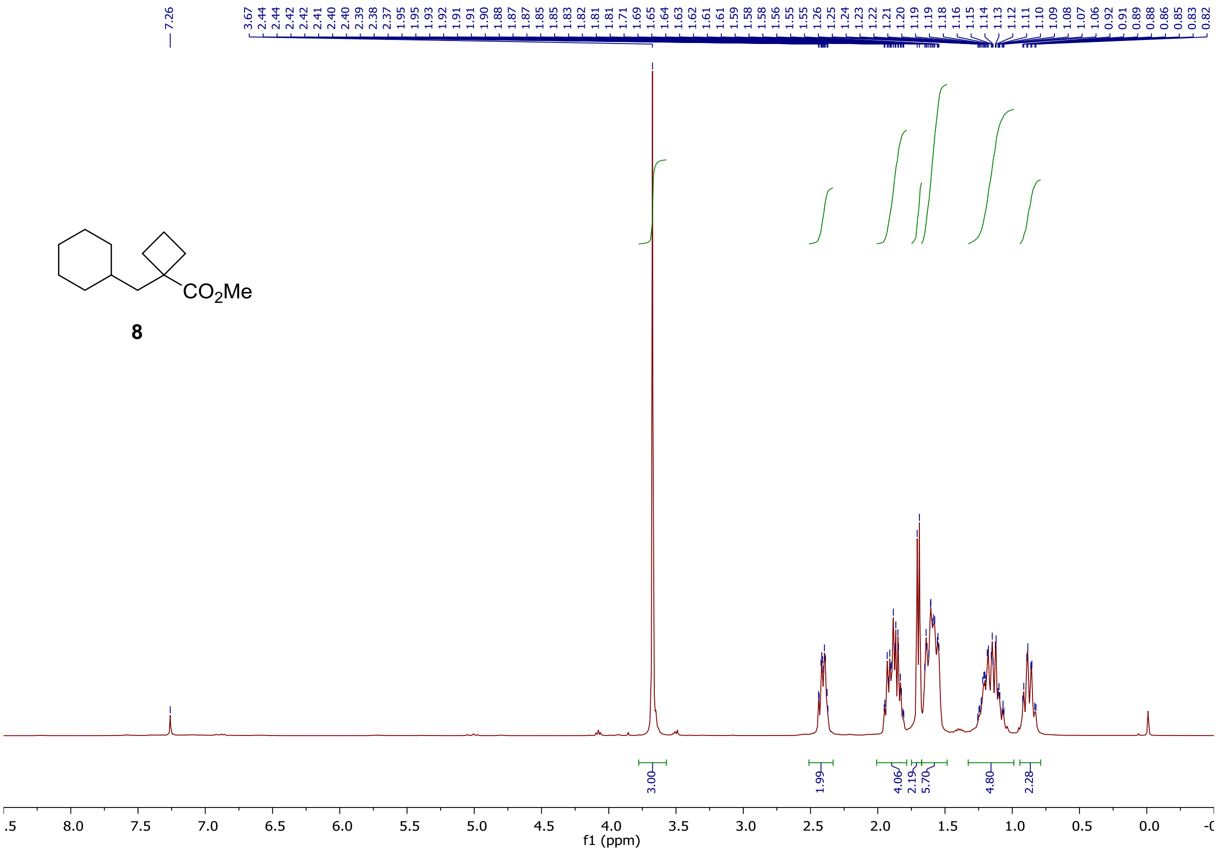

^{13}C NMR (101 MHz, $CDCl_3$)

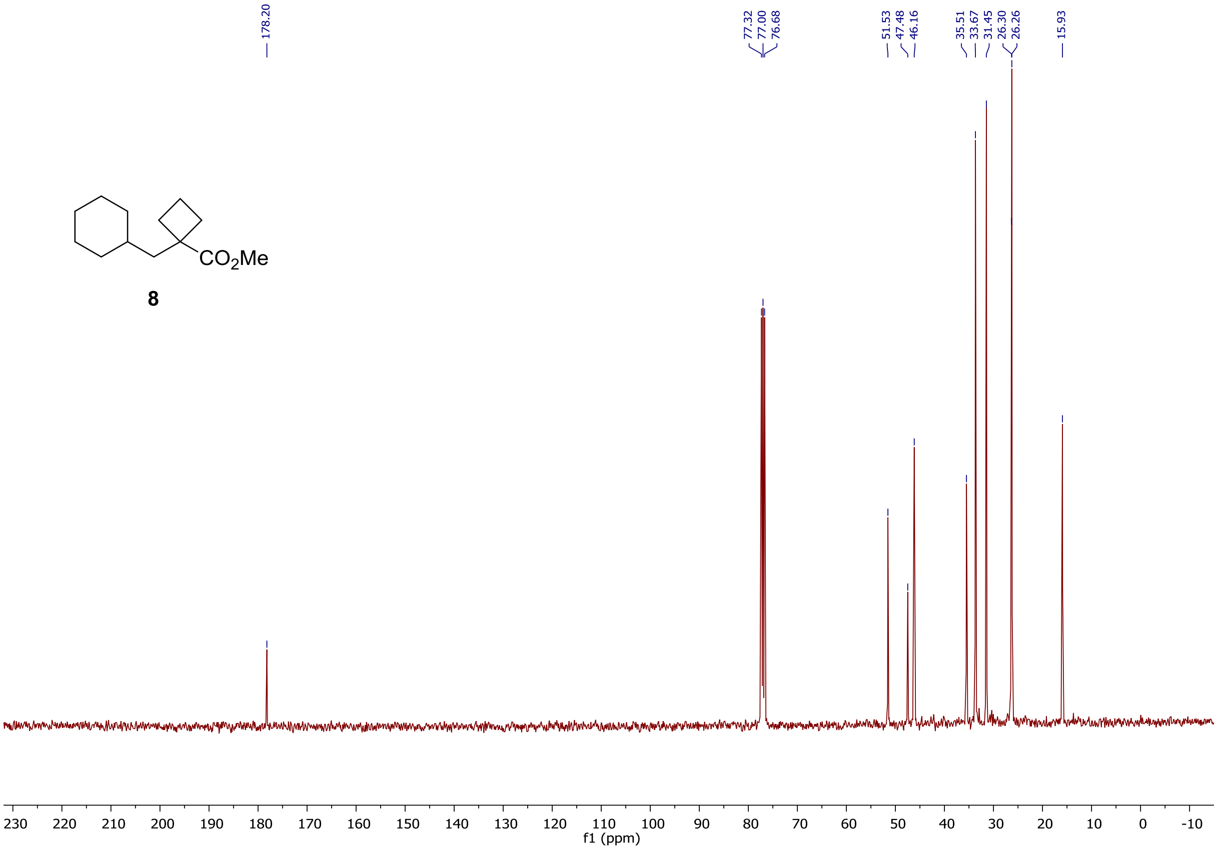

^{1}H NMR (400 MHz, $CDCl_3$)

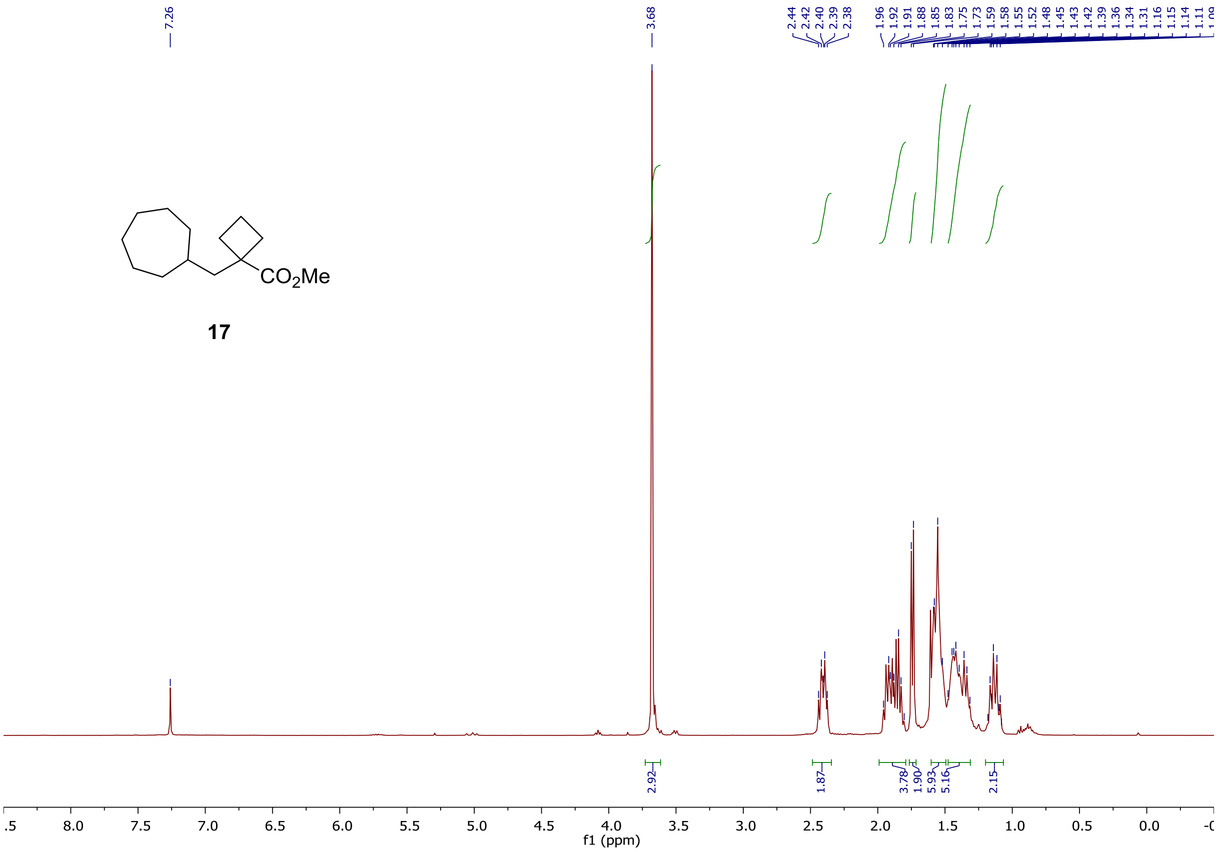

^{13}C NMR (101 MHz, $CDCl_3$)

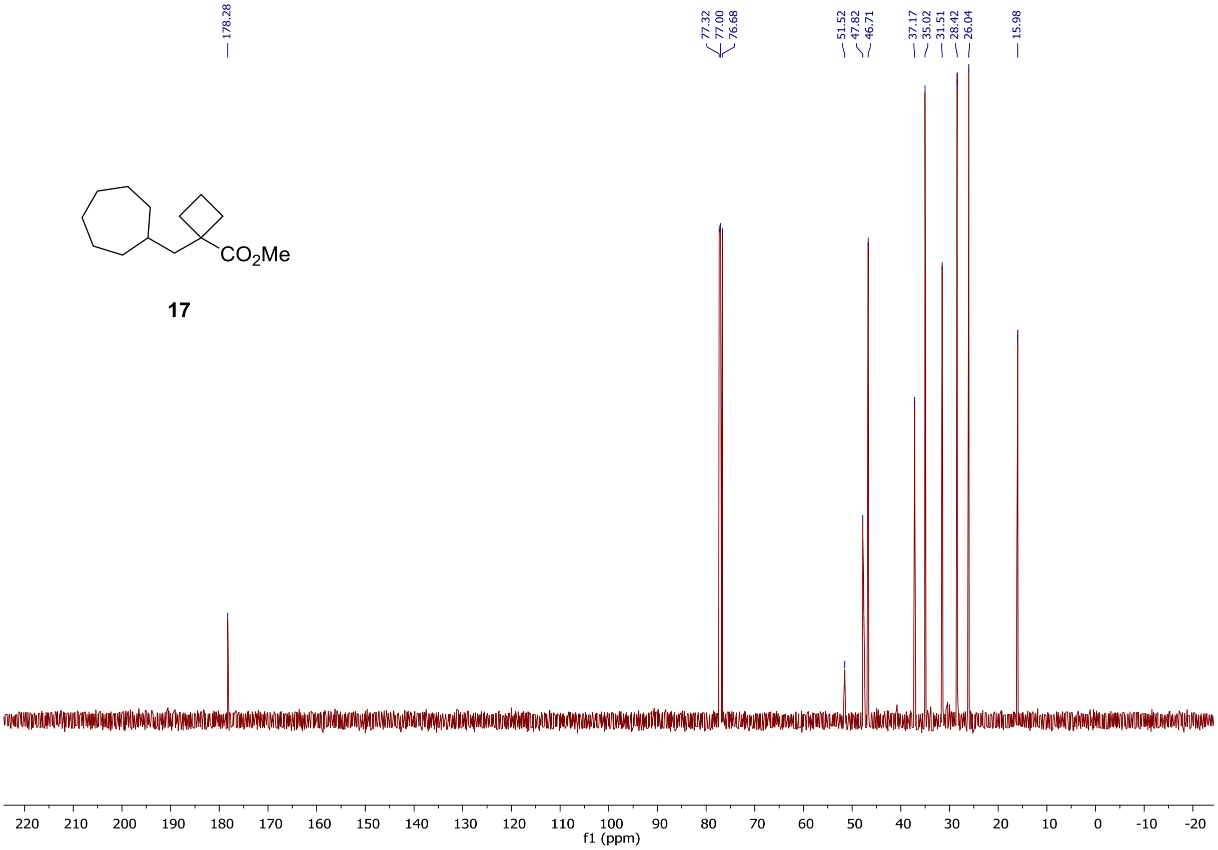

^{1}H NMR (400 MHz, $CDCl_3$)

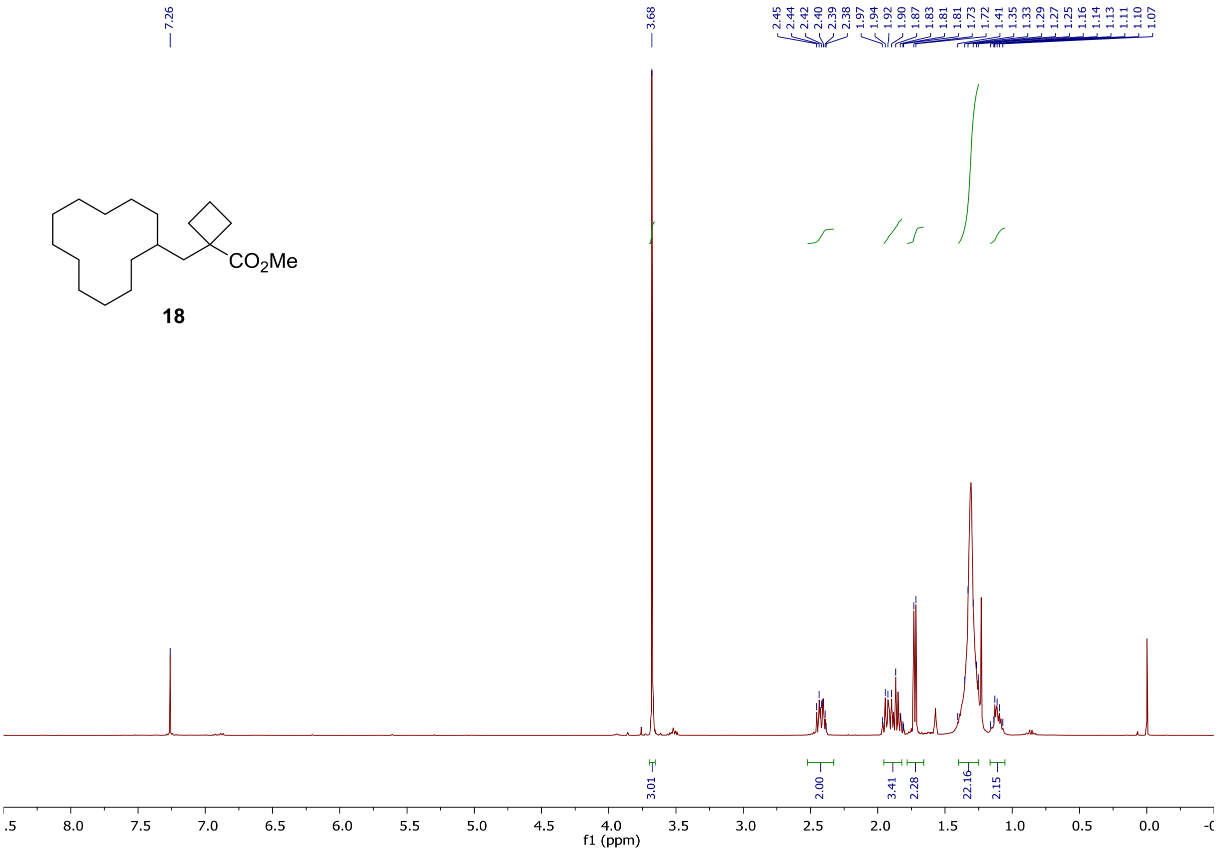

^{13}C NMR (101 MHz, $CDCl_3$)

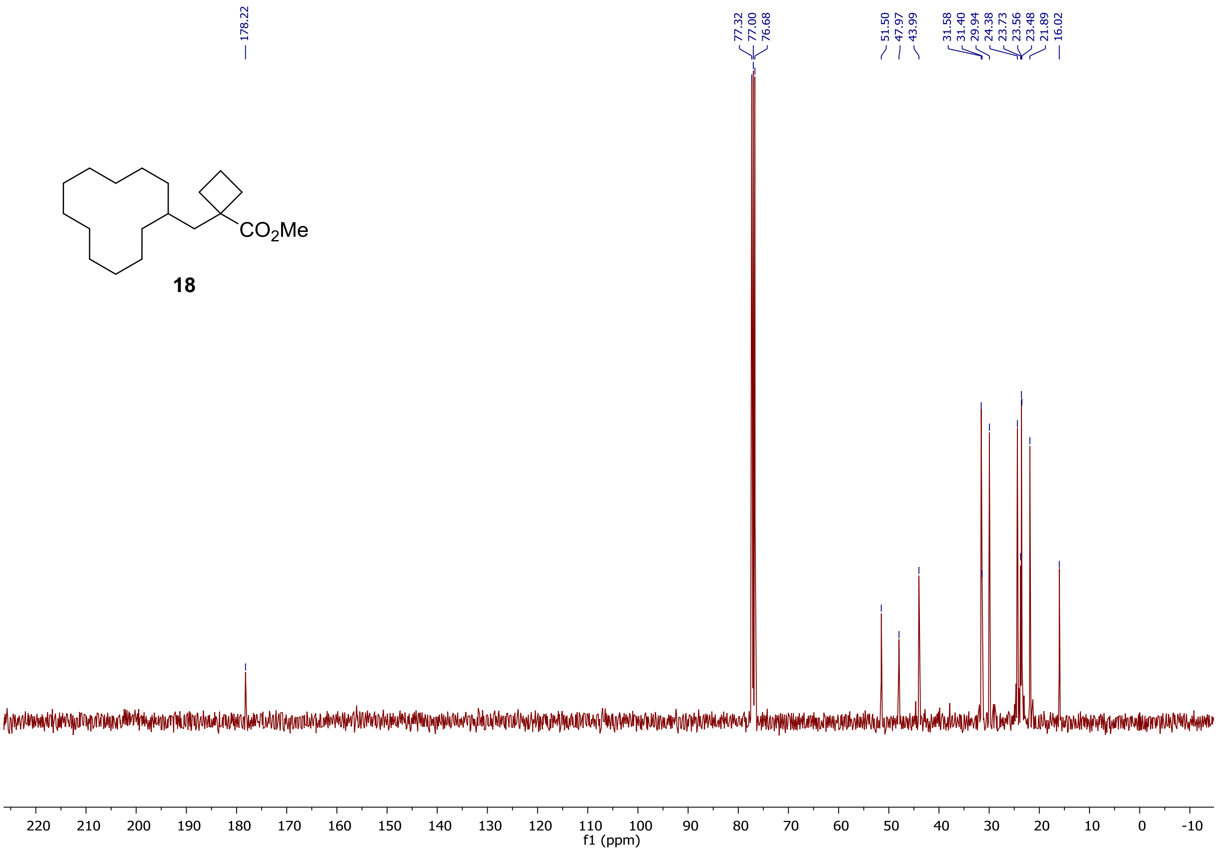

^{1}H NMR (400 MHz, $CDCl_3$)

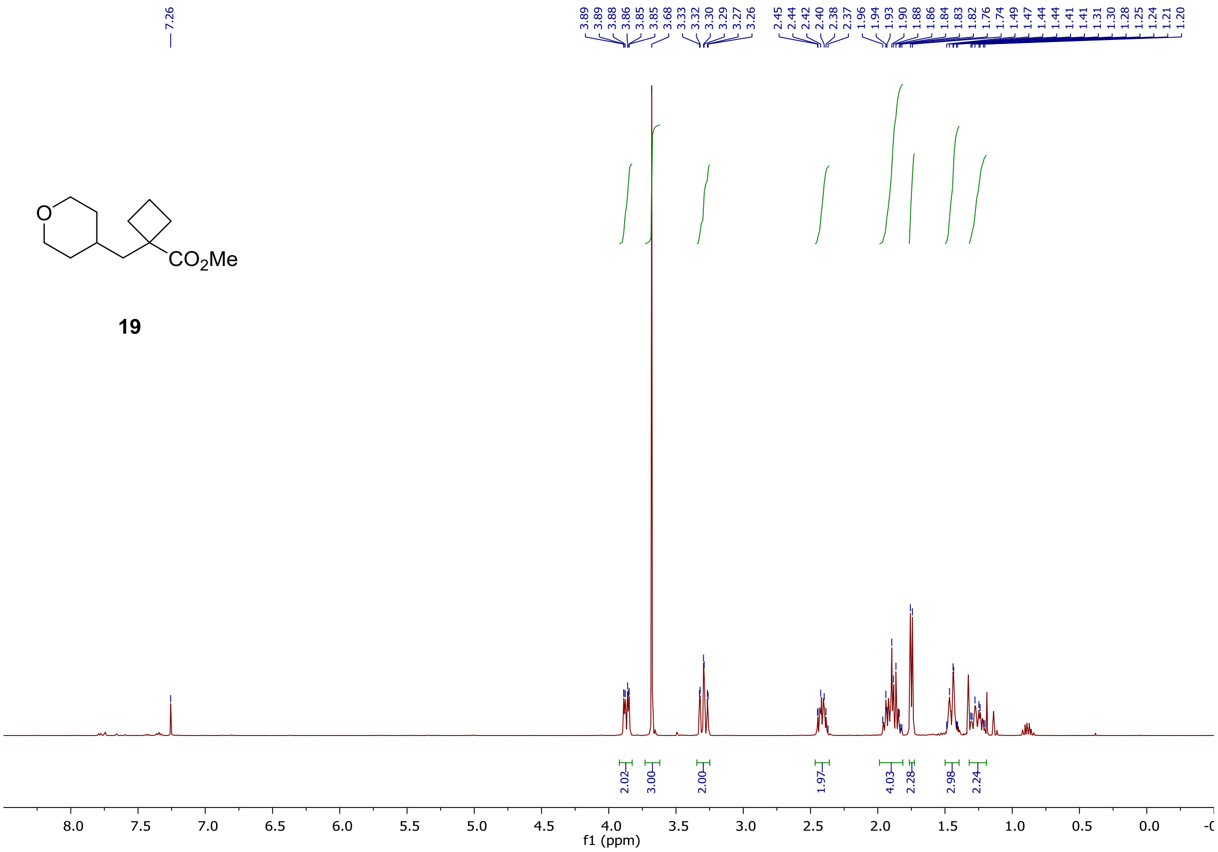

^{13}C NMR (101 MHz, $CDCl_3$)

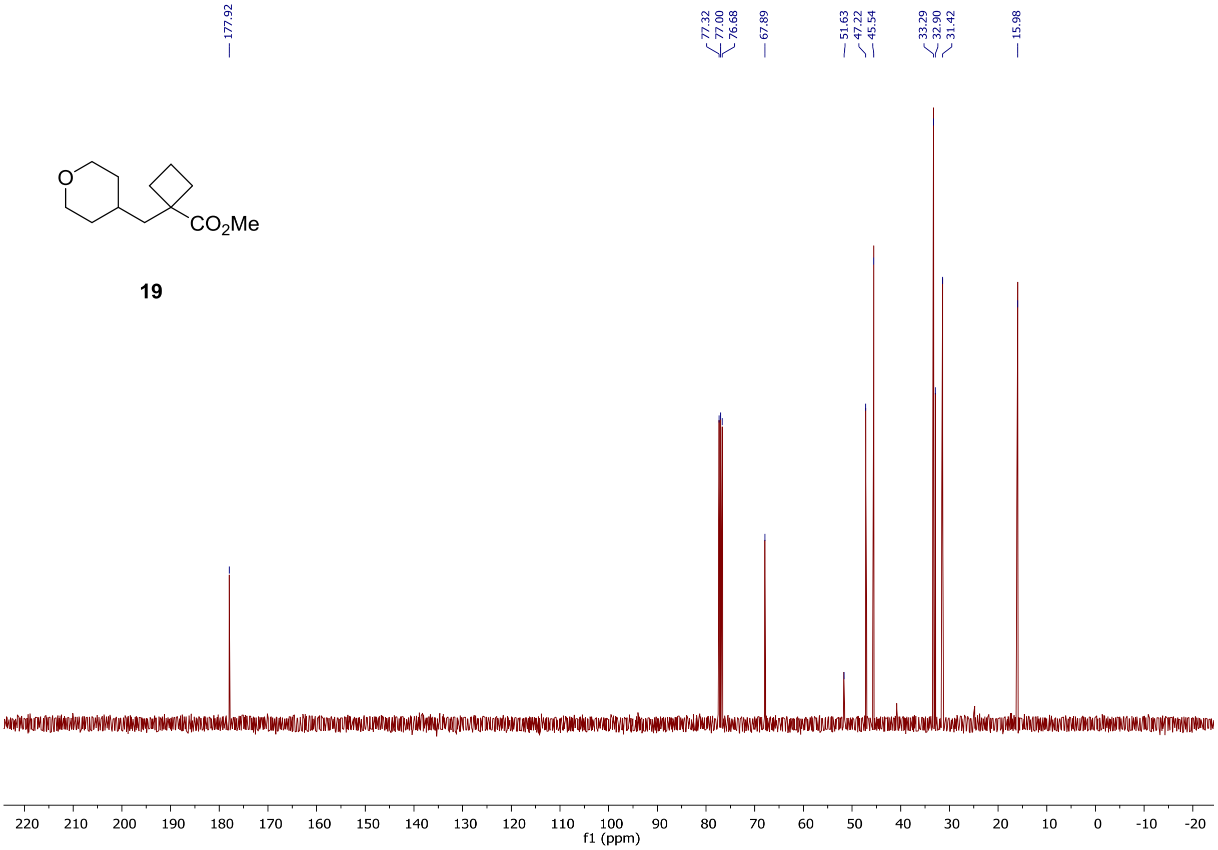

^{1}H NMR (400 MHz, $CDCl_3$)

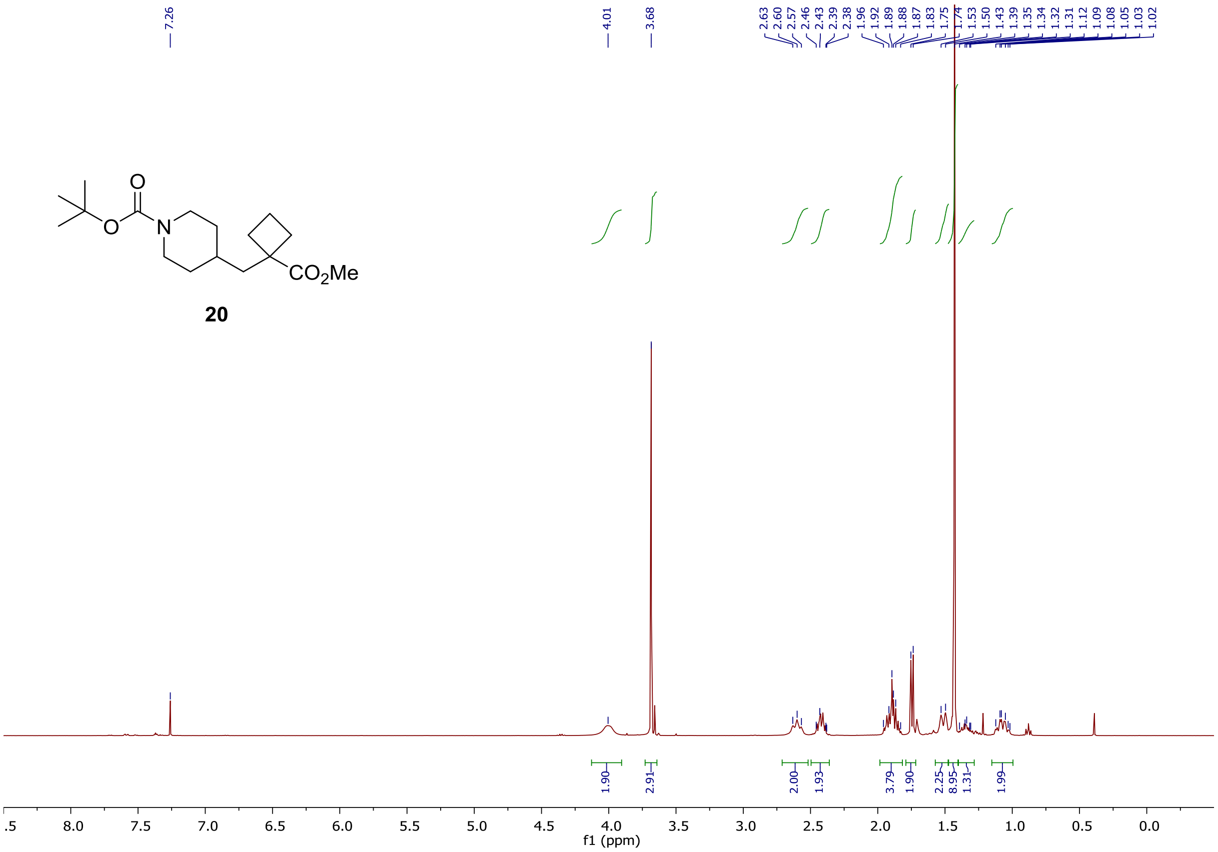

20

^{13}C NMR (101 MHz, $CDCl_3$)

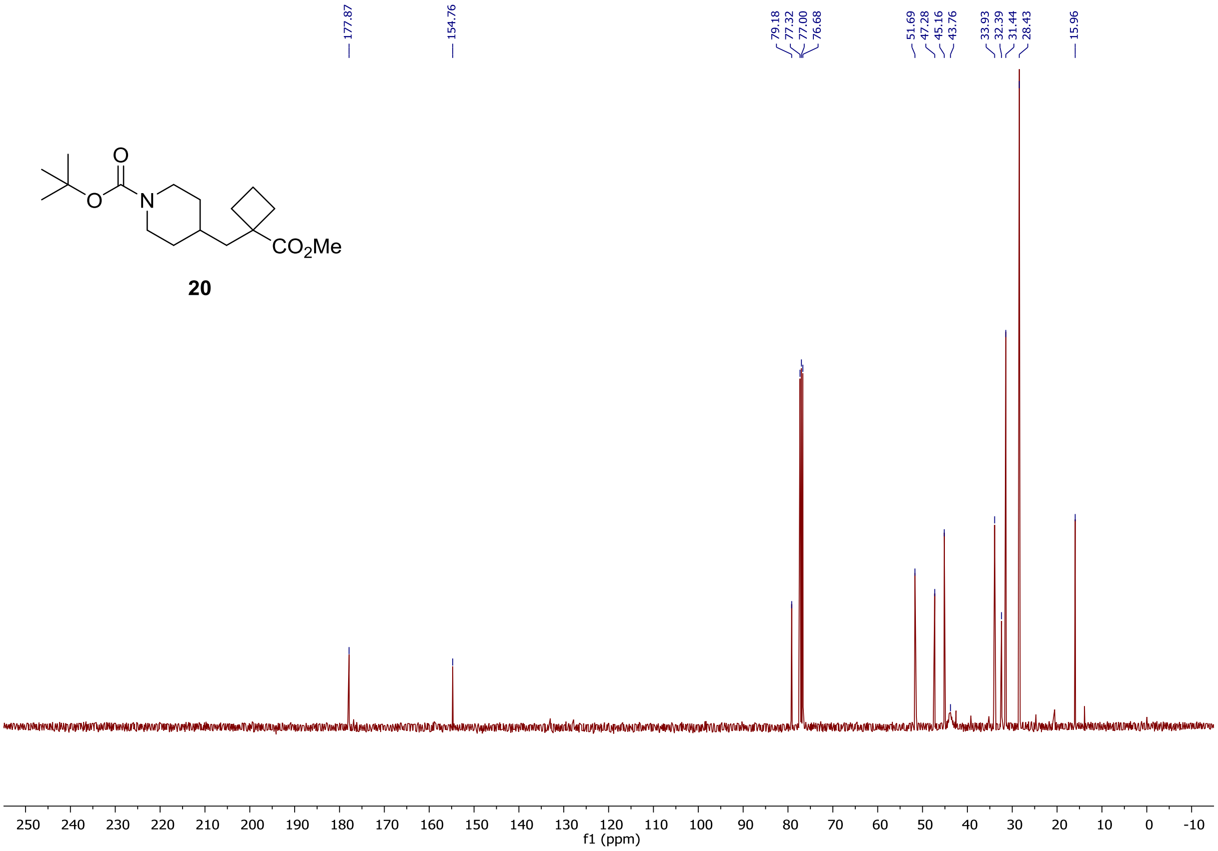

20

^{1}H NMR (400 MHz, $CDCl_3$)

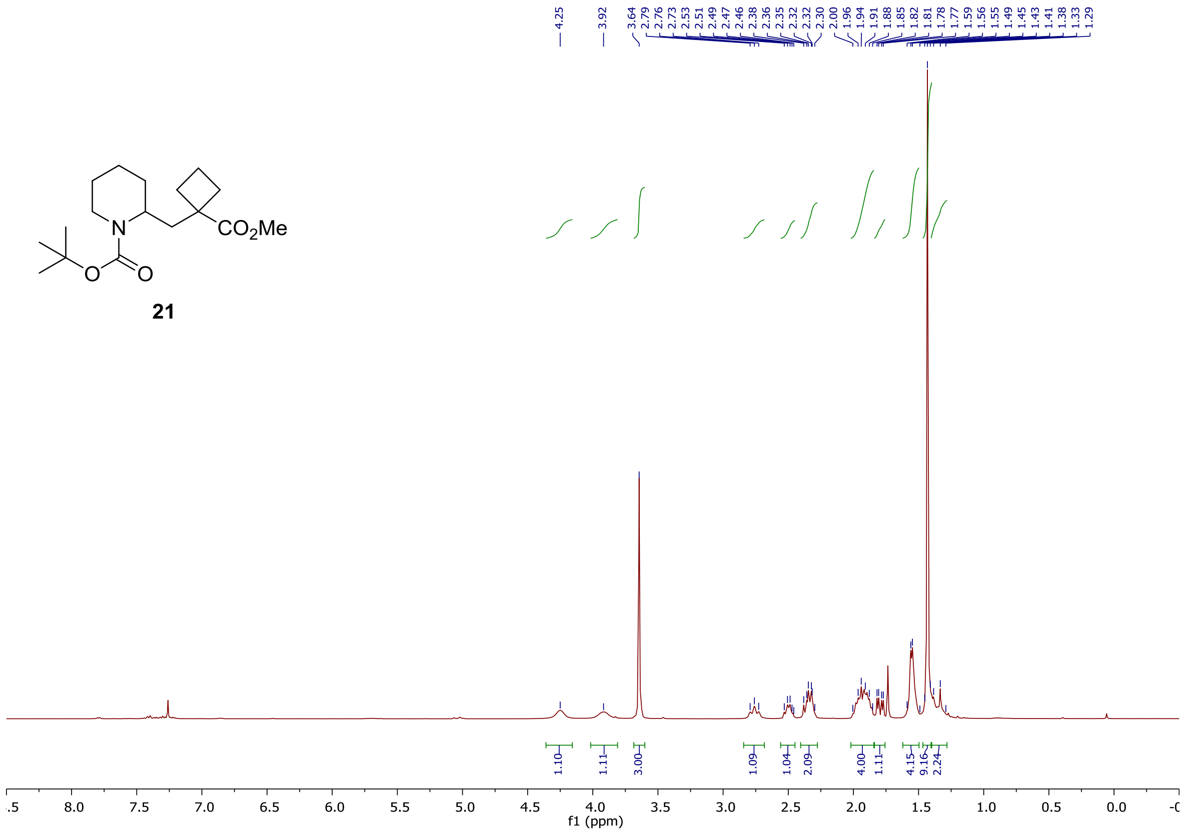

^{13}C NMR (101 MHz, $CDCl_3$)

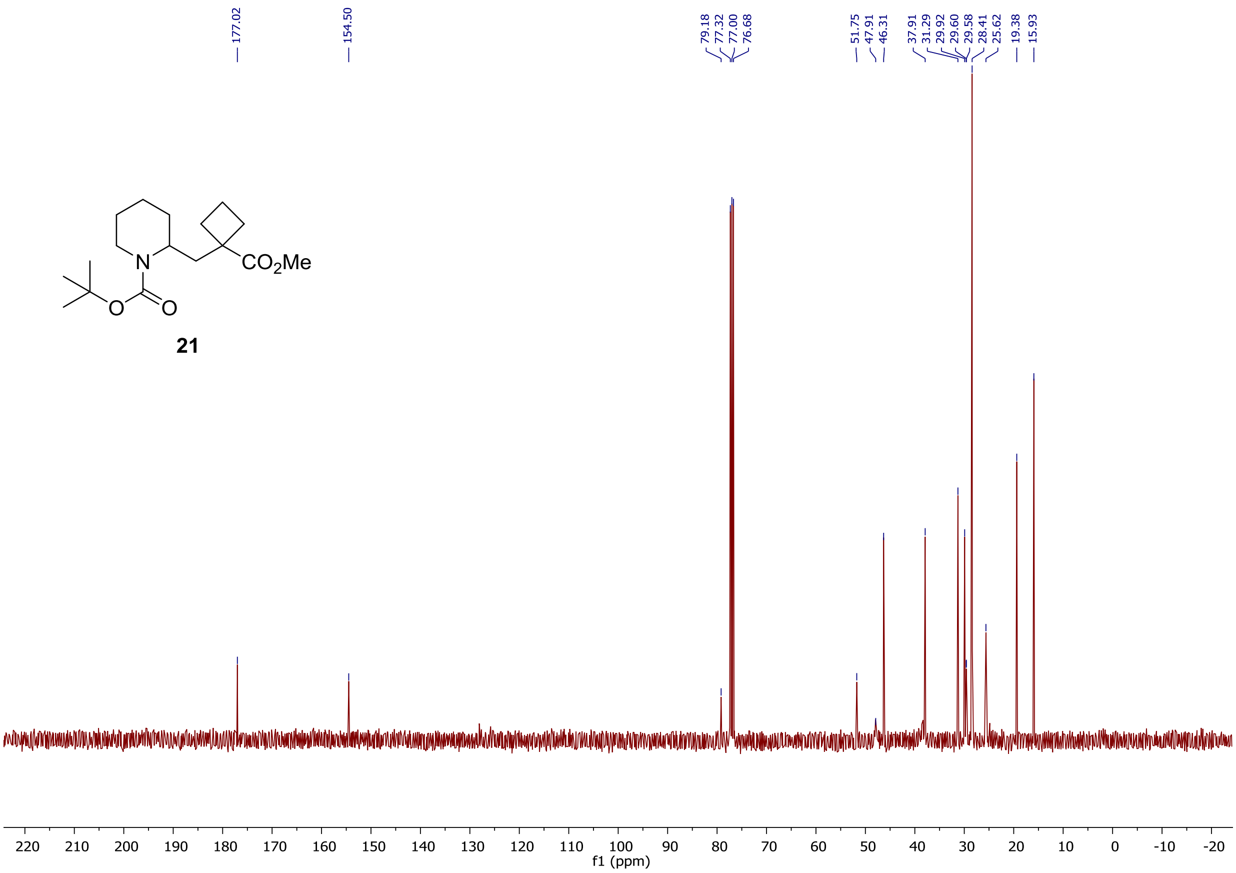

^{1}H NMR (400 MHz, $CDCl_3$)

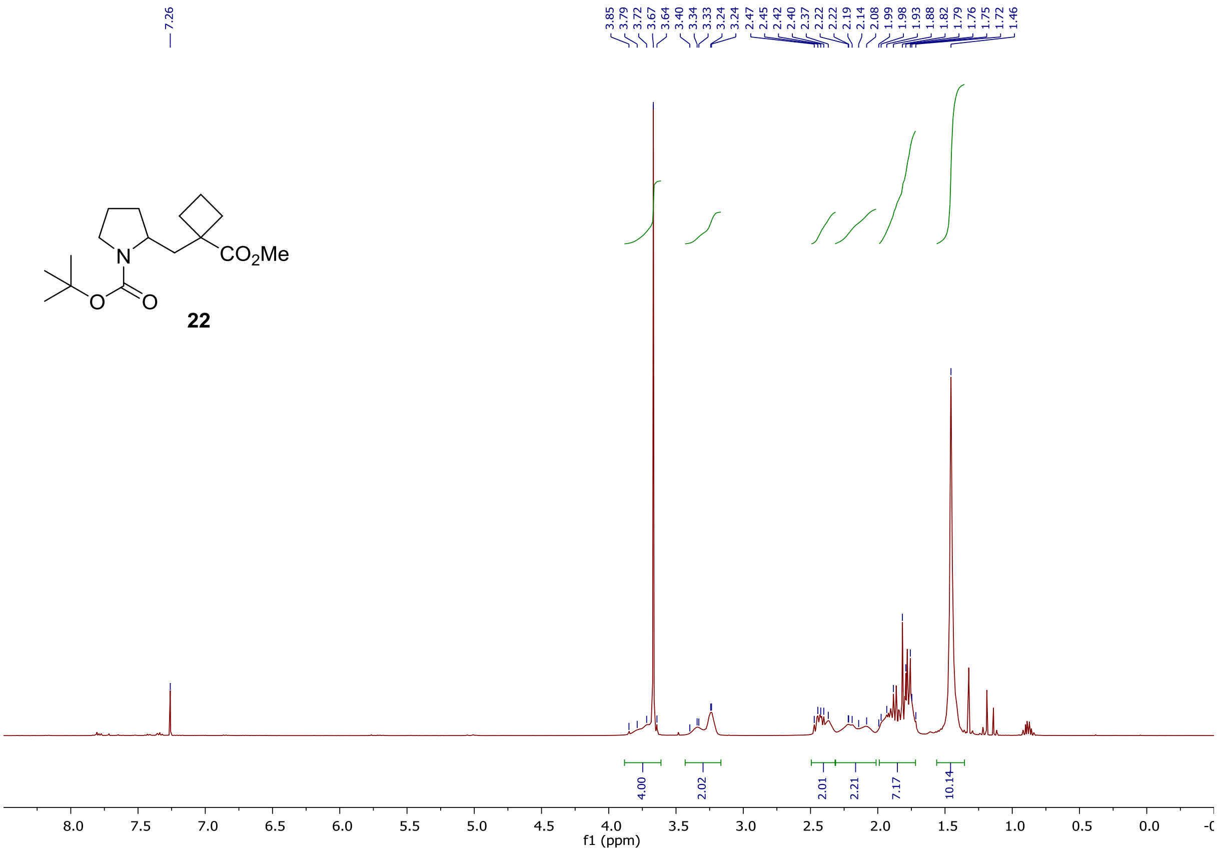

^{13}C NMR (101 MHz, $CDCl_3$)

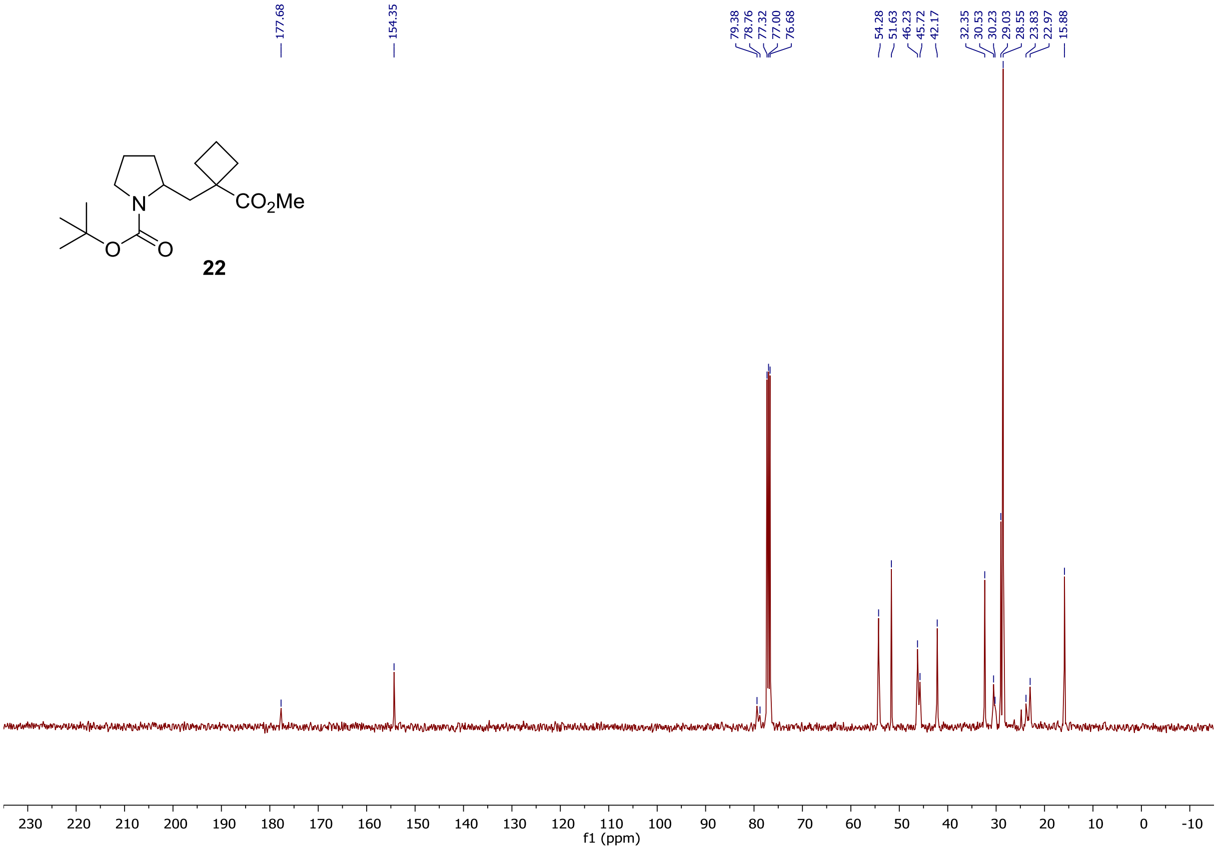

^{1}H NMR (400 MHz, $CDCl_3$)

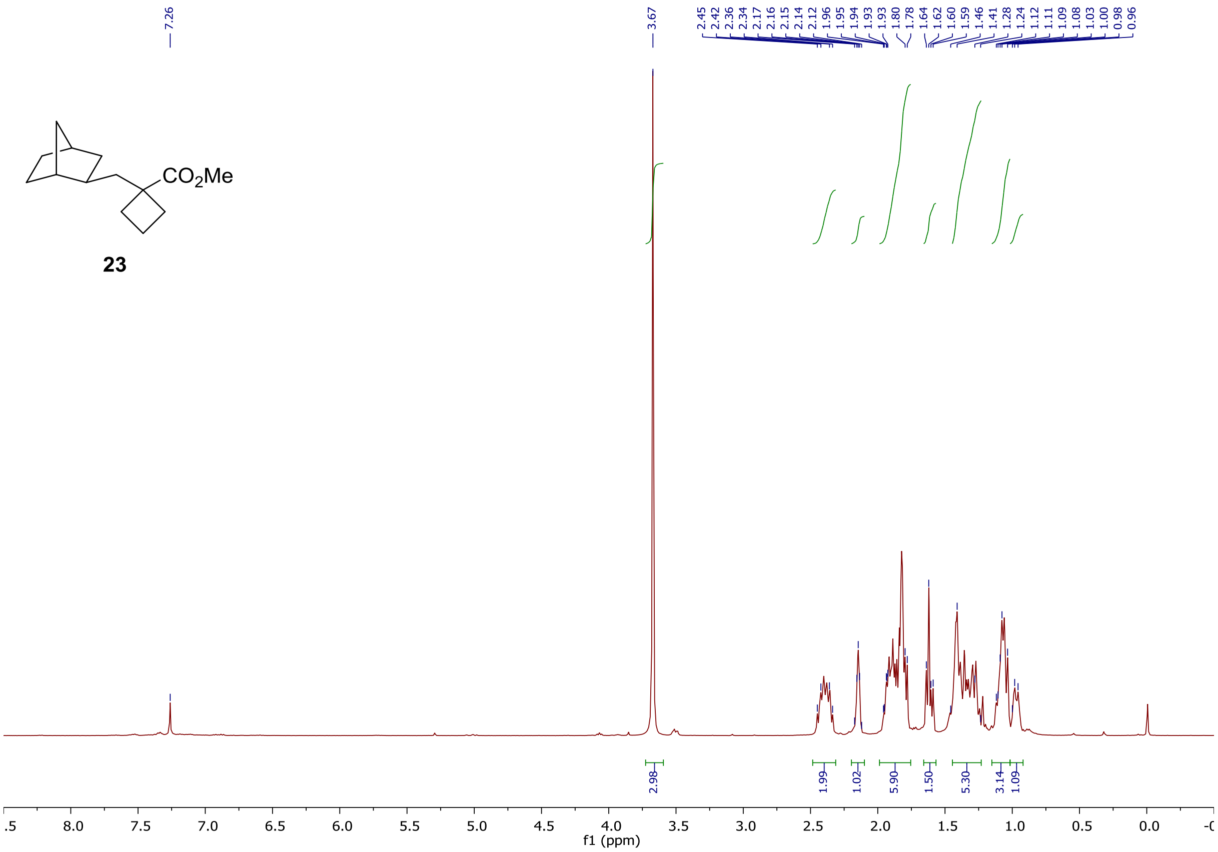

^{13}C NMR (101 MHz, $CDCl_3$)

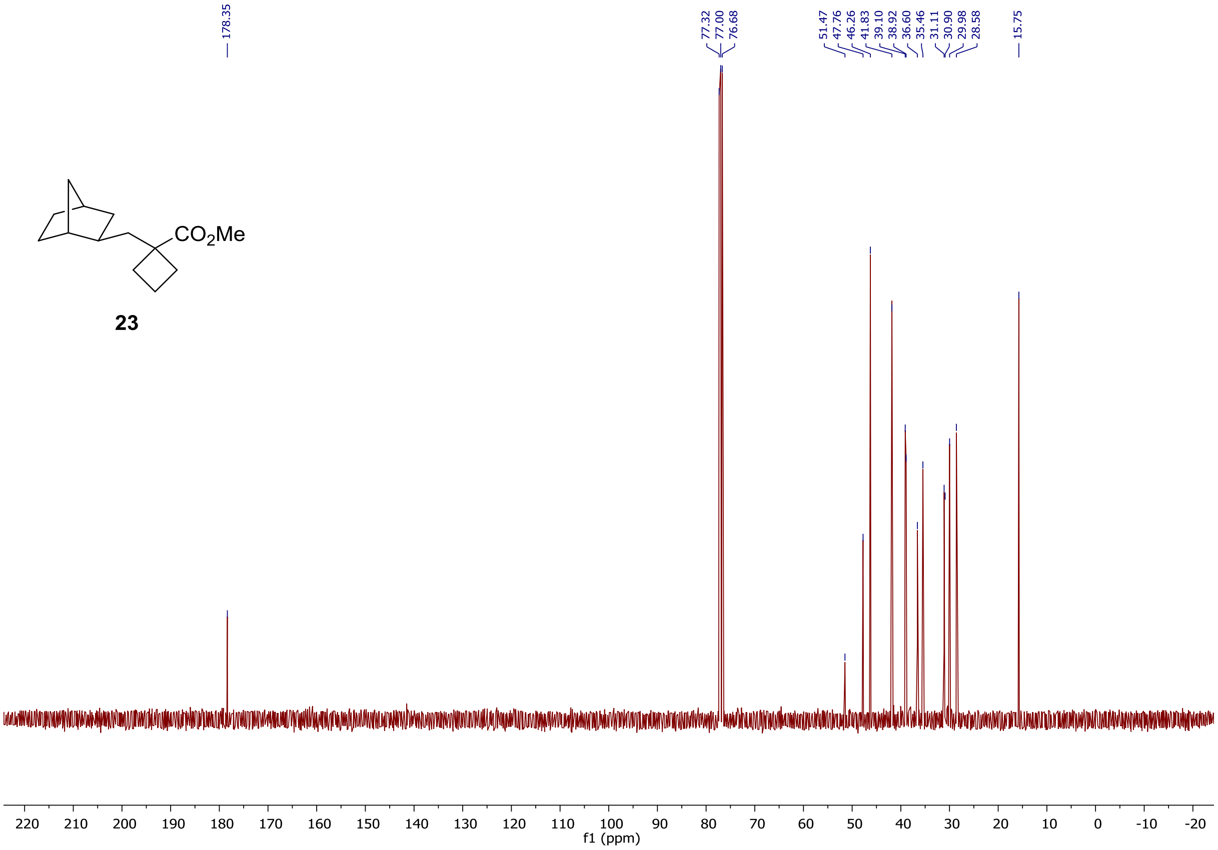

^{1}H NMR (400 MHz, $CDCl_3$)

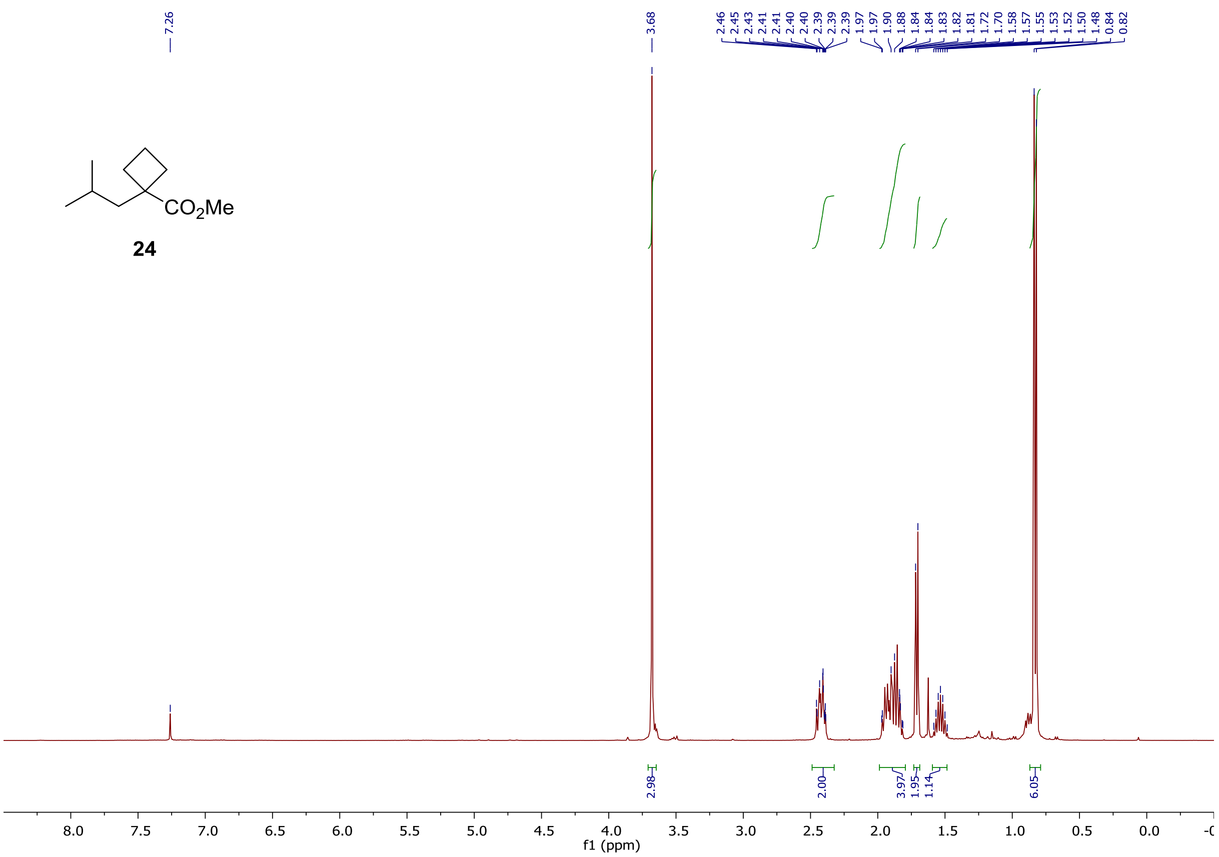

^{13}C NMR (101 MHz, $CDCl_3$)

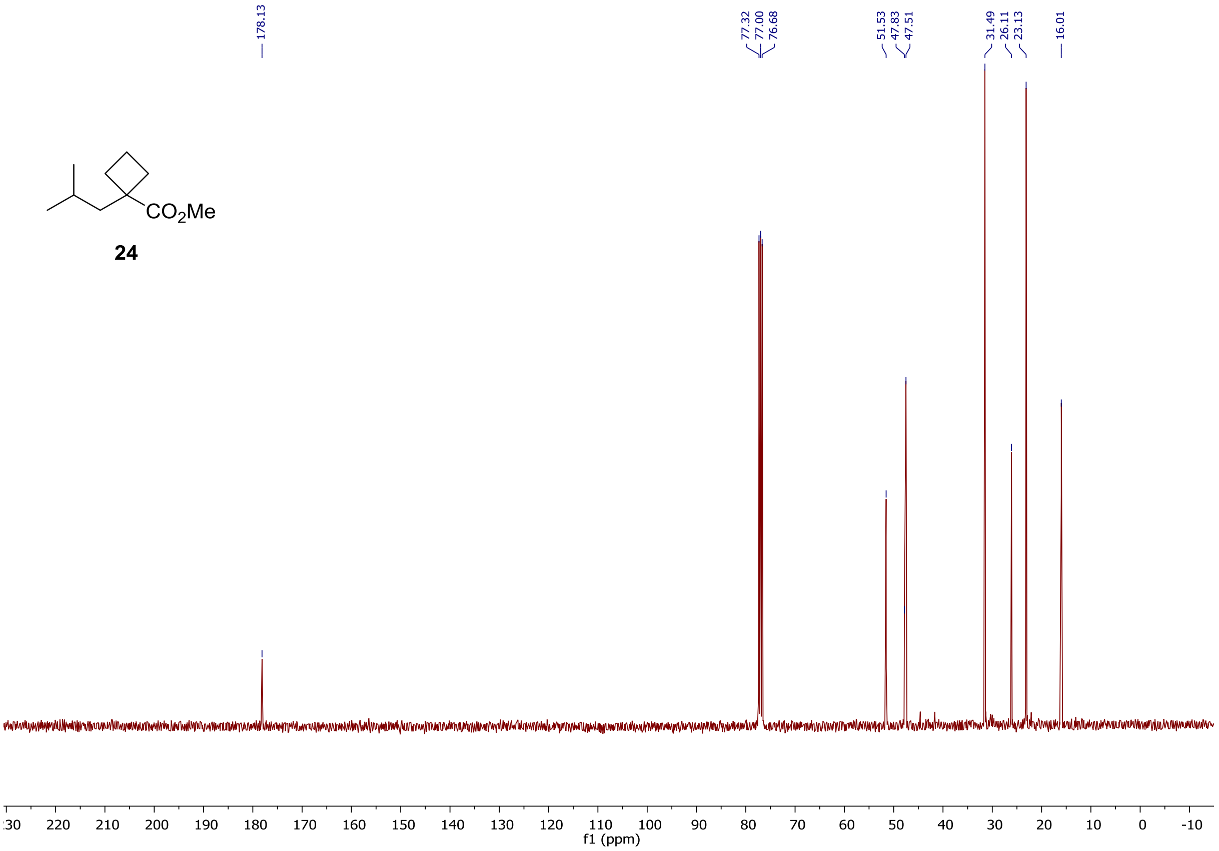

^{1}H NMR (400 MHz, $CDCl_3$)

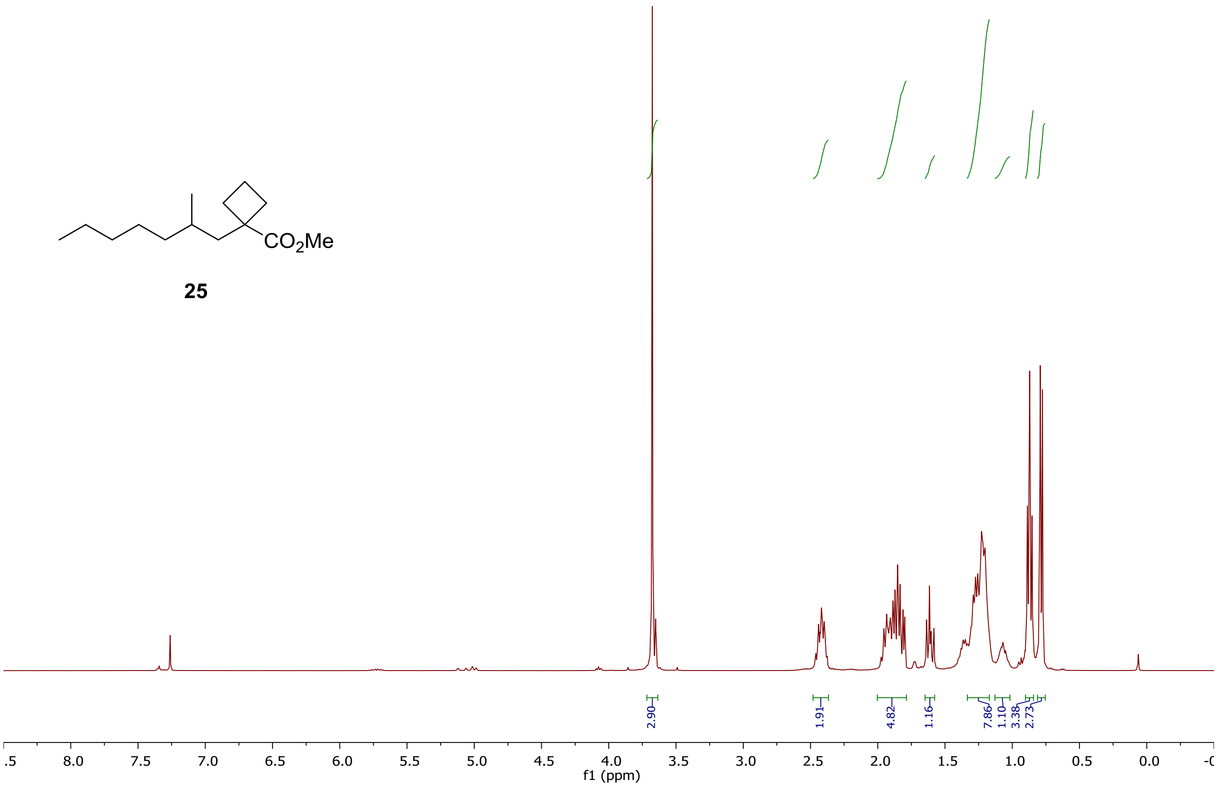

^{13}C NMR (101 MHz, $CDCl_3$)

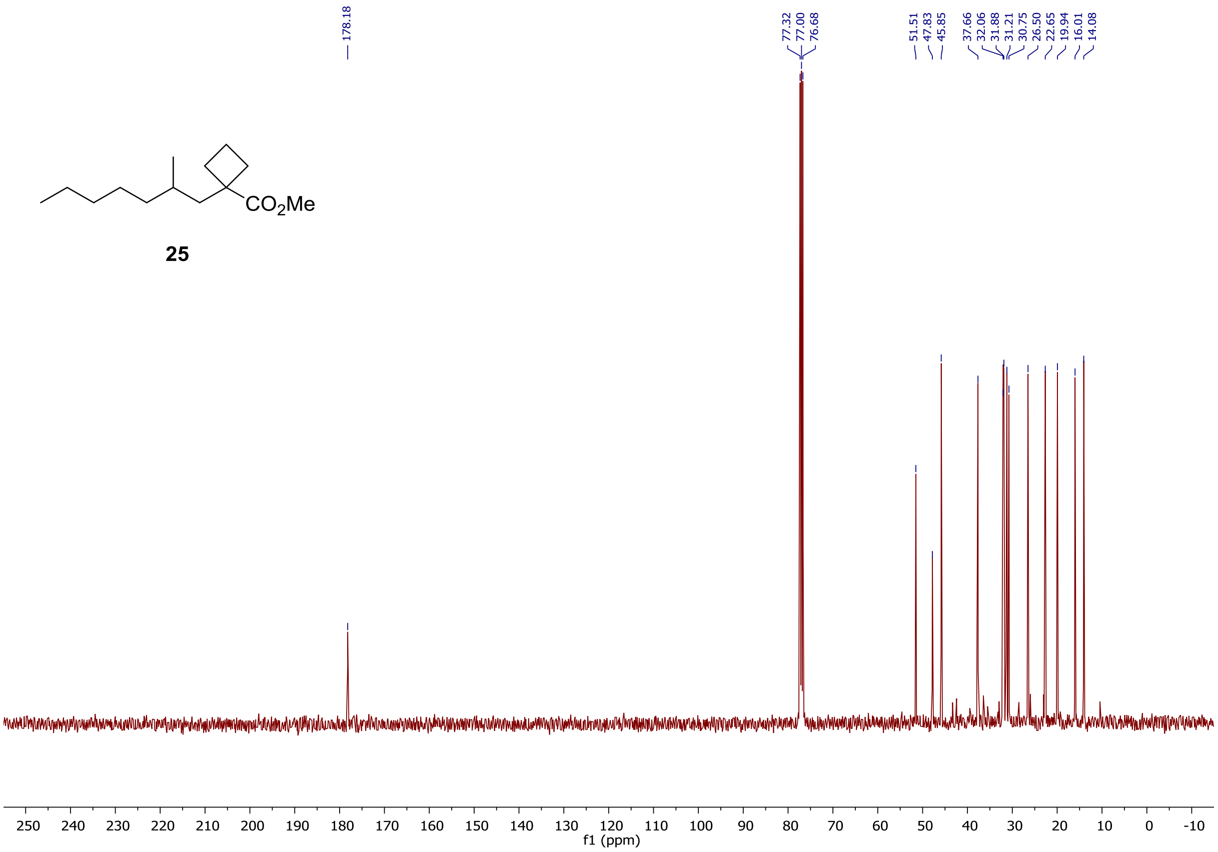

^{1}H NMR (400 MHz, $CDCl_3$)

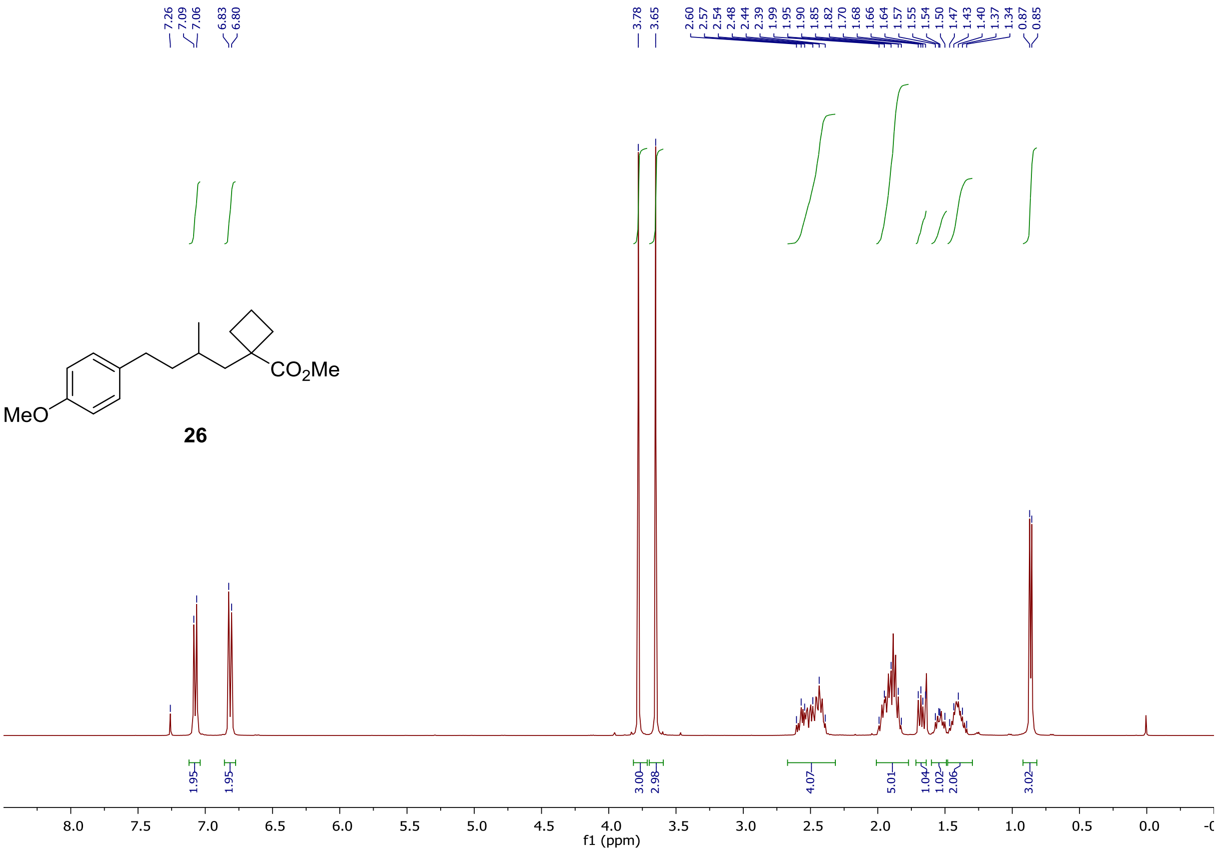

^{13}C NMR (101 MHz, $CDCl_3$)

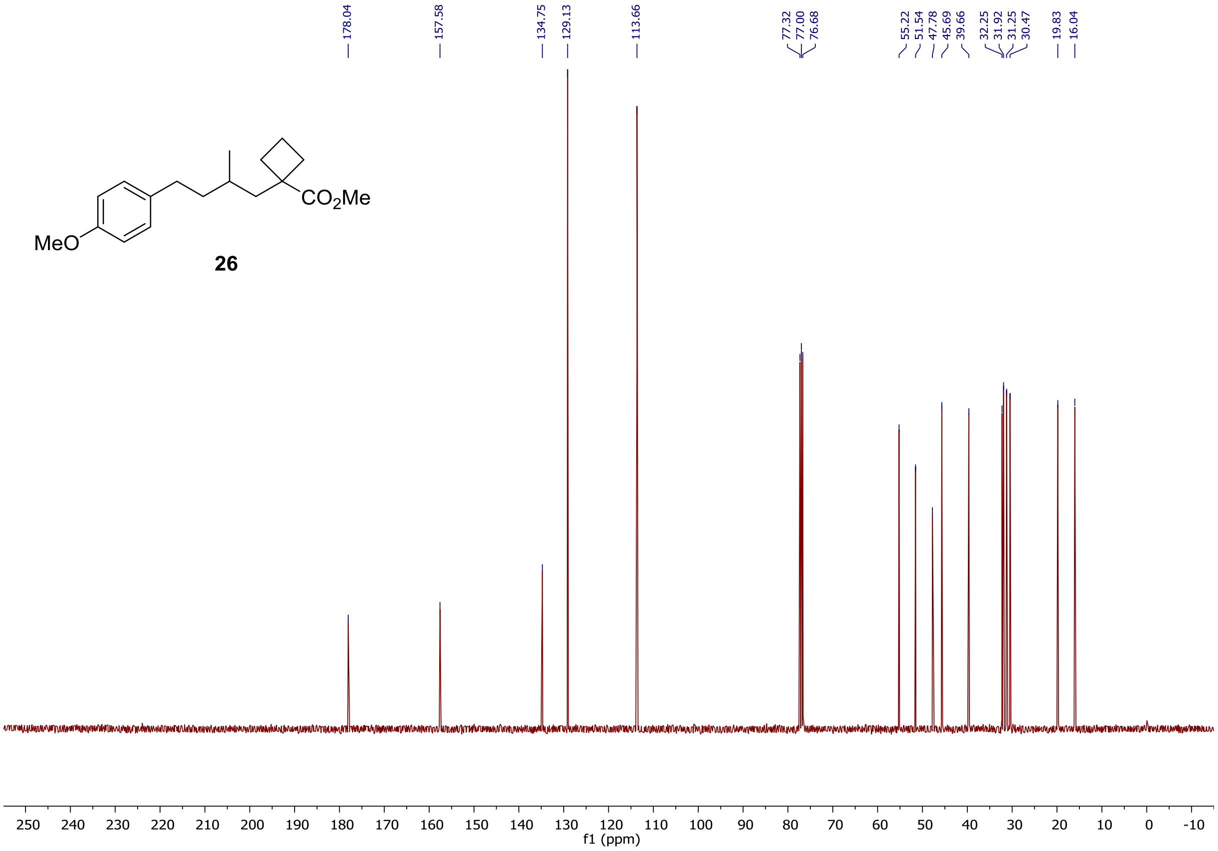

^{1}H NMR (400 MHz, $CDCl_3$)

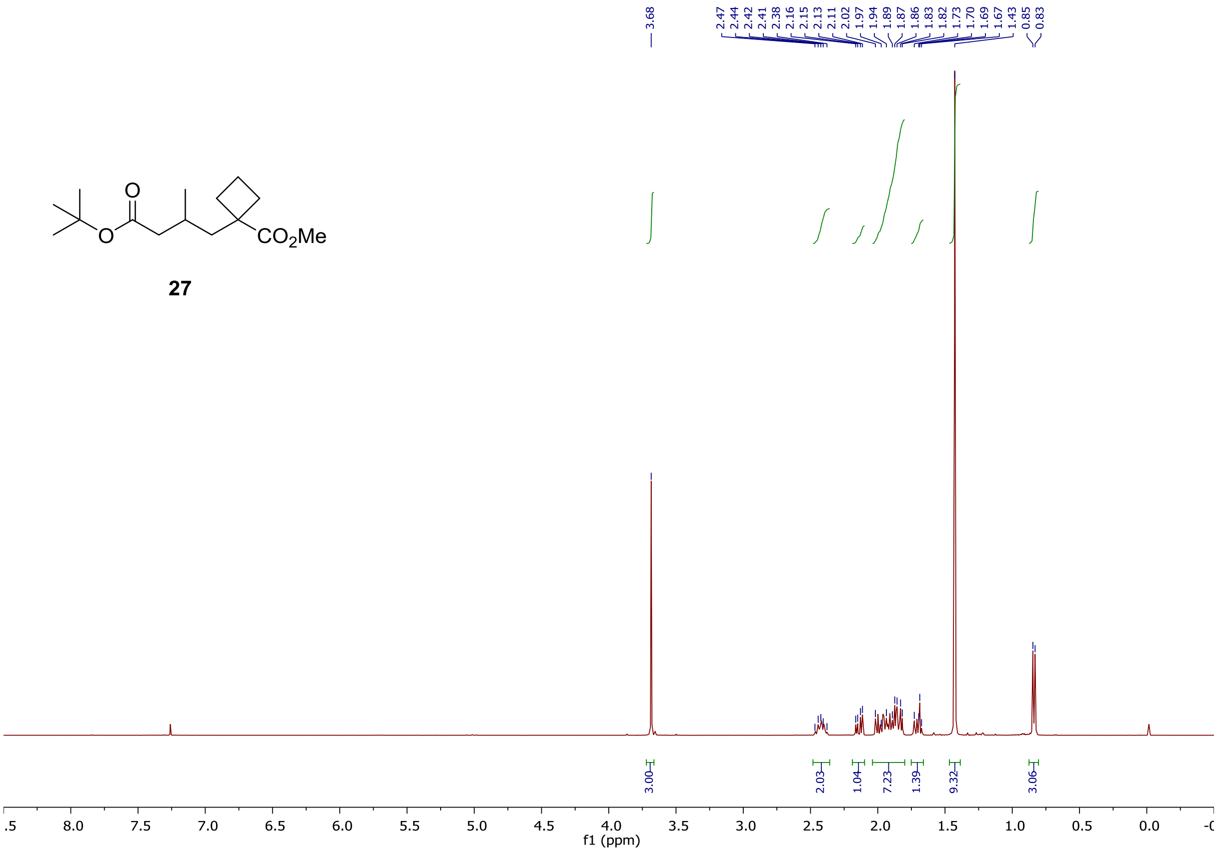

^{13}C NMR (101 MHz, $CDCl_3$)

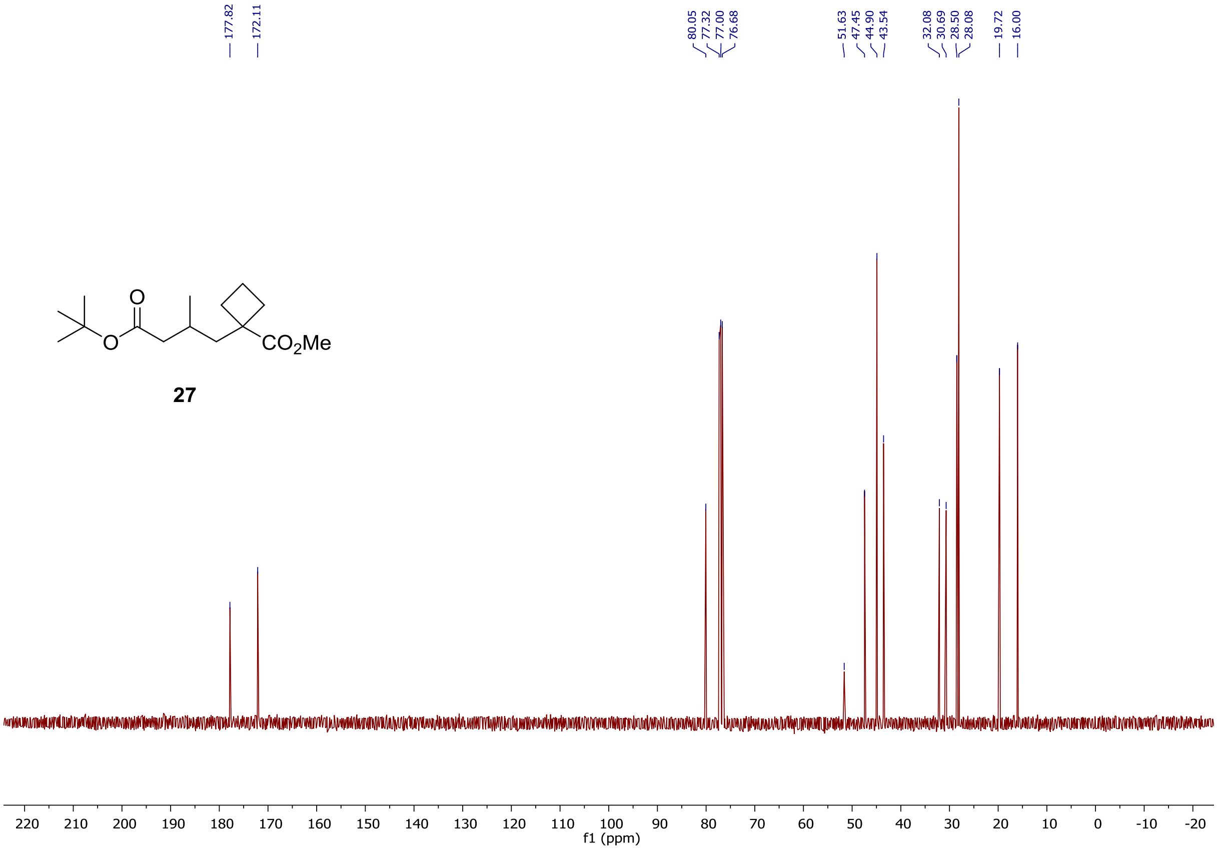

^{1}H NMR (400 MHz, $CDCl_3$)

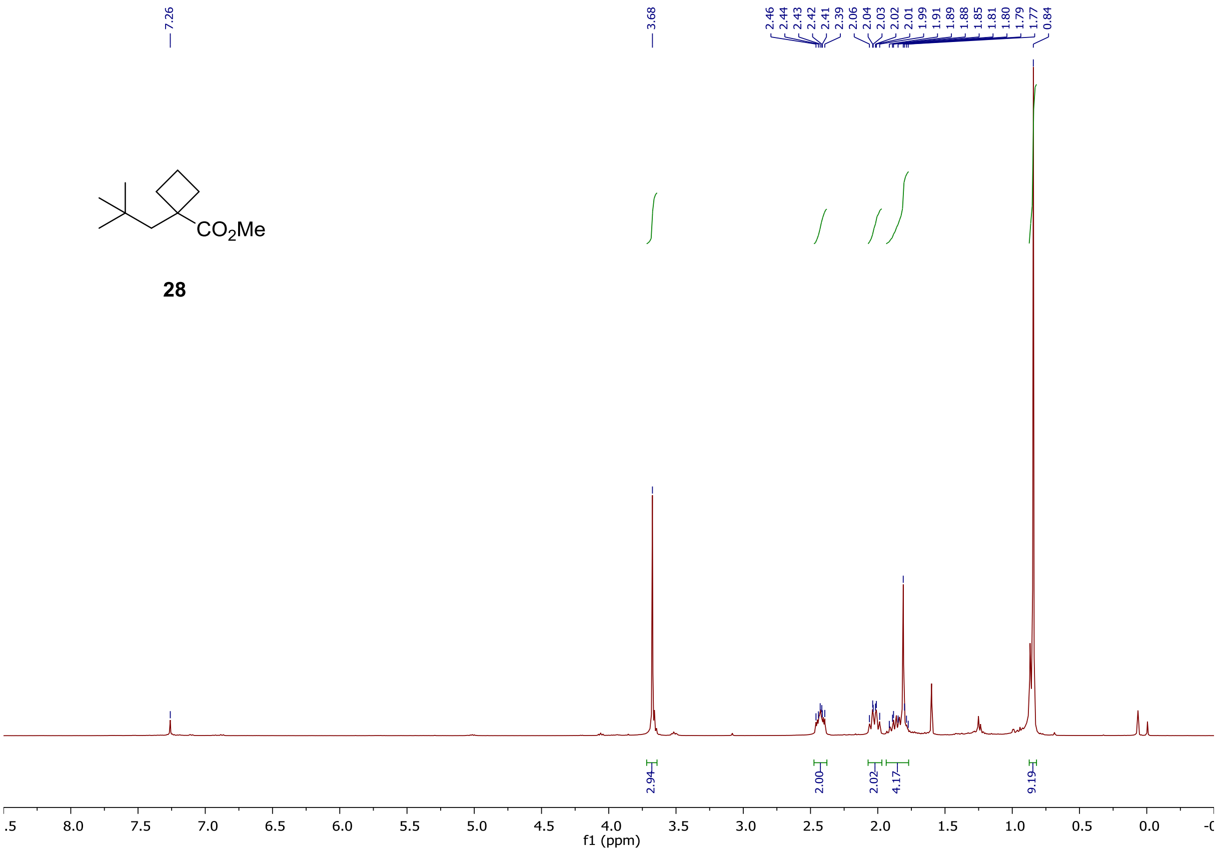

^{13}C NMR (101 MHz, $CDCl_3$)

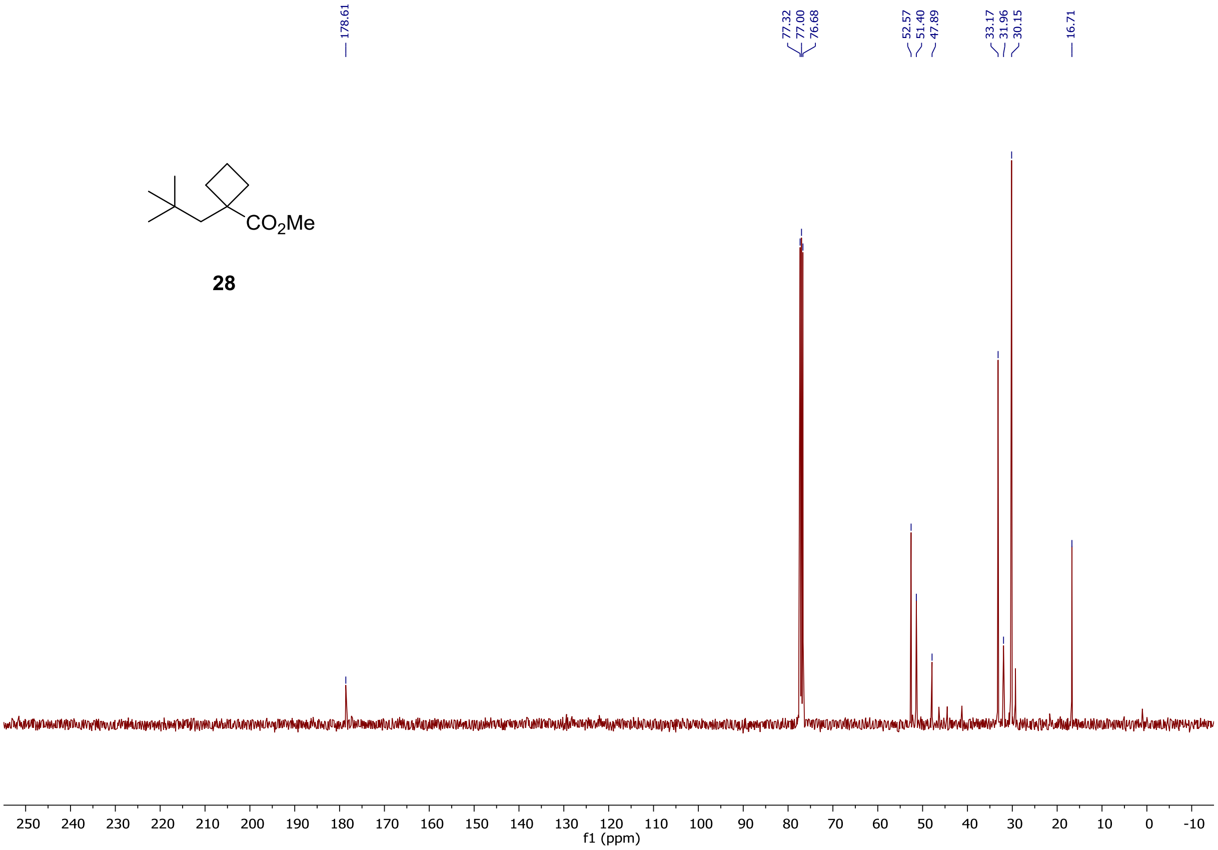

^{1}H NMR (400 MHz, $CDCl_3$)

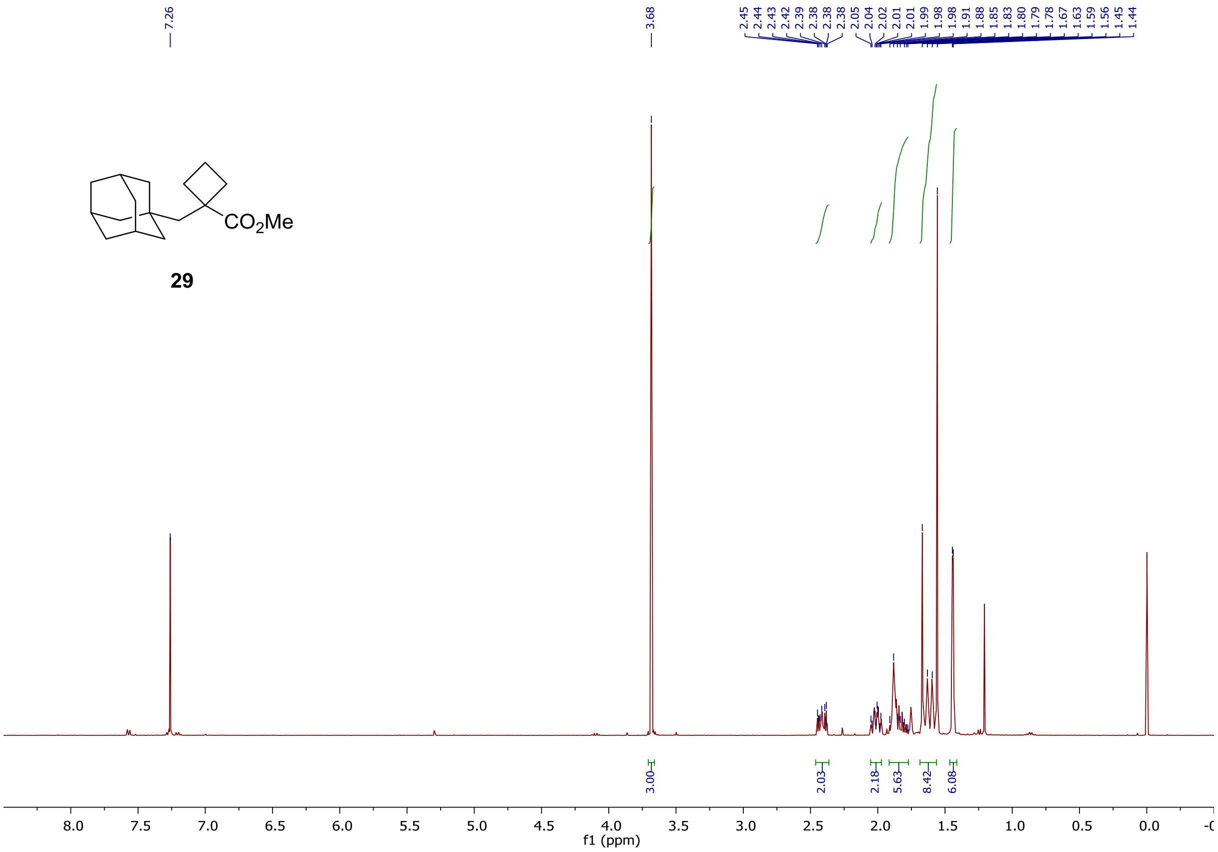

^{13}C NMR (101 MHz, $CDCl_3$)

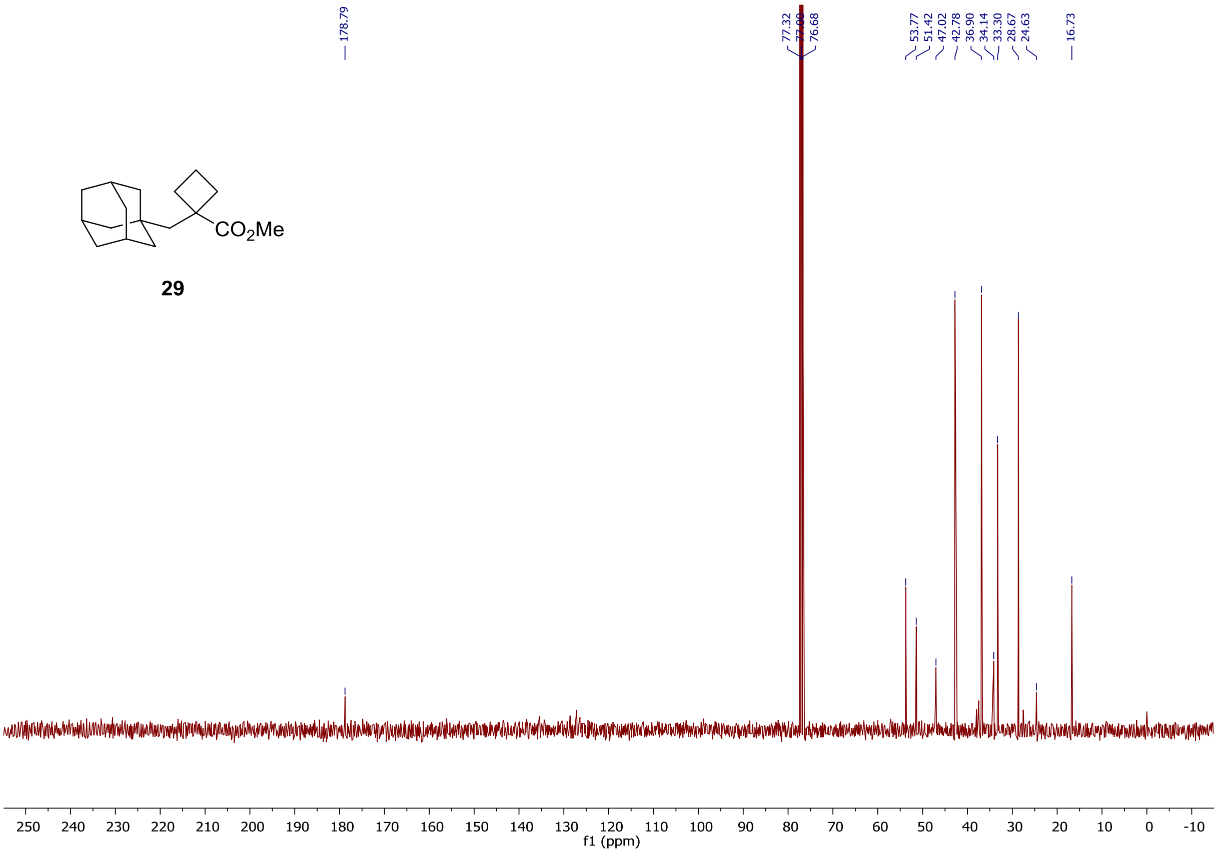

^{1}H NMR (400 MHz, $CDCl_3$)

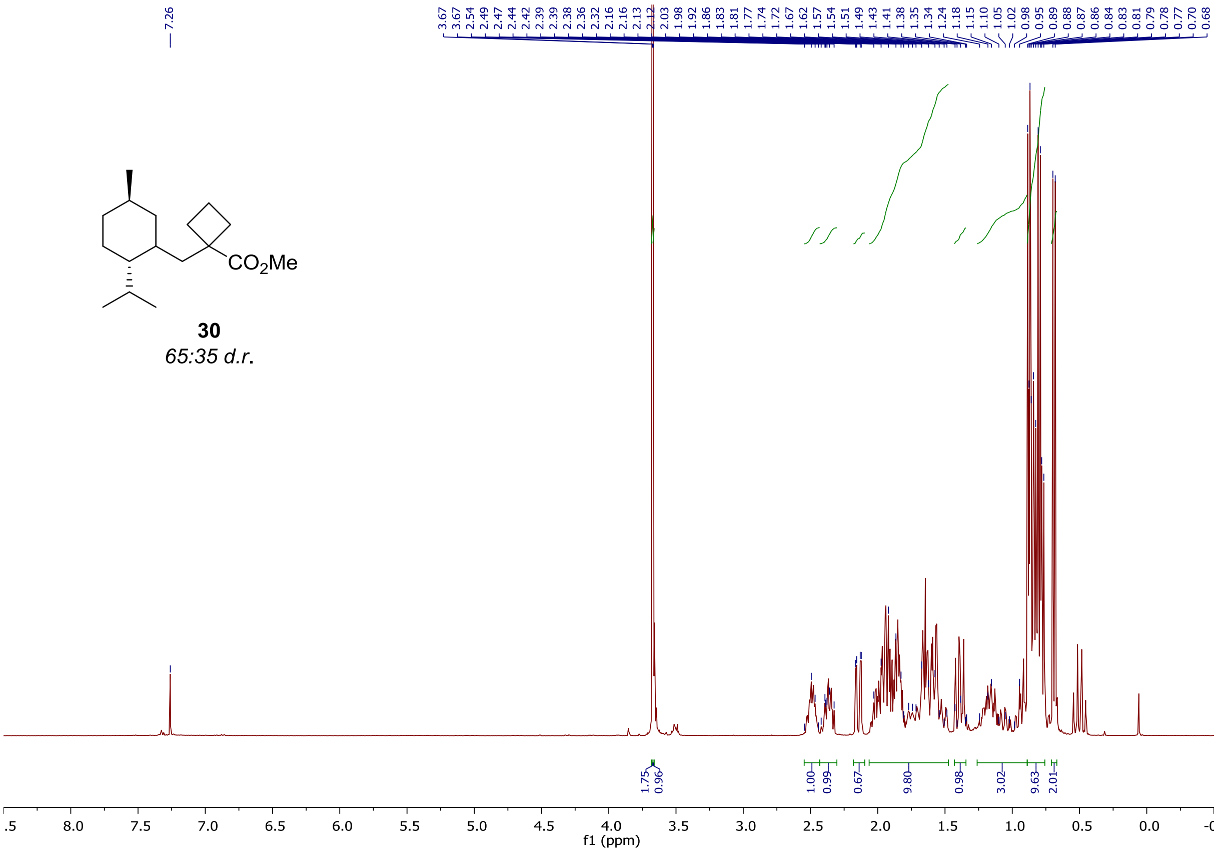

^{13}C NMR (101 MHz, $CDCl_3$)

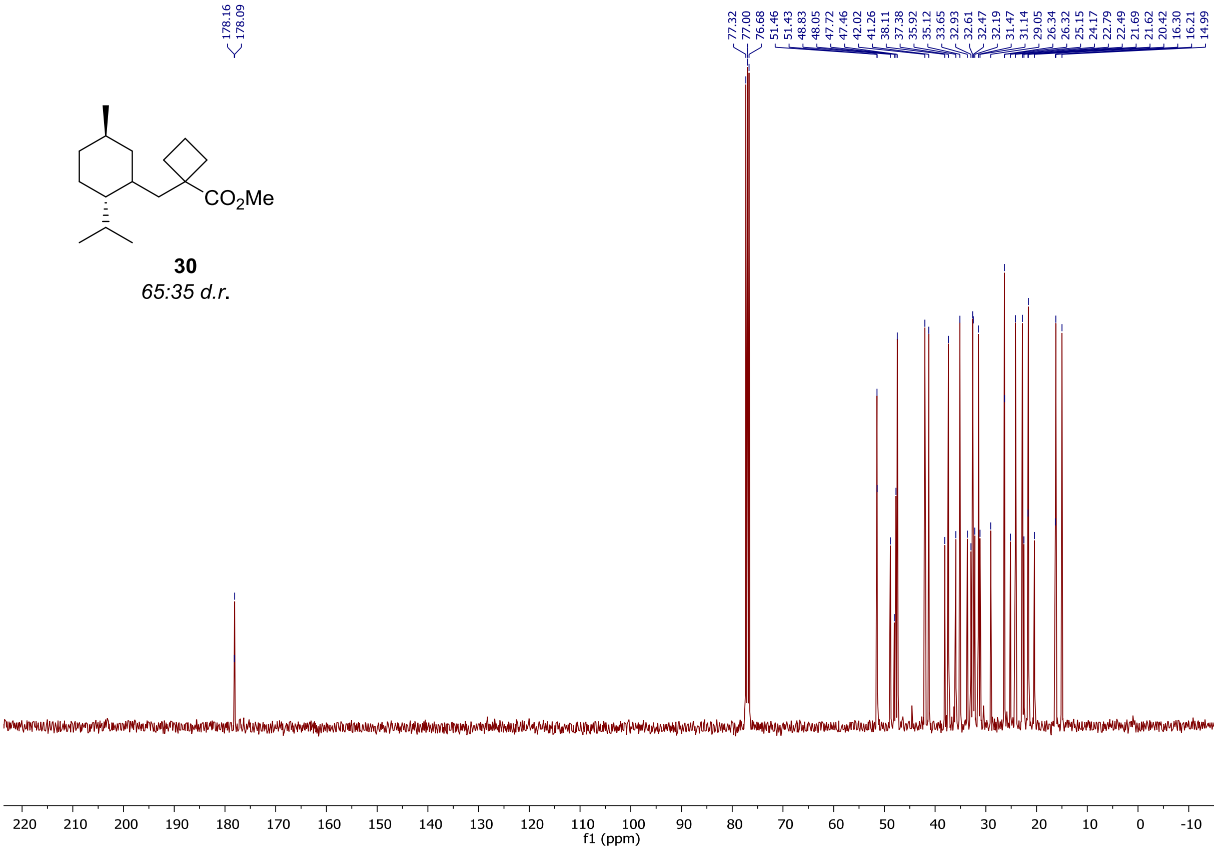

^{1}H NMR (400 MHz, $CDCl_3$)

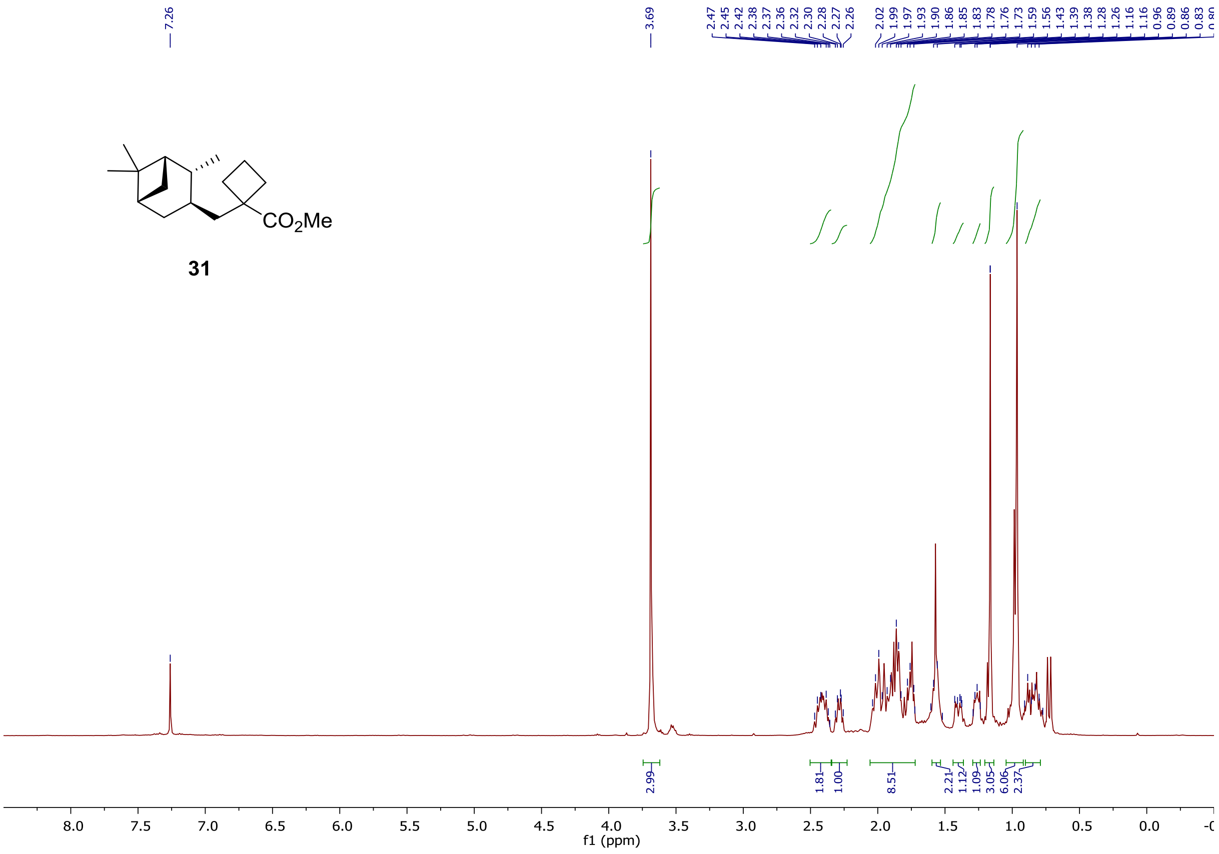

i

^{13}C NMR (101 MHz, $CDCl_3$)

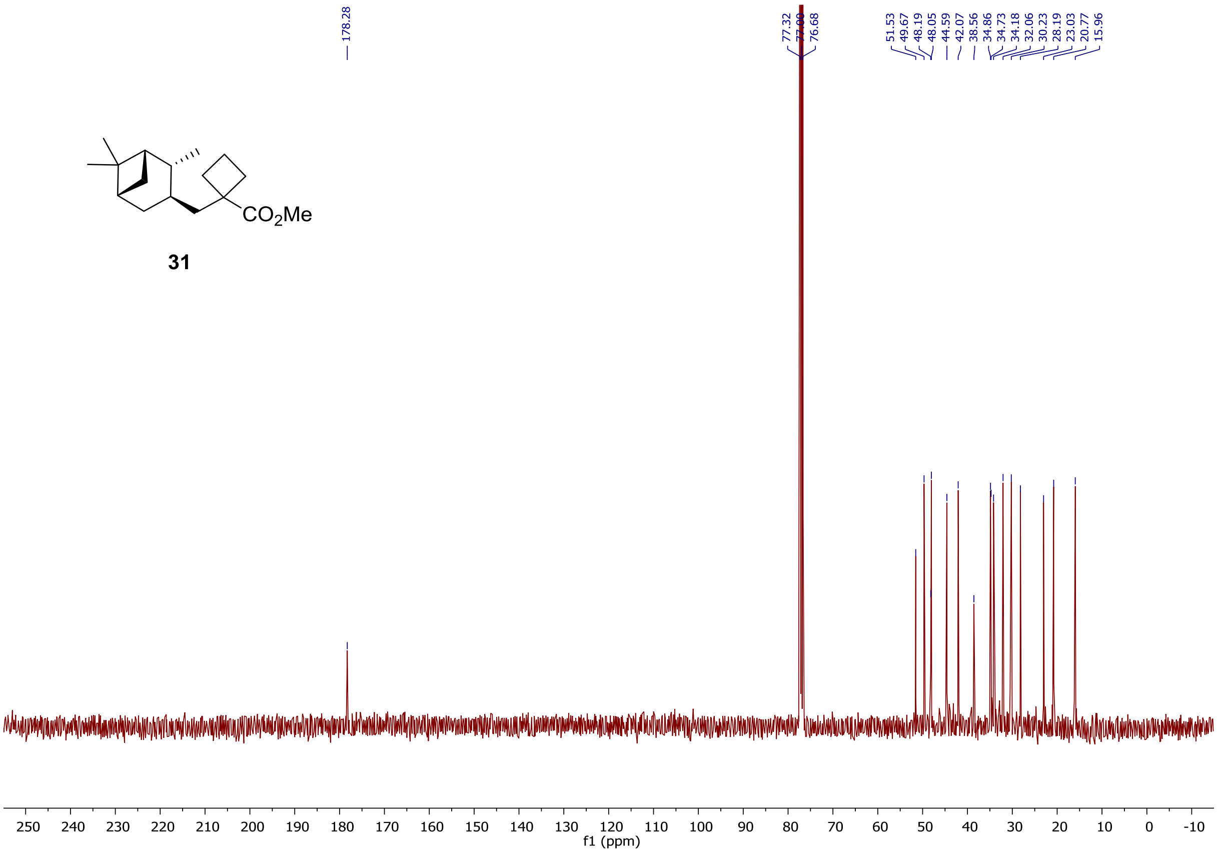

^1H NMR (400 MHz, $CDCl_3$)

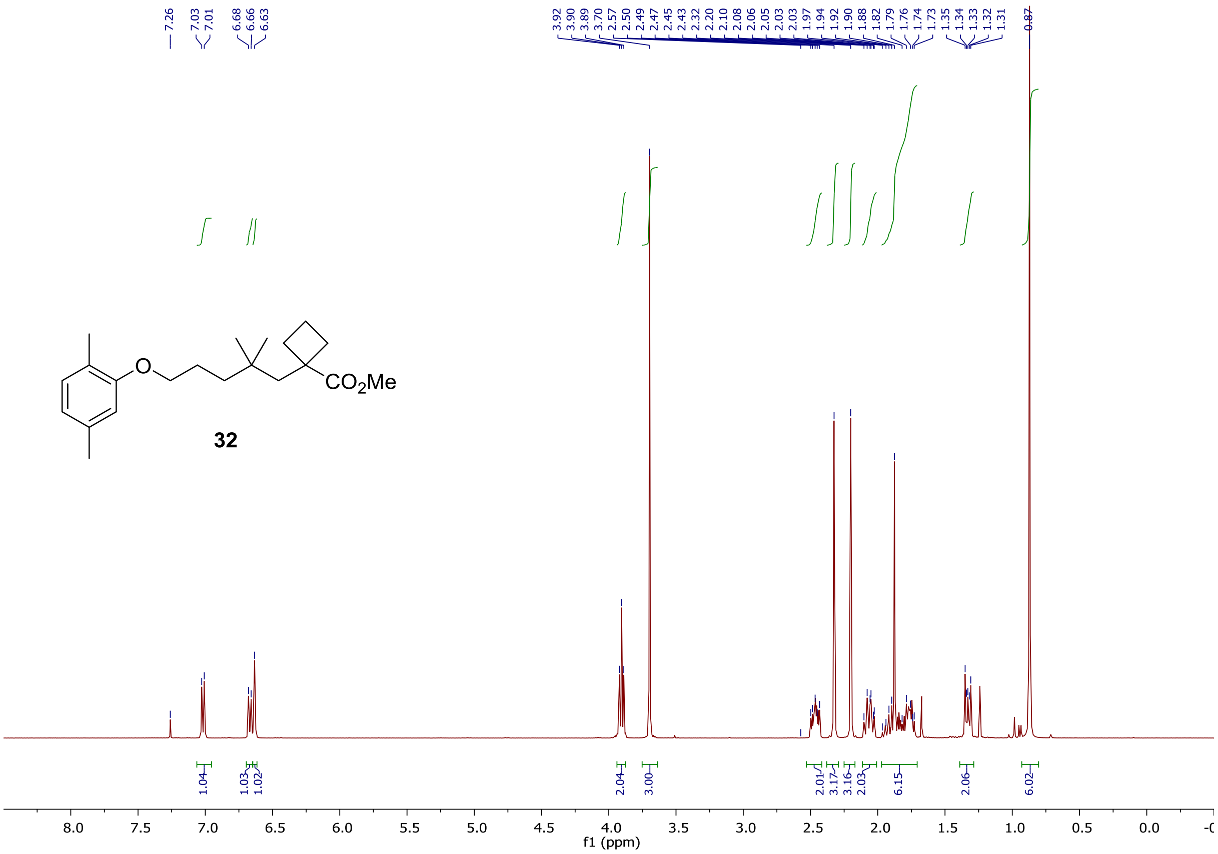

^{13}C NMR (101 MHz, $CDCl_3$)

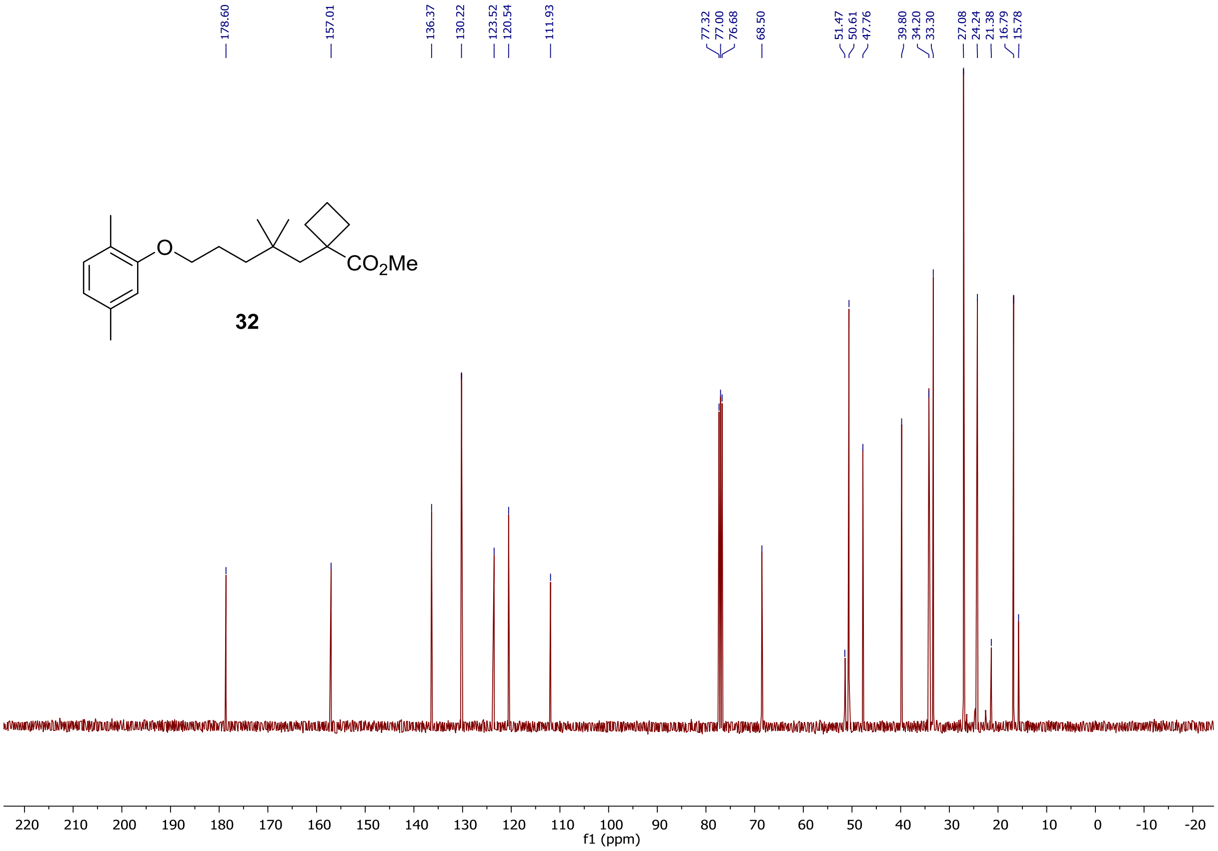

^{1}H NMR (400 MHz, $CDCl_3$)

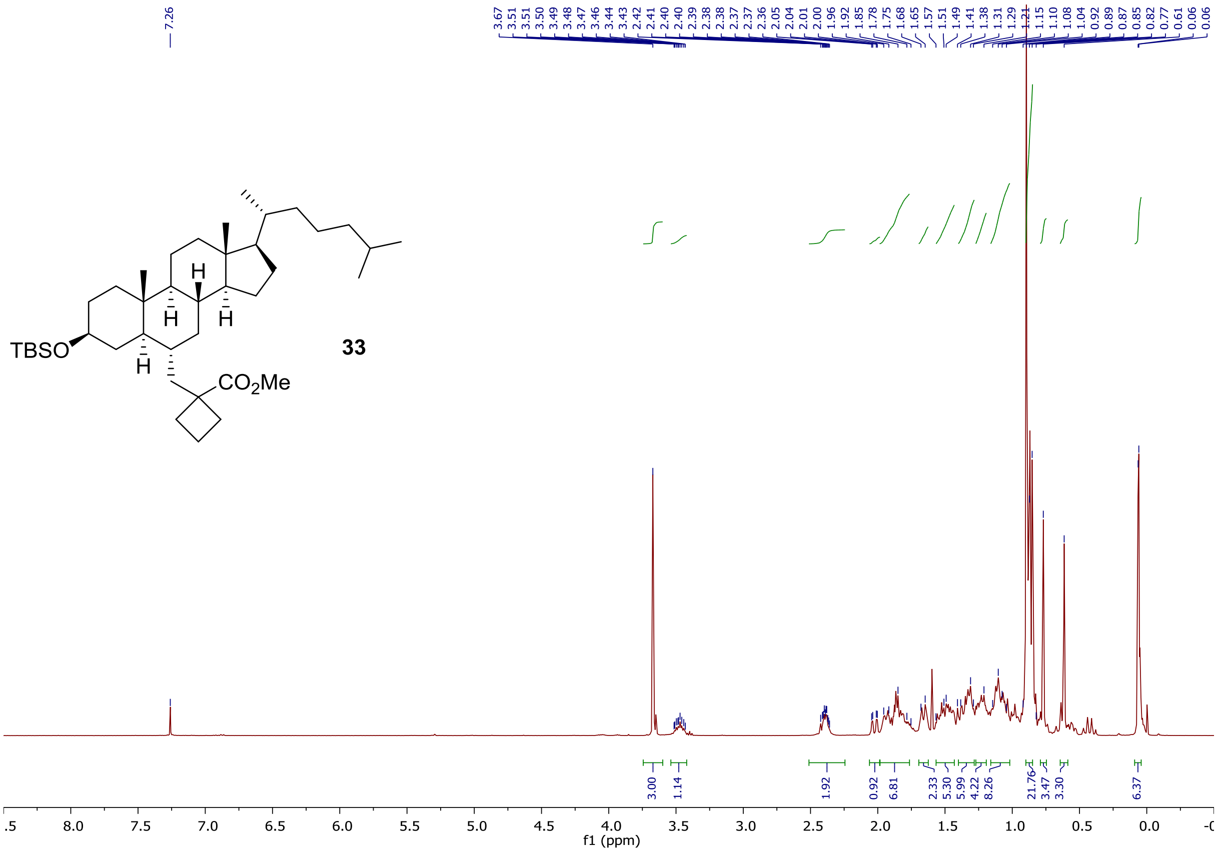

^{13}C NMR (101 MHz, $CDCl_3$)

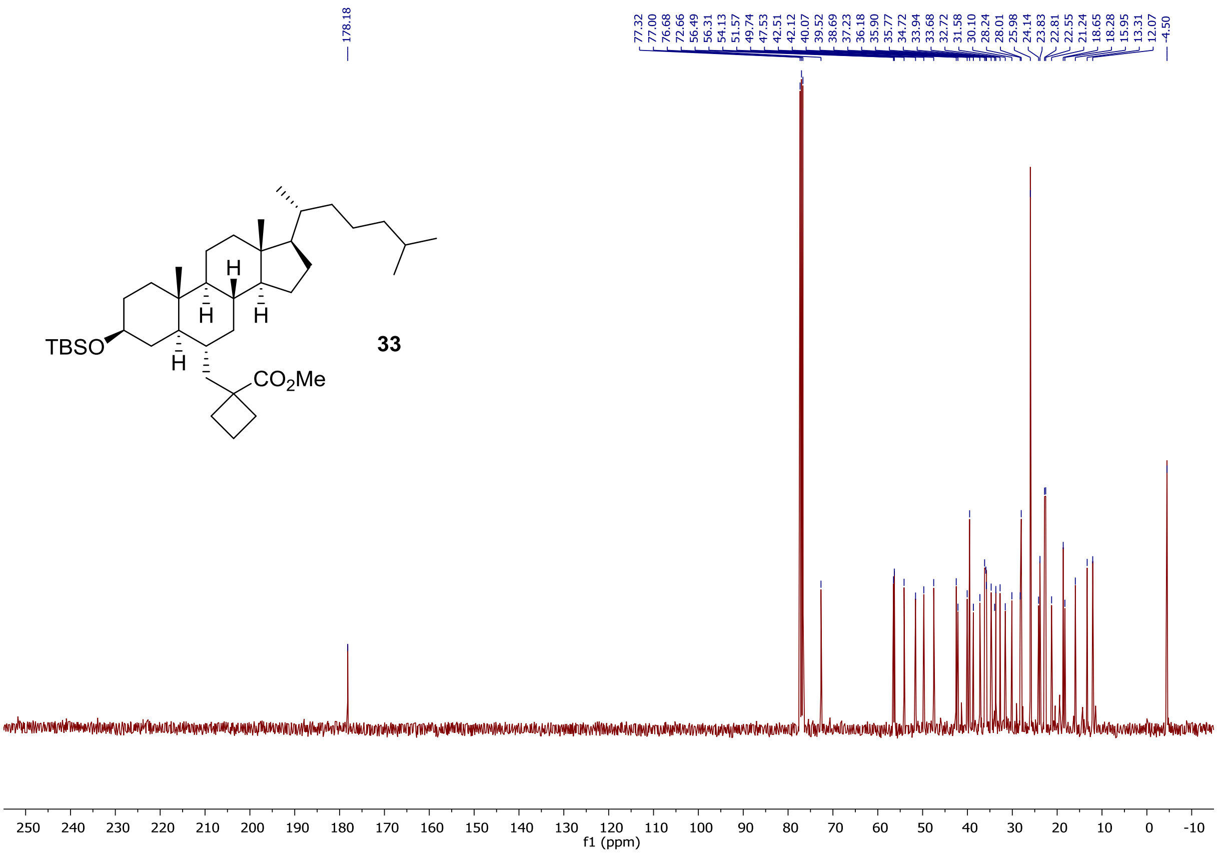

^{1}H NMR (400 MHz, $CDCl_3$)

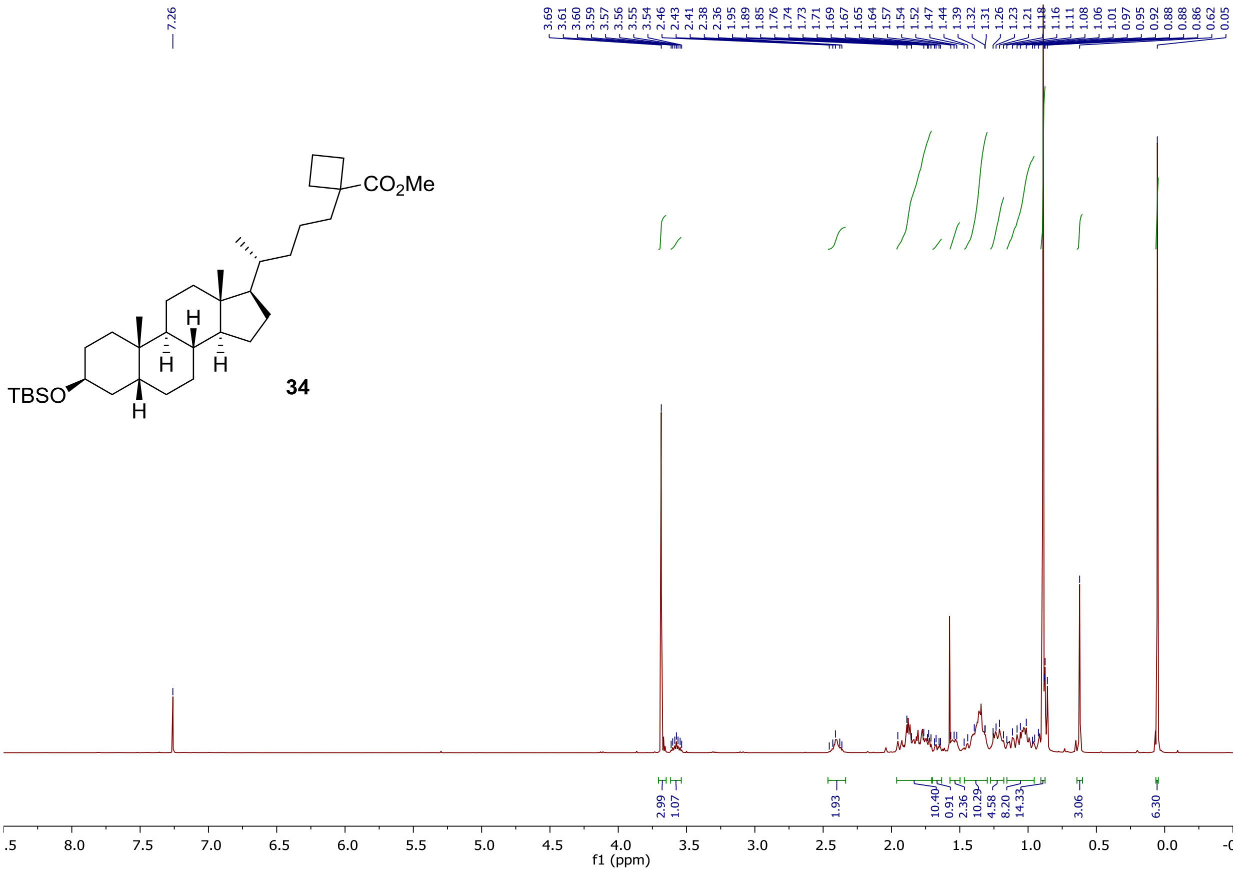

^{13}C NMR (101 MHz, $CDCl_3$)

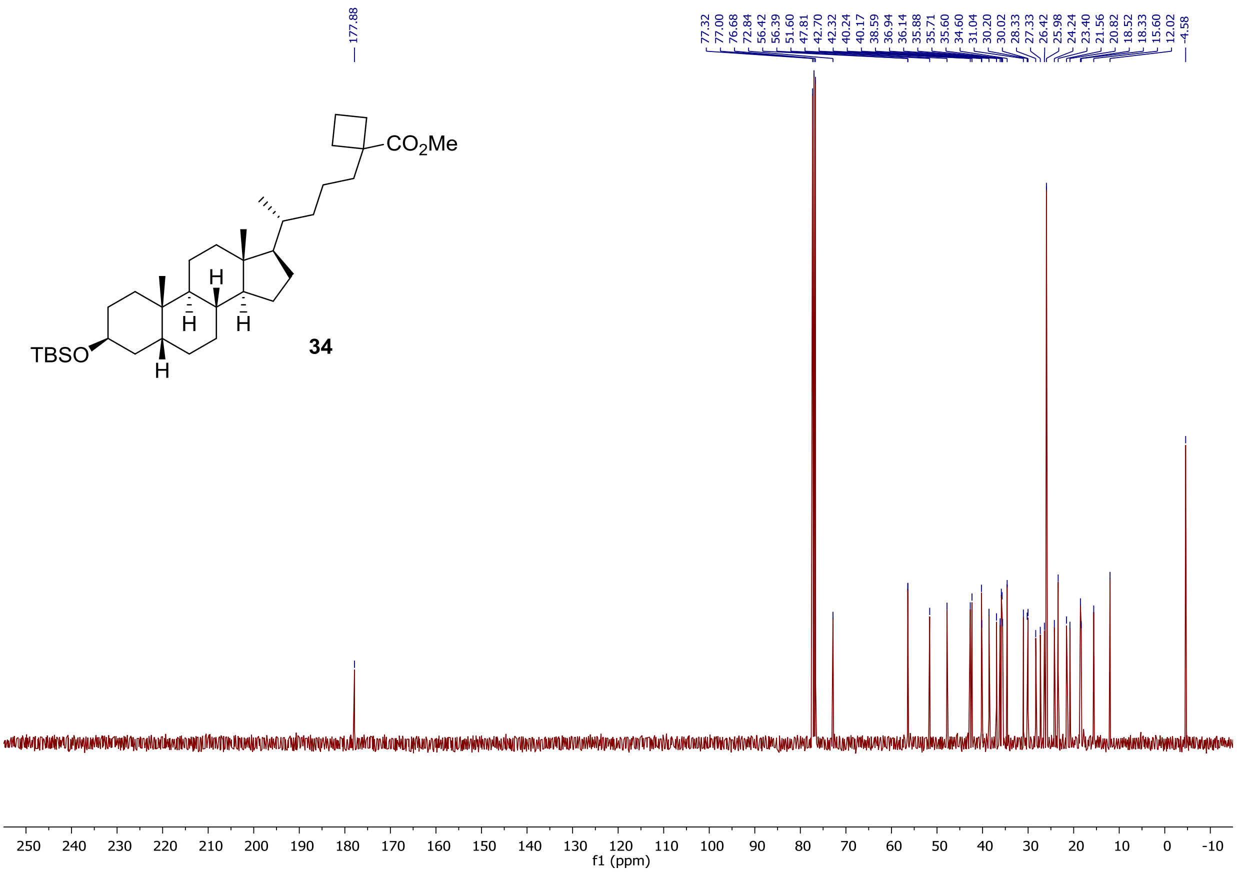

^{1}H NMR (400 MHz, $CDCl_3$)

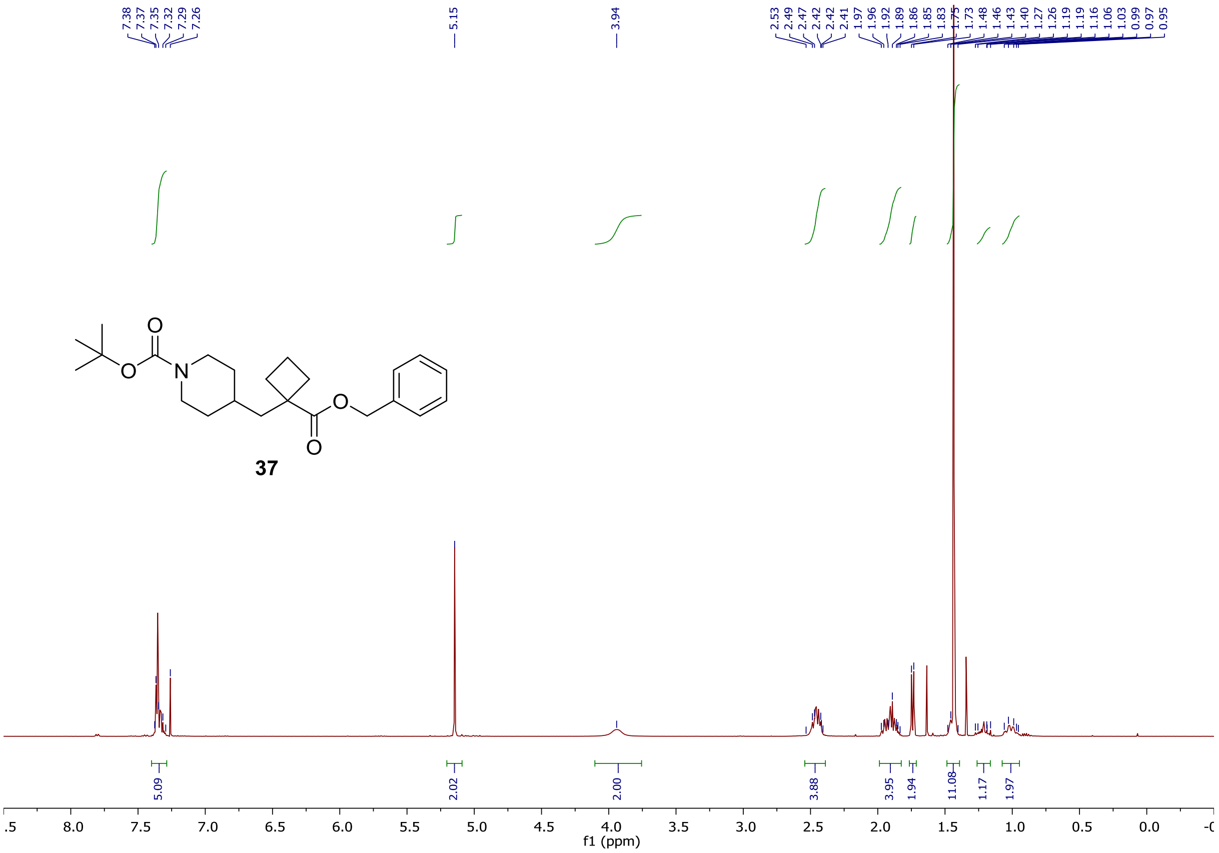

^{13}C NMR (101 MHz, $CDCl_3$)

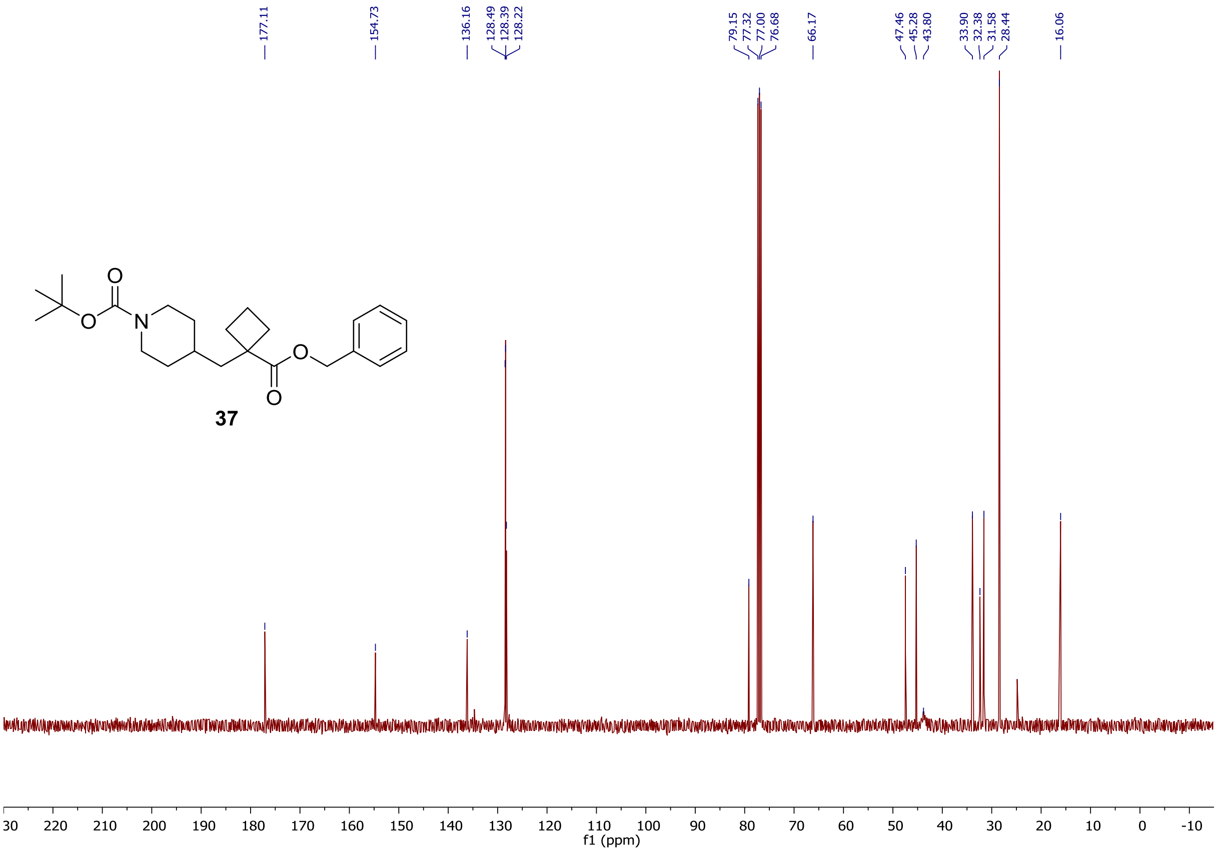

^{1}H NMR (400 MHz, $CDCl_3$)

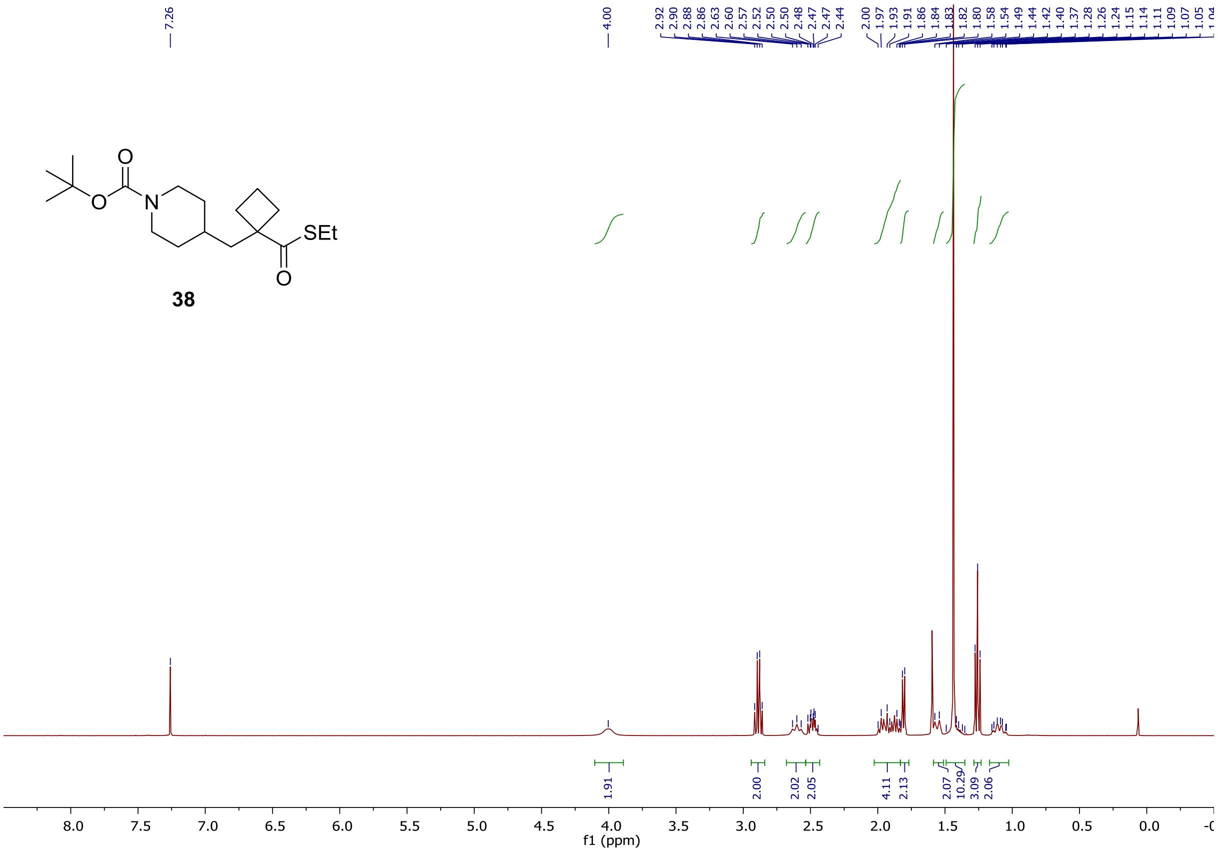

^{13}C NMR (101 MHz, $CDCl_3$)

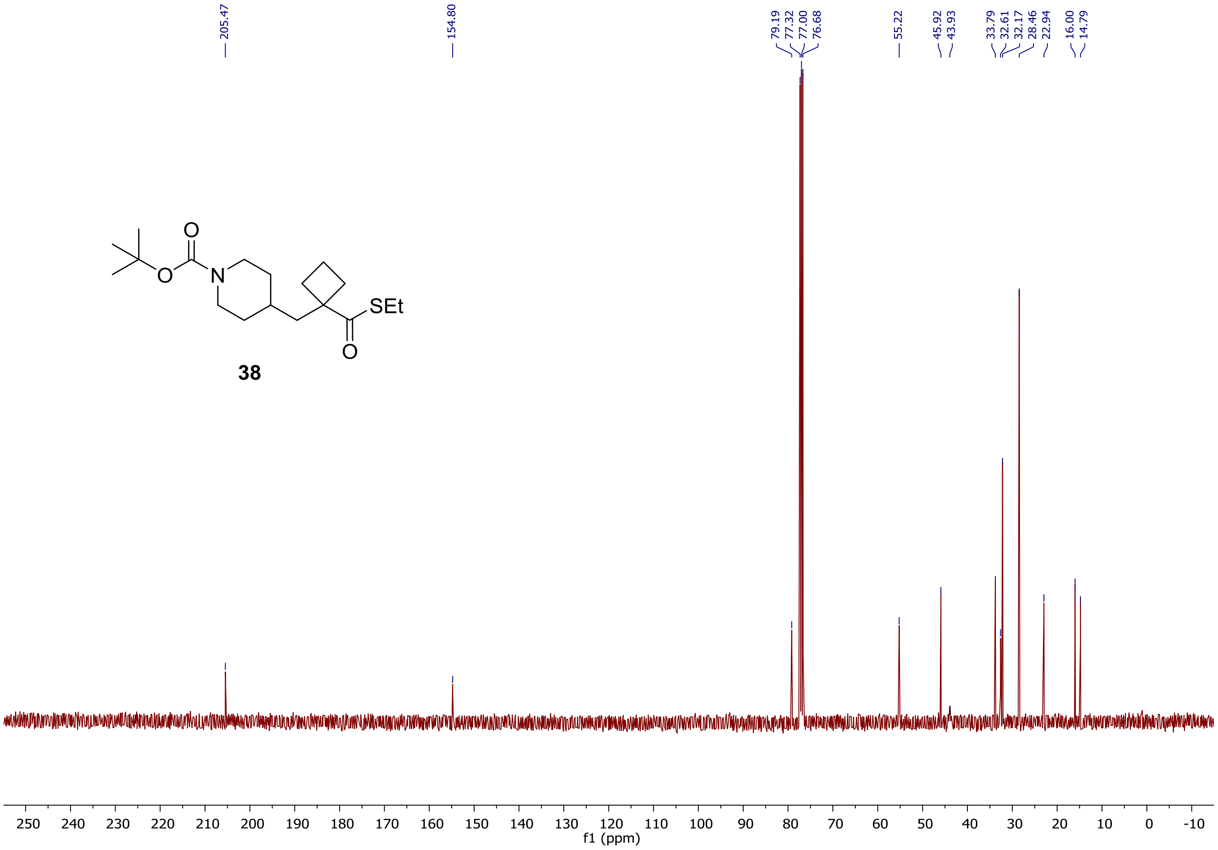

^{1}H NMR (400 MHz, $CDCl_3$)

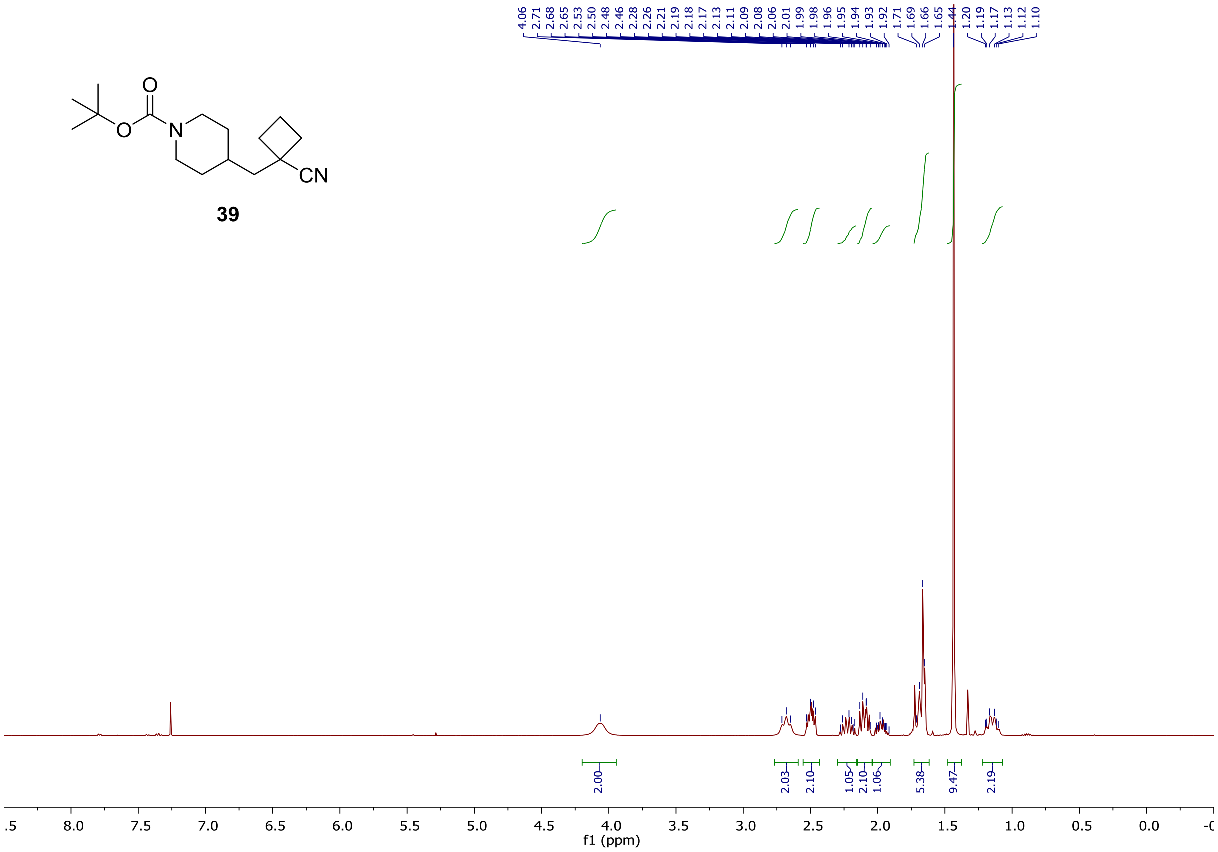

^{13}C NMR (101 MHz, $CDCl_3$)

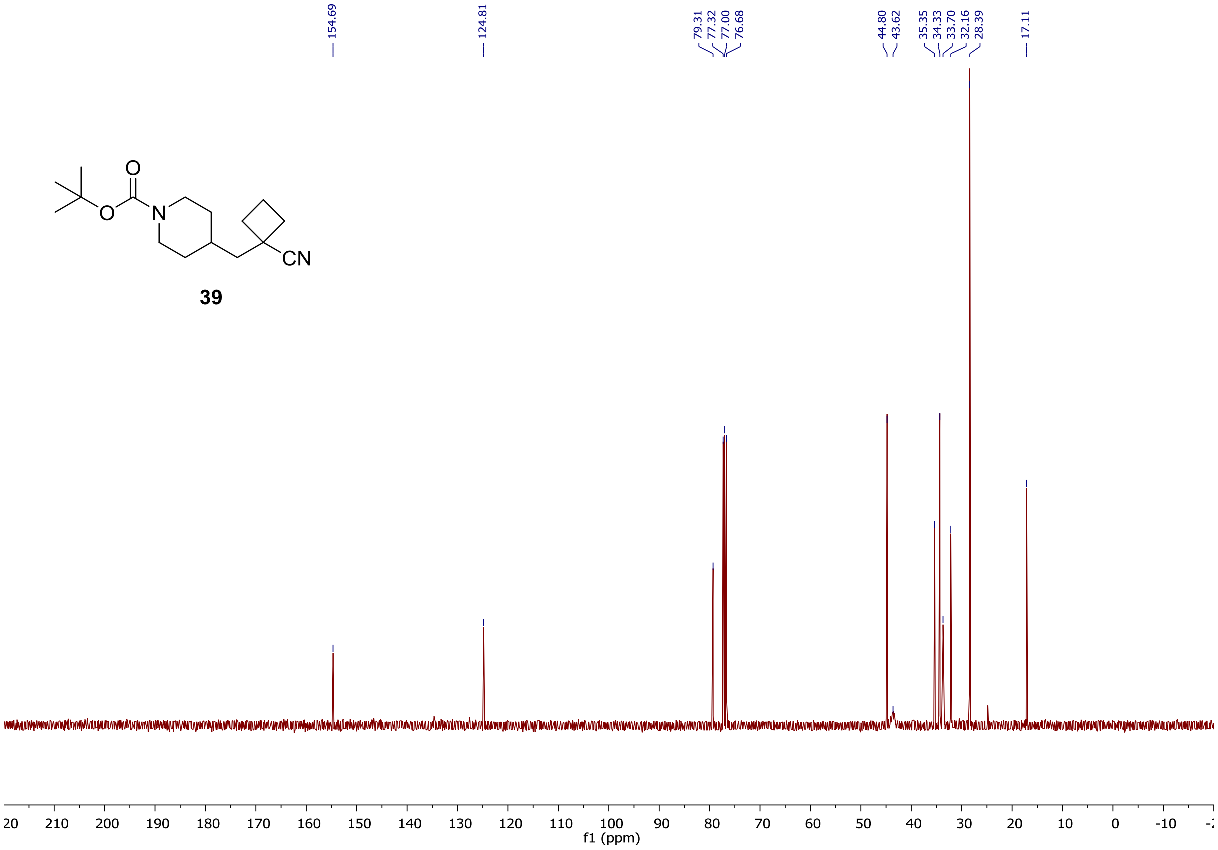

^{1}H NMR (400 MHz, $CDCl_3$)

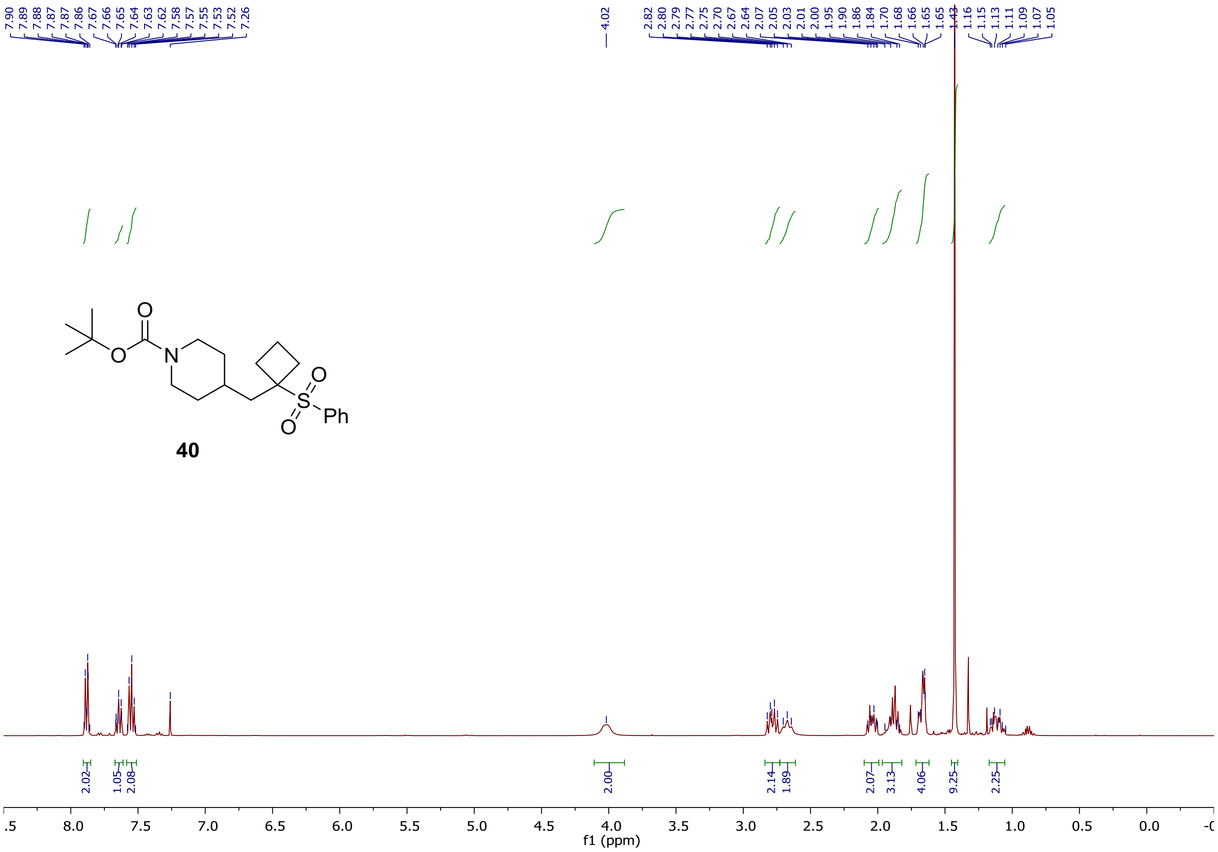

^{13}C NMR (101 MHz, $CDCl_3$)

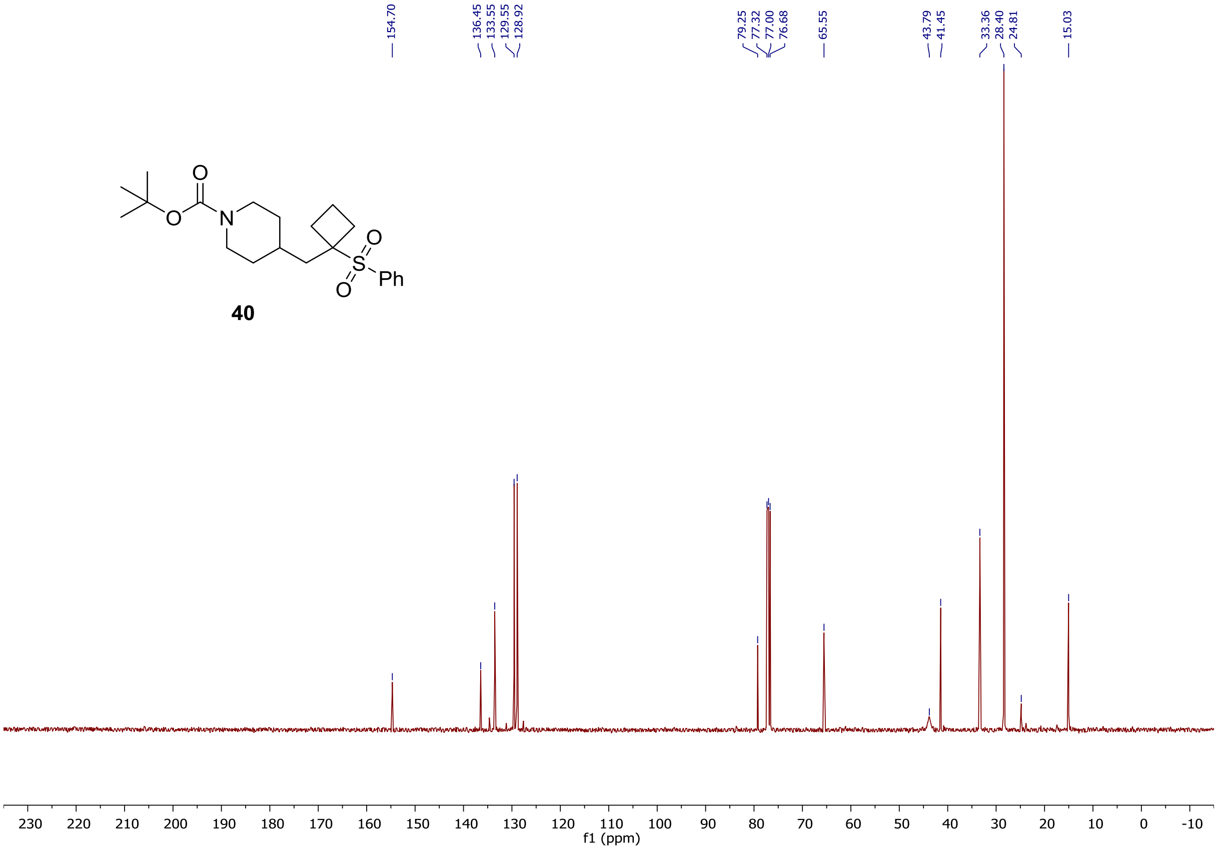

^{1}H NMR (400 MHz, $CDCl_3$)

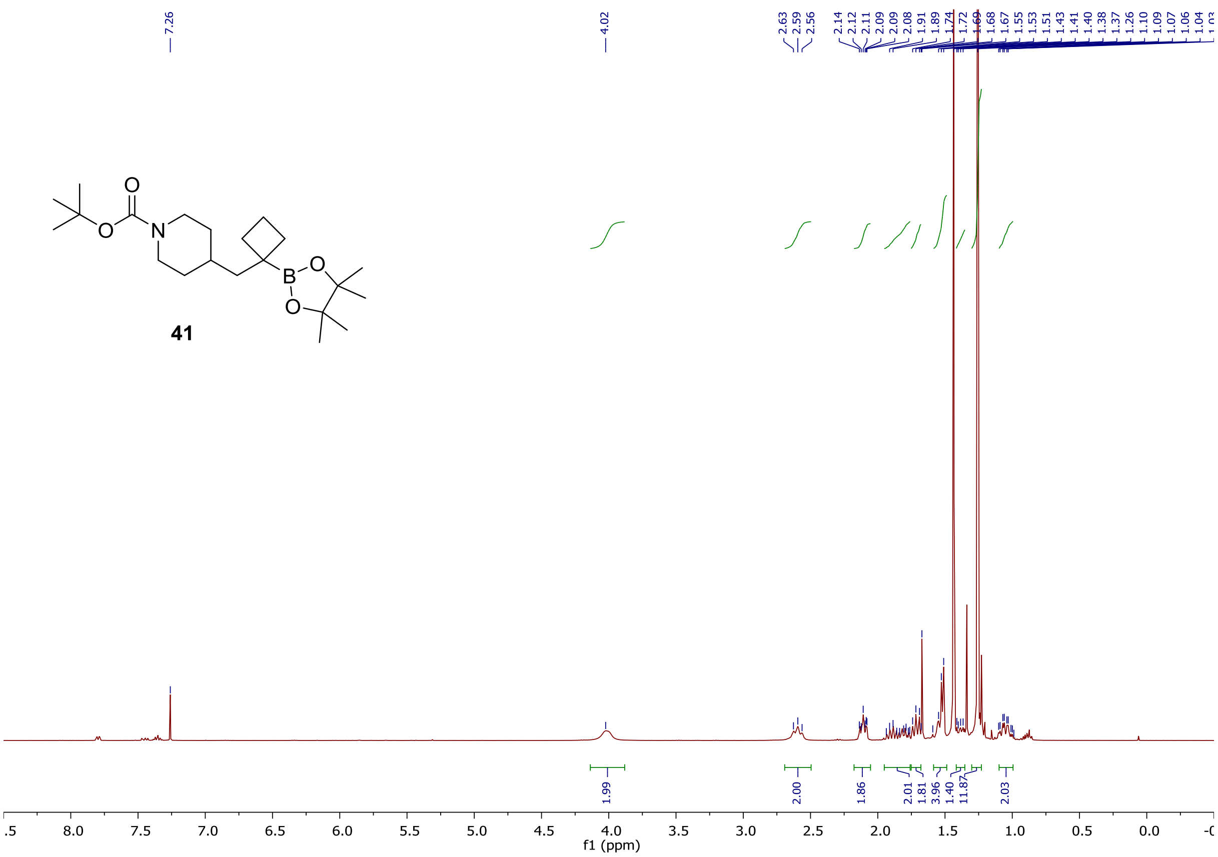

^{13}C NMR (101 MHz, $CDCl_3$)

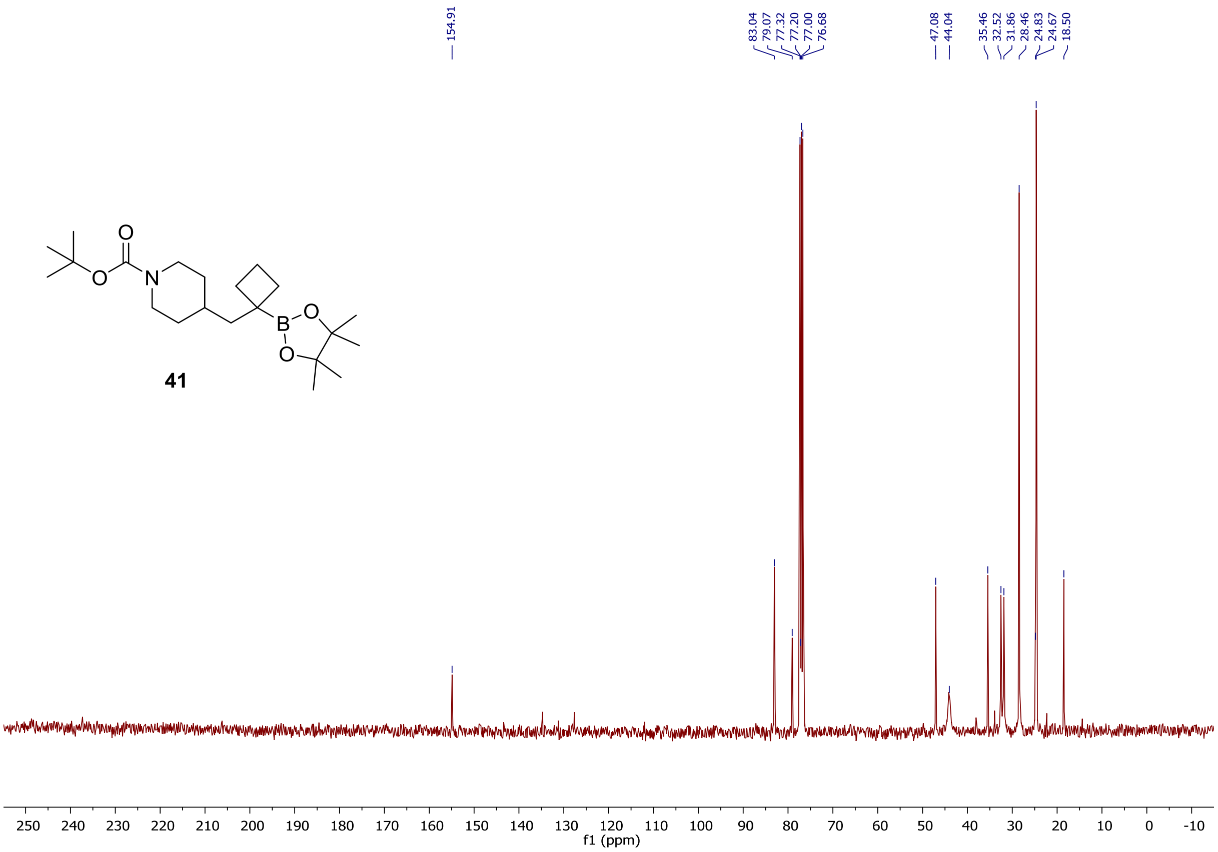

^{1}H NMR (400 MHz, $CDCl_3$)

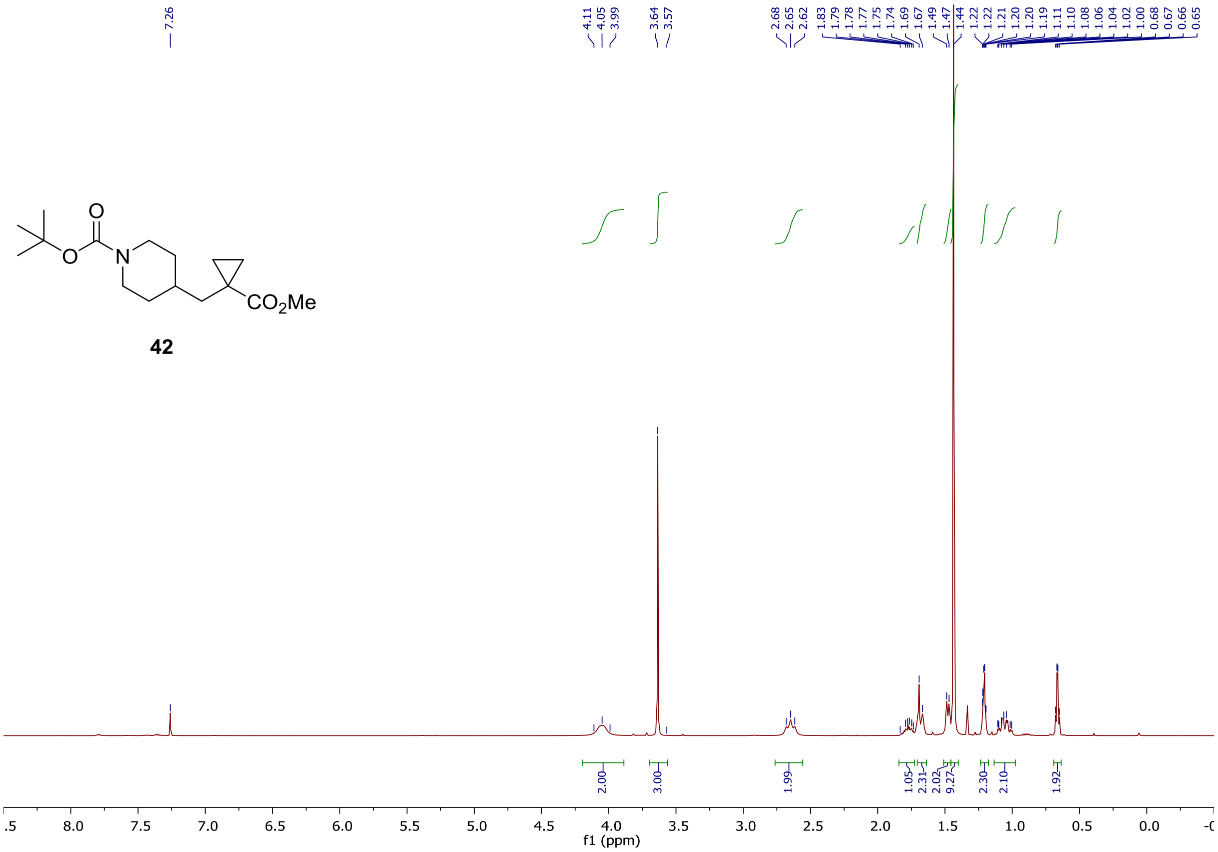

^{13}C NMR (101 MHz, $CDCl_3$)

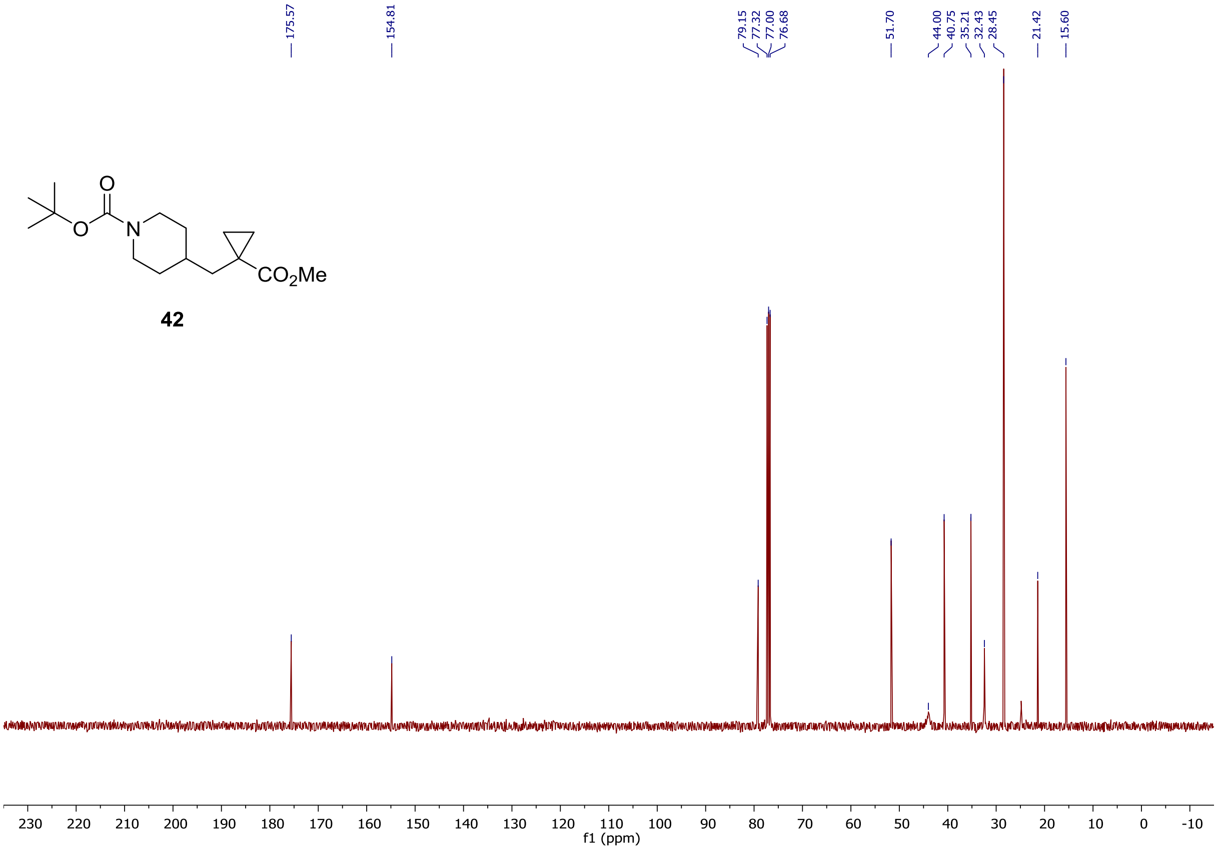

^{1}H NMR (400 MHz, $CDCl_3$)

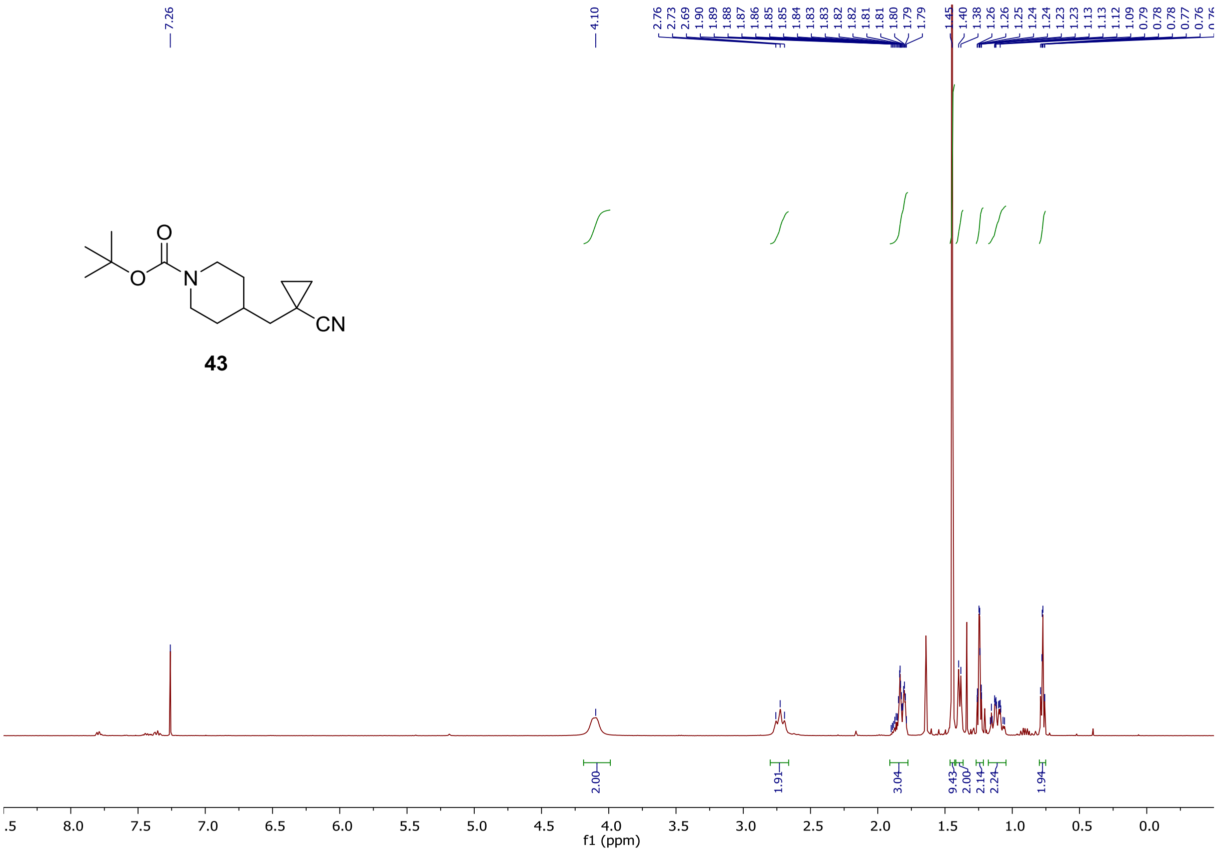

^{13}C NMR (101 MHz, $CDCl_3$)

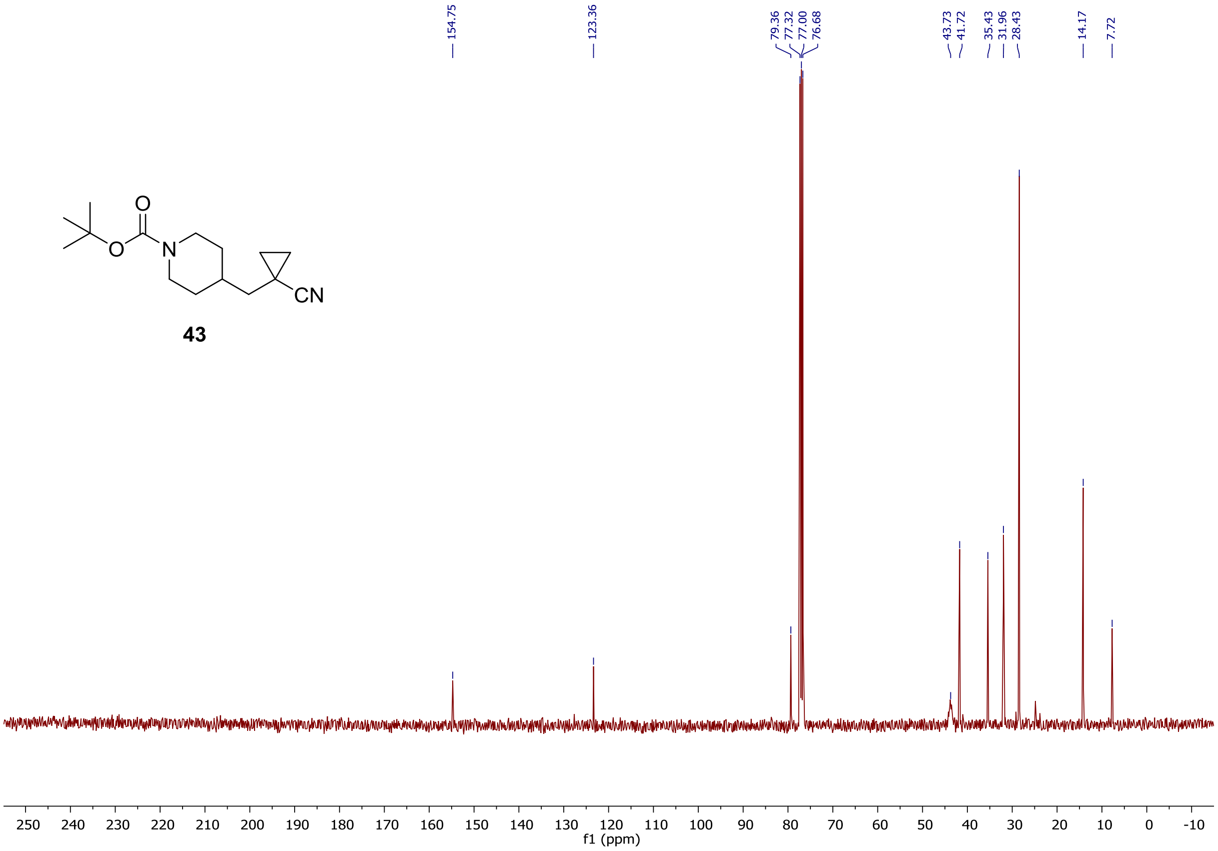

^{1}H NMR (400 MHz, $CDCl_3$)

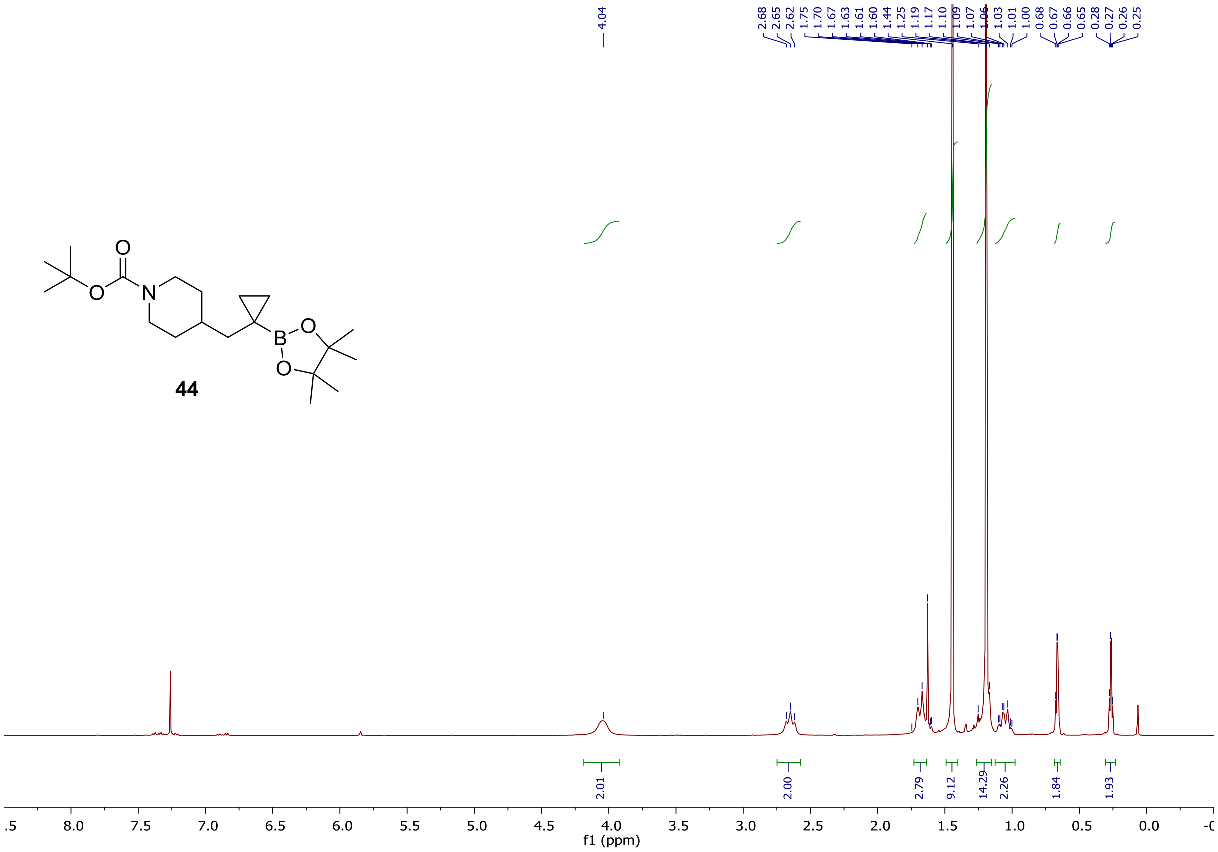

^{13}C NMR (101 MHz, $CDCl_3$)

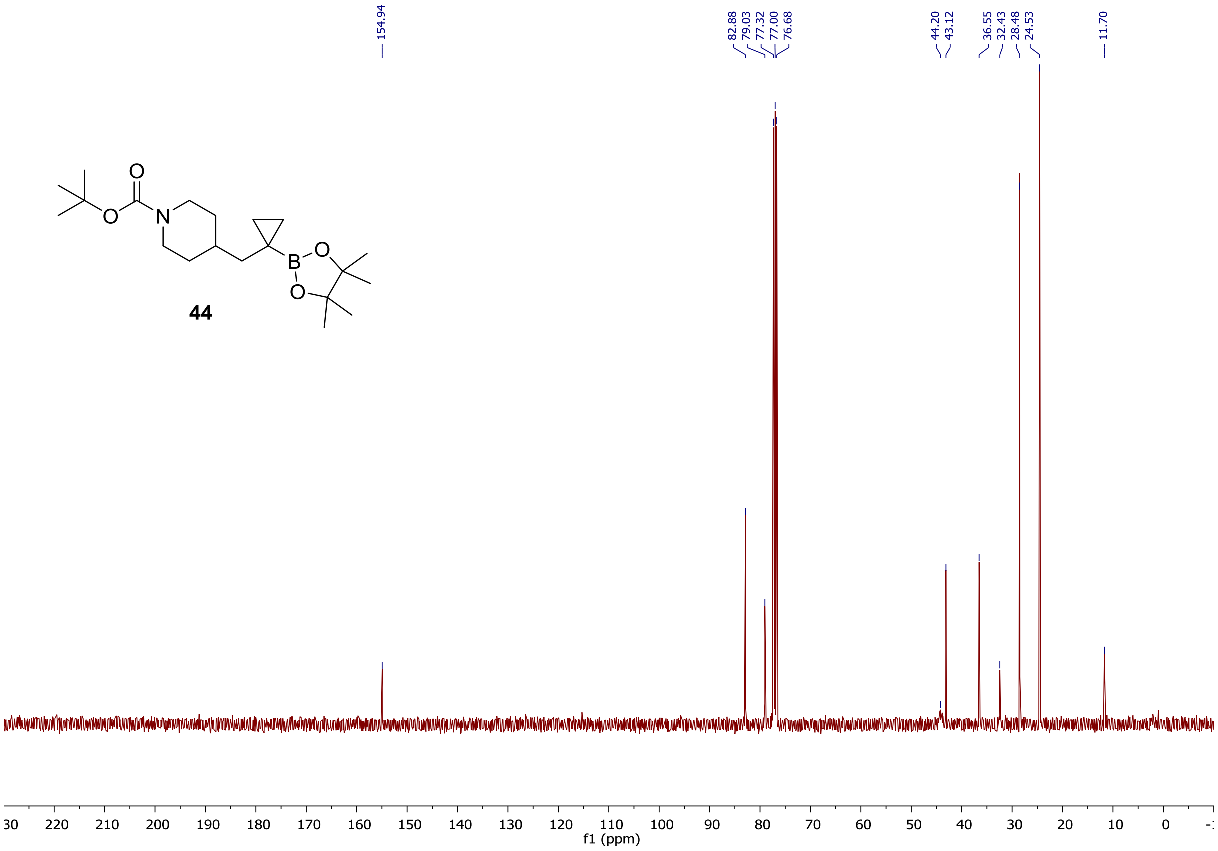

^{1}H NMR (400 MHz, $CDCl_3$)

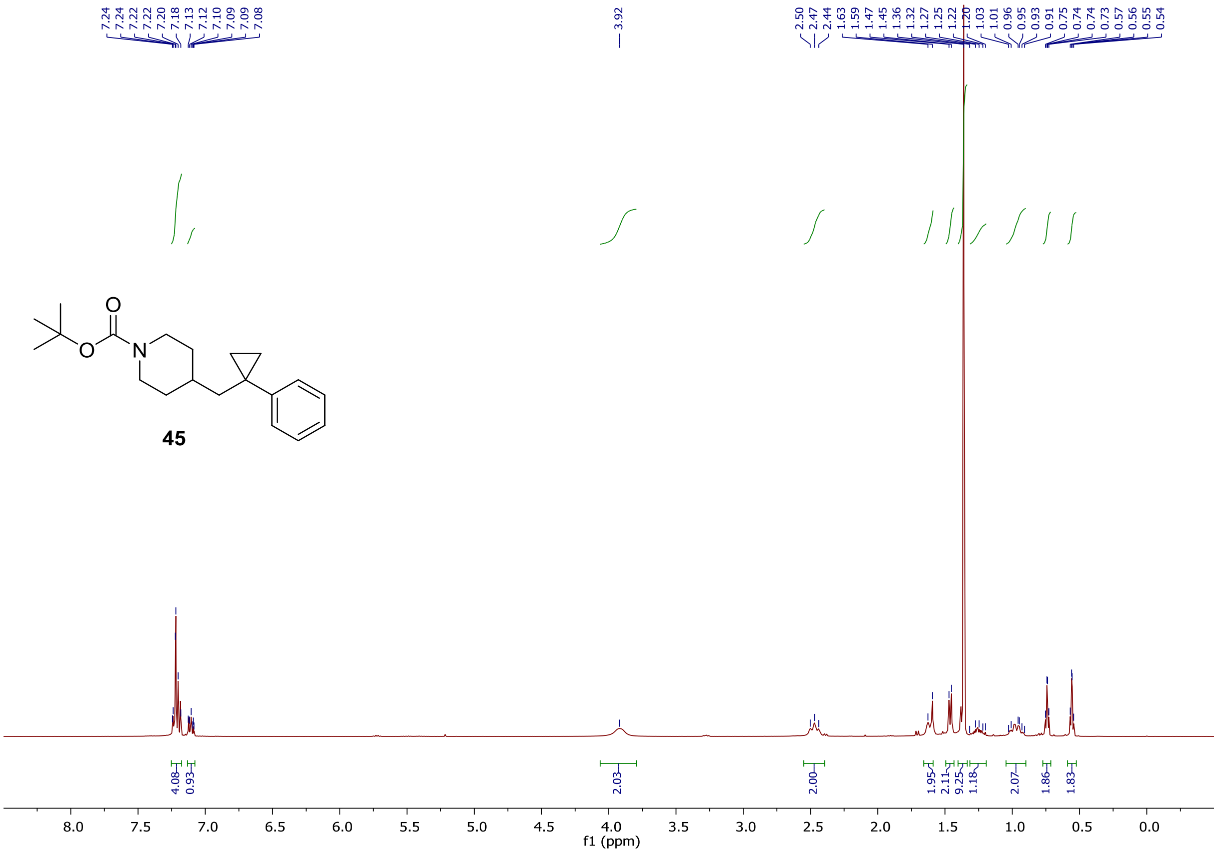

^{13}C NMR (101 MHz, $CDCl_3$)

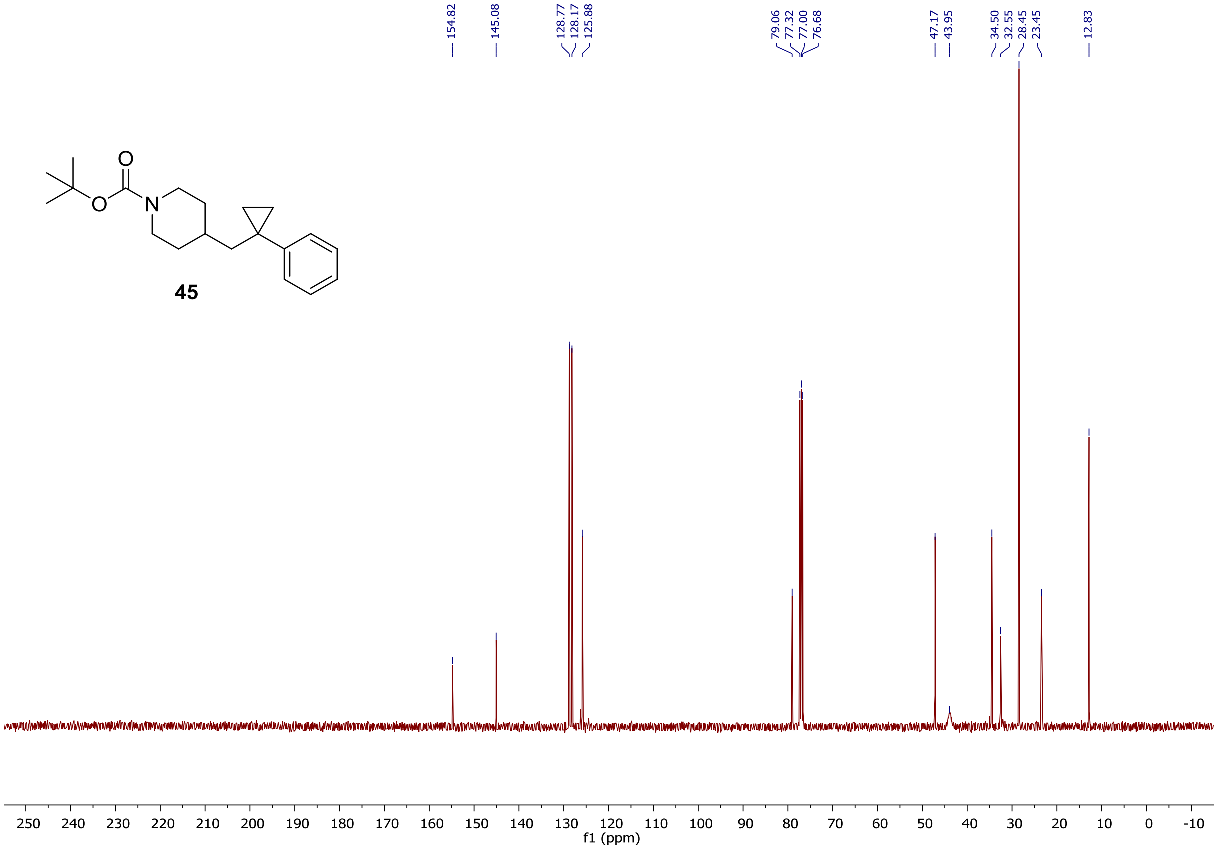

^{1}H NMR (400 MHz, $CDCl_3$)

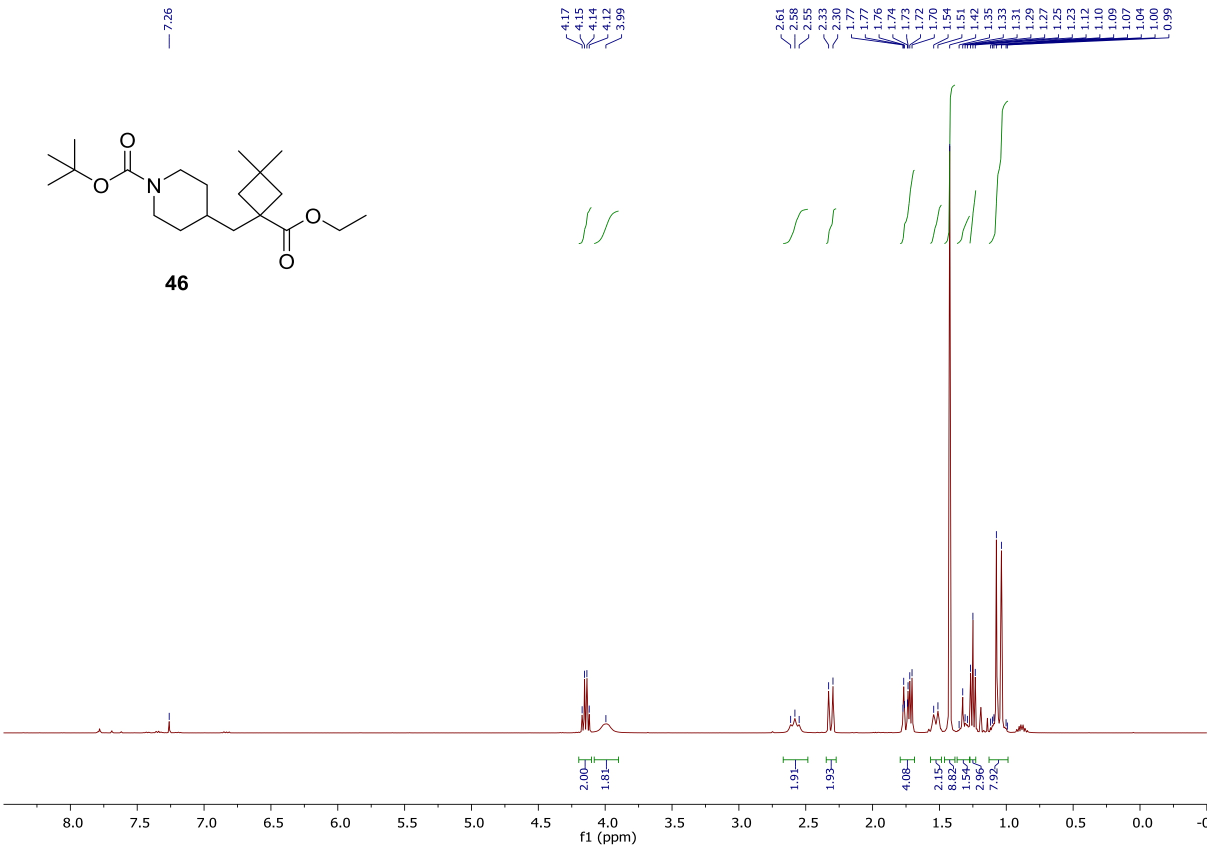

^{13}C NMR (101 MHz, $CDCl_3$)

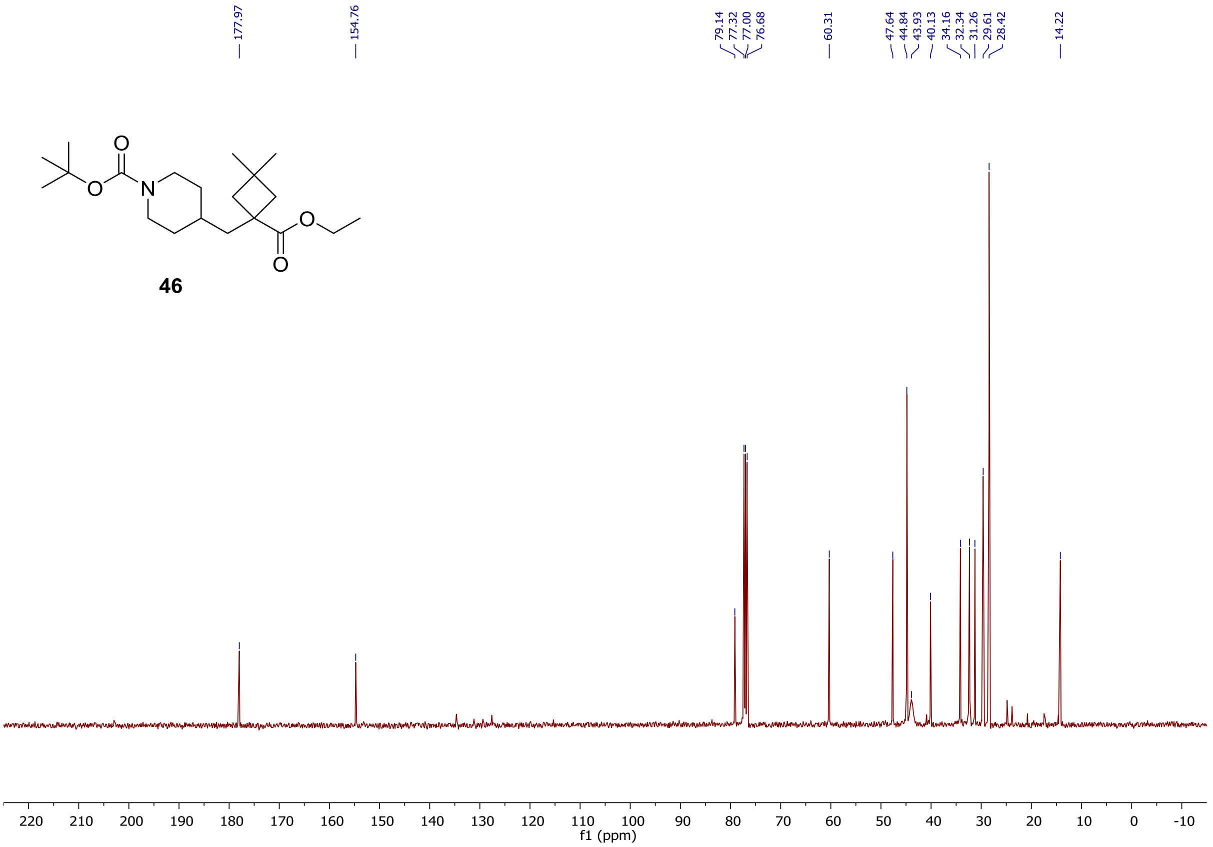

^{1}H NMR (400 MHz, $CDCl_3$)

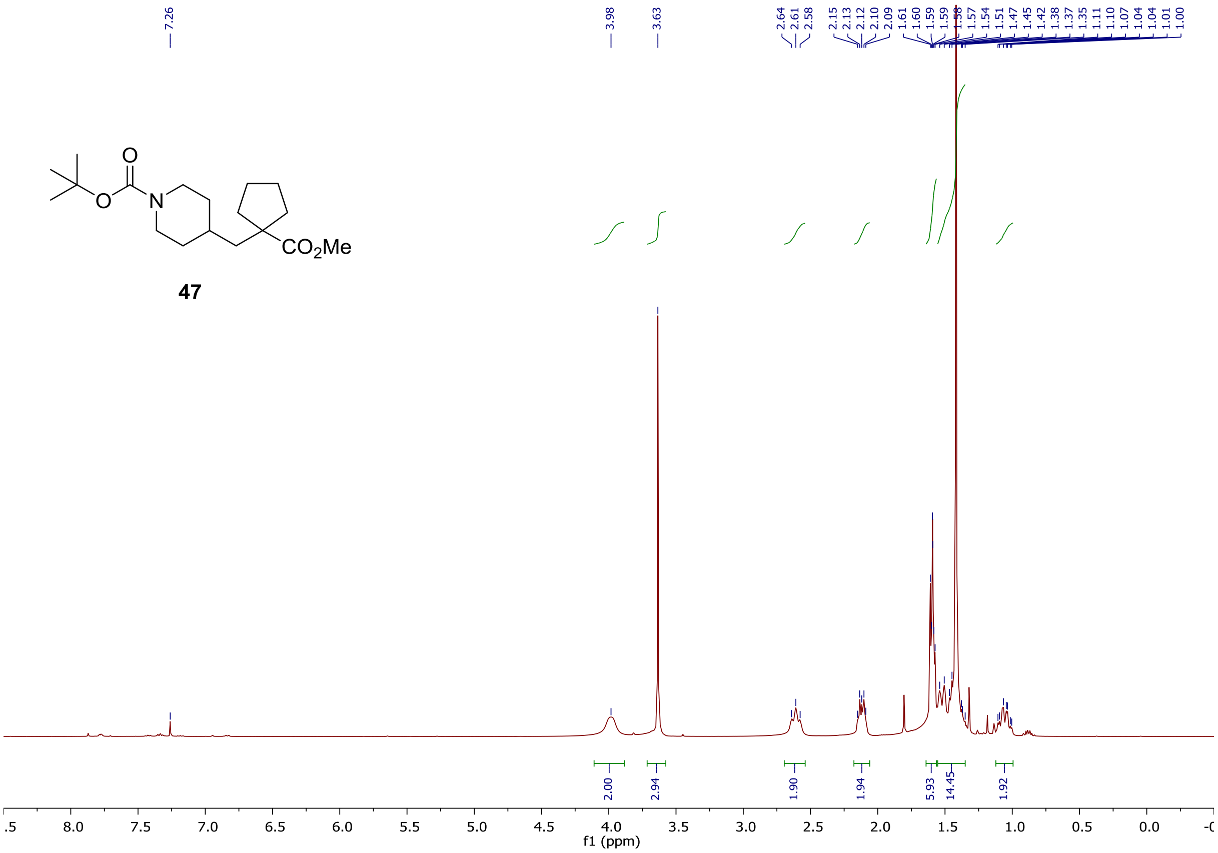

^{13}C NMR (101 MHz, $CDCl_3$)

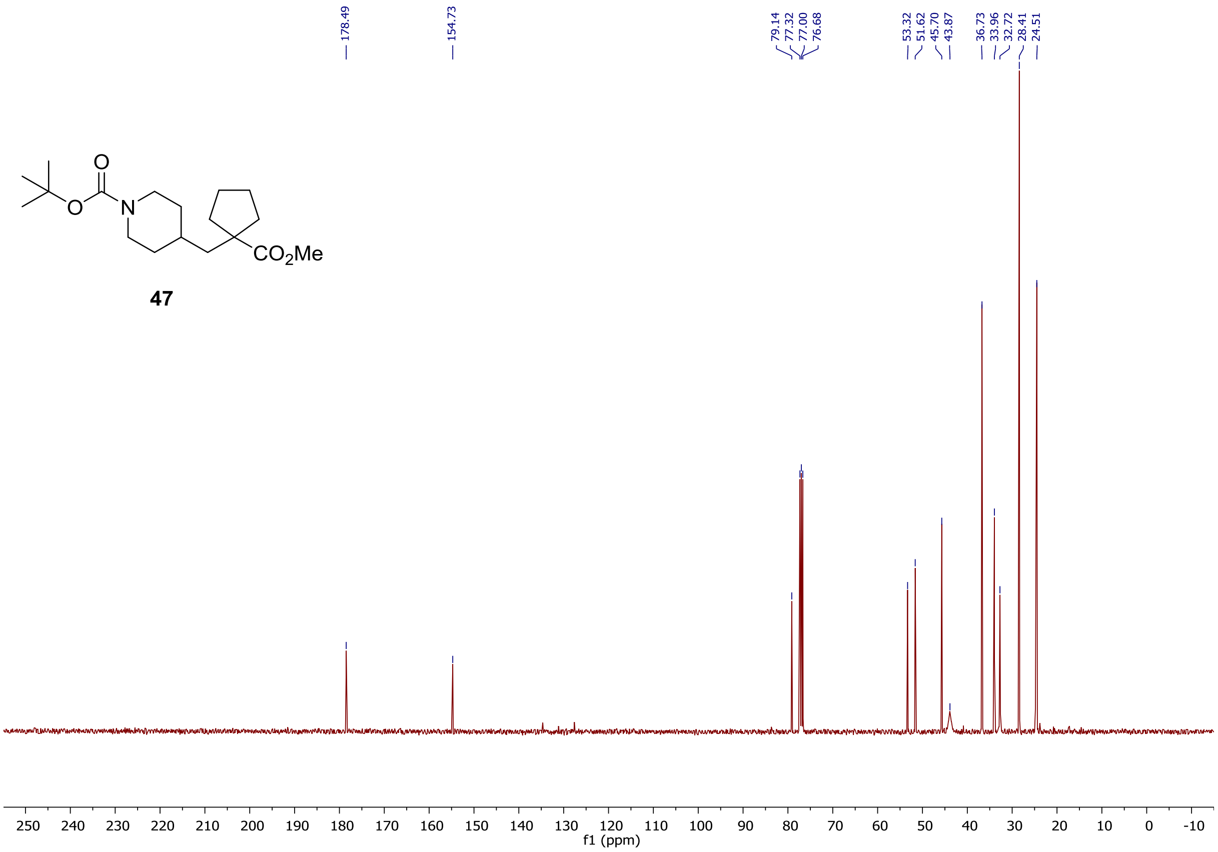

^{1}H NMR (400 MHz, $CDCl_3$)

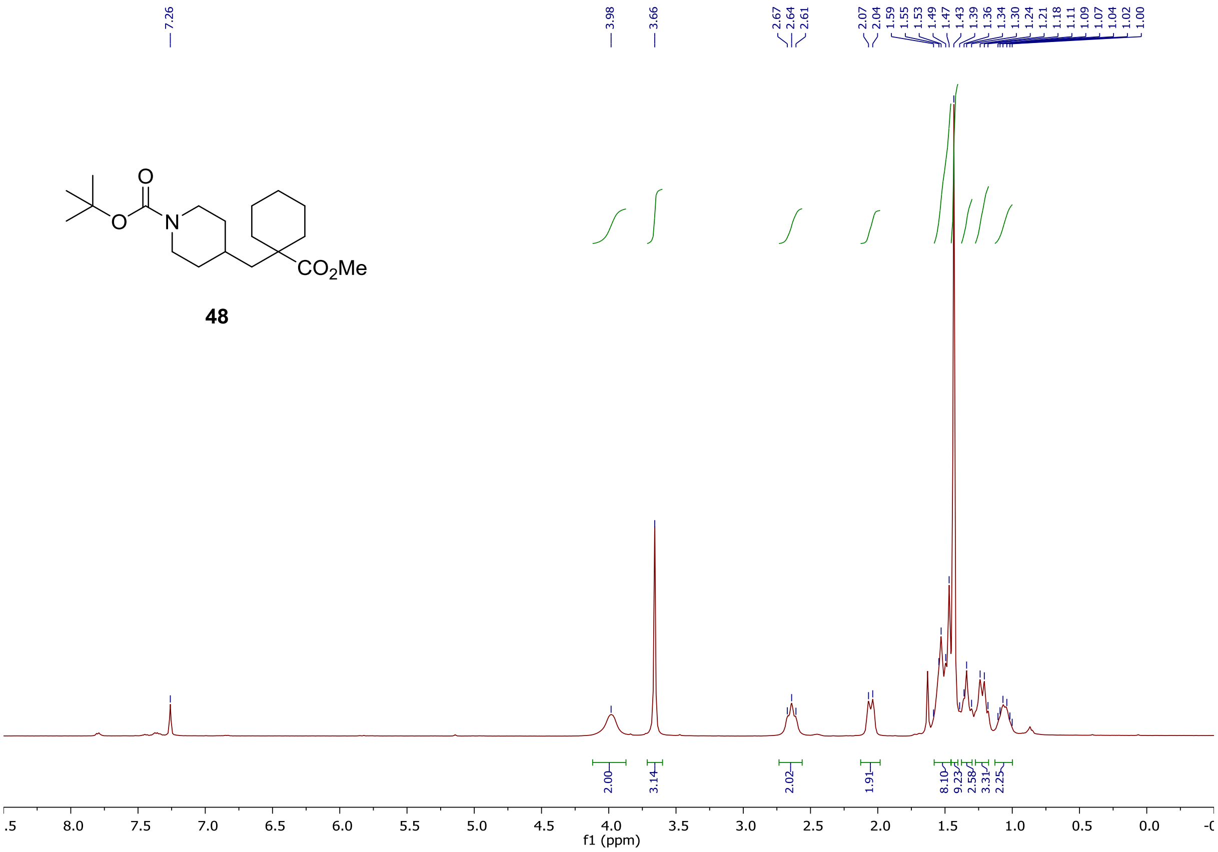

48

^{13}C NMR (101 MHz, $CDCl_3$)

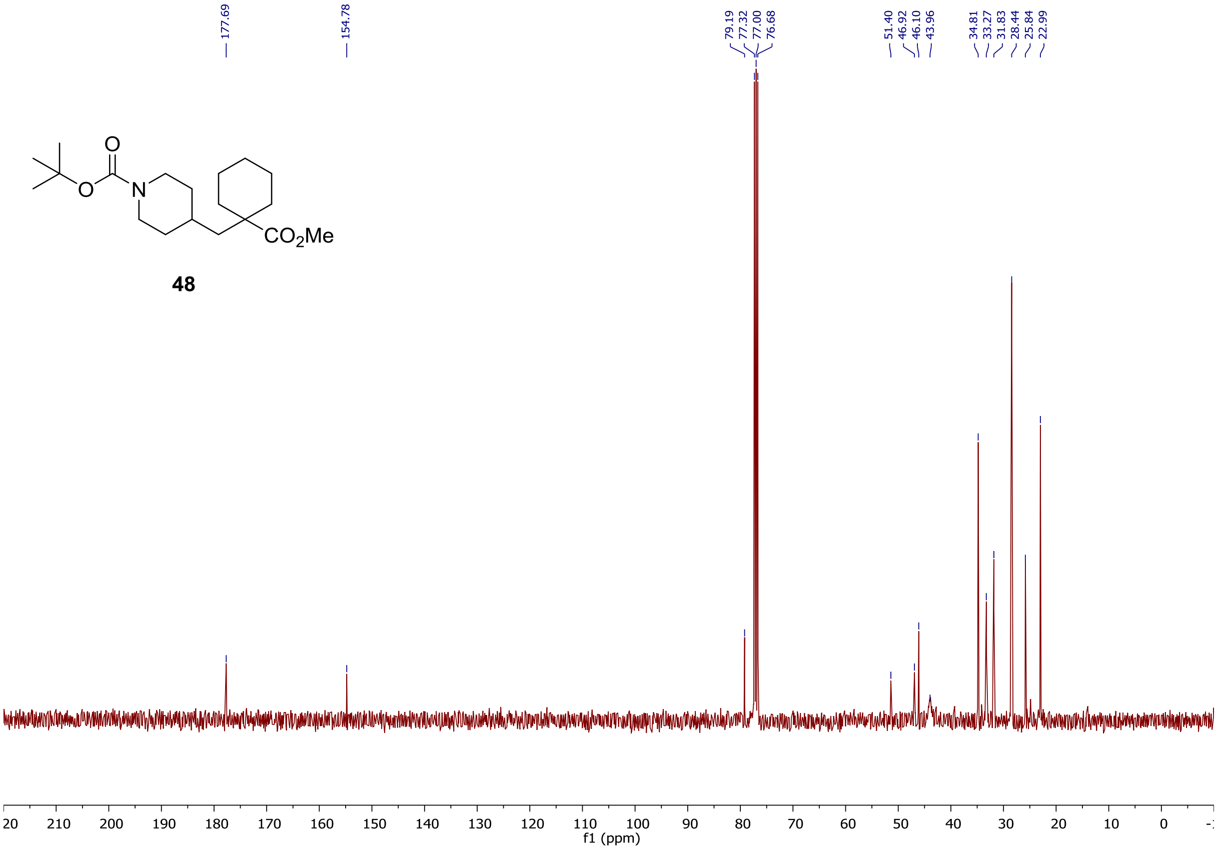

48

^{1}H NMR (400 MHz, $CDCl_3$)

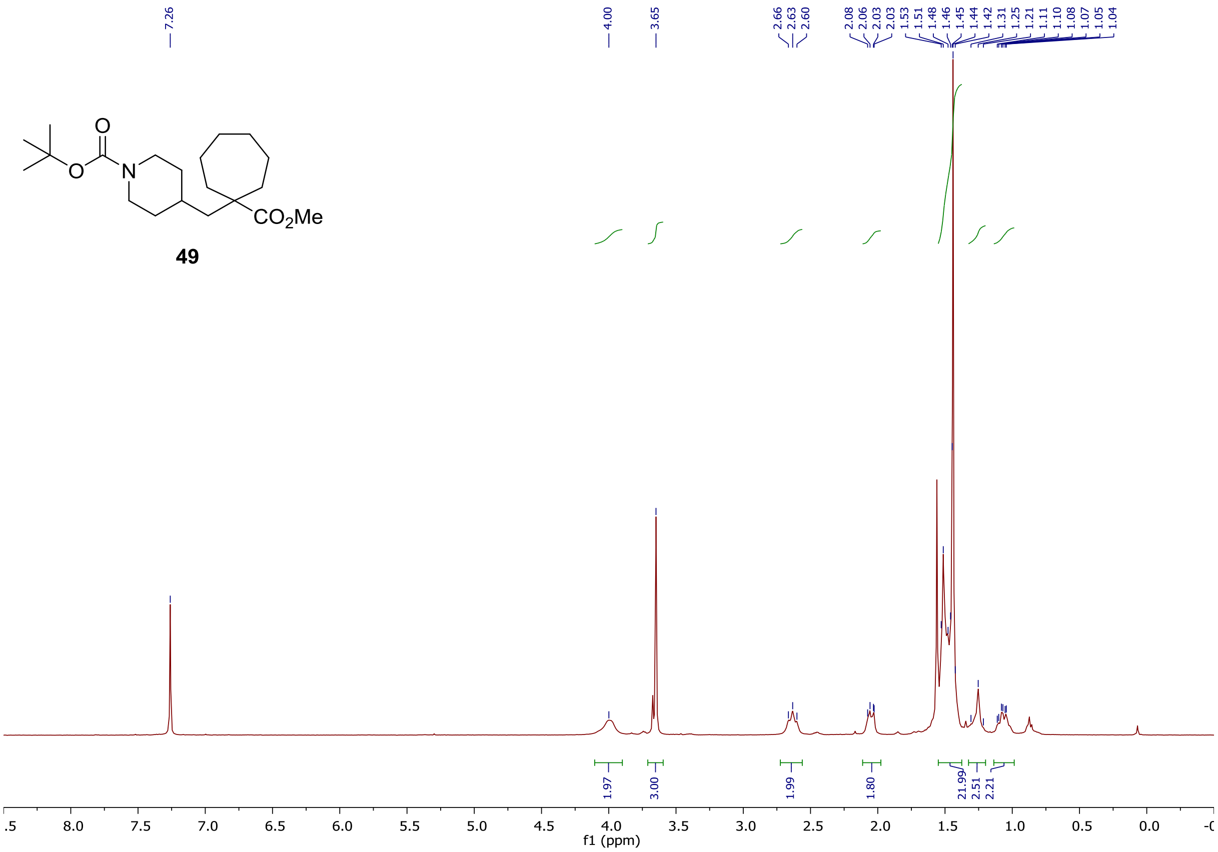

^{13}C NMR (101 MHz, $CDCl_3$)

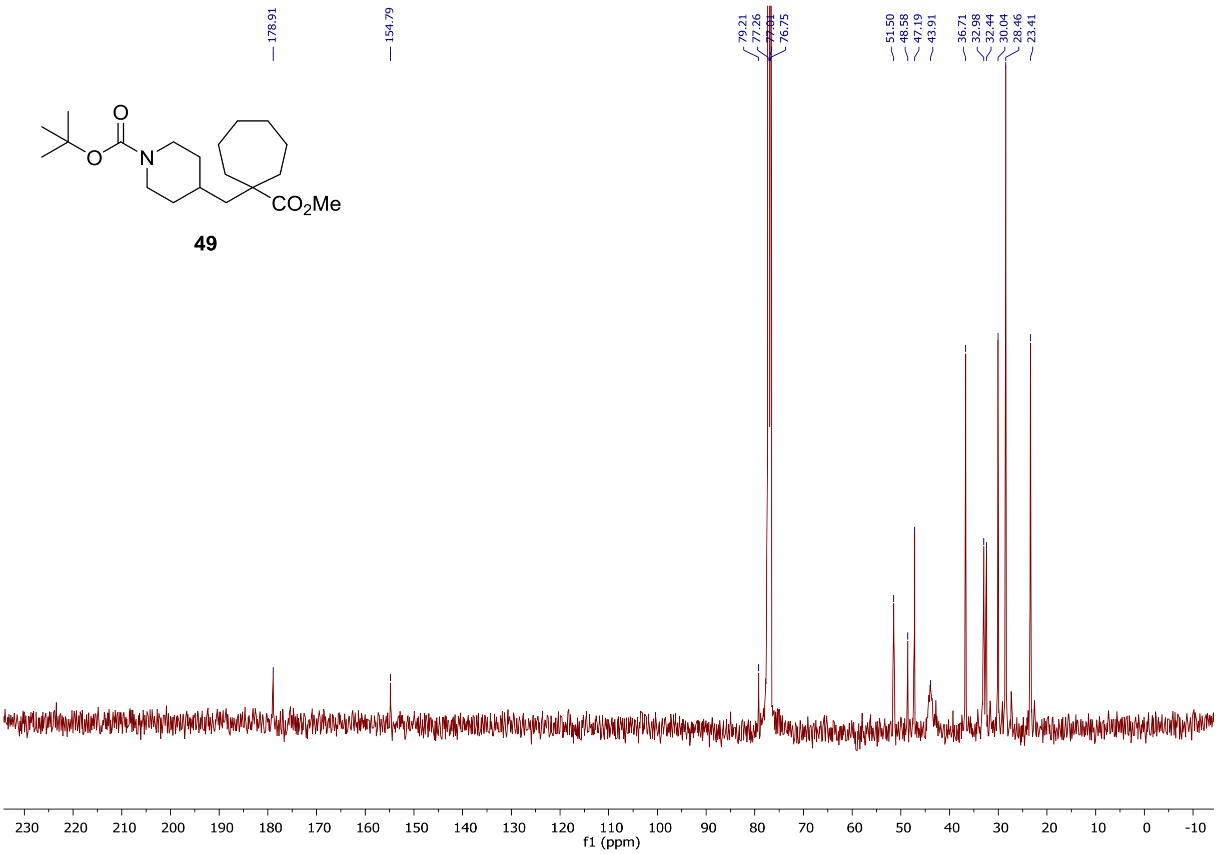

^{1}H NMR (400 MHz, $CDCl_3$)

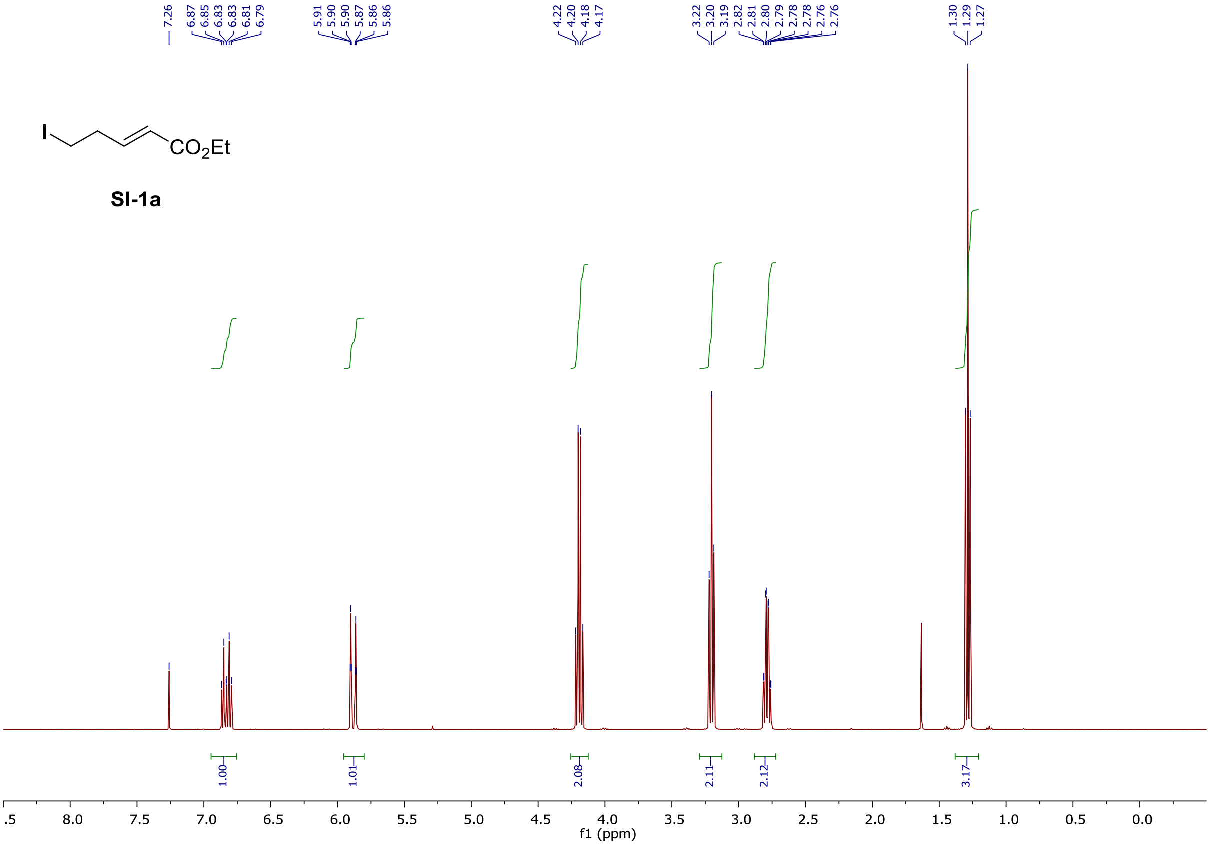

^{13}C NMR (101 MHz, $CDCl_3$)

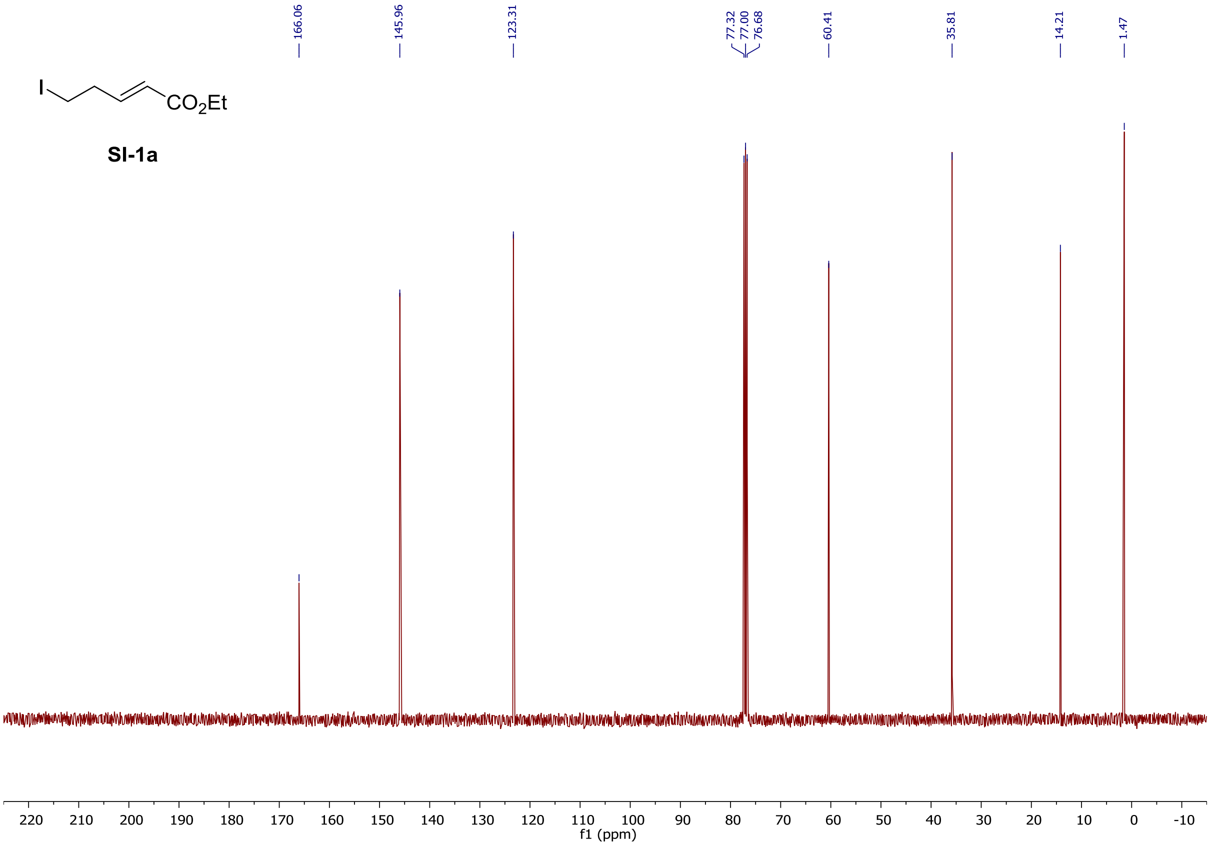

^{1}H NMR (400 MHz, $CDCl_3$)

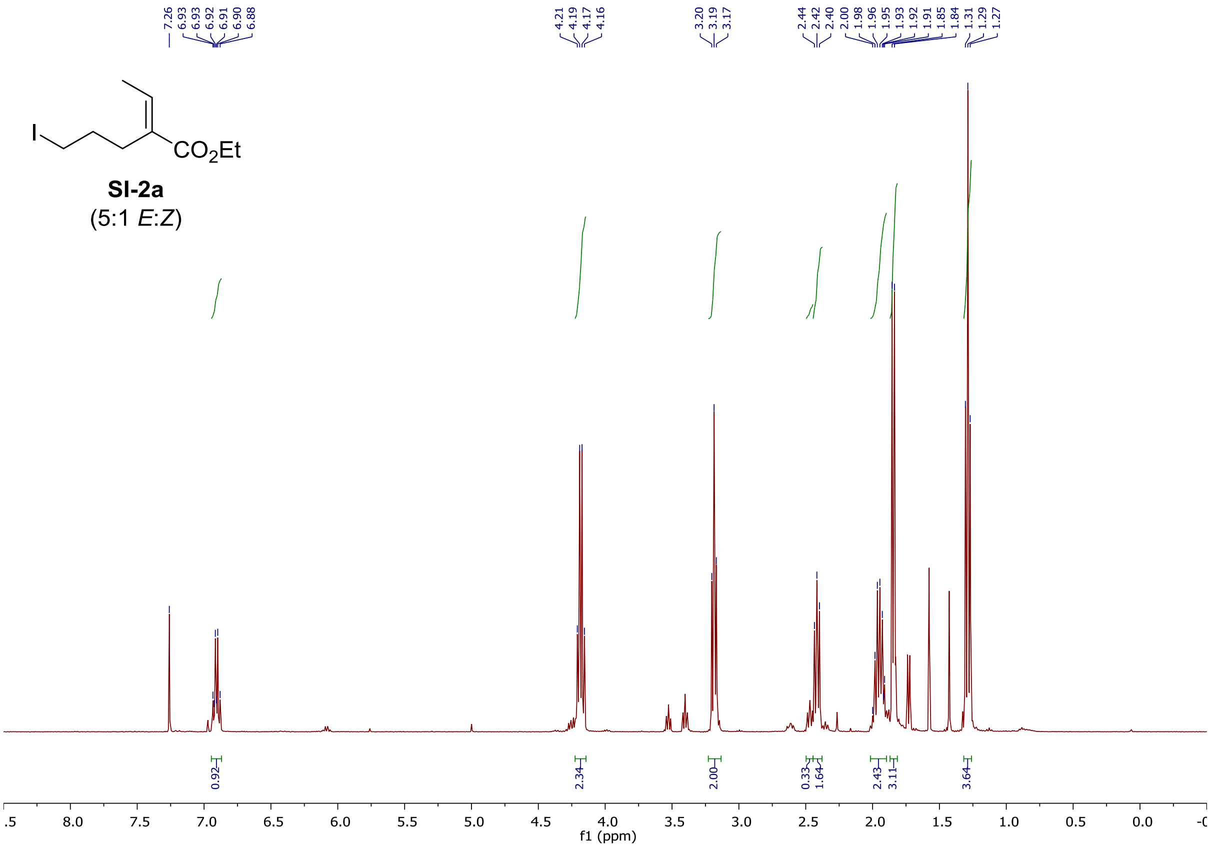

^{13}C NMR (101 MHz, $CDCl_3$)

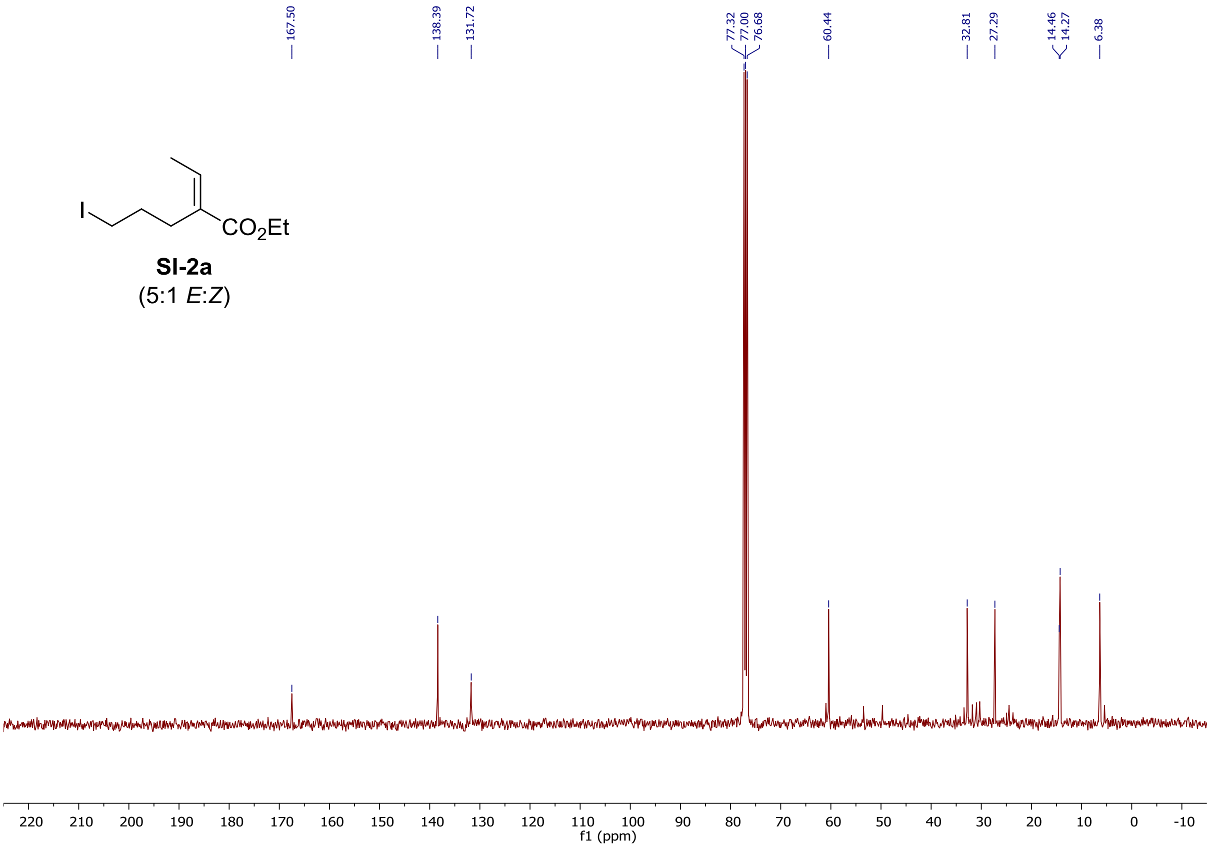

^{1}H NMR (400 MHz, DMSO-d_6)

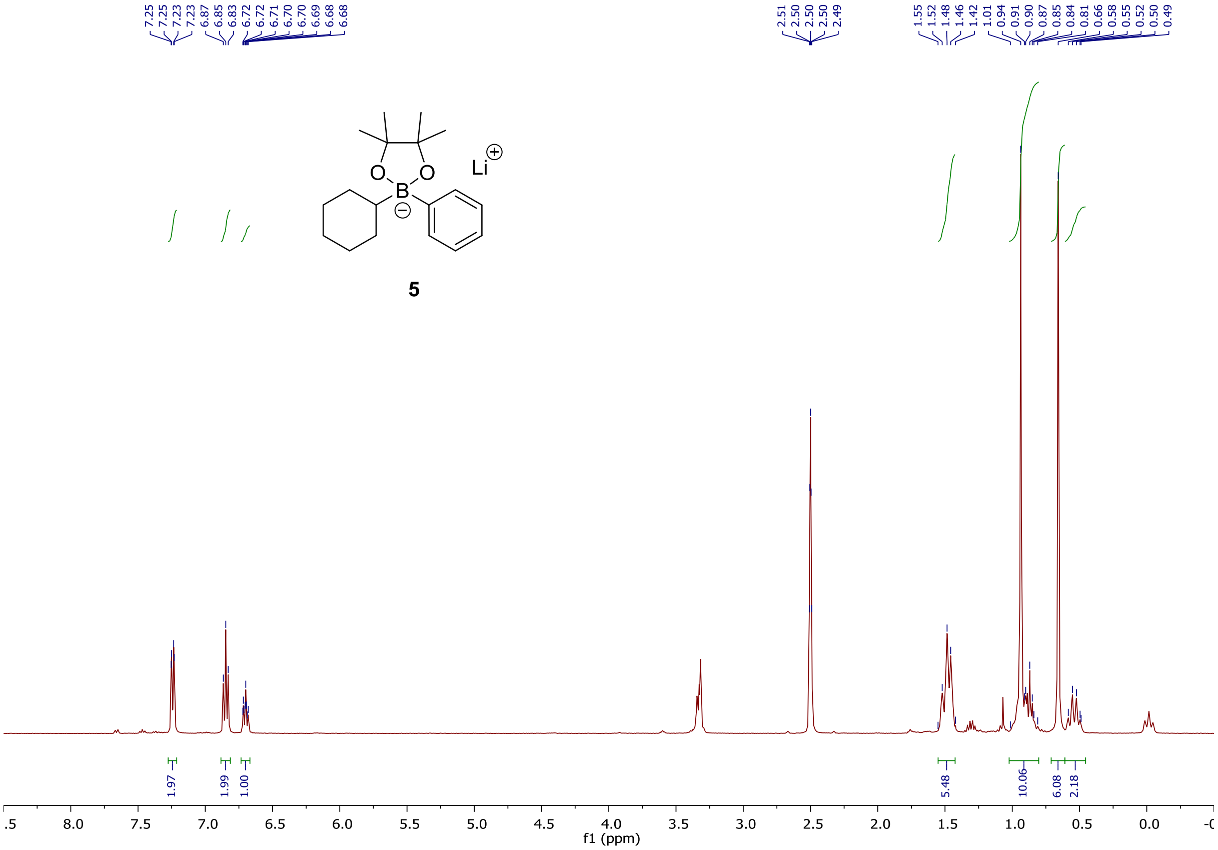

^{13}C NMR (101 MHz, 400 MHz, DMSO-d_6)

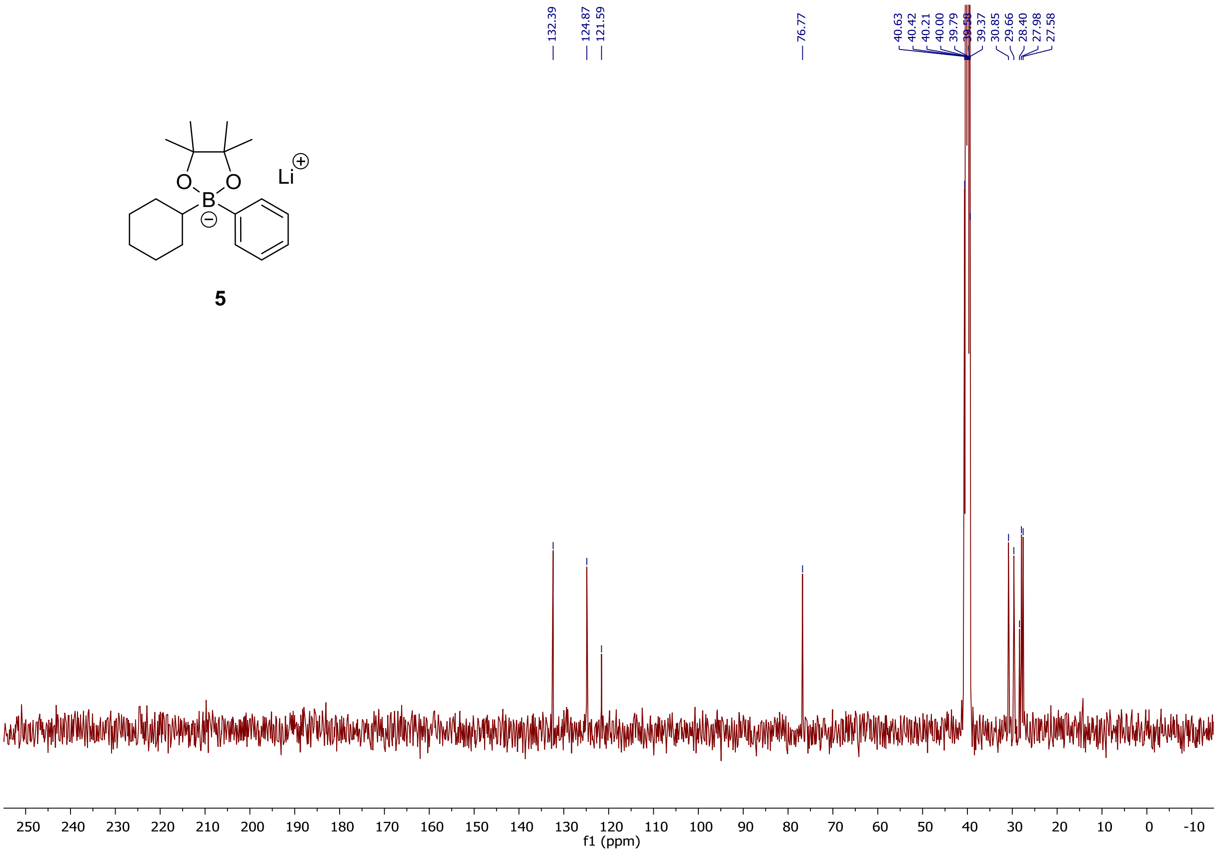

^{1}H NMR (400 MHz, $CDCl_3$)

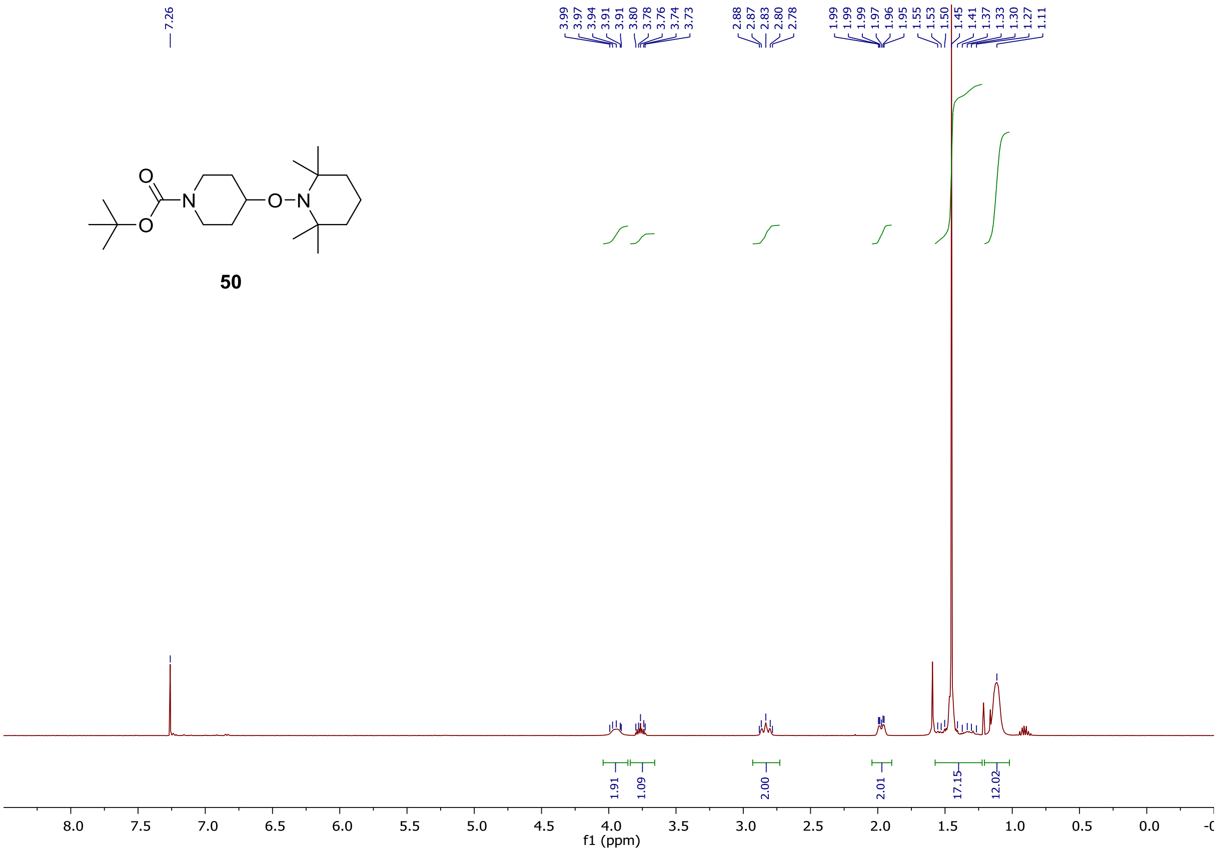

^{13}C NMR (101 MHz, $CDCl_3$)

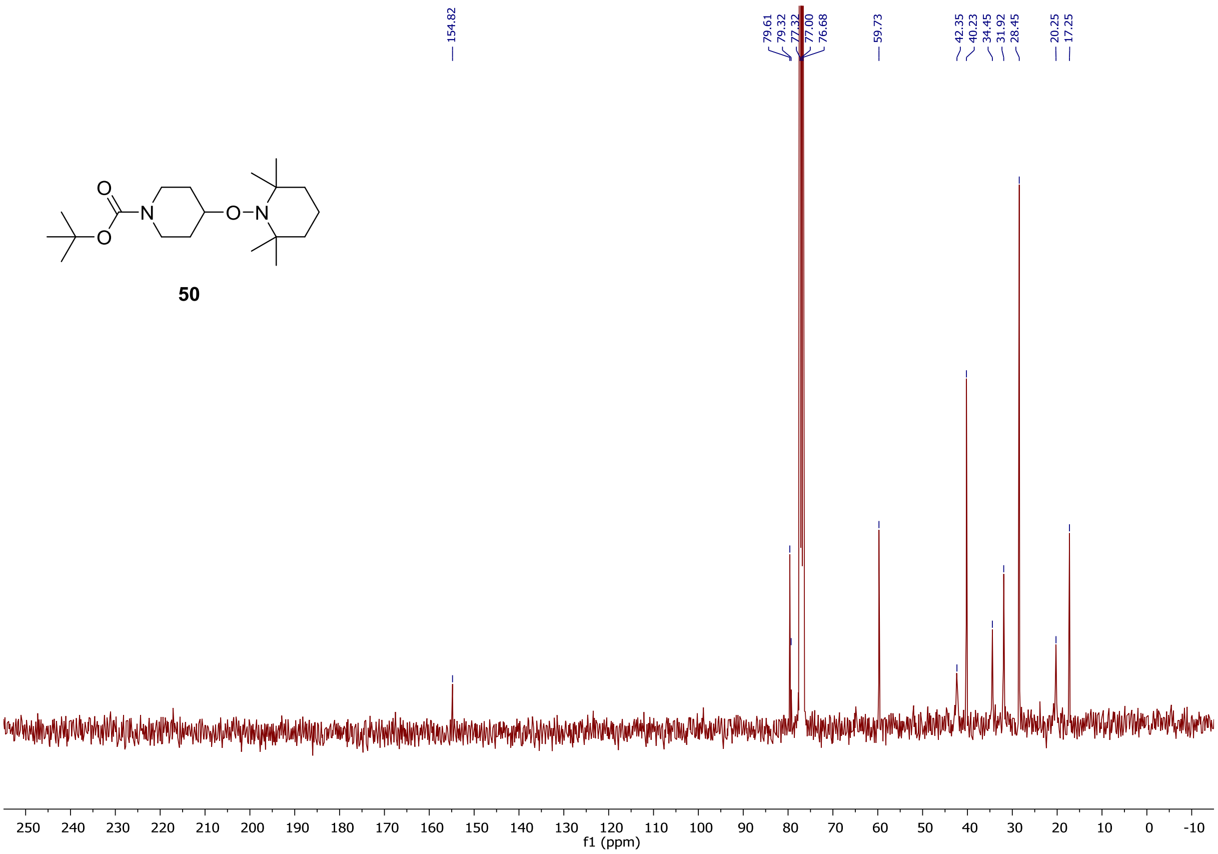

^{1}H NMR (400 MHz, $CDCl_3$)

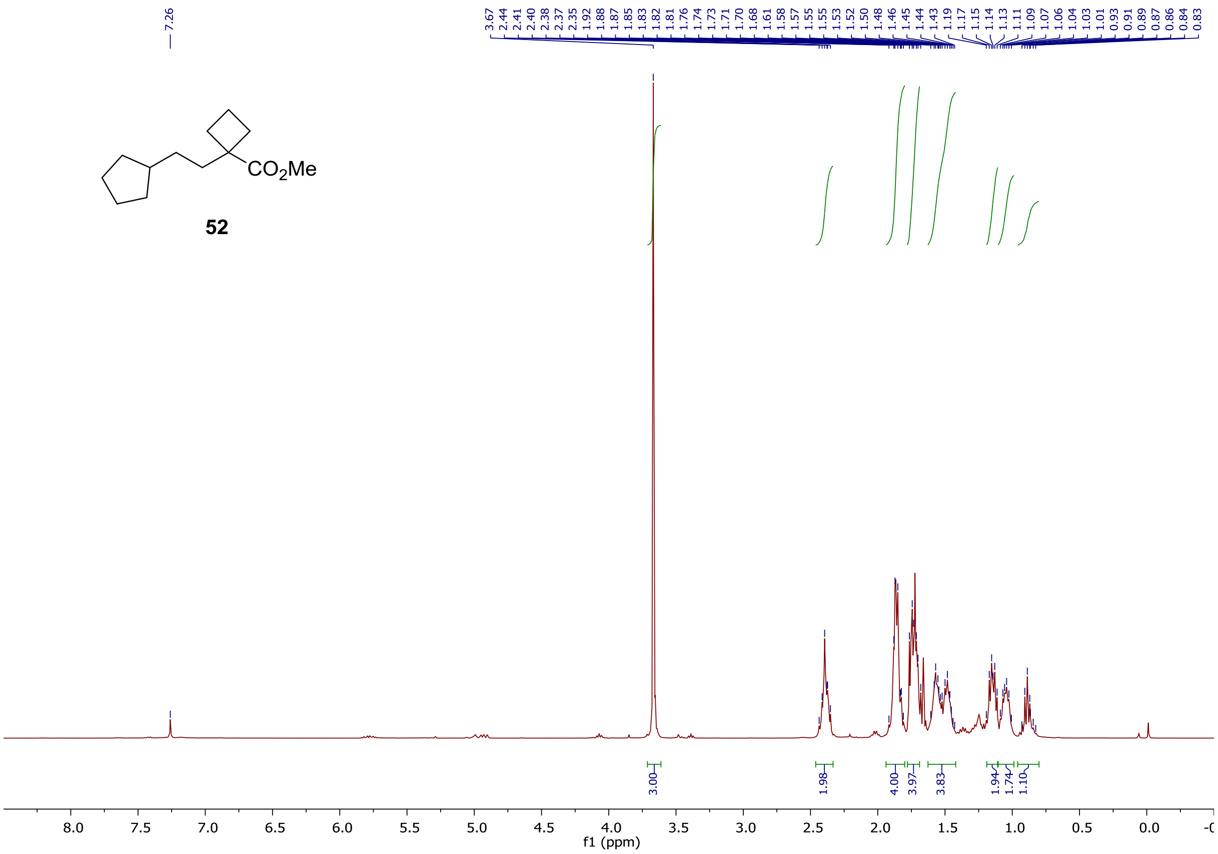

^{13}C NMR (101 MHz, $CDCl_3$)

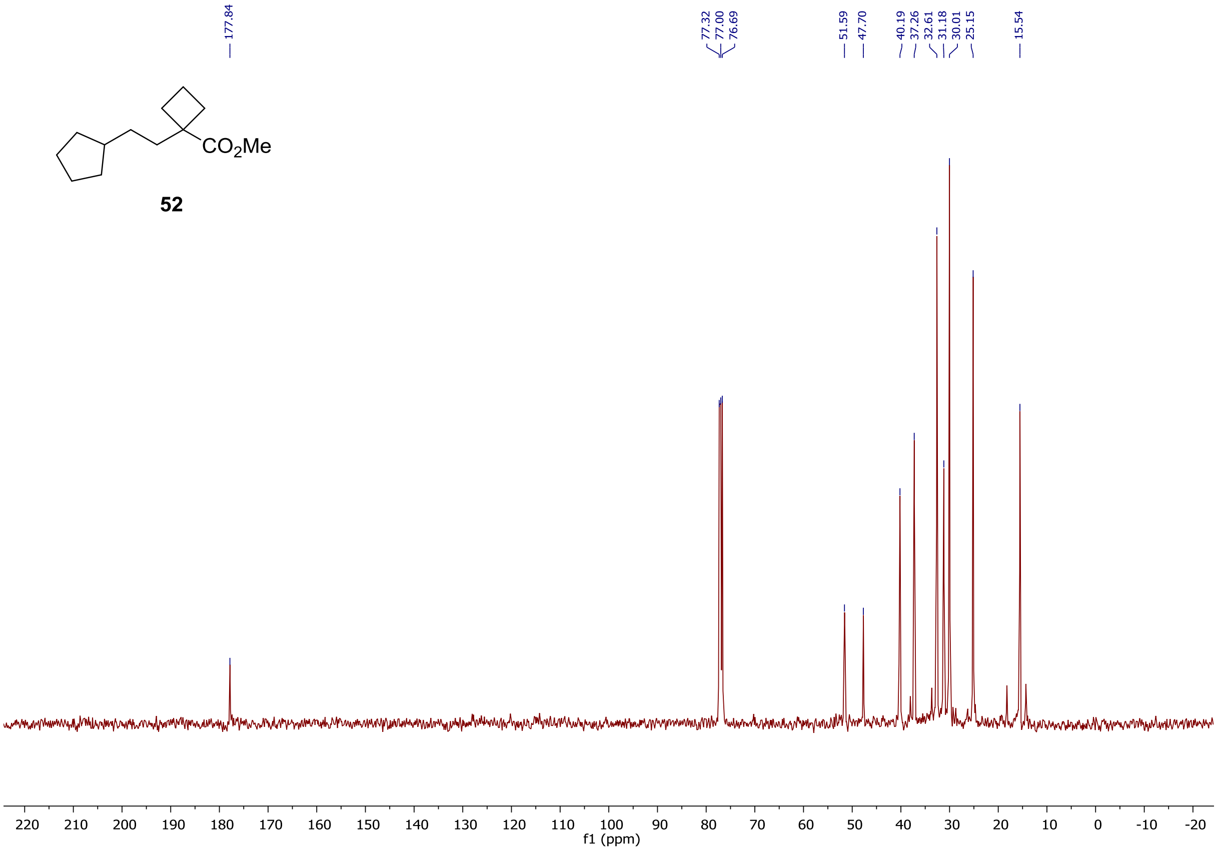

^{1}H NMR (400 MHz, $CDCl_3$)

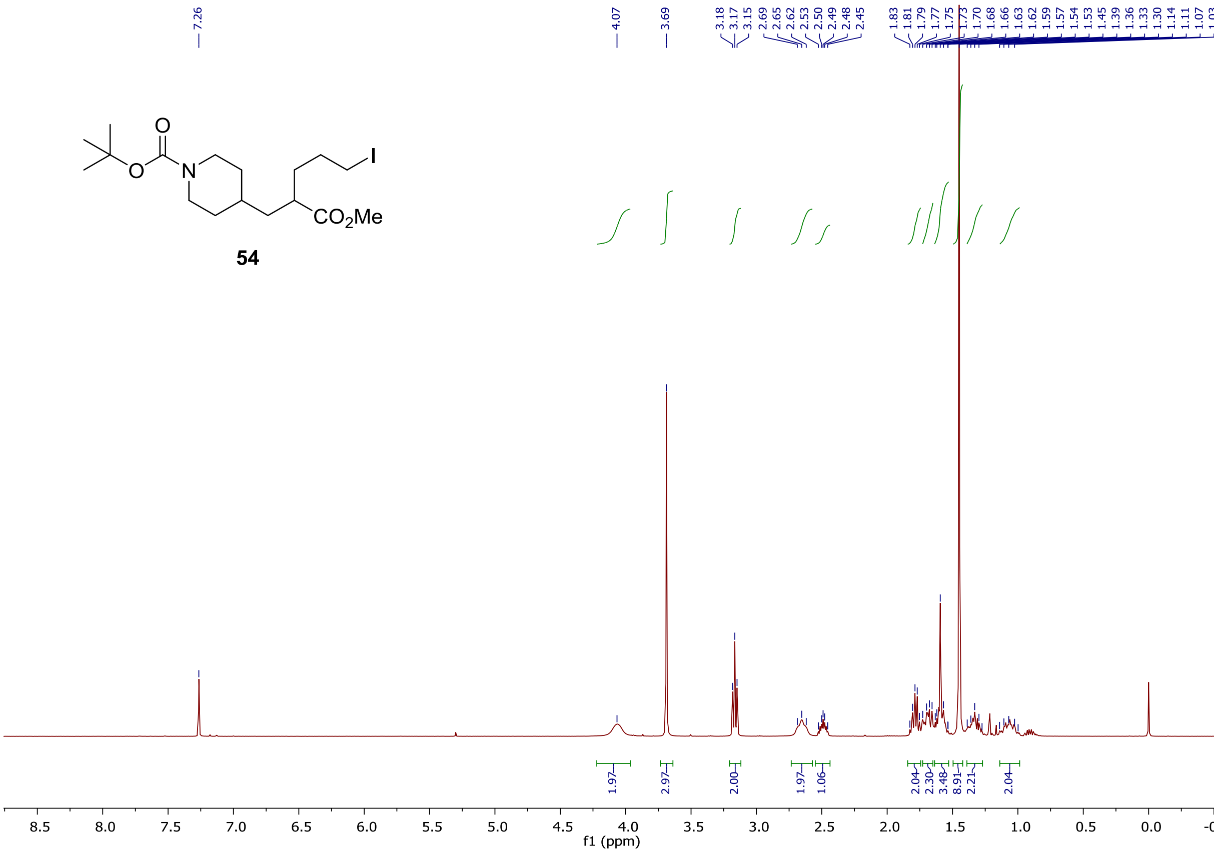

^{13}C NMR (101 MHz, $CDCl_3$)

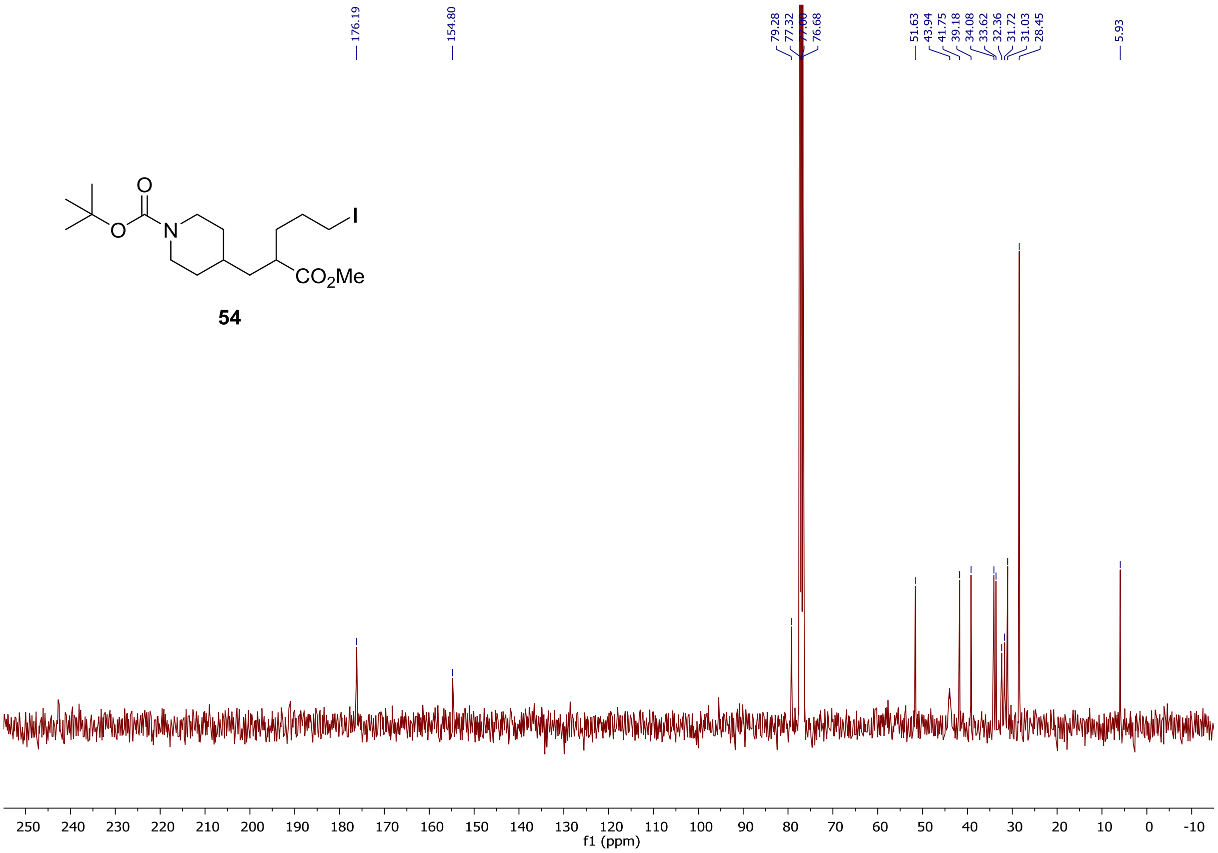

^1H NMR (400 MHz, $CDCl_3$)

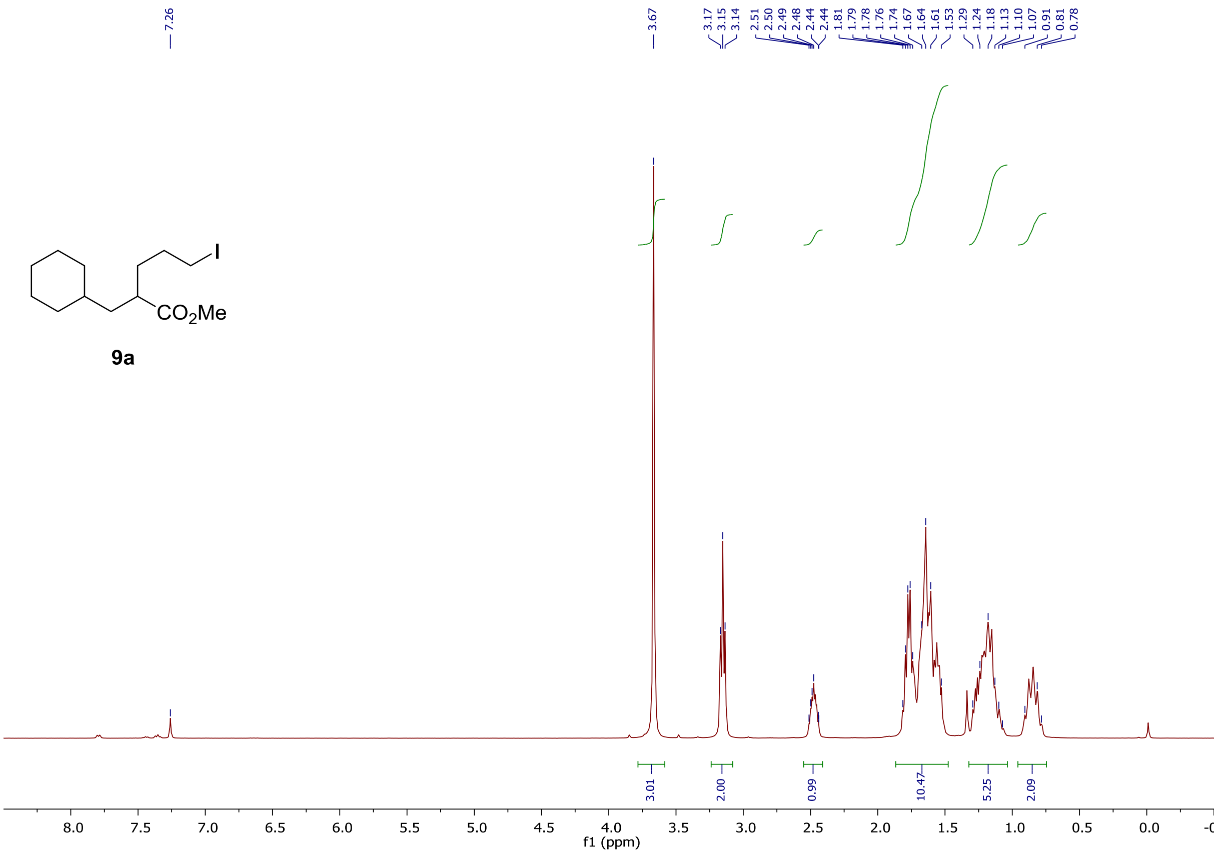

^{13}C NMR (101 MHz, $CDCl_3$)

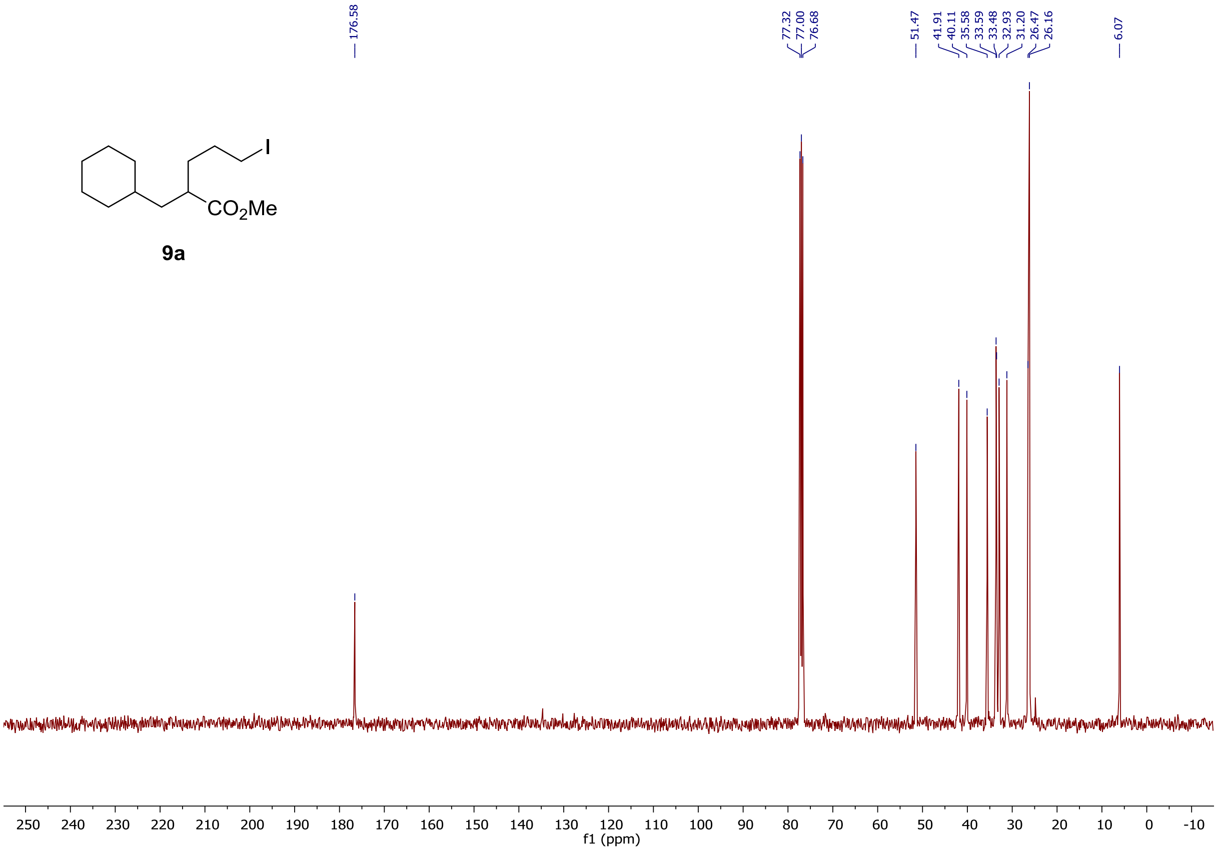

^{1}H NMR (400 MHz, $CDCl_3$)

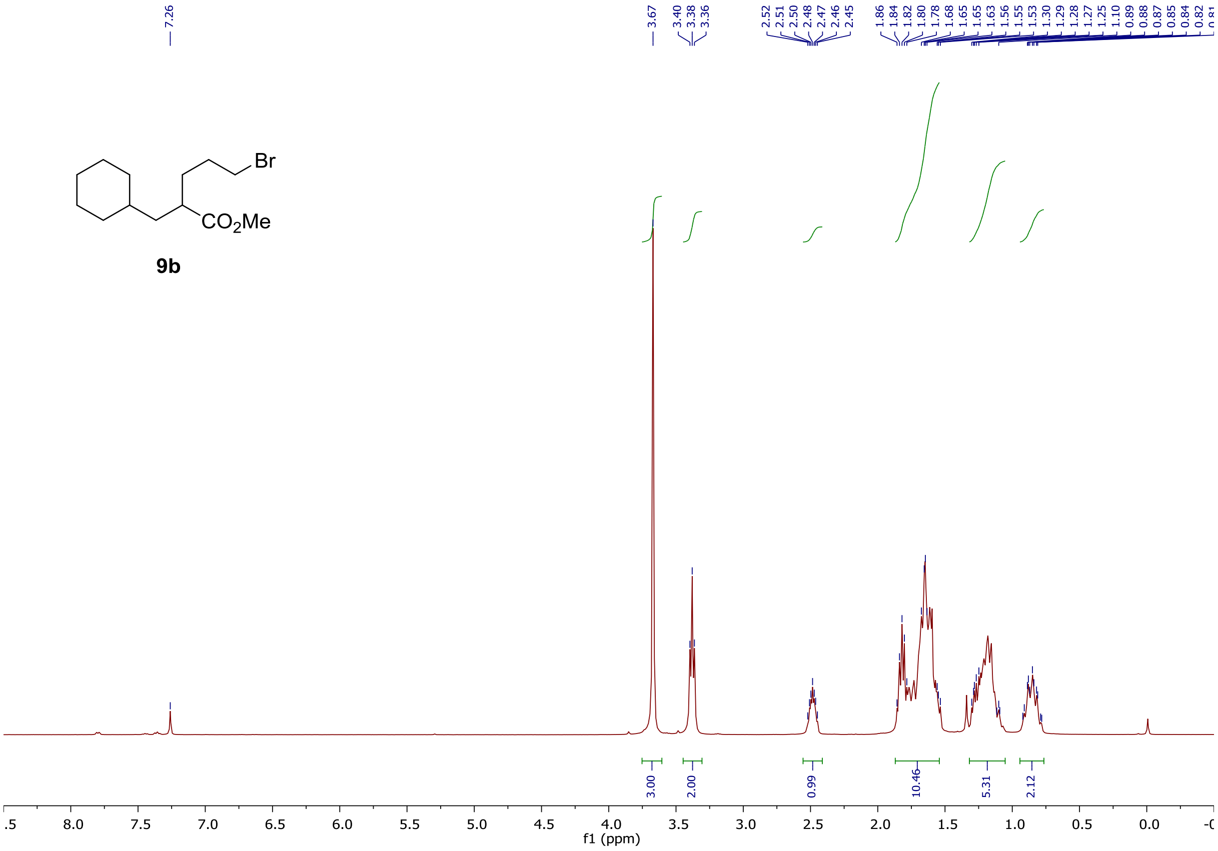

^{13}C NMR (101 MHz, $CDCl_3$)

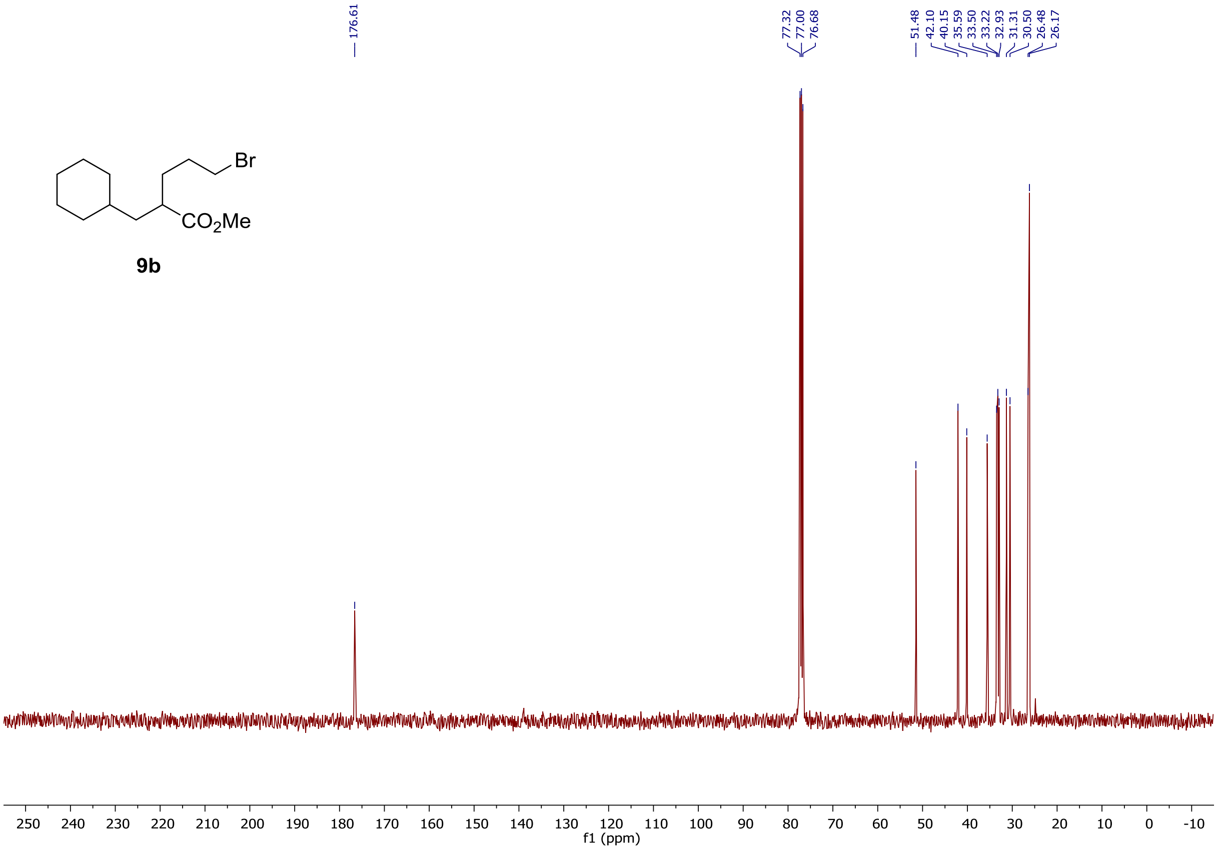

^{1}H NMR (400 MHz, $CDCl_3$)

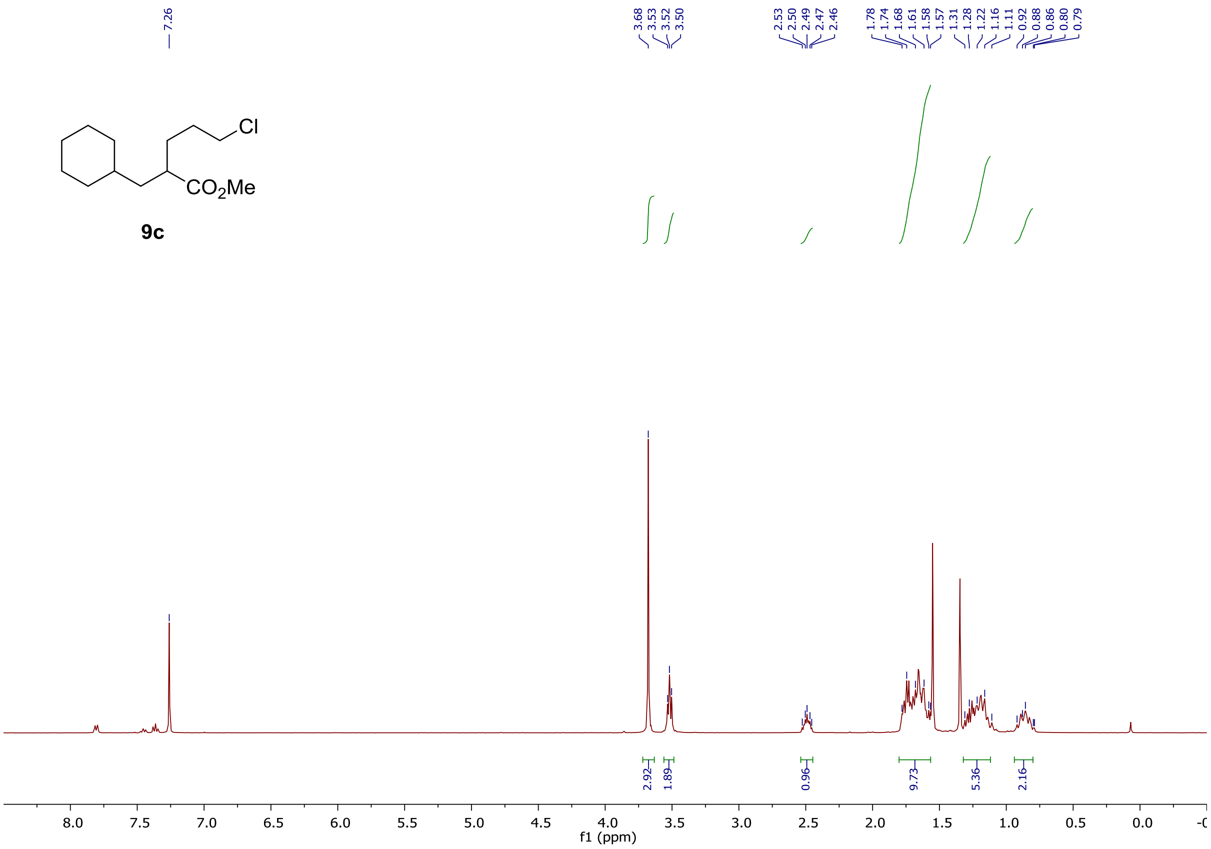

^{13}C NMR (101 MHz, $CDCl_3$)

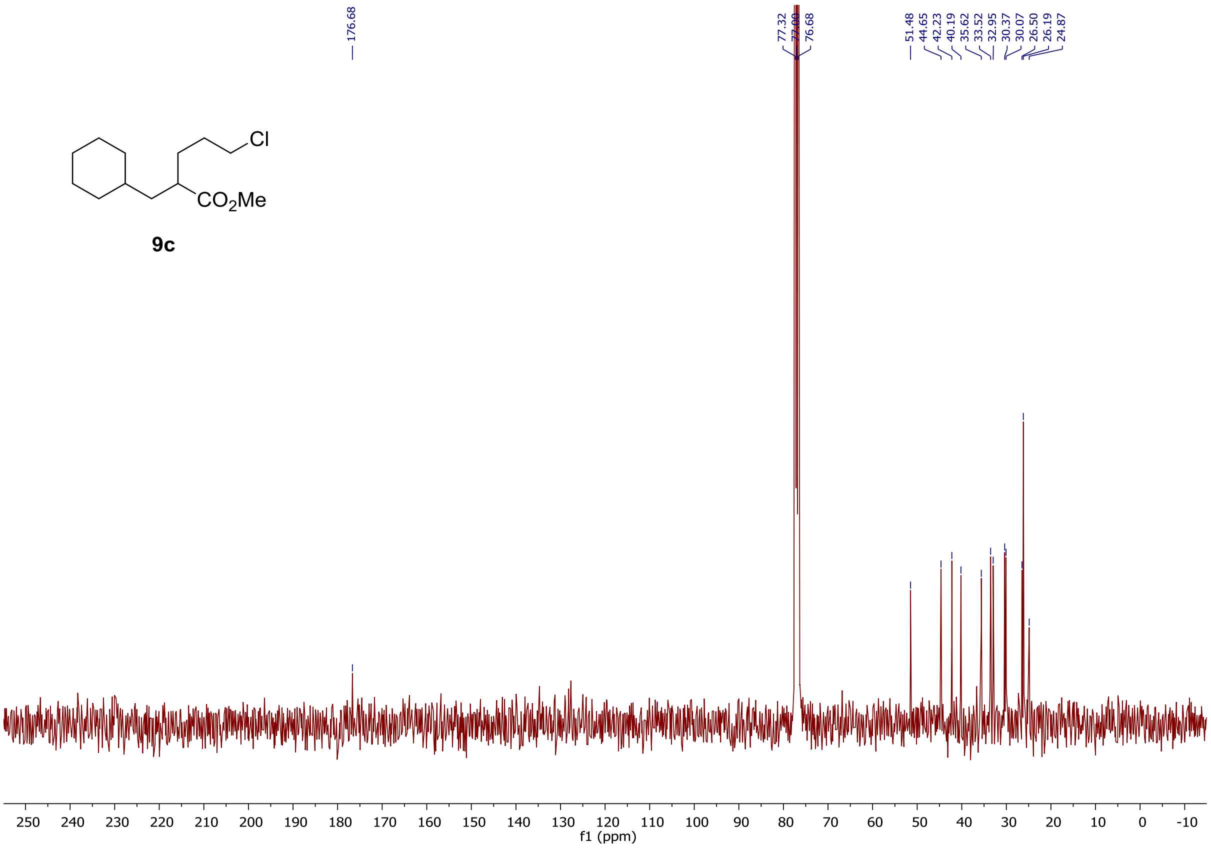

^{1}H NMR (400 MHz, $CDCl_3$)

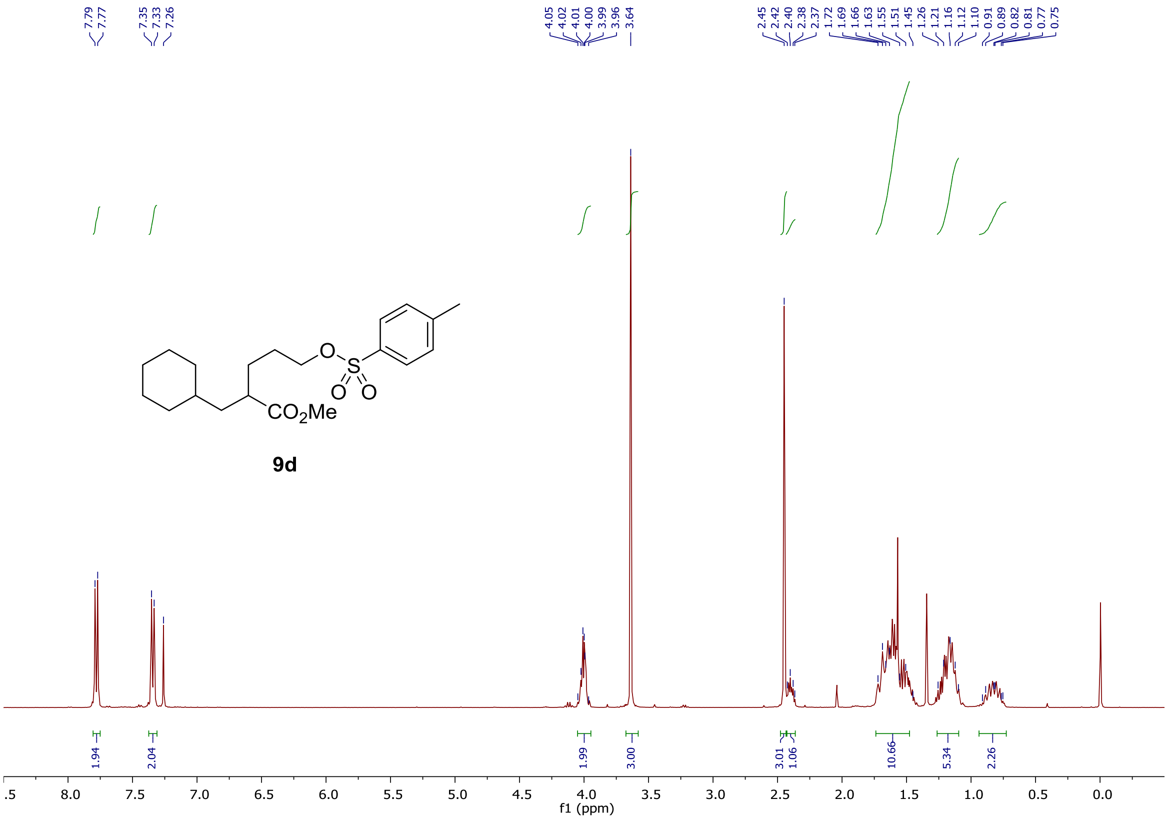

^{13}C NMR (101 MHz, $CDCl_3$)

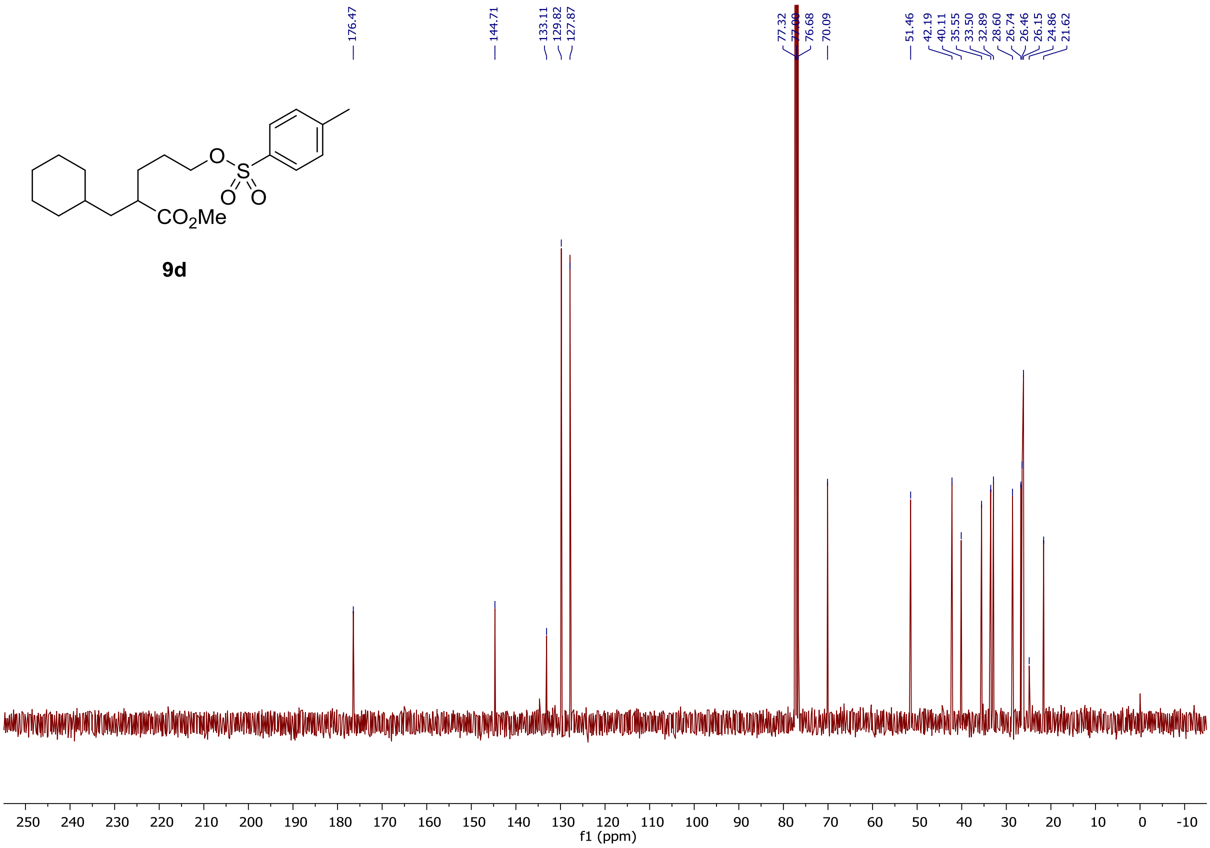

Supplement: Supplementary file 1 — Supplementary [file ANIE-58-3870-s001.pdf]
